# Fully Aqueous and Air-Compatible Cross-Coupling of Primary Alkyl Halides with Aryl Boronic Species: A Possible and Facile Method

**DOI:** 10.1021/acscatal.3c00252

**Published:** 2023-04-24

**Authors:** Samuel Molyneux, Rebecca J. M. Goss

**Affiliations:** School of Chemistry, University of St Andrews, North Haugh, St Andrews, Fife KY16 9ST, U.K.

**Keywords:** aqueous, cross-coupling, sustainable, biocompatible, late-stage diversification

## Abstract

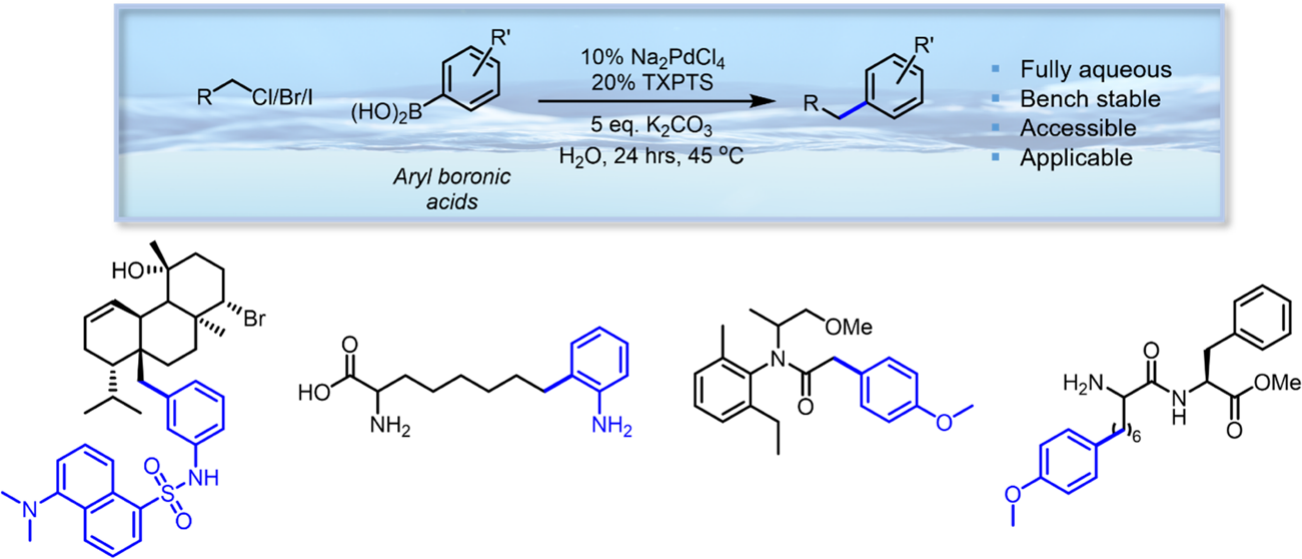

Aqueous transformations confer many advantages, including decreased environmental impact and increased opportunity for biomolecule modulation. Although several studies have been conducted to enable the cross-coupling of aryl halides in aqueous conditions, until now a process for the cross-coupling of primary alkyl halides in aqueous conditions was missing from the catalytic toolbox and considered impossible. Alkyl halide coupling in water suffers from severe problems. The reasons for this include the strong propensity for β-hydride elimination, the need for highly air- and water-sensitive catalysts and reagents, and the intolerance of many hydrophilic groups to cross-coupling conditions. Here, we report a broadly applicable and readily accessible process for the cross-coupling of water-soluble alkyl halides in water and air by using simple and commercially available bench-stable reagents. The trisulfonated aryl phosphine TXPTS in combination with a water-soluble palladium salt Na_2_PdCl_4_ allowed for the Suzuki–Miyaura coupling of water-soluble alkyl halides with aryl boronic acids, boronic esters, and borofluorate salts in mild, fully aqueous conditions. Multiple challenging functionalities, including unprotected amino acids, an unnatural halogenated amino acid within a peptide, and herbicides can be diversified in water. Structurally complex natural products were used as testbeds to showcase the late-stage tagging methodology of marine natural products to enable liquid chromatography–mass spectrometry (LC–MS) detection. This enabling methodology therefore provides a general method for the environmentally friendly and biocompatible derivatization of sp^3^ alkyl halide bonds.

## 1 Introduction

**Figure 1:**
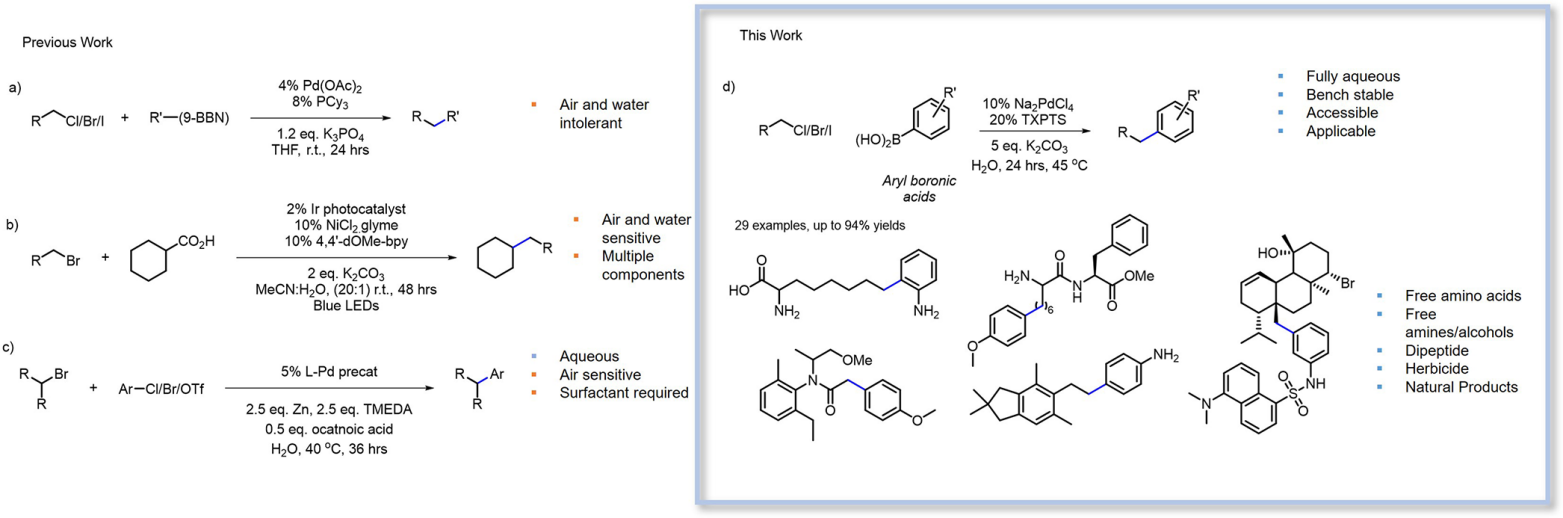
(a) Organic solvent-phase C(sp^3^)–X primary alkyl halide Suzuki–Miyaura coupling using alkyl phosphine ligands.^5^ (b) Water-tolerant C(sp^3^)–Br primary alkyl bromide metallophotoredox coupling.^16^ (c) Micellar-based aqueous C(sp^3^)–Br primary and secondary alkyl bromide Lipshutz–Negishi coupling.^34^ (d) This work: fully aqueous general C(sp^3^)–X primary alkyl halide coupling. Examples highlight the functionalities generated through this method.

The metal-catalyzed cross-coupling of carbon–halogen bonds with organometallic reagents, one of the most important reactions of the 20th century, was recognized through the award of the Nobel Prize in 2010.^1^ Its application to the formation of new carbon–carbon bonds is instrumental in enabling the synthesis and diversification of numerous active agents, from small-molecule drug targets^2^ and complex natural products to biomolecules.^3^ Although most studies have focused on the more electron-poor sp^2^ aryl halide electrophiles, over the last 10 years, attention has been diverted to the much needed, but perceived as more challenging, cross-coupling of sp^3^ alkyl halides. Drug candidates that have escaped from the medicinal chemistry flatlands of aryl and heteroaryl systems, adopting the more three-dimensional structures that higher sp^3^ bond counts and chiral centers exhibit, have met with clinical success^4^ and have driven both the need and desire for new chemistries that can be used to access or modulate these systems. Novel methodologies enabling sp^3^–sp^2^ or sp^3^–sp^3^ bond formation have adapted palladium (Figure 1a),^5−8^ nickel,^9−11^ and iron catalysis,^12−14^ as well as more complex but highly powerful metallophotoredox chemistries (Figure 1b).^15,16^ These methods leverage electron-rich metal ligands to enable the oxidative addition of electron-rich C(sp^3^)–X bonds, while placing steric bulk around the metal center to avoid β-hydride elimination reactions and the formation of unwanted alkene side products.^17^ For secondary alkyl halides, the catalytic system assists in the stabilization of radical intermediates.^18^

The scope of the research into aqueous cross-coupling continues to expand rapidly, with one of the preliminary driving forces for aqueous cross-coupling being the crucial foundation of robust and mild conditions in bioorthogonal settings. Great successes in this area have enabled *in vivo* natural product derivatization,^19,20^ site-selective protein and biomolecule labeling,^21−25^ prodrug activation,^26−28^ and one-pot C–H activation combining bio- and metal catalysis.^29,30^ These aqueous reactions have also been considered as a step forward to more green processes of cross-coupling, moving away from conditions dependent on toxic and unsustainable organic solvents.^31^ Although there are a growing number of procedures enabling the cross-coupling of alkyl halides in organic solvents,^32^ there are no general methods for the cross-coupling of alkyl halides in water, as current methods often require the rigorous exclusion of both air and moisture and are unable to tolerate hydrophilic functionalities with free protons. As the initial investigations into water-active catalysts for aryl halide coupling led to a plethora of further applications, it is obvious that there is a need for the cornerstones of aqueous alkyl halide coupling to be set, enabling access to more 3D and sensitive molecules under mild and green chemistry.^33^

However, cross-coupling of alkyl halides often requires more active catalysts or organometallic coupling partners than those required for aryl halide couplings, such as alkyl phosphine ligands and alkyl borane species (Figure 1a), and these higher energy catalysts tend to be prone to degradation in the presence of air and water. Encouragingly, the Lipshutz–Negishi-type coupling of alkyl halides with aryl electrophiles and *insitu* organozinc formation have been demonstrated using surfactants to enable micellar reactivity using palladium catalysis (Figure 1c),^34,35^ but, in general, the functional group tolerance is poor. This micellar method has found a great deal of success with iron catalysis, enabling sp^3^–sp^3^ bond formation between complex molecules.^36^ Yet, this methodology is incompatible with many functional groups, notably acidic protons, and requires a large amphiphile to enable micelle formation; as such, it is highly unsuited as a base for bioorthogonal chemistry. To address the need for greener methodologies for C(sp^3^)–X alky halide cross-coupling that could be used when modulating compounds containing sensitive functional groups or even biomolecules, we explored various catalysts and ligands. We demonstrate that Na_2_PdCl_4_ and TXPTS could be employed in a fully aqueous reaction, even in the presence of air, to enable the cross-coupling of primary alkyl halides with aryl boronic species (Figure 1d).

## 2 Results and Discussion

### 2.1 Initial Screening

Aliphatic amino acids are highly challenging testbed substrates for aqueous Suzuki–Miyaura coupling, with low solubility in organic solvents and modest solubility in water. Both the amine and carboxy groups have a propensity to coordinate to palladium, often competing with the active catalyst ligand, and free protons often interfere with more sensitive catalysts and reagents.^19^ We find that starting with the bar set high paves the way for identifying conditions that may readily be translated as a general method for functionalizing a broad suite of compounds. Therefore, the potential for the Suzuki–Miyaura cross-coupling of a water-soluble unnatural brominated amino acid (**1a**) was explored in the first instance (Table 1). Aryl boronic acids were examined as initial coupling partners due to their general water solubility in basic conditions and commercial availability. First, coupling reactions were explored with *para*-methoxyphenyl boronic acid (**2a**), using 10% Pd(OAc)_2_ and 5 equiv of NaOH.

**Table 1:**
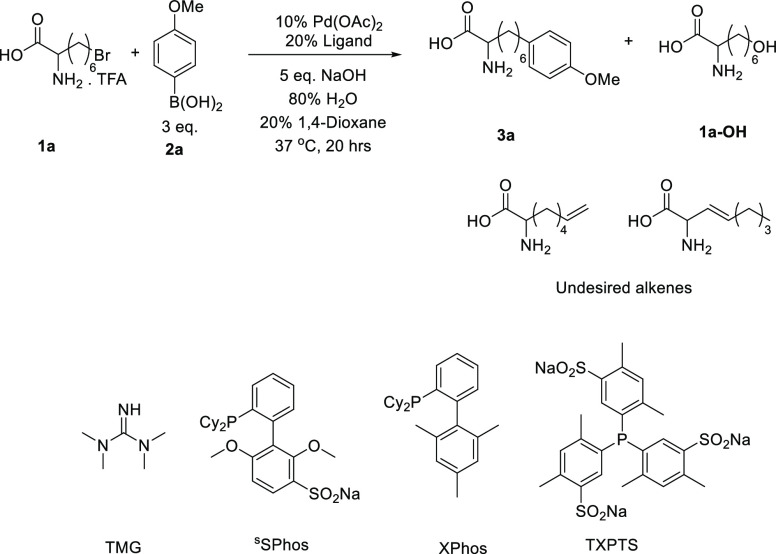
Catalyst Screening for Aqueous Alkyl Halide Coupling^a^

entry	variations to the conditions	yield^b^	alkene^b^
1	[HP^t^Bu_2_Me][BF_4_]	5%	0%
2	[HPCy_3_][BF_4_]	0%	0%
3	[HP^t^Bu_3_][BF_4_]	0%	0%
4	TMG	0%	0%
5	^s^SPhos	21%	44%
6	XPhos	0%	0%
7	TXPTS	50%	3%
8	TXPTS, Na_2_PdCl_4_	34%	0%
9	TXPTS, Na_2_PdCl_4_, 100% H_2_O	51%	0%
10	TXPTS, Na_2_PdCl_4_, 100% H_2_O, in air	61%	0%
11	TXPTS, Na_2_PdCl_2_, 100% H_2_O, in air, K_2_CO_3_, 45 °C, 24 h	84%	0%

a Total solvent volume, 0.6 mL; halide, 0.1 mmol; boronic acid, 0.3 mmol.

b NMR yields reported are determined in comparison to the internal standard 1,4-dimethoxybenzene (0.0667 mmol, 6.9 mg) in the crude sample.

The first reported application of the Suzuki–Miyaura cross-coupling to unfunctionalized alkyl halides employed alkyl phosphine ligands in rigorous degassed and dry systems,^37^ and this provided one starting point for our explorations into determining and developing a methodology for the aqueous cross-coupling of alkyl halides. Using the same catalysts and ligands, we explored the tolerance of the cross-coupling reaction to high concentrations of water. Pd(OAc)_2_ and [HPCy_3_][BF_4_] or [HP^t^Bu_3_][BF_4_] were explored as ligands in the 80% aqueous reaction of **1a** and **2a**; however, only unreacted starting material could be observed (entries 2 and 3; Table 1). Promisingly, when [HP^t^Bu_2_Me][BF_4_] was used as a ligand, a small amount of the desired cross-coupled product could be determined (5% yield as determined by NMR) (entry 1, Table 1).

Aqueous cross-coupling of C(sp^2^)–X aryl halides provided a parallel starting point in our search for conditions to establish and develop the aqueous cross-coupling of C(sp^3^)–X alkyl halides. Both tetramethylguanidine (TMG) and X-Phos are well known and highly useful ligands for the aqueous Suzuki–Miyaura coupling of aryl halides;^19^ however, when explored in the context of alkyl halides, no conversion to the coupled product was observed (entries 4 and 6; Table 1). Ligands with greater solubility were next explored; pleasingly, the sulfonated ligands ^s^SPhos and TXPTS afforded the desired reaction (21 and 50% yields, as determined by NMR, respectively); however, the employment of ^s^SPhos also resulted in considerable β-hydride elimination and the generation of alkene side products (44% by NMR). Gratifyingly, TXPTS, a simple sulphonated triarylphosphine,^38^ led to minimal alkene formation. A combination of high steric bulk, electron richness at the phosphine center, and water solubility led TXPTS to enable high conversions in aqueous conditions and, under these conditions, prohibit β-hydride elimination (entry 7, Table 1) and enable coupling in the presence of this notoriously tricky free amino acid functionality. To further leverage the beneficial impact of sterics and water solubility, TXPTS was explored in combination with a water-soluble palladium species. It was found that in fully aqueous conditions, by using a water-soluble palladium source in conjunction with TXPTS, (entry 10, Table 1), an NMR yield of 61% of our desired product was achieved for this initial reaction, providing a good starting point for further optimization. To the best of our knowledge, this is the first example of a fully aqueous homogeneous metal-catalyzed Suzuki–Miyaura cross-coupling of alkyl bromides. Furthermore, there is no need to employ rigorous degassing as the reaction can be run open to the air. The reaction proceeds, within a reasonable time frame (20 h), at 37 °C, paving the way for the development of this methodology toward biocompatibility.

With 61% yield using TXPTS as the ligand, we observed full consumption of the starting material. We identified alcohol **1a-OH** as the undesired but readily separable side product present in all of our catalyst screening reactions. To investigate whether we could suppress the competing hydrolytic route, we next turned our attention toward the exploration of bases less harsh than sodium hydroxide (see the Supporting Information, SI, Table S1, for the full screening). Generally, as anticipated, lower yields were observed when stronger bases were used, presumably a consequence of the SN2 hydrolysis of the alkyl bromide to the primary alcohol. Less nucleophilic and milder bases reduced this side reaction and resulted in higher yields. It was found that the employment of inorganic bases, in particular, sodium, potassium, and cesium carbonate, could enhance yields. NMR yields of 22% could be observed when K_3_PO_4_ was used; this is promising and affords future opportunities for the employment of phosphates as both biological buffers and bases in potential biorthogonal cross-coupling reactions. For the purpose of developing a robust, yet mild, set of conditions, potassium carbonate was taken forward as the best candidate, enabling yields of 68%. Increasing the temperature to 45 °C and reaction time to 24 h led to a good NMR yield of 84% (isolated yield 71%) for this challenging brominated amino acid substrate.

### 2.2 Mechanistic Discussion

**Figure 2:**
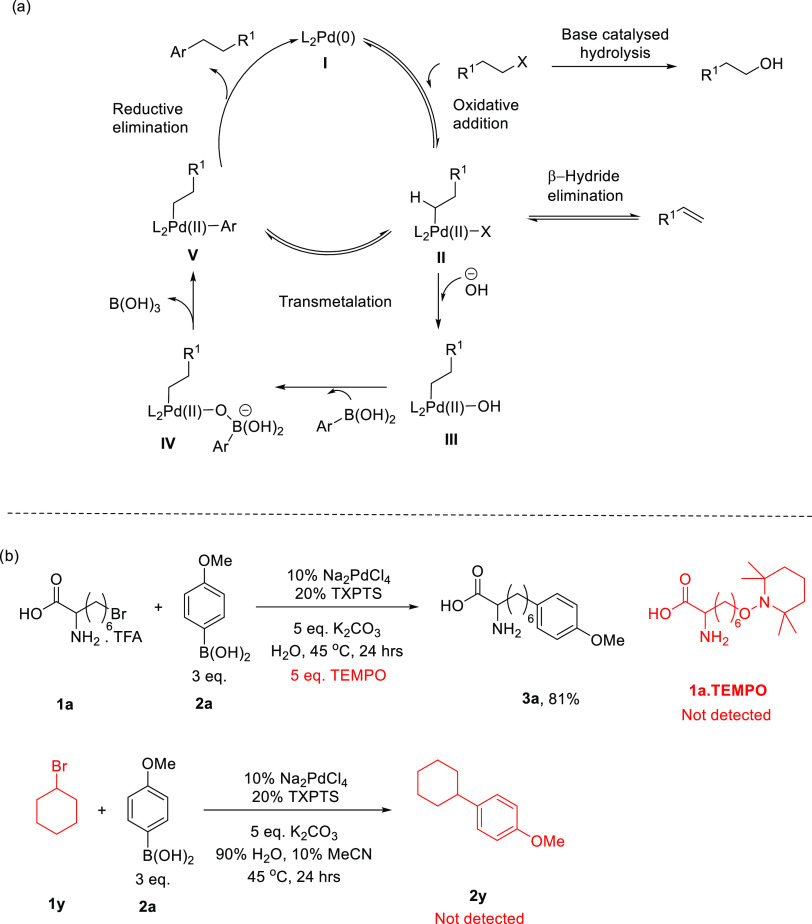
(a) Generally accepted mechanism of the Suzuki–Miyaura cross-coupling as applied to alkyl halides, showing competing reactions of hydrolysis and β-hydride elimination. (b) Experiments into the potential radical nature of the reaction. Total solvent volume, 0.6 mL; halide, 0.1 mmol; boronic acid, 0.3 mmol; and TEMPO, 0.5 mmol. NMR yields reported are determined in comparison to the internal standard 1,4-dimethoxybenzene (0.0667 mmol, 6.9 mg) in the crude sample.

As discussed above, alkene and alcohol **1a-OH** were observed as the prominent undesired products. In control experiments (see Table 1), the primary bromide was converted almost completely to **1a-OH** without the TXPTS ligand and palladium catalyst, indicating that this competing reaction is likely base catalyzed rather than palladium catalyzed. While the catalyst loading can be reduced to 5 mol% and still enable coupling, the yield is lowered significantly to 50%, with a much higher proportion of **1a-OH**, indicating a direct relation between the concentration of the catalyst and the rate of coupling compared to hydrolysis. We found that the cross-coupling is generally faster, thus outcompeting the alcohol-forming side reaction at higher temperatures with potassium carbonate. It is worth noting that at room temperature, conversions to both the product and the alcohol side product were very low, indicating a need for employment of higher temperatures for both reactions. We can consider multiple competing pathways for the coupling of primary alkyl bromides in aqueous conditions: alcohol formation from alkyl bromide competing with the “SN2 type” oxidative addition of palladium species **I** (Figure 2),^39^ and the β-hydride elimination in competition with transmetalation with boron species to **V**. In our screening reactions, we show that ^S^SPhos allows for the oxidative addition of alkyl halides, yet almost all of this halide is converted to alkene. We therefore postulate that the TXPTS ligand is the key cause of β-hydride elimination inhibition.

Whereas an SN2-type oxidative addition is generally the accepted mechanism for the Suzuki–Miyaura couplings of primary alkyl halides, to rule out a radical mechanism, the standard reaction conditions were doped with radical trap TEMPO. If **1a** proceeded by a radical oxidative addition mechanism, the addition of excess TEMPO would lead to trapped **1a.TEMPO**. Under these conditions, **1a.TEMPO** was not observed by liquid chromatography–mass spectrometry (LC–MS), and the yield of the reaction was not reduced, indicating the lack of radical formation within species **I** oxidative addition. This lack of radical reactivity is thought to be the reason for the limited success of palladium catalysis toward secondary alkyl halide cross-coupling. Most secondary halide couplings involve metals that have access to a greater number of stable oxidation states, such as nickel, enabling radical-type mechanisms. When the secondary halide bromocyclohexane **1y** was subjected to our cross-coupling conditions, we observed no conversion to product **2y** or alkene, indicating again a lack of radical oxidative addition within our mechanism and that our conditions can be used to regioselectively react primary alkyl halides.

We propose a mechanism here involving palladium-coordinated hydroxide from basic aqueous media involving oxo-palladium transmetalation (Figure 2, species **II**–**V**).^40^ However it is worth noting that the majority of the Suzuki–Miyaura mechanistic studies have been performed on the cross-coupling of aryl halides and boronic acids in purely organic or biphasic systems,^41^ very different to this alkyl halide aqueous system. Water has been shown to have a profound effect on coupling reactions^42,43^ and yet is partitioned away from the organic catalytic cycle in previous proposed mechanisms; because of the hydrophobic nature of aryl halides and boronic acids, the Suzuki reactions in pure water are still considered biphasic.^44,45^ A full mechanistic investigation of this unique system of water-soluble catalyst and alkyl halide substrates would be highly complex, and as such is outside the scope of this project.

### 2.3 Scope

**Figure 3:**
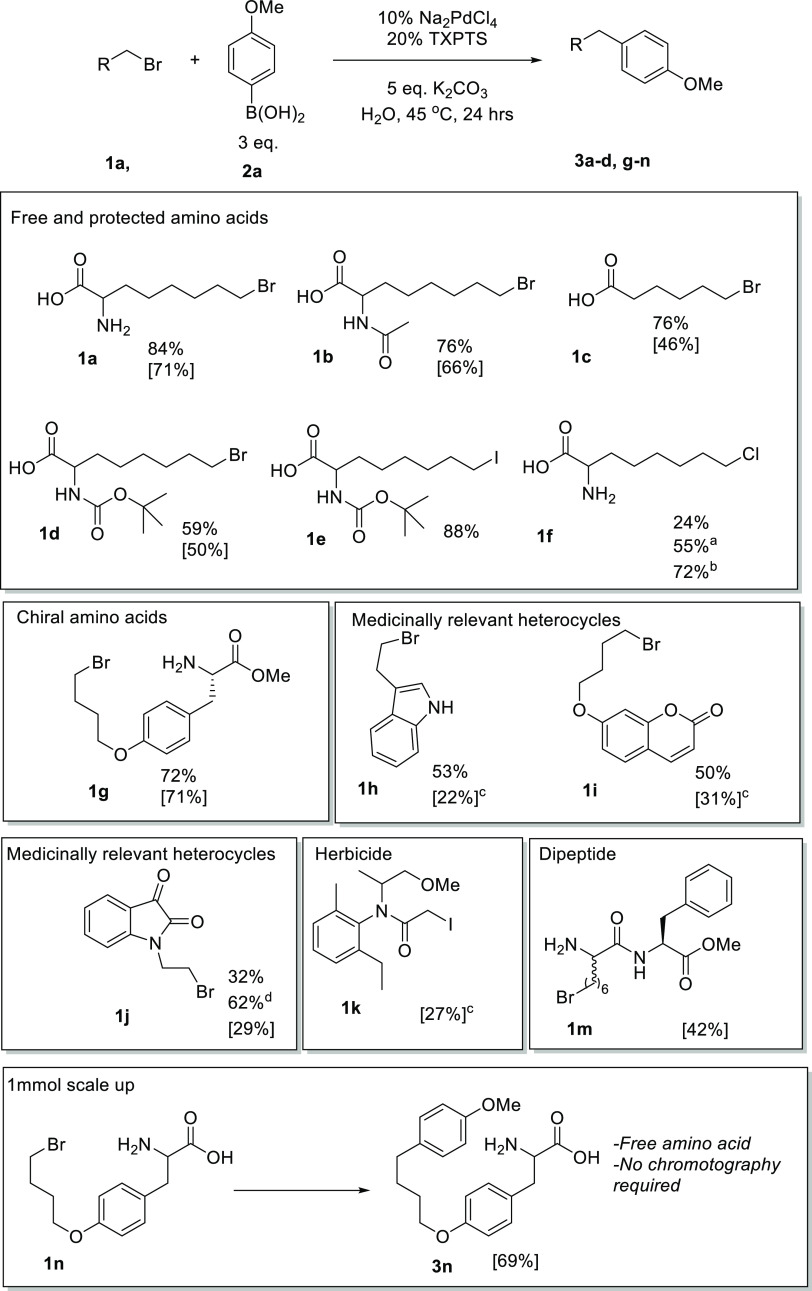
Scope of alkyl halide: total solvent volume, 0.6 mL; halide, 0.1 mmol; and boronic acid, 0.3 mmol. NMR yields reported are determined in comparison to the internal standard 1,4-dimethoxybenzene (0.0667 mmol, 6.9 mg) in the ^1^H NMR of the crude sample. ^a^20 mol% KI; ^b^72 h; ^c^10% MeCN cosolvent; ^d^Double catalyst loading. Isolated yields are given in square brackets.

Using the conditions developed on testbed compound **1a**, the substrate scope was explored. As amino acids are often protected for further derivatization, we were pleased to see that not only could free amino acids be coupled, but so could a wide range of protected or simplified species, including *N*-acetyl (**1b**), free acid (**1c**), and Boc (**1d**), in similarly good yields (Figure 3). Pleasingly, these conditions also enabled the coupling of capricious alkyl iodides (**1e**), and notably enabled the reaction with an alkyl chloride (**1f**). Often, much more forcing conditions are required for the coupling of alkyl chlorides, a much cheaper and more stable reagent than alkyl bromides. Simply extending the reaction time of this more unreactive chloride enabled high yields akin to that of bromides with minimal conversion to hydroxide.

Avoiding epimerization is often a challenge associated with cross-coupling, particularly when organic solvents necessitate the use of stronger bases. To determine whether our conditions would be sufficiently mild to avoid epimerization, an enantiopure unnatural (*S*)-tyrosine (**1g**) derivative was synthesized. The reaction of the enantiopure compound **1g** afforded good conversion while gratifyingly retaining the stereochemical integrity at the α-carbon in these basic aqueous conditions. Furthermore, medicinally relevant privileged heterocyclic moieties, often found in drug-like molecules, were successfully derivatized. Heterocycles, including brominated indole, (**1h**) coumarin (**1i**), and isatin (**1j**), were afforded in good yields under these conditions. The low yield of **1j**, potentially due to the low water solubility, could be almost doubled by doubling the catalyst loading. As an example of a more complex halogenated biomolecule, a dipeptide (**1m**) containing the unnatural brominated amino acid was synthesized and cross-coupled with *para*-methoxyphenyl boronic acid in good yields. The use of the Suzuki–Miyaura reactions to site-selectively derivatize amino acids and peptides in biorthogonal (or aqueous conditions) reactions has been established as an important reaction in the derivatization of peptides, synthesis of peptide-based drugs, or even protein tagging.^25^ Our coupling of **1m** represents the first such example of this being translated to alkyl halide amino acids and peptides.

Metolachlor is a racemic alkyl chloride used as a herbicide, particularly against grass.^46^ To show that these aqueous coupling chemistries could be used on diverse biologically active compounds, including sterically congested systems, the coupling of metolachlor with *para*-methoxyboronic acid was explored. After 24 h, no coupling or hydrolysis was observed, and the compound remained unmodified; the sterically hindered nature of all four stereoisomers of metolachlor, as well as the hydrophobic nature of the molecule, may impede the approach of boronic acid for transmetalation, making the coupling challenging. To overcome this, metolachlor was reacted with potassium iodide to yield the corresponding alkyl iodide (**1k**) and then coupled with boronic acid to generate a metolachlor analogue, providing a potential two-step method for the diversification of less reactive alkyl halide agents in water. As practicality and scalability are often important considerations in the development of novel methodologies, the unnatural free amino acid **1n** was synthesized and coupled at a 1 mmol scale with a very good, isolated yield of 69%. High levels of conversion meant that column chromatography was not required during purification, with the product being precipitated as a hydrochloride salt. By simply varying the boronic acid, this process could be used for the effective large-scale synthesis of amino acid derivatives.

**Figure 4:**
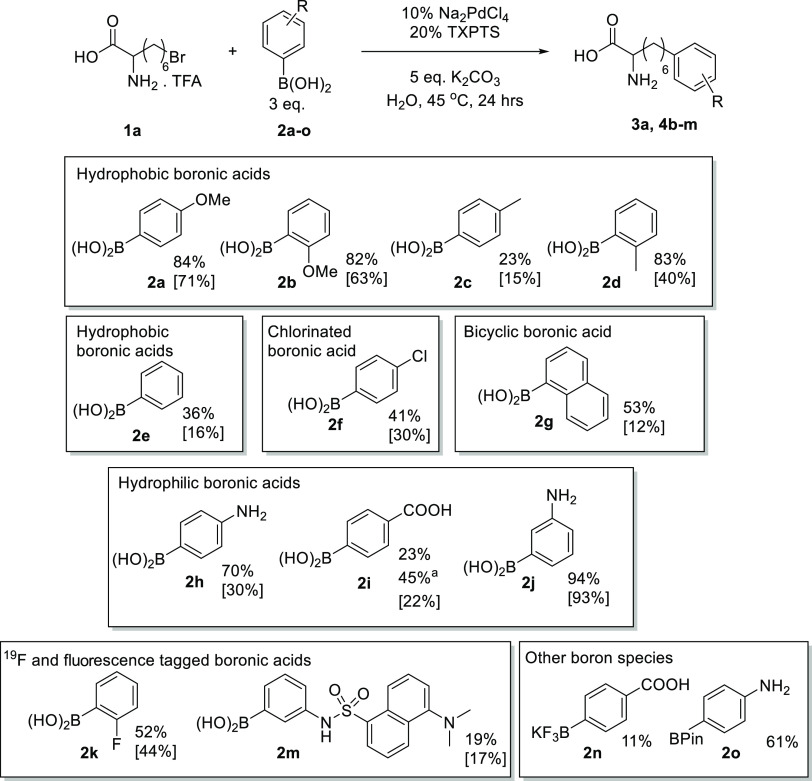
Boronic acid scope: total solvent volume, 0.6 mL; halide, 0.1 mmol; boronic acid, 0.3 mmol. NMR yields reported are determined in comparison to the internal standard 1,4-dimethoxybenzene (0.0667 mmol, 6.9 mg) in the ^1^H NMR of the crude sample. Isolated yields are given in square brackets. **4i** was purified by normal-phase column chromatography after a one-pot coupling and *N*-Boc protection (see Section S1.4). ^a^ Double catalyst loading.

Having demonstrated the ability to couple a range of challenging alkyl halides, we next set out to explore how different boronic acids would perform under these mild, aqueous conditions, again using brominated amino acid **1a** as the testbed (Figure 4). All coupling partners that we explored worked well, including small and medium-sized unfunctionalized aryl boronic acids, aryl systems with methyl, amino, carboxy, or fluoro substituents, and the sterically bulky dansylboronic acid (**2m**). Reactivity was generally determined by the electron richness of the carbon–boron bond rather than solubility, with *para-* and *ortho*-methoxyphenyl boronic acids (**2a** and **2b**) giving excellent yields and the electron-poor hydrophilic **2i** giving a much lower yield than the electron-rich hydrophilic **2h**. Curiously, *ortho*-tolyl (**2d**) gave much higher yields than *para*-tolyl boronic acid (**2c**). *Ortho*-substituted boronic acids are often resistant to palladium-based coupling, with the steric bulk inhibiting the transmetalation step onto palladium. This organometallic intermediate is less sterically hindered in reactions with terminal alkyl halides when compared to aryl halides, and thus, conversion is enabled. With the addition of 10% acetonitrile cosolvent, the yield of **2c** coupling could be increased to 46%, indicating that the low solubility of unfunctionalized boronic acids under aqueous conditions is likely to impact yields; however, this seems to be less impactful than the aryl group electronics. Boronic acids containing a fluorine (**2k**) and a dansyl (**2m**) group could be reacted with **1a**, indicating that this chemistry has high practical use in the tagging of halogenated biomolecules with either a ^19^F or a fluorescent probe. A BPin ester (**2o**) and borofluorate salt (**2n**) could also be reacted, meaning that this system is not entirely dependent on the boronic acid species. It is worth noting that the isolation of modified amino acids proved nontrivial on a small scale, leading to relatively low isolated yields. While attempts were made to derivatize these amino acids to their Boc carbamates for easier purification, this method did not improve isolated yields.

### 2.4 Natural Product Analogue Generation

**Figure 5:**
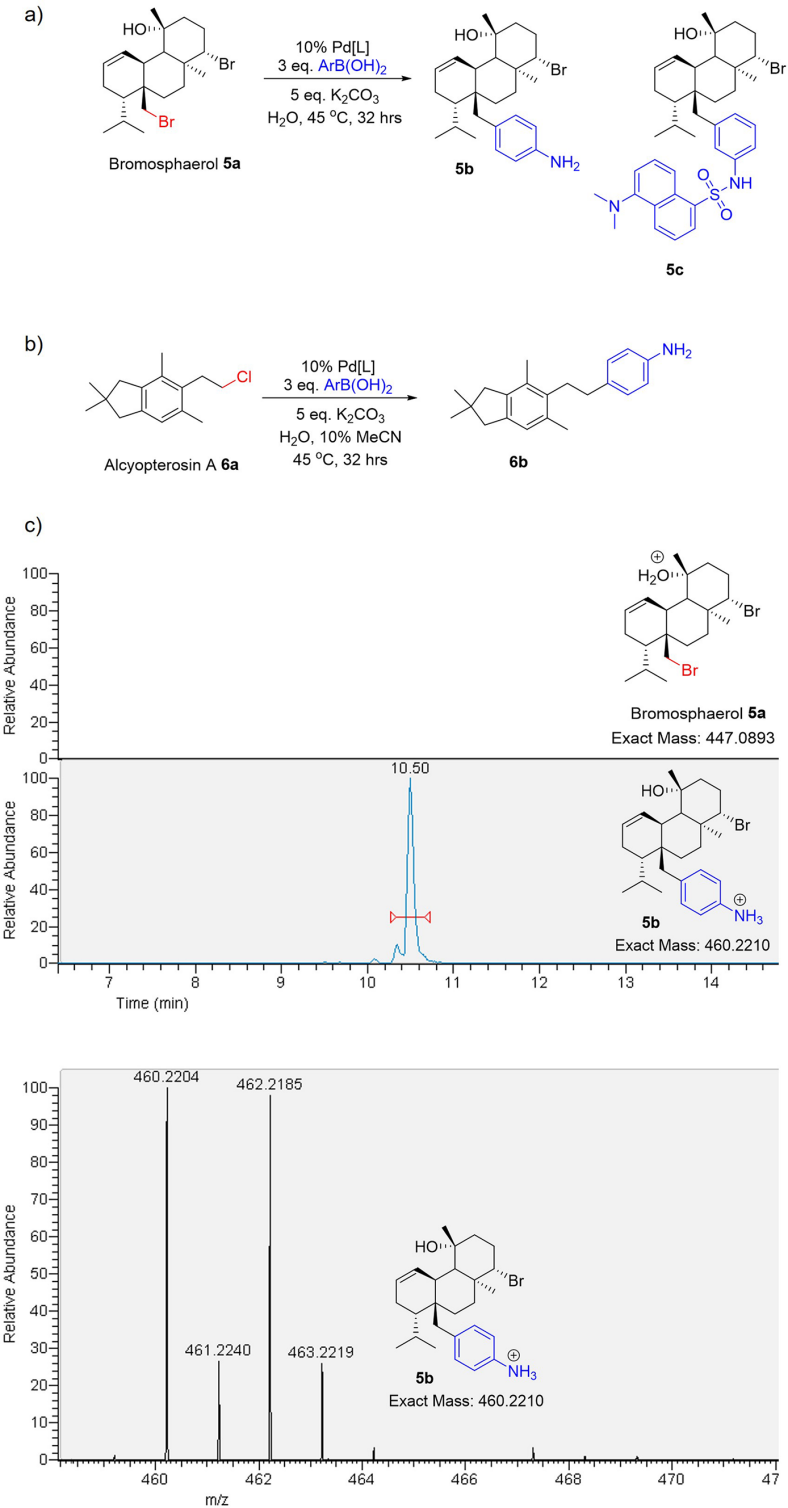
Natural product diversification: (a) bromosphaerol, **5a**, from *S. coronopifolius* and (b) alcyopterosin A, **6a**, from *A. roseum*, analogue generation in water. Products analyzed and confirmed by LC–MS. Total solvent volume, 30 μL; halide, 0.001 mmol; boronic acid, 0.005 mmol. (c) Example of cross-coupling for visualization on LC–MS; top: LC–MS trace of **5a** (*m*/*z* = 447.0880–447.092) and **5b** (*m*/*z* = 460.2200–460.2300); bottom: found *m*/*z* of **5b** against retention time (10.33–10.66 min).

We demonstrated the applicability of our aqueous cross-coupling chemistry to amino acids, a representative peptide, medicinally relevant molecules, and a potent agrochemical. Encouraged by the applicability of our mild conditions in derivatizing relatively complex halogenated molecules containing sensitive functionalities, we explored their application as a method of late-stage functionalization for halogenated natural products. Marine metabolites have evolved to perform a series of roles, especially in signaling inter and intra species.^47^ Produced in the oceans, where they could be readily washed away, certain metabolites are often highly lipophilic,^48,49^ potentially promoting their uptake and retention within various biological systems. Their interplay between organic and aqueous environments is critical, and the possibility to tag and track or immobilize these metabolites has the potential to open new horizons in the chemical ecology. The natural marine product, bromosphaerol (**5a**), produced by the red alga *Sphaerococcus coronopifolius*,^50^ is shown to have marine antifouling activity. Semisynthetic generation of analogues to explore their function and tune their activity has been explored successfully;^51^ however, this requires a very selective and mild chemistry on the native molecule, which is inherently prone to substitution, elimination, and rearrangement reactions. By using the primary alkyl bromide as an orthogonal handle, we have been able to selectively install aryl groups to generate analogues **5b**, a primary amine tag to enable LC–MS visualization, and **5c**, a fluorescent tag, in 100% water on an analytical scale (Figure 5a). Alcyopterosin A (**6a**) is found in the deep-sea soft coral *Alcyonium roseum*;^52^ subjecting this inherently less reactive organochloride to our coupling conditions enabled the detection of the derivative by LC–MS by virtue of the readily ionized primary amine tag (Figure 5b).

Not only do these methods allow for the mild and late-stage generation of natural product analogues in water, but also aid in the tagging of these molecules with functionalities that allow them to be ionized and detected by mass spectrometry. Both native alcyopterosin A and bromosphaerol were invisible on positive-mode LC–MS; however, by attaching ionizable tags, we were able to carry out mass spectrometry analysis (Figure 5c). Hence, subjecting the metabolome of a growing organism in culture to this cross-coupling with the appropriate boronic acid could potentially be used to identify novel primary halides containing natural products more easily.

## 3 Conclusions

The discovery and development of aqueous reaction conditions is an important but challenging pursuit that can not only open a gateway to more sustainable synthetic procedures and increase compatibility with sensitive functional groups but also provide new tools for chemical biology or even enable synbio–synchem compatibilities. In this study, we have shown the first general method for the fully aqueous Suzuki–Miyaura coupling of unactivated primary alkyl halides with boronic acids, allowing for the diversification of C(sp^3^)–X bonds in molecules containing highly sensitive functionalities. We designed our approach to be accessible using commercially available catalysts and ligands. We demonstrated that the reaction can be carried out on the bench with no need for air exclusion and is tolerant to a range of challenging functional groups as well as applied to two natural products. Potential practical uses of this reaction are evident, such as bioorthogonal peptide or amino acid diversification, natural product analogue generation, and enabling the orthogonal tagging of metabolites for analysis or even discovery. The mild conditions and low temperature open up the possibility of merging aqueous alkyl halide cross-coupling with bioenzymatic alkyl halide generation. This expands our groups’ “GenoChemetic toolbox” of aqueous coupling reactions, this being the first example on alkyl halide substrates, paving the way to potentially modulate and tune alkyl halides in the presence of living systems.^19^

## Supporting Information Available

Full methods, synthesis of compounds, and characterization (PDF)

## Abbreviations

NMR: nuclear magnetic resonance
TMG: tetramethylguanadine
TEMPO: (2,2,6,6-tetramethylpiperidin-1-yl)oxyl
LC–MS: liquid chromatography–mass spectrometry
