# Supplementary material for: Fully Aqueous and Air-Compatible Cross-Coupling of Primary Alkyl Halides with Aryl Boronic Species: A Possible and Facile Method
Source: ACS Catal. 2023 Apr 24;13(9):6365–74. doi: 10.1021/acscatal.3c00252 (PMC10167655; doi:10.1021/acscatal.3c00252)
Supplement: Supplementary file 1 — cs3c00252_si_001.pdf [file cs3c00252_si_001.pdf]

Supporting Information

Fully Aqueous and Air Compatible Cross-Coupling of Primary Alkyl Halides with Aryl Boronic Species: Possible and Facile.

Samuel Molyneux, and Rebecca J. M. Goss*

rjmg@st-andrews.ac.uk

School of Chemistry, University of St Andrews, North Haugh, St Andrews, Fife, KY16 9ST, UK.

Index

1. General experimental details 1

- 1.1. Procedure A- Catalyst screening for cross coupling of brominated amino acid 2
- 1.2. Procedure B- Aqueous alkyl bromide coupling 2
- 1.3. Preparation of Pd-TXPTS stock solution (Pd-catalyst stock) 2
- 1.4. Procedure C- One pot cross coupling of brominated amino acid and *N*-Boc protection 2
- 1.5. Procedure D- Analytical scale aqueous cross coupling natural product diversification 3
- 1.6. Base screening 3
- 1.7. TEMPO addition 4
- 1.8. Coupling of bromocyclohexane 4
- 1.9. General procedure for LC-MS 4
- 1.10. General procedure for UPLC 5
- 1.11. Procedure for stereochemical elucidation using FDAA 5

2. Coupling procedures and compounds 5

3. Synthetic procedures and compounds 27

4. References 43

5. NMR Spectra 44

1. General Experimental Details

Proton NMR (^1H), carbon NMR (^{13}C) and fluorine NMR (^{19}F) were recorded on a Bruker Ascend 500 (500 MHz), Bruker 500 UltraShield (500 MHz), Bruker 400 UltraShield (400 MHz). NMR spectra were processed and analysed using MestReNova. Chemical shifts are quoted in parts per million (ppm) and coupling constants are reported in Hertz (Hz). The NMR experiments were carried out in deuterated chloroform ($CDCl_3$) or deuterated MeOH (MeOD). The chemical shifts (δ) are quoted in parts per million (ppm). ^{13}C NMR signals were assigned to CH_3, CH_2, CH and C using a DEPTQ or an HSQC experiment with multiplicity editing. LC-MS analysis was performed using a ThermoScientific Dionex Ultimate 3000 UHPLC+ and a ThermoScientific Orbitrap Velos Pro mass spectrometer.

Analysis of the LC-MS results was carried out by ThermoScientific Xcalibur and ThermoScientific SIEVETM v2.2 x64 software.

Flash chromatography was performed using Davisil silica gel LC60A (40-63 micron). Thin layer chromatography (TLC) was executed using aluminium sheets of silica gel 60 F254 and was visualised under a Mineralight model UVGL-58 lamp (254 nm). The plates were developed with basic potassium permanganate solutions or ninhydrin in acetone. Flash column chromatography was carried out on a Biotage Isolera Four using normal phase SNAP-sil 10 g or reverse-phase SNAP C18 12 g column cartridge. Microwave reactions were conducted in sealed vials (MW vial) using a Biotage Initiator+ microwave reactor.

All solvents and regents were purchased from Alfa Aesar, Thermo Scientific, Fluorochem, or Sigma Aldrich and were used without further purification unless otherwise stated.

1.1. Procedure A- Catalyst screening for cross-coupling of brominated amino acid

2-Amino-8-bromohexanoic acid TFA salt (1 eq, 0.10 mmol), *p*-methoxyphenylboronic acid (3 eq, 0.30 mmol), palladium source (10 mol%, 0.01mmol) and ligand (20 mol%, 0.02 mmol) were added in air to a screw cap vial. The vial was sealed, evacuated and backfilled with argon three times, after which dioxane (0.3 ml), and aqueous NaOH (aq, 1M, 0.3 ml) were added over argon. The heterogenous mixture was heated with stirring at the desired temperature for the appropriate time. Reaction was monitored by ^{1}H NMR of aliquots (0.06 ml). The reaction mixture was then diluted with H_2O (2 ml) and washed with Et_2O (2 ml x 1). The aqueous layer was then acidified to pH 2 with HCl (1M),and extracted with EtOAc (2 ml x 4). The combined EtOAc extracts were dried over $MgSO_4$, filtered, and reduced in vacuo to give orange/brown solid. Bromo-amino acid consumption was determined by the ratio of a defined starting material peak to product peak.

1.2. Procedure B- Aqueous alkyl bromide coupling

Alkyl bromide (1 eq, 0.10 mmol), boronic acid (3 eq, 0.30 mmol), and potassium carbonate (5 eq, 0.5 mmol, 69.1 mg) were added to a MW vial in air. H_2O (0.5 ml) was added, and reaction mixture was stirred for 5 minutes. Pd-TXPTS catalyst stock solution (0.1 ml, corresponding to 10 mol% Pd) was added and reaction mixture was stirred at 45 ^{o}C for 24 hours. Reaction mixture was diluted with H_2O (2 ml), and washed with Et_2O (2 ml x 1). The aqueous layer was then acidified to pH 2 with HCl (1M) (unless otherwise stated), and H_2O evaporated *in vacuo*. Internal standard (dimethoxybenzene 6.9 mg) was added to determine NMR yield in MeOD. Crude mixture subjected to reverse-phase column chromatography on Biotage Isolera Four using reverse-phase SNAP C18 12 g column cartridge for purification. A generic binary gradient elution was carried out using different ratios of eluents A (H_2O

containing 0.1% TFA) and B (MeOH). Following gradient was used: 2 CV (0% B), 14 CV (0% to 100% B), 4 CV (100% B).

1.3. Preparation of Pd-TXPTS stock solution (Pd-catalyst stock)

A stock solution of Na_2PdCl_4 (10.2 mg, 0.035 mmol) and TXPTS (45.6 mg, 0.07 mmol) in H_2O (0.35 ml) was prepared and appropriate volume was used for cross coupling reactions. This solution, when prepared in degassed H_2O, was stored in a freezer at -20 ^oC and used within 3 days.

1.4. Procedure C- One pot cross coupling of brominated amino acid and *N*-Boc protection

Alkyl bromide (1 eq, 0.10 mmol), boronic acid (3 eq, 0.30 mmol), and potassium carbonate (69.1 mg, 5 eq, 0.5 mmol) added in air to a MW vial. H_2O (0.5 ml) added, and reaction mixture stirred for 5 minutes. Pd-catalyst stock solution (0.1 ml, corresponding to 10 mol% Pd) was added and reaction mixture stirred at 45 ^oC for 24 hours. Di-*tert*-butyl dicarbonate (2 eq, 0.2 mmol, 45.4 mg) in dioxane (0.6 ml) was added to reaction mixture and stirred overnight at 45 ^oC. Reaction mixture was acidified to pH 3 with citric acid (1M), and extracted with EtOAc (5 x 2 ml). Combined organic extract was washed with citric acid (1M, 5 ml) and H_2O (5 ml), dried over $MgSO_4$, concentrated under reduced pressure, and purified by normal phase flash column chromatography.

1.5. Procedure D- Analytical scale aqueous cross coupling- natural product diversification

Natural product (1 µmol) was transferred into 100 µl eppendorf. A solution of potassium carbonate and aryl boronic acid in H_2O (20 µl, 2.5 mM base, 1.5 mM boronic acid) was added to vial, followed by H_2O (9 µl) and Pd-catalyst stock solution (1 µl corresponding to 10mol% Pd). Reaction mixture was stirred at 45 ^oC for 48 hours. MeOH (100 µl) added to the reaction mixture, followed by DTT (2 µl, 1M) to quench, for 10 minutes at rt. HCl (100 µl, 1M) was added to the reaction mixture, which was then centrifuged (17,000 x g, rt, 10 mins) The crude supernatant was injected into the LC-MS for analysis.

1.6. Base screening

Procedure A was followed using 2-Amino-8-bromohexanoic acid TFA salt (1 eq, 0.10 mmol), *p*-methoxyphenylboronic acid (3 eq, 0.30 mmol), Pd-TXPTS catalyst stock solution (0.1 ml, corresponding to 10 mol% Pd), and base (5 eq, 0.5 mmol).

Supplementary Table 1: Base screen for aqueous alkyl halide coupling

Entry	*Variation to the conditions*	*SM**	*Alcohol*	*Product*†
1	NaOH	0%	39%	61%
2	Na_2CO_3	6%	35%	59%

3	$NaHCO_3$	86%	7%	7%
4	K_2CO_3	3%	29%	68%
5	$KHCO_3$	80%	11%	9%
6	Cs_2CO_3	10%	32%	58%
7	CsF	100%	0%	0%
8	TEA	36%	41%	23%
9	K_3PO_4	50%	28%	22%
10	K_2HPO_4	92%	8%	0%
11	KH_2PO_4	91%	9%	0%
12	K_2CO_3, 45 °C, 24 hrs	0%	16%	84%
13	K_2CO_3, 45 °C, 24 hrs, 10% Na_2PdCl_4, 0% TXPTS	10%	90%	0%
14	K_2CO_3, 45 °C, 24 hrs, 0% Na_2PdCl_4, 0% TXPTS	6%	94%	0%
15	K_2CO_3, 45 °C, 24 hrs, 5% Na_2PdCl_4, 10% TXPTS	5%	45%	50%
16	K_2CO_3, r.t., 24 hrs	81%	14%	5%

*High conversions to yields ratio are a result of formation of hydroxylated amino acid **1c** competing with desired coupling. A synthetic standard of the of hydroxylated amino acid was generated through direct reaction with LiOH to confirm (see SI). †Yields reported are determined by comparison to internal standard 1,4-dimethoxybenzene (0.0667 mmol, 6.9 mg) in crude sample ^1H NMR.

1.7. TEMPO addition

General Procedure B was followed using 2-Amino-8-bromohexanoic acid TFA salt (35.2 mg, 0.10 mmol), *p*-methoxyphenylboronic acid (45.6 mg, 0.30 mmol), and TEMPO (78.1 mg, 0.50 mmol). Once reaction was complete an aliquot (50 µl) of reaction mixture was removed and subjected to LC-MS analysis.

1.8. Coupling of bromocyclohexane

General Procedure B was followed using bromocyclohexane (16.3 mg, 0.10 mmo) (35.2 mg, 0.10 mmol) and *p*-methoxyphenylboronic acid (45.6 mg, 0.30 mmol).

1.9. General procedure for LC-MS

Liquid chromatography-high resolution mass spectrometry (LC-HRMS) analysis of the cross-coupling reactions was conducted on a Thermo Scientific Dionex Ultimate 3000 Rapid Separation LC system using XBridge BEH C18 column (130 Å, 3.5 µm, 2.1 × 100 mm). The flow rate was set to 0.35 ml min−1 and the column temperature was maintained at 40 °C. A generic binary gradient elution was carried out using different ratios of eluents A (H_2O containing 0.1% formic acid) and B (MeCN). Following gradient was used: 0–0.5 min (5% B), 0.5–9.5 min (5% to 95% B), 9.5–11.5 min (95% B), 11.5–12.0 min (95% to 5% B) and 12.0–15.0 min (5% B). The mass spectrometric analysis was performed on an Orbitrap Velos Pro™ mass spectrometer system equipped with a Thermo Scientific Ion MAX

API source housing. The MS conditions were as follows: heated electrospray ionization (HESI-II) probe, positive ionization mode, spray voltage 3.5 kV, capillary temperature 350 °C, normalized collision energy 35% for collision induced dissociation, and sheath gas and auxiliary gas flow rates of 35 and 10 arbitrary units, respectively. Full scan MS spectra (from m/z 50–1000) were acquired in the orbitrap with resolution R = 60 K.

1.10 General procedure for UPLC

UPLC analysis was performed on Acquity UPLC BEH C18 (1.7 µm, 2.1 × 50 mm) column eluting with 0.1% TFA in H_2O (solvent A) and MeCN (solvent B). Following gradient was used: 0–0.5 min (5% B), 0.5–5.5 min (5% to 95% B), 5.5–6.0 min (95% B), 6.0–6.3 (95–5% B), 6.3–6.6 min (5% B). The flow rate was set to 350 µl min^{-1} and the column temperature was maintained at 40 °C. Detection was achieved by UV detection (PDA 200–400 nm).

1.11 Procedure for stereochemical elucidation using FDAA (Marfey's reagent)

Substrate to elucidate stereochemical purity (1 mg) was dissolved in H_2O (150 µl), followed by the addition of N-α-(2,4-dinitro-5-fluorophenyl)-L-alaninamide (FDAA)[1] (1% w/v in acetone, 300 µl) and $NaHCO_3$ (1M, 140 µl). Mixture was incubated at 40 °C for 1 hour, followed by quenching with HCl (1M, 140 µl), and diluted with MeOH (1 ml). Sample was then centrifuged (17,000 x g, rt, 10 mins), and clear supernatant was used for UPLC or LCMS analysis.

2. Coupling procedures and compounds

2-Amino-8-(4-methoxyphenyl)octanoic acid-3a

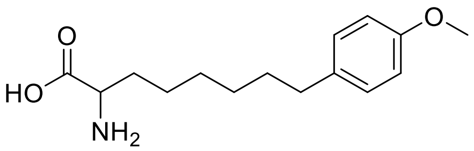

General Procedure B was followed using 2-Amino-8-bromohexanoic acid TFA salt (35.2 mg, 0.10 mmol) and *p*-methoxyphenylboronic acid (45.6 mg, 0.30 mmol). Crude mixture subjected to reverse-phase column chromatography on Biotage Isolera Four, using H_2O (1% v/v TFA): MeOH (gradient 0 – 100%) to give a pale yellow solid (18.8 mg, 55%)

^{1}H NMR (500 MHz, MeOD) δ 7.07 (d, J = 8.5 Hz, 2H, Ar-H), 6.81 (d, J = 8.5 Hz, 2H, Ar-H), 3.93 (t, J = 6.2 Hz, 1H, CH), 2.55 (t, J = 7.6 Hz, 2H, CH_2Ar), 1.98 – 1.81 (m, 2H, CH_2), 1.76 - 1.60 (m, 2H, CH_2), 1.50-1.33 (m, 6H, $CH_2CH_2CH_2$). ^{13}C NMR (126 MHz, MeOD) δ 171.9 (COOH), 159.3 (C) 135.8 (C), 130.2 (Ar-H), 114.7 (Ar-H), 55.6 (CH), 53.9 (CH_3), 35.9 (CH_2), 32.7 (CH_2), 31.5 (CH_2), 30.1 (CH_2), 29.8 (CH_2), 25.8 (CH_2).

LCMS-ESI-MS. Positive Mode. Expected mass of $[C_{15}H_{24}O_3N]^+$ 266.1751, m/z of sample 266.1746.

FTIR (neat) cm^{-1}: 3007, (OH, br), 2924 (CH, m), 2853 (NH, br) 1732 (CO, s), 1512 (CH, m), 1464 (CH, m), 1414 (CH, m), 1242 (CO, s)

N-Acetyl-2-amino-8-(p-methoxyphenyl)-octanoic acid- 3b

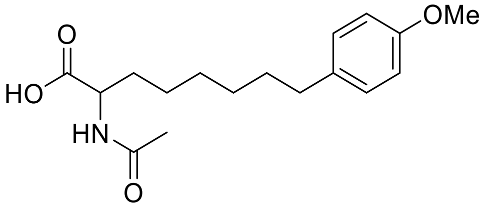

General Procedure B was followed using *N*-acetyl bromo amino acid (38.2 mg, 0.10 mmol) and *p*-methoxyphenyl boronic acid (45.6 mg, 0.30 mmol). The crude mixture was purified by flash silica chromatography using DCM: MeOH (gradient 0 – 10%) (10% MeOH in DCM, Rf = 0.15) to give white solid product (20.3 mg, 66%)

^1H NMR (500 MHz, $CDCl_3$) δ 7.07 (d, J = 8.6 Hz, 2H, Ar-H), 6.82 (d, J = 8.6 Hz, 2H. Ar-H), 6.14 (d, J = 7.7 Hz, 1H, NH), 4.57 (td, J = 7.5, 5.3 Hz, 1H, CH), 3.78 (s, 3H, OMe), 2.53 (t, J = 7.8 Hz. 2H. CH_2), 2.04 (s, 3H, $COCH_3$), 1.91–1-.82 (m, 1H, CHH), 1.68 (m, 1H, CHH), 1.59 – 1.53 (m, 2H, CH_2), 1.37 – 1.30 (m, 6H, $CH_2CH_2CH_2$). ^{13}C NMR (126 MHz, $CDCl_3$) δ 175.8 (COOH), 171.1 (N-COMe), 157.7 (C), 134.9 (C), 129.4 (CH), 113.8 (CH), 55.4 (OCH_3), 52.5 (CH), 35.1 (CH_2), 32.1 (CH_2), 31.7 (CH_2), 29.2 (CH_2), 29.1 (CH_2), 25.3 (CH_2), 23.2 (CH_3)

LCMS-ESI-MS. Positive Mode. Expected mass of $[C_{17}H_{26}O_4N]^+$ 308.1865, m/z of sample 308.1849.

7-(4-methoxyphenyl)heptanoic acid-3c

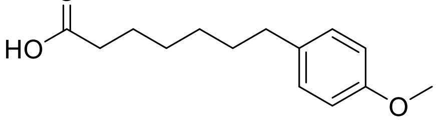

General Procedure B was followed using 7-bromoheptanoic acid (21.0 mg, 0.10 mmol) and *p*-methoxyphenyl boronic acid (45.6 mg, 0.30 mmol). The crude mixture diluted with H_2O (3 ml) and was washed with Et_2O (3 x 10 ml). Aqueous layer was adjusted to pH 2 with HCl (1M), concentrated under reduced pressure, and extracted with DCM (5 x 5ml) to give pale brown solid (11.0 mg, 46%)

^1H NMR (500 MHz, $CDCl_3$) δ 7.08 (d, J = 8.6 Hz, 2H, Ar-H), 6.82 (d, J = 8.6 Hz, 2H, Ar-H), 3.79 (s, 3H, OCH_3), 2.54 (t, J = 7.6 Hz, 2H, CH_2Ar), 2.34 (t, J = 7.5 Hz, 3H, C<u>H</u>$_2$COOH), 1.65 – 1.56 (m, 4H, CH_2CH_2), 1.41 – 1.30 (m, 4H, CH_2CH_2). ^{13}C NMR (126 MHz, CDC_{l3}) δ 177.9 (COOH), 157.6 (C), 134.8 (C), 129.3 (CH), 113.7 (CH), 55.3 (CH), 34.9 (CH_2), 33.6 (CH_2), 31.5 (CH_2), 28.9 (CH_2), 28.8 (CH_2), 24.6 (CH_2). These data are consistent with reported values[2]

2-((*Tert*-butoxycarbonyl)amino)-8-(4-methoxyphenyl)octanoic acid- 3d

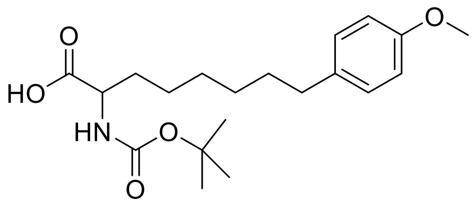

General Procedure C was followed using 2-Amino-8-bromohexanoic acid TFA salt (35.2 mg, 0.10 mmol) and *p*-methoxyohenylboronic acid (45.6 mg, 0.30 mmol). Crude mixture purified by flash column chromatography using DCM, 1% Tol: MeOH, 1% Tol) (gradient 0 – 10%) to give a colourless solid (22.9 mg, 71%, 10% MeOH in DCM, Rf = 0.27)

General Procedure B was followed using 8-bromo-2-((tert-butoxycarbonyl)amino)octanoic acid (33.8 mg, 0.10 mmol) and *p*-methoxyboronic acid (45.6 mg, 0.30 mmol). Crude mixture purified by flash column chromatography using DCM (1% v/v Toluene): MeOH (1% v/v Toluene) (gradient 0 – 10%) (10% MeOH in DCM: Rf = 0.27) to give a colourless solid (18.3 mg, 50%)

^1H NMR (500 MHz, $CDCl_3$) δ 7.08 (d, J = 8.6 Hz, 2H, Ar-H), 6.82 (d, J = 8.6 Hz, 2H, Ar-H), 4.95 (d, J = 8.3 Hz, 1H, NH), 4.31 – 4.27 (m, 1H, CH), 3.78 (s, 3H, OCH_3), 2.53 (t, J = 9.0, 7.9 Hz, 2H, CH_3Ar), 1.89 – 1.78 (m, 1H, C<u>H</u>H), 1.69 – 1.62 (m, 1H, CH<u>H</u>), 1.57 (p, J = 7.4 Hz, 2H, CH_2), 1.44 (s, 9H, $C(CH_3)_3$), 1.38 – 1.29 (m, 6H, $CH_2CH_2CH_2$). ^{13}C NMR (126 MHz, $CDCl_3$) δ 177.7 (COOH), 157.7 (COO^{t}Bu), 155.8 (C), 134.9 (C), 129.4 (CH), 113.8 (CH), 80.3 ($OCMe_3$), 55.4 (CH_3), 53.5 (CH), 35.1 (CH_2), 32.5 (CH_2), 31.7 (CH_2), 29.2 (CH_2), 29.1 (CH_2), 28.4 $(CH_3)_3$, 25.4 (CH_2)

LCMS-ESI-MS. Positive Mode. Expected mass of sodium adduct $[C_{20}H_{31}NO_5Na]^+$ 388.2100, m/z of sample 388.2092

(S)-2-amino-3-(4-(4-(4-methoxyphenyl)butoxy)phenyl)propanoic acid- 3g

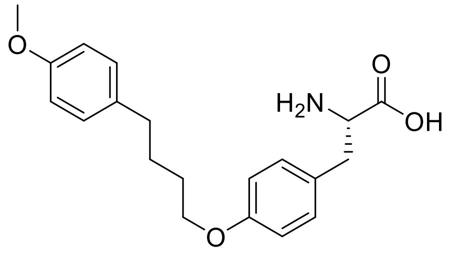

General Procedure B was followed using **1g** (44.1 mg, 0.01 mmol) and *p*-methoxybenzene boronic acid (45.6 mg, 0.30 mol). After 24 hrs lithium hydroxide (1M, 0.5 ml) was added to the reaction mixture and left to stir overnight. The pH of the reaction mixture was adjusted to 4 using HCl (1M), and H_2O was removed *in vacuo*. Crude mixture subjected to reverse-phase column chromatography on Biotage Isolera Four, using H_2O (1% v/v TFA): MeOH (gradient 0 – 100%) to give pure product as a yellow solid (24.3 mg, 71%)

^{1}H NMR (500 MHz, MeOD) δ 7.18 (d, J = 8.6 Hz, 2H, Ar-H), 7.10 (d, J = 8.6 Hz, 2H, Ar-H), 6.89 (d, J = 8.6 Hz, 1H, Ar-H), 6.82 (d, J = 8.7 Hz, 2H. Ar-H), 4.18 (dd, J = 7.7, 5.3 Hz, 1H, CH), 3.96 (t, J = 5.9 Hz, 2H, CH_2), 3.75 (s, 3H, OCH_3), 3.27 – 3.04 (m, 2H, CH_2), 2.61 (t, J = 6.9 Hz, 2H, CH_2), 1.83 – 1.68 (m, 4H, CH_2CH_2). ^{13}C NMR (126 MHz, MeOD) δ 171.3 (COOH), 160.3 (C), 159.3 (C), 135.5 (C), 131.5 (CH), 130.3 (CH), 127.1 (C), 116.1 (CH), 114.7 (CH), 68.9 (CH_2), 55.6 (CH_3), 55.3 (CH), 36.5 (CH_2), 35.6 (CH_2), 29.9 (CH_2), 29.3 (CH_2)

LCMS-ESI-MS. Positive Mode. Expected mass of $[C_{20}H_{26}NO_4]^+$ 344.1856, m/z of sample 344.1842.

FTIR (neat) cm^{-1}: 3004 (OH, br), 2928 (CH, m), 2854 (NH, m) 1609 (CO, s), 1509 (CH, m), 1471 (CH, m), 1420 (CH, m), 1246 (CO, s)

$[\alpha]^{20}_D$ + 5.2 (c = 0.71 MeOH)

Stereochemical elucidation performed using FDAA (see page 3), with substrates L-tyrosine and **3g**. Single peak from product of reaction between **3g** and FDAA indicates the formation of a single diastereomer.

Racemic **3g**, prepared by the coupling of racemic **1n** (see SI page 37), was subject to stereochemical elucidation using FDAA, and compared with enantiopure **3g** (Supplementary Figure 2). This led to the appearance of two peaks with the expected mass of **3g.FDAA** in the LC-MS trace indicates the formation of two diastereomers.

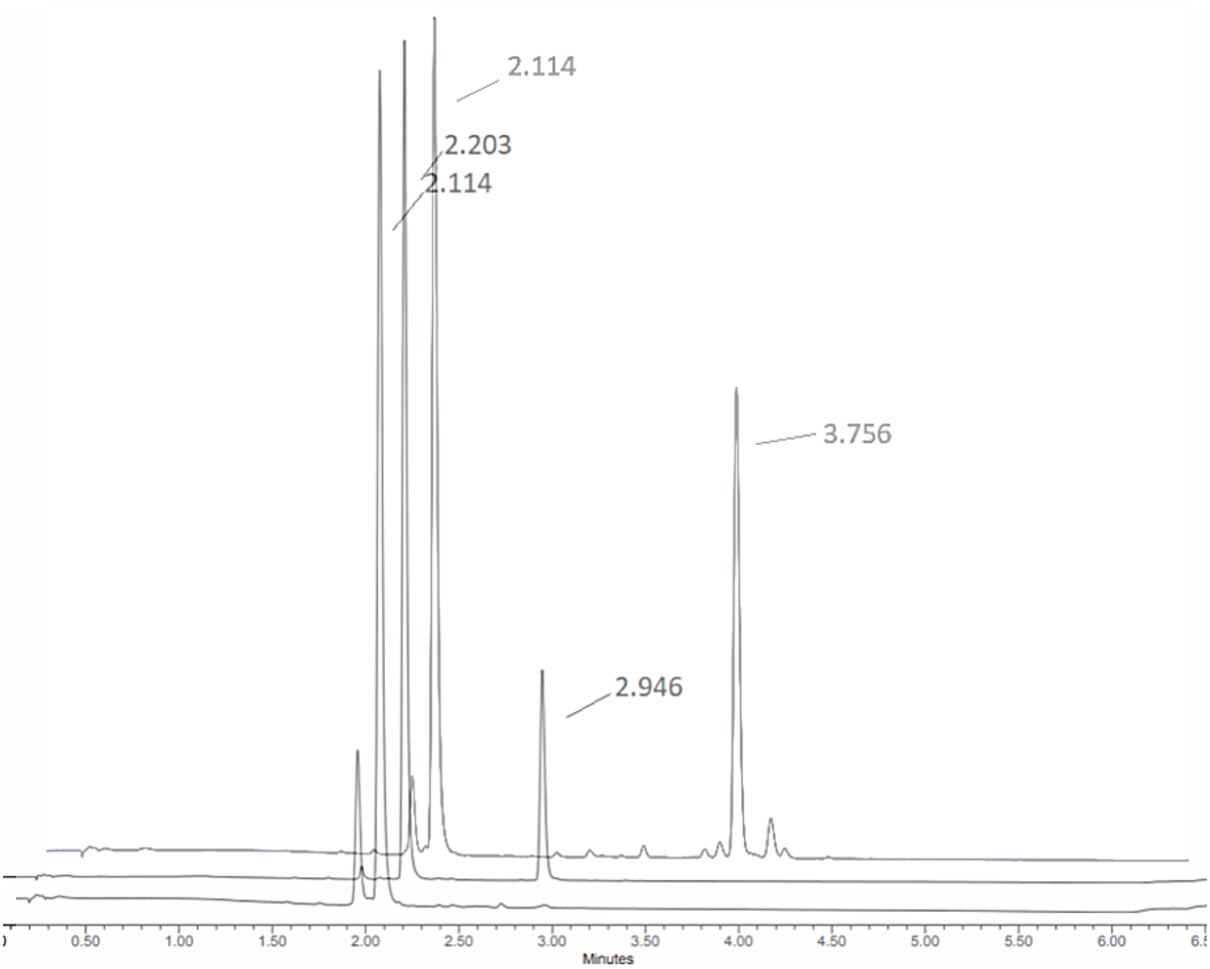

Supplementary Figure 1: Stereochemical elucidation of **3g**. Bottom line: control reaction containing no substrate. FDAA (subjected to reaction conditions) retention time 2.1 min. Middle line: L-Tyrosine.FDAA, retention time 2.9 min. Top line: **3g.FDAA**, retention time 3.8 min.

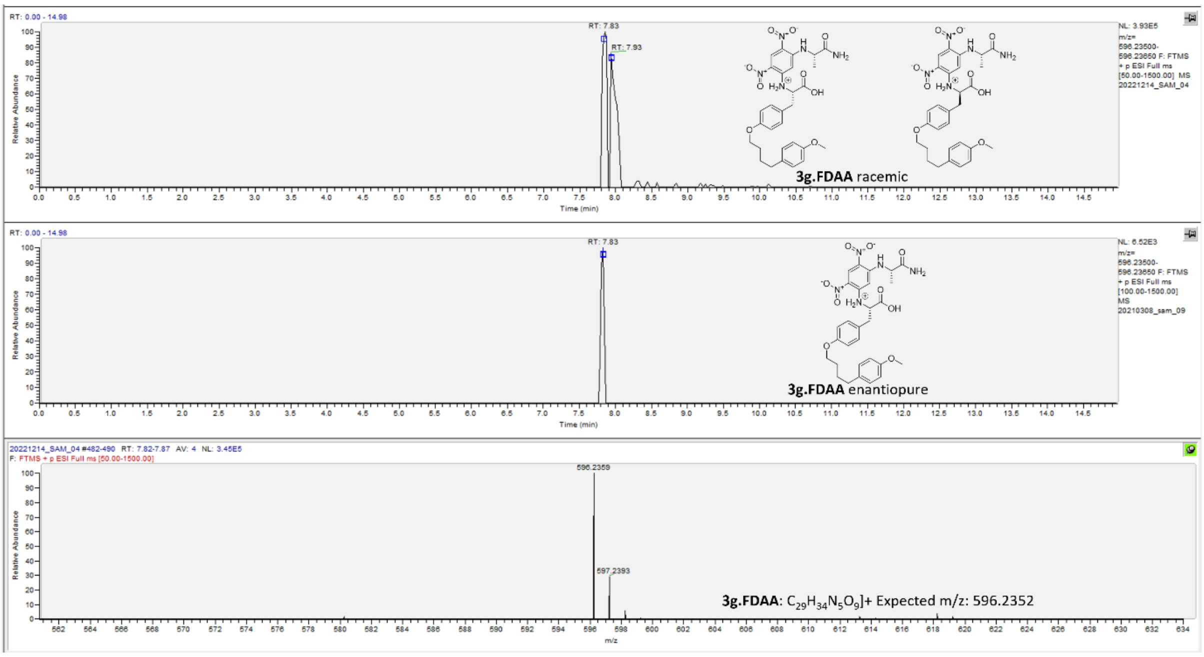

Supplementary Figure 2 Top: LC-MS trace of racemic **3g.FDAA**. Expected mass of **3g.FDAA** $[C_{29}H_{34}N_5O_9]^+$ 596.2352, m/z of peak retention time 7.83 min, 596.2359. m/z of peak retention time 7.93 min, 596.2358 Middle LC-MS trace of enantiopure **3g.FDAA**. Expected mass of **3g.FDAA** $[C_{29}H_{34}N_5O_9]^+$ 596.2352, m/z of peak retention time 7.83 min, 596.2354. Botttom: m/z of **3g.FDAA**

1 mmol scale cross-coupling of 2-amino-3-(4-(4-bromobutoxy)phenyl)propanoic acid hydrochloride (1n)

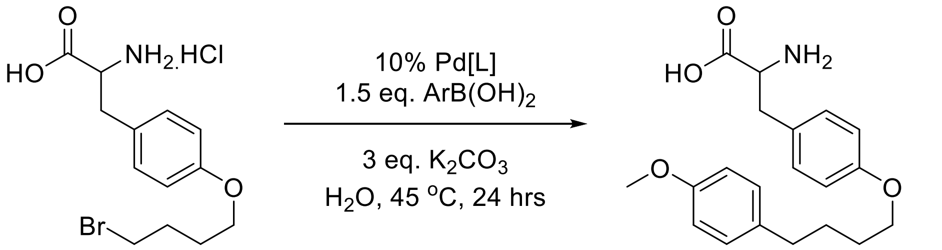

1n (1 mmol, 353 mg) and *p*-methoxyphenyl boronic acid (1.5 mmol, 227.9 mg) were suspended in H_2O (5 ml), and K_2CO_3 (3 mmol, 414.6 mg) added slowly. Once all base was added a solution of Na_2PdCl_4 (0.10 mmol, 29.4 mg) and TXPTS (0.2 mmol, 130 mg) in H_2O (1 ml) was added to the reaction mixture. The reaction was stirred at 45 °C for 24 hrs, then was allowed to cool to room temperature. The reaction mixture was then diluted with H_2O (50 ml) and washed with Et_2O (2 x 50 ml). The aqueous layer was then acidified to pH 4 with HCl (1M) to give a brown precipitate. The precipitate was filtered out and washed with H_2O (4 x 25 ml). The solid was then dried under high vacuum to give pure product (261 mg, 69%).

1n synthesised as a racemic mixture (see Synthetic Procedures **1n**).

^{1}H NMR (500 MHz, MeOD) δ 7.19 (d, J = 8.6 Hz, 2H, Ar-H), 7.10 (d, J = 8.6 Hz, 2H, Ar-H), 6.90 (d, J = 8.7 Hz, 2H. Ar-H), 6.82 (d, J = 8.6 Hz, 2H, Ar-H), 4.18 (t, J = 6.5 Hz, 1H, CH), 3.97 (t, J = 5.8 Hz, 2H, CH_2), 3.76 (s, 3H, OCH_3), 3.29 – 3.06 (m, 2H, CH_2, 2.61 (t, J = 7.0 Hz, 2H, CH_2), 1.76 (m, 4H, CH_2CH_2). These data are consistent with previous characterisation of **3g**.

3-(4-methoxyphenethyl)-1H-indole- 3h

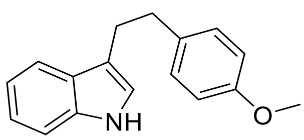

General Procedure B was followed using 3-(2-bromoethyl)indole (22.4 mg, 0.100 mmol) and *p*-methoxyphenyl boronic acid (45.6 mg, 0.30 mmol). 0.06 ml MeCN was added as cosolvent. Product was extracted with EtOAc (3 x 1 ml) and purified by flash silica chromatography using Hexane: EtOAc (gradient 0 – 100%) (10% EtOAc in Hexane, Rf = 0.47) to give white solid product (5.6 mg, 22%).

^{1}H NMR (500 MHz, $CDCl_3$) δ 7.66 – 7.60 (m, 1H), 7.37 (dt, J = 8.1, 0.9 Hz, 1H), 7.23 – 7.11 (m, 4H), 6.92 (dd, J = 2.3, 1.2 Hz, 1H), 6.89 – 6.81 (m, 2H), 3.80 (s, 3H, OCH_3), 3.11 – 2.90 (m, 4H, CH_2CH_2). ^{13}C NMR (126 MHz, $CDCl_3$) δ 157.9 (C), 136.4 (C), 134.7 (C), 129.5 (CH), 127.6 (C), 122.1 (CH), 121.4 (CH), 119.3 (CH), 119.0 (CH), 116.4 (C), 113.8 (CH), 111.2 (CH), 55.41 (CH) 35.7 (CH_2), 27.6 (CH_2).
LCMS-ESI-MS. Positive Mode. Expected mass of $[C_{17}H_{18}NO]^+$ 252.1383, m/z of sample 252.1380

7-(4-(4-methoxyphenyl)butoxy)-2H-chromen-2-one- 3i

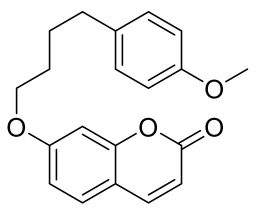

General Procedure B was followed using synthesised 7-(4-bromobutoxy)-2H-chromen-2-one **1i** (29.7 mg, 0.10 mmol) and *p*-methoxybenzene boronic acid (45.6 mg, 0.03 mol). 0.06 ml MeCN added as cosolvent. Product was extracted with EtOAc (3 x 1 ml) and purified by sequential flash column chromatography, using DCM (10% v/v Toluene): MeOH (gradient 0 – 5%), isocratic DCM (10% v/v Toluene) (10% Toluene in DCM, Rf = 0.20), to give pure product as a colourless solid (10.1 mg, 31%).

^{1}H NMR (500 MHz, $CDCl_3$) δ 7.63 (d, J = 9.5 Hz, 1H, Ar-H), 7.35 (d, J = 8.6 Hz, 1H, Ar-H), 7.12 (d, J = 8.6 Hz, 2H, Ar-H), 6.84 (d, J = 8.5 Hz, 2H), 6.82 – 6.78 (m, 2H), 6.24 (d, J = 9.4 Hz, 1H, Ar-H), 4.01 (t, J = 6.2 Hz, 2H, CH_2O), 3.79 (s, 3H, OCH_3), 2.64 (t, J = 7.4 Hz, 2H, CH_2Ar), 1.89 – 1.72 (m, 4H, CH_2CH_2). ^{13}C

NMR (126 MHz, $CDCl_3$) δ 162.5 (C), 161.5 (C), 157.9 (C), 156.0 (C), 143.6 (Ar-H), 134.2 (C), 129.4 (Ar-H), 128.8 (Ar-H), 113.9 (Ar-H), 113.1 (Ar-H), 113.1 (Ar-H), 112.5 (C), 101.4 (Ar-H), 68.6 (CH_2), 55.4 (CH_3), 34.7 (CH_2), 28.6 (CH_2), 28.1 (CH_2).

LCMS-ESI-MS. Positive Mode. Expected mass of $[C_{20}H_{21}O_4]^+$ 325.1434, m/z of sample 325.1429.

1-(4-methoxyphenethyl)indoline-2,3-dione- 3j

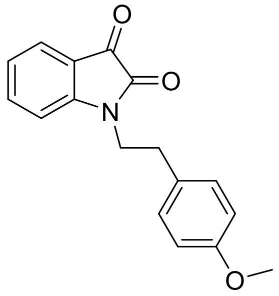

General procedure B was followed using synthesised 1-(2-bromoethyl)indoline-2,3-dione (**1j**) (0.10 mmol, 24.9 mg) and *p*-methoxyphenyl boronic acid (0.30 mmol, 45.6 mg). The reaction mixture was adjusted to pH 4 with HCl (1M). Product extracted with EtOAc (5 x 5 ml), washed with brine (3 x 5 ml), dried over $MgSO_4$, and purified by flash column chromatography using DCM: MeOH (gradient 0 – 2%) (1% MeOH in DCM, Rf = 0.2) to give product as an orange solid (8.2 mg, 29%)

^{1}H NMR (500 MHz, $CDCl_3$) δ 7.59 (ddd, J = 7.5, 1.4, 0.8 Hz, 1H, Ar-H), 7.53 (td, J = 7.8, 1.4 Hz, 1H, Ar-H), 7.14 (d, J = 8.6 Hz, 2H, Ar-H), 7.09 (td, J = 7.5, 0.8 Hz, 1H, Ar-H), 6.84 – 6.79 (m, 2H, Ar-H), 6.76 (d, J = 7.9 Hz, 1H, Ar-H), 3.92 (t, J = 7.5 Hz, 2H, NCH_2), 3.76 (s, 3H, OCH_3), 2.94 (t, J = 8.2, 7.4 Hz, 2H, CH_2Ar). ^{13}C NMR (126 MHz, $CDCl_3$) δ 198.0 (CO), 194.1 (CO), 158.2 (C), 151.1 (C), 138.44 (Ar-H), 129.9 (Ar-H), 129.6 (C), 125.6 (Ar-H), 123.7 (Ar-H), 117.6 (C), 114.3 (Ar-H), 110.2 (Ar-H), 55.4 (OCH_3), 42.1 (CH_2), 32.9 (CH_2).

LCMS-ESI-MS. Positive Mode. Expected mass of $[C_{17}H_{16}NO_3]^+$ 282.1125, m/z of sample 282.1122.
FTIR (neat) cm^{-1}: 2912 (s) 2849 (CH, br) 1732 (CO, s), 1612 (CO, s), 1470 (CH, m), 1414 (CH, m), 1248 (CO, s)

Coupled Metalachlor (N-(2-ethyl-6-methylphenyl)-2-(4-methoxyphenyl)-N-(1-methoxypropan-2-yl)acetamide)- 3k

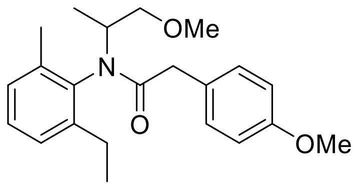

General procedure B was followed using synthesised N-(2-ethyl-6-methylphenyl)-2-iodo-N-(1-methoxypropan-2-yl)acetamide (**1k**) (0.10 mmol,) and *p*-methoxyphenyl boronic acid (0.30 mmol, 45.6 mg). Product was extracted with EtOAc (3 x 5ml), washed with H_2O (1 x 5ml) and brine (1 x 5ml), dried over $MgSO_4$, and solvent removed *in vacuo*. Crude product purified by flash column chromatography using DCM: MeOH (gradient 0 – 5%) (5% MeOH in DCM, Rf = 0.17) to give product as a yellow oil (9.6 mg, 27%)

Native metalachlor exists as four stable stereoisomers in solution at ambient conditions. aSS, aRS, aSR, and aRR.[3] Enantiomers due to chiral centre, and atropisomers due to limited rotation around the Ar-N bond- leading to separation of some peaks in NMR experiments of the aSS/aRR enantiomers (major, approximately 65%) and the aSR/aRS enantiomers (minor, approximately 35%).[4] Based on similar separation in **1k** peaks we can assume similar proportions of stereoisomers in solution. Where peaks are overlapping integrations are as expected, when peaks are separate their combined integrations are as expected.

^{1}H NMR (500 MHz, $CDCl_3$) δ 7.29-7.23 (m, 1H Ar-H, overlapping isomers), 7.23 – 7.14 (m, 1H, Ar-H overlapping isomers), 7.13 – 7.04 (m, 1H, overlapping isomers), 6.95 – 6.84 (m, 2H, Ar-H overlapping isomers), 6.79 – 6.73 (m, 2H, Ar-H overlapping isomers), 4.19 – 4.09 (m, 1H, CH overlapping isomers), 3.76 (s, 3H, Ar-OCH_3 overlapping isomers), 3.76-3.72 (m, 1H, CHC$\underline{H}$H, major isomer), 3.69 (dd, J = 9.3, 4.0 Hz, 1H, CHC$\underline{H}$H minor isomer), 3.50 – 3.42 (m, 1H, CHCH$\underline{H}$ overlapping isomers), 3.28 (s, 3H, $CH_2OC\underline{H_3}$ major isomer), 3.26 (s, 3H, $CH_2OC\underline{H_3}$ minor isomer) 3.18 – 3.06 (m, 2H, CH_2Ar overlapping isomers), 2.65-2.47 (m, 2H, Ar-$C\underline{H_2}CH_3$ major isomer), 2.44-1.33 (m, 2.30 2H, Ar-$C\underline{H_2}CH_3$ minor isomer), 2.10 (s, 3H, Ar-CH_3, minor isomer), 2.06 (s, 3H, major isomer) 1.29 – 1.17 (m, 3H, Ar-$CH_2C\underline{H_3}$, major isomer), 1.17 – 1.06 (m, 3H, Ar-$CH_2C\underline{H_3}$ minor isomer).

^{13}C NMR (126 MHz, $CDCl_3$) δ 172.1 (CON overlapping isomers), 158.4 (C overlapping isomers), 142.7 (C minor isomer), 142.5 (C major isomer), 137.3 (C overlapping isomers), 137.0 (C overlapping isomers), 130.4 (Ar-H, overlapping isomers), 128.7 (Ar-H, overlapping isomers), 128.3 (Ar-H, overlapping isomers), 127.0 (C overlapping isomers), 126.5 (Ar-H, major isomer), 126.3 (Ar-H minor isomer), 113.7 (Ar-H, overlapping isomers), 75.2 ($\underline{C}H_2OCH_3$ overlapping isomers), 58.6 ($CH_2O\underline{C}H_3$ overlapping isomers), 55.2 (Ar-$O\underline{C}H_3$ overlapping isomers), 54.8 (CH minor isomer), 54.6 (CH major

isomer), 41.1 (CO$\underline{C}H_2$Ar major isomer), 41.0 (CO$\underline{C}H_2$Ar minor isomer) 23.8 (Ar-$\underline{C}H_2CH_3$ minor isomer), 23.6 (Ar-$\underline{C}H_2CH_3$ major isomer) , 19.0 (Ar-$\underline{C}H_3$ overlapping isomers), 15.9 (CH$\underline{C}H_3$ minor isomer) 15.6 (CH$\underline{C}H_3$ major isomer), 14.1 (Ar-$CH_2\underline{C}H_3$ major isomer), 13.7 (Ar-$CH_2\underline{C}H_3$ minor isomer).

LCMS-ESI-MS. Positive Mode. Expected mass of $[C_{22}H_{30}NO_3]^+$ 356.2220, m/z of sample 356.2221.

(2-amino-8-(*p*-methoxyphenyl) octanoyl)-L-phenylalaniline – 3m

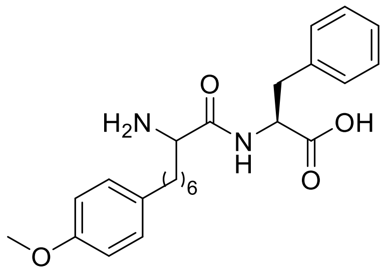

General procedure B was followed using synthesised methyl (2-amino-8-bromooctanoyl)-L-phenylalaninate (**1m**) and *p*-methoxyphenyl boronic acid (0.30 mmol, 45.6 mg). LiOH (0.5 ml, 1M) added and reaction stirred at 45 °C for 2 hours. Reaction mixture washed with Et_2O (3 x 5ml), and was adjusted to pH 4 with HCl (1M). Crude mixture subjected to reverse-phase column chromatography on Biotage Isolera Four, using H_2O (1% v/v TFA): MeOH (gradient 0 – 100%) to give product as white solid (17.3 mg, 42%).

Based on ^1H NMR and ^{13}C NMR there are two diastereomeric forms present, as with **1m** starting material. Single diastereomer (**3m d1**) isolated, and mixture of two diastereomers (**3m d1, 3m d2)** was also isolated, although **3m d2** could not be isolated and therefore was not assigned. See Supplementary Figure 2.

^1H NMR (400 MHz, MeOD) δ 7.32 – 7.17 (m, 5H, Ar-H), 7.07 (m, 2H, Ar-H), 6.86 – 6.73 (m, 2H, Ar-H), 4.68 (dd, J = 9.3, 5.1 Hz, 1H, CH), 3.79 (t, J = 6.2 Hz, 1H, CH), 3.75 (s, 3H, OCH_3), 3.25 (dd, J = 14.1, 5.1 Hz, 1H, CHC$\underline{H}$H), 3.02 (dd, J = 14.1, 9.3 Hz, 1H, CHCH$\underline{H}$), 2.54 (td, J = 7.5, 4.4 Hz, 2H, CH_2Ar), 1.91 – 1.75 (m, 2H, CH_2), 1.66 – 1.52 (m, 2H, CH_2), 1.33 (m, 6H, $CH_2CH_2CH_2$). ^{13}C NMR (101 MHz, MeOD) δ 174.2 (CO), 170.4 (CO), 159.1 (C) 138.3 (C), 135.9 (C), 130.2 (CH), 130.1 (CH), 129.5 (CH), 127.9 (CH), 114.7 (CH), 55.7 (CH), 55.4 (OCH_3), 54.3 (CH), 38.0 (CH_2), 35.8 (CH_2), 32.7 (CH_2), 32.6 (CH_2), 30.2 (CH_2), 29.8 (CH_2), 25.4 (CH_2)

LCMS-ESI-MS. Positive Mode. Expected mass of $[C_{20}H_{26}NO_4]^+$ 413.2435, m/z of sample 413.2435

FTIR (neat) cm^{-1}: 3003, (OH, br), 2920 (CH, m), 2851 (NH, m), 1668 (CO, s, br), 1612 (CO, m), 1512 (CH, m), 1440, (CH, m), 1414 (CH, m), 1188 (CO, s)

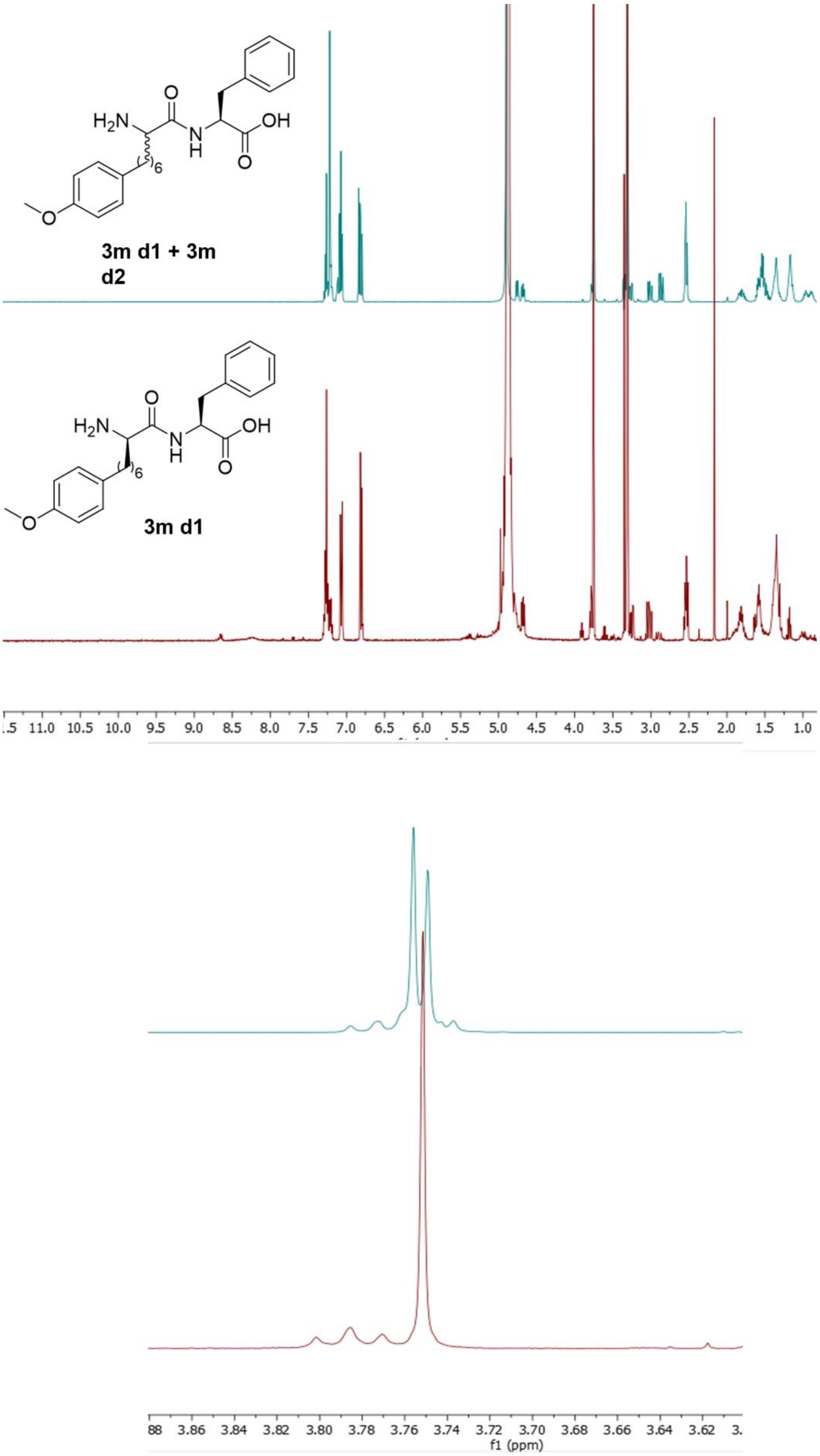

Supplementary Figure 3: Top: Overlay of (**3m d1** + **3m d2**) ^{1}H NMR in MeOD (green) and (**3m d1**) ^{1}H NMR (red). Bottom: Zoom on Overlay of (**3m d1** + **3m d2**) ^{1}H NMR in MeOD (green) and (**3m d1**) ^{1}H NMR (red) to show separation of methoxy peaks in diastereomeric mixture. Note absolute stereochemistry is not known.

2-amino-8-(2-methoxyphenyl)octanoic acid- 4b

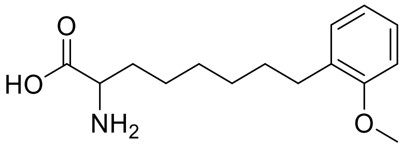

General Procedure B was followed using 2-Amino-8-bromohexanoic acid TFA salt (35.2 mg, 0.10 mmol) and *o*-methoxyphenylboronic acid (45.6 mg, 0.30 mmol). Crude mixture subjected to reverse-phase column chromatography on Biotage Isolera Four, using H_2O (1% v/v TFA): MeOH (gradient 0 – 100%) to give a white solid product. (22.9 mg, 62%).

^{1}H NMR (500 MHz, MeOD) δ 7.13 (td, J = 7.8, 1.7 Hz, 1H, Ar-H), 7.07 (dd, J = 7.4, 1.7 Hz, 1H, Ar-H), 6.88 (dd, J = 8.2, 1.0 Hz, 1H. Ar-H), 6.82 (td, J = 7.4, 1.1 Hz, 1H. Ar-H), 3.80 (s, 3H, OCH_3), 3.72 (m, 1H, CH), 2.59 (t, J = 7.8 Hz, 2H, Ar-CH_2), 1.94-1.87 (m, 1H, C<u>H</u>H), 1.86-1.78 (m, 1H, CH<u>H</u>), 1.58 (p, J = 7.4 Hz, 2H, CH_2), 1.38 (m, 6H, $CH_2CH_2CH_2$). ^{13}C NMR (126 MHz, MeOD) δ 175.3 (COOH), 158.8 (C), 131.9 (C), 130.8 (Ar-H), 128.0 (Ar-H), 121.3 (Ar-H), 111.3 (Ar-H), 55.7 (CH_3), 55.2 (CH), 32.0 (CH_2), 31.1 (CH_2), 31.0 (CH_2), 30.3 (CH_2), 30.2 (CH_2), 26.1 (CH_2)

LCMS-ESI-MS. Positive Mode. Expected mass of $[C_{15}H_{24}O_3N]^+$ 266.1751, m/z of sample 266.1746.

2-amino-8-(p-tolyl)octanoic acid- 4c

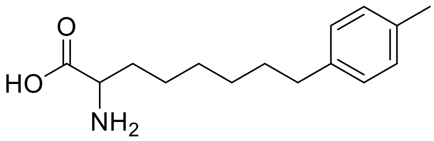

General Procedure B was followed using 2-Amino-8-bromohexanoic acid TFA salt (35.2 mg, 0.10 mmol) and *p*-tolylboronic acid (40.8 mg, 0.30 mmol). Crude mixture was adjusted to pH 4 with HCl (1M), washed with H_2O (3 x 1 ml), and subjected to reverse-phase column chromatography on Biotage Isolera Four, using H_2O (1% v/v TFA): MeOH (gradient 0 – 100%) to give a solid white product (3.8 mg, 15%).

^{1}H NMR (500 MHz, MeOD) δ 7.08 – 7.02 (m, 4H, Ar-H), 3.52 – 3.50 (m, 1H, CH), 2.55 (t, J = 7.6 Hz, 2H, C<u>H</u>$_2$Ar), 2.28 (s, 3H, Ar-CH_3), 1.91 – 1.74 (m, 2H, CH_2), 1.63 – 1.57 (m, 2H, CH_2), 1.42 – 1.32 (m, 6H, $CH_2CH_2CH_2$).^{13}C NMR (126 MHz, MeOD) δ 174.5 (COOH), 142.9 (C), 137.6 (C), 129.85 (Ar-H), 129.3 (Ar-H), 56.2 (CH), 36.4 (CH_2), 32.7 (CH_2), 32.4 (CH_2), 30.4 (CH_2), 30.0 (CH_2), 26.2 (CH_2), 19.8 (CH_3)

LCMS-ESI-MS. Positive Mode. Expected mass of $[C_{15}H_{24}NO_2]^+$ 250.1802, m/z of sample 250.1800

2-amino-8-(*o*-tolyl)-octanoic acid -4d

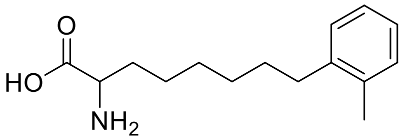

General Procedure B was followed using 2-Amino-8-bromohexanoic acid TFA salt (35.2 mg, 0.10 mmol) and *o*-tolylboronic acid (40.8 mg, 0.30 mmol). Crude mixture was carefully acidified to pH 4 using 1M HCl, H_2O removed *in vacuo*, and the solid washed with H_2O (3 x 1 ml) to give a pale yellow solid product (8.6 mg, 30%) with no further purification needed.

^{1}H NMR (500 MHz, MeOD) δ 7.22 – 6.89 (m, 4H, Ar-H), 3.93 – 3.89 (m, 1H, CH) 2.61 (t, J = 7.7 Hz, 2H, CH_2Ar), 2.29 (s, 3H, CH_3), 1.98 – 1.82 (m, 2H, CH_2), 1.62 – 1.55 (m, 2H, CH_2), 1.52 – 1.40 (m, 6H, $CH_2CH_2CH_2$). ^{13}C NMR (126 MHz, MeOD) δ 171.9 (COOH), 141.8 (C), 136.7 (C), 131.1 (CH), 129.9 (CH), 126.9 (CH), 126.8 (CH), 53.9 (CH), 34.1 (CH_2), 31.5 (CH_2), 31.4 (CH_2), 30.2 (CH_2), 30.1 (CH_2), 25.8 (CH_2), 19.4 (CH_3).

LCMS-ESI-MS. Positive Mode. Expected mass of $[C_{15}H_{24}NO_2]^+$ 250.1802, m/z of sample 250.1796

2-amino-8-phenyl-octanoic acid- 4e

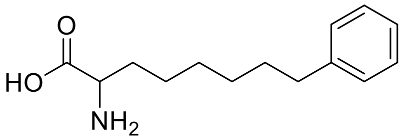

General Procedure B was followed using 2-Amino-8-bromohexanoic acid TFA salt **1a** (35.2 mg, 0.10 mmol) and phenylboronic acid (36.6 mg, 0.30 mmol). Crude mixture was adjusted to pH 4 with HCl (1M), washed with H_2O (3 x 1 ml), and subjected to reverse-phase column chromatography on Biotage Isolera Four, using H_2O (1% v/v TFA): MeOH (gradient 0 – 100%) to give a white solid (3.9 mg, 16%)

^{1}H NMR (500 MHz, MeOD) δ 7.27 – 7.21 (m, 2H, Ar-H), 7.19 – 7.10 (m, 3H, Ar-H), 3.95 (t, J = 6.2 Hz, 1H, CH), 2.61 (t, J = 7.9 Hz, 2H, CH_2Ar), 1.98 – 1.82 (m, 2H, CH_2), 1.64 (p, J = 7.5 Hz, 2H, CH_2), 1.55 – 1.46 (m, 2H, CH_2), 1.42 –1.36 (m, 4H, CH_2CH_2). ^{13}C NMR (126 MHz, MeOD) δ 171.9 (COOH), 151.2 (C), 129.39 (CH), 129.3 (CH), 126.7 (CH), 53.8 (CH), 36.8 (CH_2), 32.5 (CH_2), 31.5 (CH_2), 30.0 (CH_2), 29.9 (CH_2), 25.8 (CH_2)

LCMS-ESI-MS. Positive Mode. Expected mass of $[C_{14}H_{21}NO_2]^+$ 236.1645, m/z of sample 236.1641

2-amino-8-(4-chlorophenyl)octanoic acid- 4f

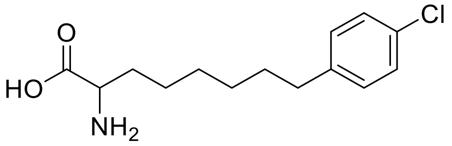

General Procedure B was followed using 2-Amino-8-bromohexanoic acid TFA salt **1a** (35.2 mg, 0.10 mmol) and 4-chlorophenyl boronic acid (46.9 mg, 0.30 mmol). Crude mixture was adjusted to pH 4 with HCl (1M), washed with Et_2O (3 x 1 ml), and subjected to reverse-phase column chromatography on Biotage Isolera Four, using H_2O (1% v/v TFA): MeOH (gradient 0 – 100%) to give a white solid (9.6 mg, 36%)

^{1}H NMR (500 MHz, MeOD) δ 7.24 (d, J = 8.4 Hz, 2H, Ar-H), 7.16 (d, J = 8.4 Hz, 2H, Ar-H), 3.94 (t, J = 6.4 Hz, 1H, CH), 2.60 (t, J = 7.6 Hz, 2H, CH_2Ar), 2.00 – 1.71 (m, 2H, CH_2), 1.61 (p, J = 7.6 Hz, 2H, CH_2), 1.52 – 1.33 (m, 6H, $CH_2CH_2CH_2$). ^{13}C NMR (126 MHz, MeOD) δ 173.4 (COOH), 142.6 (C), 132.3 (C), 141.0 (CH), 129.2 (CH), 53.9 (CH), 35.9 (CH_2), 32.2 (CH_2), 31.4 (CH_2), 29.9 (CH_2), 29.6 (CH_2), 25.6 (CH_2).

LCMS-ESI-MS. Positive Mode. Expected m/z of $[C_{14}H_{21}ClNO_2]^+$ 270.1256, m/z of sample 270.1254

2-amino-8-(naphthalen-1-yl)octanoic acid- 4g

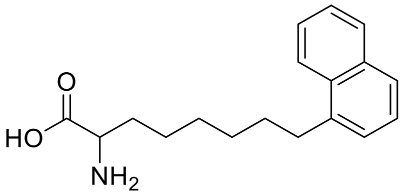

General Procedure B was followed using 2-Amino-8-bromohexanoic acid TFA salt (35.2 mg, 0.10 mmol) and napthylboronic acid (51.6 mg, 0.30 mmol Crude mixture was adjusted to pH 4 with HCl (1M), washed with Et_2O (3 x 1 ml), subjected to reverse-phase column chromatography on Biotage Isolera Four, using H_2O (1% v/v TFA): MeOH (gradient 0 – 100%) to give a white solid (3.8 mg. 12%)

^{1}H NMR (500 MHz, MeOD) δ 8.05 (d, J = 8.3 Hz, 1H, Ar-H), 7.84 (d J = 8.0 Hz, 1H), 7.70 (d, J = 8.2 Hz, 1H), 7.51 – 7.43 (m, 2H, Ar-H), 7.40 – 7.35 (m, 1H, Ar-H), 7.32 (d, J = 6.9 Hz, 1H, Ar-H), 3.95 (t, J = 6.0 Hz, 1H, CH), 3.10 (t, J = 7.7 Hz, 2H, CH_2Ar), 1.99 – 1.84 (m, 2H, CH_2), 1.76 (q, J = 7.5 Hz, 2H, CH_2), 1.51 – 1.44 (m, 6H, $CH_2CH_2CH_2$). ^{13}C NMR (126 MHz, MeOD) δ 174.8 (COOH), 139.9 (C), 135.5 (C), 133.2

(C), 129.7 (Ar-H), 127.5 (Ar-H), 127.0 (Ar-H), 126.7 (Ar-H), 126.5 (Ar-H), 126.4 (Ar-H), 124.8 (Ar-H), 56.2 (CH), 33.9 (CH_2), 32.4 (CH_2), 32.0 (CH_2), 30.5 (CH_2), 30.4 (CH_2), 26.2 (CH_2).

LCMS-ESI-MS. Positive Mode. Expected mass of $[C_{18}H_{24}NO_2]^+$ 286.1802, m/z of sample 286.1794

2-amino-8-(4-aminophenyl)octanoic acid hydrochloride salt- 4h

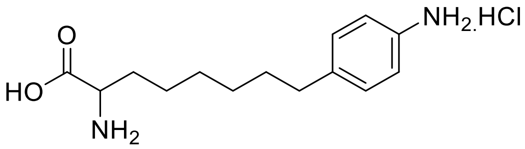

General Procedure B was followed using 2-Amino-8-bromohexanoic acid TFA salt (35.2 mg, 0.10 mmol) and *p*-aminophenyl boronic acid hydrochloride salt (52.3 mg, 0.30 mmol). Crude mixture subjected to reverse-phase column chromatography on Biotage Isolera Four, using H_2O (1% v/v TFA): MeOH (gradient 0 – 100%) to give a white solid (9.1 mg, 30%).

^{1}H NMR (500 MHz, MeOD) δ 7.56 (dd, J = 8.4, 6.9 Hz, 2H, Ar-H), 7.51 – 7.42 (m, 2H, Ar-H), 3.95 (t, J = 6.3 Hz, 1H, CH), 3.38 (t, J = 7.9 Hz, 2H, CH_2Ar), 1.95 – 1.86 (m, 2H, CH_2), 1.74 (p, J = 7.5 Hz, 2H, CH_2), 1.54 – 1.45 (m, J = 14.9 Hz, 6H, $CH_2CH_2CH_2$). ^{13}C NMR (126 MHz, MeOD) δ 171.9 (COOH), 144.2 (C), 131.5 (CH), 130.2 (C), 123.2 (CH), 53.8 (CH), 52.7 (CH_2Ar), 31.4 (CH_2), 29.7 (CH_2), 27.2 (CH_2), 27.2 (CH_2), 25.8 (CH_2)

LCMS-ESI-MS. Positive Mode. Expected mass of $[C_{14}H_{23}N_2O_2]^+$ 251.1754, m/z of sample 251.1751

±*4*-(7-((tert-butoxycarbonyl)amino)-7-carboxyheptyl)benzoic acid- *N*-Boc 4i

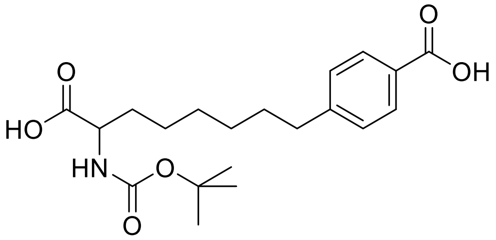

General Procedure C was followed using 2-Amino-8-bromohexanoic acid TFA salt (35.2 mg, 0.10 mmol) and *p*-carboxyphenylboronic acid (49.8 mg, 0.30 mmol). Crude mixture purified by flash column chromatography using DCM (1% v/v Toluene): MeOH (1% v/v Toluene) (gradient 0 – 10%) (10% MeOH in DCM, Rf = 0.21) to give a colourless solid (8.5 mg, 22%)

^{1}H NMR (500 MHz, $CDCl_3$) δ 7.97 (d, J = 8.5 Hz, 2H, Ar-H), 7.27 – 7.22 (d, J = 8.4 Hz, 2H, Ar-H), 5.02 (d, J = 8.1 Hz, 1H, NH), 4.34 (d, J = 5.9 Hz, 1H, CH), 2.73 – 2.60 (m, 2H, Ar-CH_2), 1.81 (m, 1H, CHH), 1.72 (m, 1H, CHH), 1.67 – 1.59 (m, 2H, CH_2), 1.45 (s, 9H, $C(CH_3)_3$), 1.37 – 1.25 (m, 6H, $CH_2CH_2CH_2$). ^{13}C NMR

(126 MHz, $CDCl_3$) δ 178.1 (COOH), 171.9 (COOH), 160.8 (COO^tBu), 149.4 (C), 140.4 (C), 130.4 (Ar-H), 128.8 (Ar-H), 86.1 C(CH_3) 53.5 (CH), 36.0 (CH_2), 32.3 ($CH_{2)}$, 30.8 (CH_2), 29.1 (CH_2), 28.7 (CH_2), 28.5 (C(CH_3)$_3$), 24.9 (CH_2)

LCMS-ESI-MS. Positive Mode. Expected mass of $[C_{20}H_{29}NO_6Na]^+$ 402.1884, m/z of sample 402.1884..

4-(7-amino-7-carboxyheptyl)benzoic acid- 4i

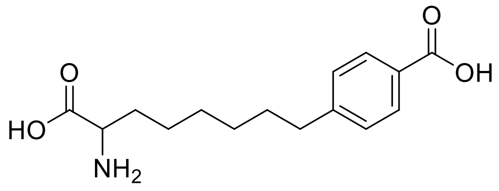

General Procedure C was followed using 2-Amino-8-bromohexanoic acid TFA salt (35.2 mg, 0.10 mmol) and *p*-carboxyphenylboronic acid (49.8 mg, 0.30 mmol). Crude mixture purified by flash column chromatography DCM : MeOH (0.1 % acetic acid. 1% toluene) to give a colourless solid (8.5 mg, 22%). ±N-Boc-2-amino-8-(*p*-carboxyphenyl)-octanoic acid (5 mg) dissolved in DCM (0.5 ml), TFA (5 ml) added and stirred at room temperature overnight to give quantitative conversion to deprotected 2-amino-8-(*p*-carboxyphenyl)-octanoic acid. (1 drop of HCl (1M) was required for solubility in MeOD for NMR analysis)

^{1}H NMR (500 MHz, MeOD) δ 7.92 (d, J = 7.9 Hz, 2H, Ar-H), 7.30 (d, J = 7.9 Hz, 2H, Ar-H), 3.94 (d, J = 6.6 Hz, 1H, CH), 2.69 (t, J = 7.6 Hz, 2H, CH_2Ar), 1.91 (dq, J = 13.0, 6.5, 5.6 Hz, 2H CH_2), 1.76 – 1.61 (m, 2H, CH_2), 1.55 – 1.31 (m, 6H, $CH_2CH_2CH_2$). ^{13}C NMR (126 MHz, MeOD) δ 172.1 (COOH), 170.2 (COOH), 149.8 (C), 130.8 (Ar-H), 129.6 (Ar-H), 129.2 (C), 53.9 (CH), 36.7 (CH_2-Ar), 32.0 (CH_2), 31.5 (CH_2), 29.9 (CH_2), 29.7 (CH_2), 25.7 (CH_2).

LCMS-ESI-MS. Positive Mode. Expected mass of $[C_{15}H_{22}NO_4]^+$ 280.1543, m/z of sample 280.1537

FTIR (neat) cm^{-1}: 3005 (OH, b), 2926 (CH, m), 2857 (NH, br) 1734 (CO, s), 1715 (CO, s), 1697 (CO, s) 1483 (CH, m), 1468 (CH, m), 1416 (CH, m), 1242 (CO, s)

2-amino-8-(3-aminophenyl)octanoic acid- 4j

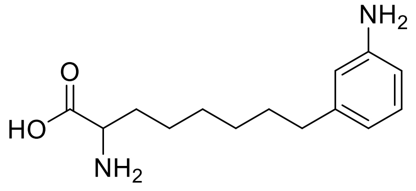

General Procedure B was followed using 2-Amino-8-bromohexanoic acid TFA salt (35.2 mg, 0.10 mmol) and *m*-aminophenyl boronic acid (41.1 mg, 0.30 mmol). Crude mixture subjected to reverse-

phase column chromatography on Biotage Isolera Four, using H_2O (1% v/v TFA): MeOH (gradient 0 – 100%) to give a white solid (26.6 mg, 93%).

^{1}H NMR (500 MHz, MeOD) δ 7.89 – 7.73 (m, 2H), 7.62 – 7.48 (m, 2H), 3.96 (t, J = 6.3 Hz, 1H), 3.40 (t, J = 7.9 Hz, 2H), 2.02 – 1.82 (m, 2H), 1.83 – 1.73 (m, 2H), 1.52-1.41 (m, 6H). ^{13}C NMR (126 MHz, MeOD) δ 171.8 (COOH), 146.9 (C-N), 136.3 (C), 136.0 (CH), 130.8 (CH), 128.6 (CH), 124.9 (CH), 53.8, (CH) 53.3 (<u>C</u>H_2Ar), 31.4 (CH_2), 29.7 (CH_2), 27.1 (CH_2), 27.0 (CH_2), 25.8 (CH_2).

LCMS-ESI-MS. Positive Mode. Expected mass of $[C_{14}H_{23}N_2O_2]^+$ 251.1754, m/z of sample 251.1754.

2-amino-8-(2-fluorophenyl)-octanoic acid- 4k

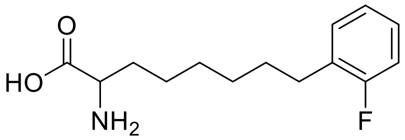

General Procedure B was followed using 2-Amino-8-bromohexanoic acid TFA salt (35.2 mg, 0.10 mmol) and *o*-fluorophenyl boronic acid (31.9 mg, 0.30 mmol). Crude product subjected to reverse-phase column chromatography on Biotage Isolera Four, using H_2O (1% v/v TFA): MeOH (gradient 0 – 100%) to give a white solid (11.4 mg, 44%).

^{1}H NMR (500 MHz, MeOD) δ 7.25 – 7.15 (m, 2H, Ar-H), 7.07 (td, J = 7.4, 1.2 Hz, 1H, Ar-H), 7.00 (ddd, J = 10.5, 8.1, 1.2 Hz, 1H, Ar-H), 3.95 (t, J = 6.2 Hz, 1H, CH), 2.65 (t, J = 7.7 Hz, 2H, CH_2Ar), 1.98 – 1.81 (m, 2H, CH_2), 1.63 (p, J = 7.4 Hz, 2H, CH_2), 1.53 – 1.36 (m, 6H, $CH_2CH_2CH_2$). ^{13}C NMR (126 MHz, MeOD) δ 171.9 (COOH), 162.5 (d, J_{C-F} = 243.0 Hz, CF), 131.9 (d, J_{C-F} = 5.6 Hz, CH) , 130.4 (d, J_{C-F} = 16.0 Hz, C), 128.7 (d, J_{C-F} = 7.9 Hz, CH), 125.1 (d, J_{C-F} = 3.5 Hz, CH), 116.0 (d, J_{C-F} = 22.2 Hz, CH), 53.9 (CH), 31.5 (CH_2), 31.3 (CH_2), 31.2 (CH_2), 29.9 (CH_2), 29.8 (CH_2), 29.7 (CH_2), 25.7 (CH_2). ^{19}F NMR (471 MHz, MeOD) δ -121.4.

LCMS-ESI-MS. Positive Mode. Expected mass of $[C_{14}H_{21}FNO_2]^+$ 254.1551, m/z of sample 254.1552.

2-amino-8-(3-((5-(dimethylamino)naphthalene)-1-sulfonamido)phenyl)octanoic acid- 4m

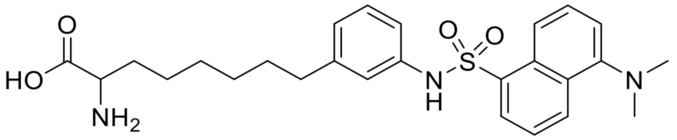

General procedure B was followed using 2-Amino-8-bromohexanoic acid TFA salt (35.2 mg, 0.10 mmol) and 3-(dansylamino)phenylboronic acid (44.4 mg, 0.12 mmol). Crude mixture subjected to

reverse-phase column chromatography on Biotage Isolera Four using H_2O (1% v/v TFA): MeOH (gradient 0 – 100%) to give pure product (8.2 mg, 17% yield)

^{1}H NMR (400 MHz, MeOD) δ 8.49 (d, J = 8.6 Hz, 1H, Ar-H), 8.42 (d, J = 8.6 Hz, 1H, Ar-H) 8.15 (dd, J = 6.2, 1.2 Hz, 1H, Ar-H), 7.65 – 7.53 (m, 1H, Ar-H), 7.53 – 7.45 (m, 1H, Ar-H), 7.32 (d, J = 7.6 Hz, 1H, Ar-H), 6.97 (t, J = 7.8 Hz, 1H, Ar-H), 6.83 (s, 1H, Ar-H), 6.79 – 6.68 (m, 2H, Ar-H, Ar-H), 3.94 (t, J = 6.2 Hz, 1H, CH), 2.90 (d, J = 1.3 Hz, 6H, $N(CH_3)_2$), 2.42 (t, J = 7.5 Hz, 2H, CH_2Ar), 1.92-1.82 (m, 2H, CH_2), 1.67-1.49 (m, 2H, CH_2), 1.48-1.41 (m, 4H, CH_2CH_2), 1.37-1.33 (m, 2H, CH_2) ^{13}C NMR (126 MHz, MeOD) δ 170.5 (COOH), 150.5 (C), 143.4 (C), 137.2 (C), 135.3 (C), 132.5 (C), 130.0 (Ar-H), 128.4 (Ar-H), 127.8 (Ar-H), 124.3 (Ar-H), 123.4 (Ar-H), 123.0 (C), 120.4 (Ar-H), 119.8 (Ar-H), 117.9 (Ar-H), 115.4 (C), 52.5 (CH), 44.6 ($N(CH_3)_2$), 35.0 (CH_2), 30.7 (CH_2), 30.1 (CH_2), 28.6 (CH_2), 28.2 (CH_2), 24.4 (CH_2).

LCMS-ESI-MS. Positive Mode. Expected mass of $[C_{26}H_{34}N_3O_4S]^+$ 484.2265, m/z of sample 484.2264.

Fluorescence analysis was performed on **4m** using general UPLC procedure 1.7. λ_{Ex} 337 nm, λ_{Em} 530 nm, retention time 2.6 min.

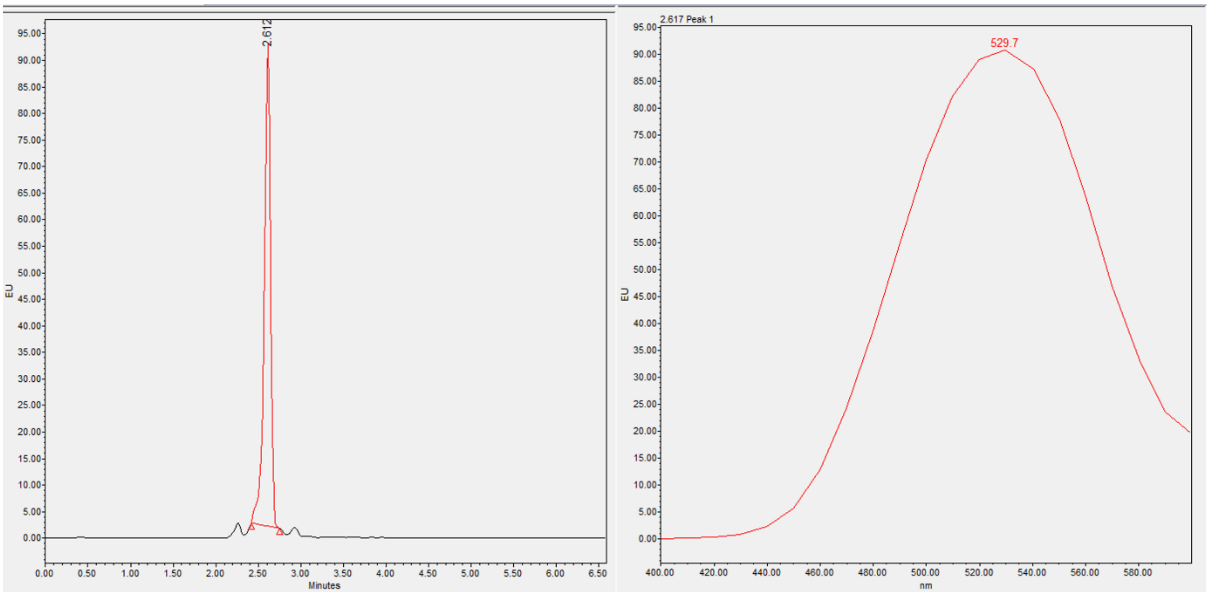

Supplementary Figure 4: Fluorescence spectra of **4m** on UPLC. Ex337/ Em400-600 nm.

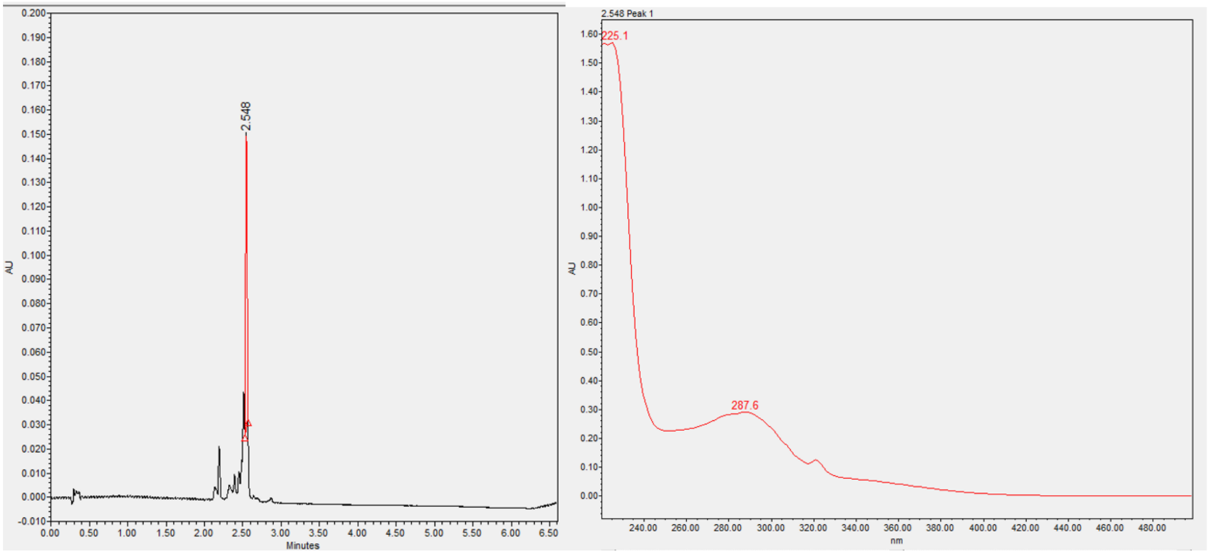

Supplementary Figure 5: UV absorption spectra of 5m on UPLC. PDA 220-500 nm.

1-(*m*-aminophenyl) Bromosphaerol – 5b

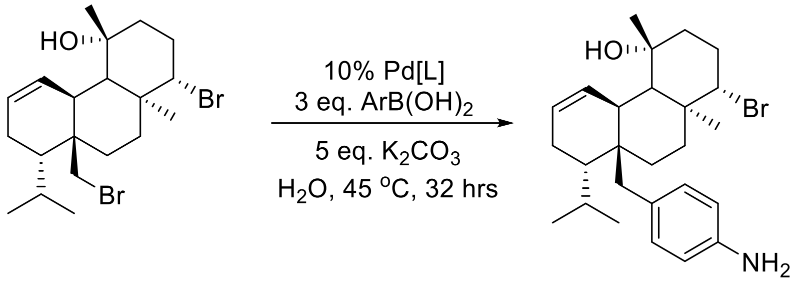

General procedure D followed with Bromosphaerol (448 µg, 1 µmol) (provided by Professor Vassilios Roussis, extract from *Sphaerococcus coronopifolius)* and *p*-aminophenyl boronic acid hydrochloride (867 µg, 5 µmol) where [L] is $[TXPTS]_2$.

LCMS-ESI-MS. Positive mode. Expected mass of $[C_{26}H_{39}{}^{79}BrNO]^+$ 460.2209. m/z of sample 460.2205. Expected mass of $[C_{26}H_{39}{}^{81}BrNO]^+$ 462.2189. m/z of sample 462.2185

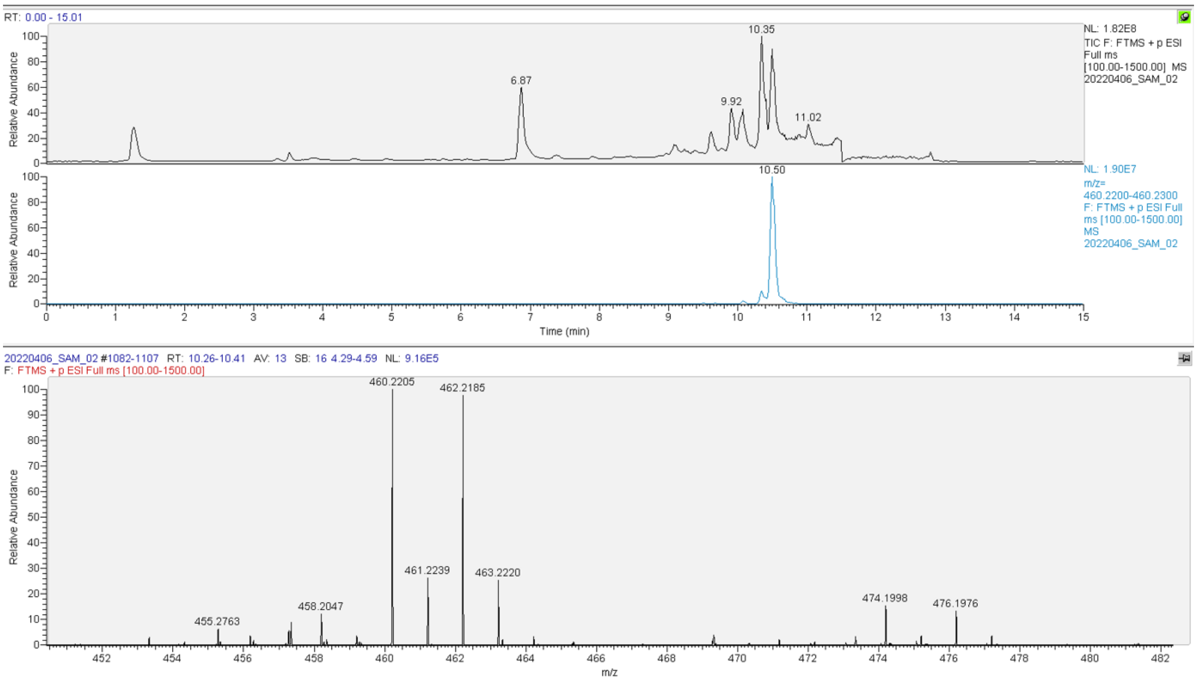

1-(3-dansylamino benzene) Bromosphaerol – 5c

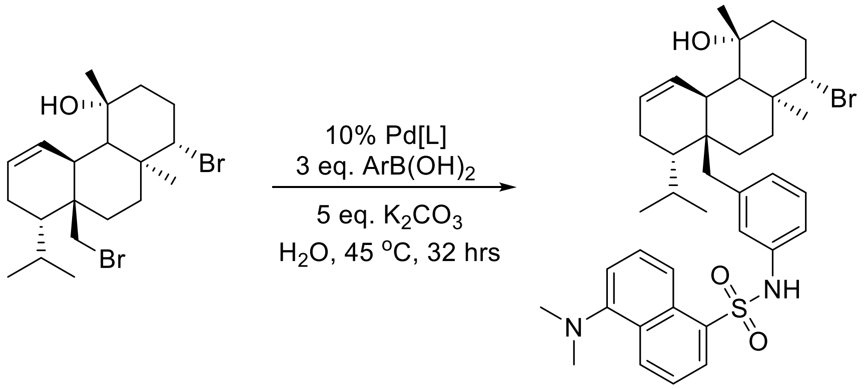

General procedure D followed with Bromosphaerol (448 µg, 1 µmol) and 3-(dansylamino)phenylboronic acid (1.88 mg, 5 µmol) where [L] is $[TXPTS]_2$.

LCMS-ESI-MS. Positive mode. Expected mass of $[C_{38}H_{50}{}^{79}BrN_2O_3S]^+$ 693.2720. m/z of sample 693.2737 Expected mass of $[C_{38}H_{50}{}^{81}BrN_2O_3S]^+$ 695.2713. m/z of sample 695.2713

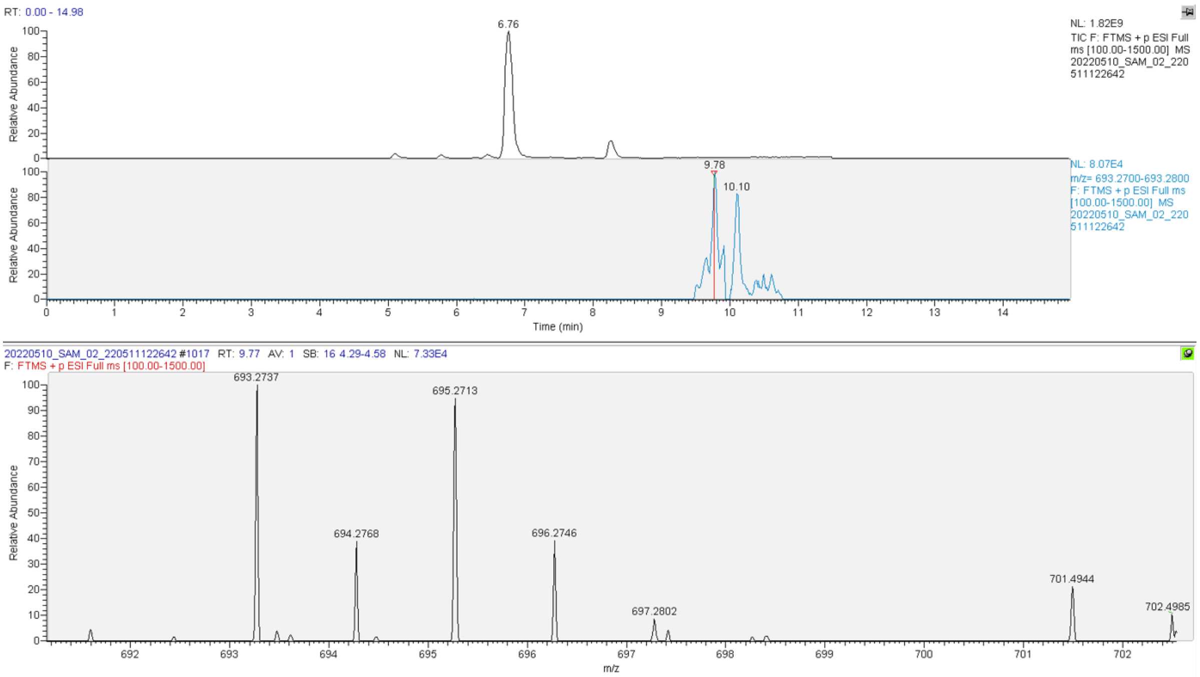

1-(*m*-aminophenyl Alcyopterosin A- 6b

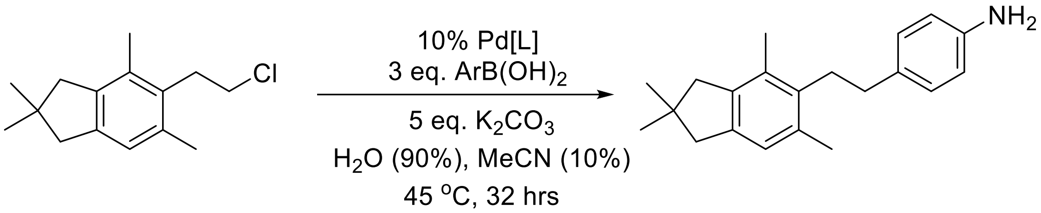

General procedure D followed with Alcyopterosin A (263 µg, 1 µmol) and *p*-aminophenyl boronic acid hydrochloride (867 µg, 5 µmol) where [L] is $[TXPTS]_2$.

LCMS-ESI-MS. Positive mode. Expected mass of $[C_{21}H_{28}N]^+$ 294.2216, m/z of sample 294.2206

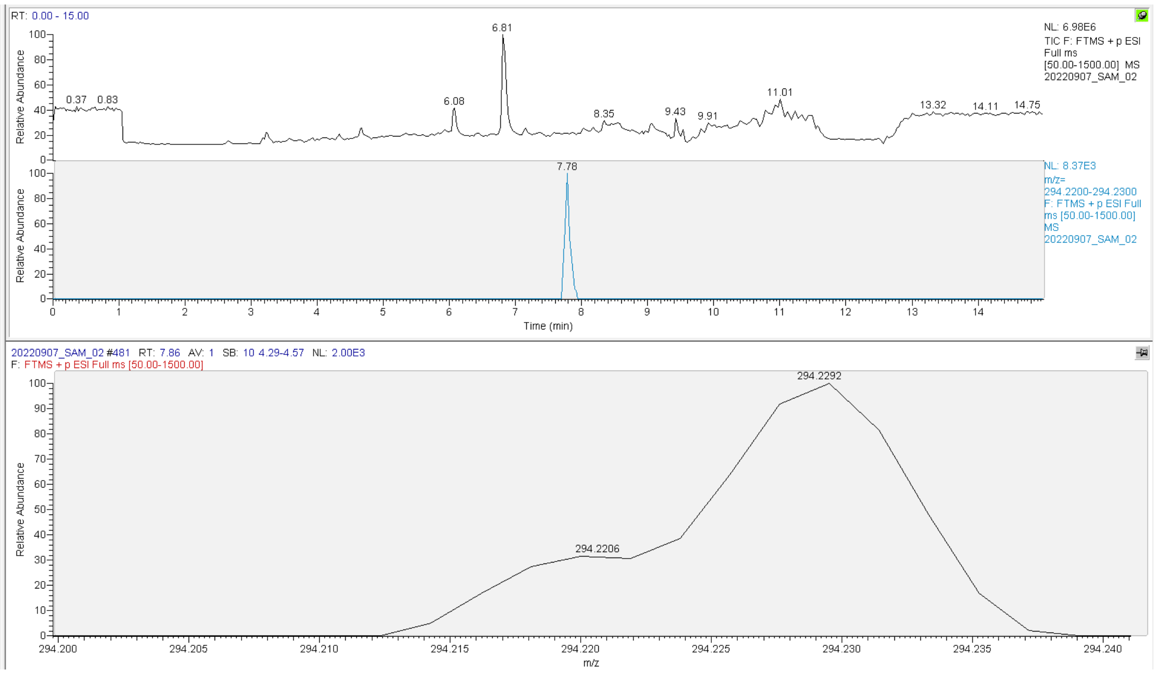

3. Synthetic procedures and compounds

Diethyl-2-acetamido-2-(6-bromohexyl)malonate

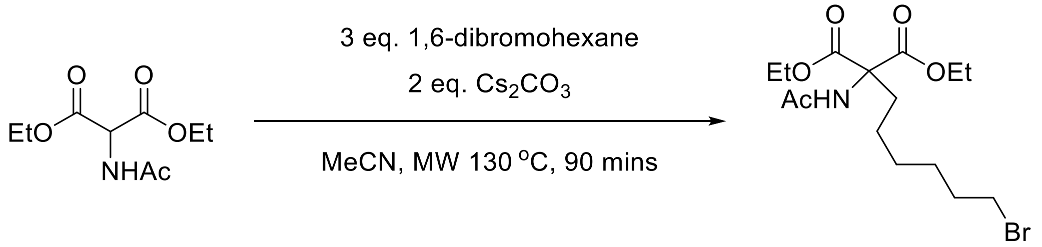

N-Acetyl diethylamino malonate (1.09 g, 5 mmol) and caesium carbonate (3.26 g, 10 mmol) were added to a MW vial and charged with a stirrer bar. Dry MeCN (15 ml) was added, followed by 1,6-dibromohexane (2.45 ml, 15 mmol). The vial was sealed and heated in the MW for 90 minutes at 130 °C. The reaction mixture was then filtered under reduced pressure, and the filtrate concentrated *in vacuo* to give a yellow oil. Crude product was either purified by flash column chromatography using Pet Ether: EtOAc (gradient 0 – 100%) (50% EtOAc in Pet Ether, Rf = 0.36) to give a colourless oil (1.36 g, 72%) or taken forward to hydrolysis as crude.

^{1}H NMR (500 MHz, $CDCl_3$) δ 6.77 (s, 1H, NH), 4.24 (q, J = 7.1 Hz, 4H, OCH_2), 3.37 (t, J = 6.8 Hz, 2H, CH_2Br), 2.40 – 2.26 (m, 2H), 2.03 (s, 3H, $COCH_3$), 1.88 – 1.74 (m, 2H), 1.47 – 1.34 (m, 2H), 1.34 – 1.28 (m, 2H), 1.25 (t, J = 7.1 Hz, 6H, CH3), 1.16 – 1.01 (m, 2H). ^{13}C NMR (126 MHz, $CDCl_3$) δ 169.1 (CO), 168.3 (CO), 66.6 (C), 62.6 (CH_2), 33.9 (CH_2), 32.7 (CH_2), 32.1 (CH_2), 28.5 (CH_2), 28.1 (CH_2), 23.8 (CH_2), 23.2 (CH_3), 14.1 (CH_3). These data are consistent with the reported values. [5]

2-acetamido-8-bromo-2-(ethoxycarbonyl)octanoic acid

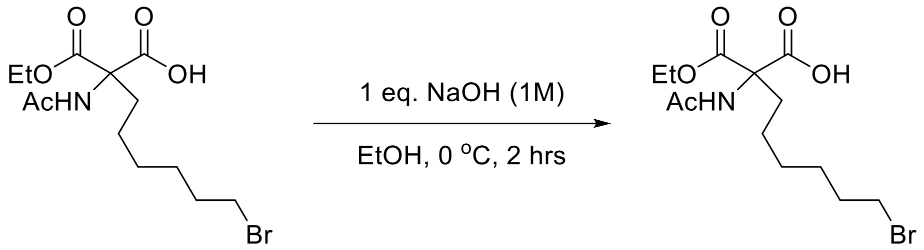

Crude mixture of diethyl-2-acetamido-2-(6-bromohexyl)malonate from the previous step was dissolved in absolute EtOH (10 ml) and cooled on ice for 20 minutes. A cooled solution of NaOH (1M, 5 ml) was added slowly at 0 °C over 2 hours in 1 ml portions. The reaction mixture was then diluted with H_2O (15 ml) and the EtOH removed *in vacuo*. The aqueous mixture was then washed with Et_2O (25 ml c 1), acidified with 1M citric acid solution to pH 2, and extracted with EtOAc (20 ml x 3). The combined organic layers were washed with H_2O (20 ml), brine (20 ml), dried over $MgSO_4$, filtered, and solvent removed *in vacuo* to give the product as a white powder (1.26 g, 71% over two steps).

^1H NMR (500 MHz, $CDCl_3$) δ 6.86 (s, 1H, NH), 4.29 (qd, J = 7.1, 2.4 Hz, 2H, $OC\underline{H_2}CH_3$), 3.38 (t, J = 6.8 Hz, 2H, CH_2Br), 2.27 (m, 2H, $C\underline{H_2}C(C{=}O)_2$), 2.09 (s, 3H, NH$\underline{Ac}$), 1.94 – 1.66 (m, 2H, CH_2), 1.51 – 1.36 (m, 2H, CH_2), 1.36 – 1.30 (m, 2H, CH_2), 1.29 (t, J = 7.1 Hz, 3H, $OCH_2C\underline{H_3}$), 1.25 – 1.17 (m, 1H, C$\underline{H}$H), 1.16 – 1.08 (m, 1H, CH$\underline{H}$). ^{13}C NMR (126 MHz, $CDCl_3$) δ 171.1 (CO), 169.5 (CO), 169.2 (CO), 66.6 (C), 63.3 (CH_2), 33.9 (CH_2), 32.7 (CH_2), 28.5 (CH_2), 28.0 (CH_2), 23.6 (CH_2), 23.1 ($COCH_3$), 14.1 (CH_3). These data are consistent with the reported values. [5]

Ethyl 2-acetamido-8-bromooctanoate

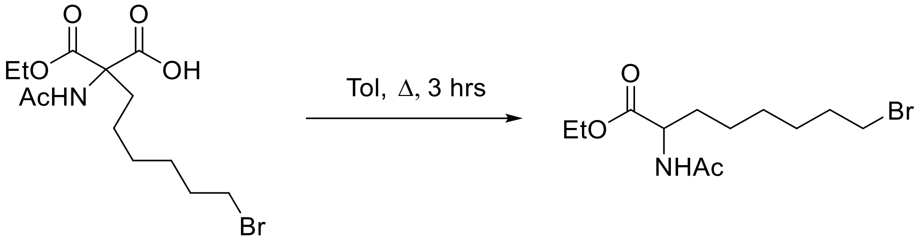

2-acetamido-8-bromo-2-(ethoxycarbonyl)octanoic acid (1.26 g, 3.56 mmol) was suspended in toluene (20 ml), and refluxed at 115 °C for 3 hours to give a clear yellow solution. Solvent was removed *in vacuo* to give quantitative conversion to product (1.08g, 99%).

^1H NMR (500 MHz, $CDCl_3$) δ 6.03 (d, J = 8.1 Hz, 1H, NH), 4.58 (ddd, J = 8.1, 7.2, 5.3 Hz, 1H, CH), 4.19 (q, J = 7.1 Hz, 2H, $OC\underline{H_2}CH_3$), 3.39 (t, J = 6.8 Hz, 2H, $C\underline{H_2}Br$), 2.02 (s, 3H, NH$\underline{Ac}$), 1.89 – 1.77 (m, 3H, $C\underline{H_2}C\underline{H}H$), 1.71 – 1.58 (m, 1H, $CH_2CH\underline{H}$), 1.46 – 1.37 (m, 2H, $C\underline{H_2}$), 1.39 – 1.29 (m, 4H), 1.27 (t, J = 7.2 Hz, 3H, $OCH_2C\underline{H_3}$) ^{13}C NMR (126 MHz, $CDCl_3$) δ 172.8 (C=O), 169.9 (C=O), 61.6 (CH), 52.2 (CH_2), 34.0 (CH_2), 32.7 (CH_2), 32.6 (CH_2), 28.4 (CH_2), 28.0 (CH_2), 25.1 (CH_2), 23.4 (CH_3), 14.3 (CH_3). These data are consistent with the reported values. [5]

2-acetamido-8-bromooctanoic acid-1b

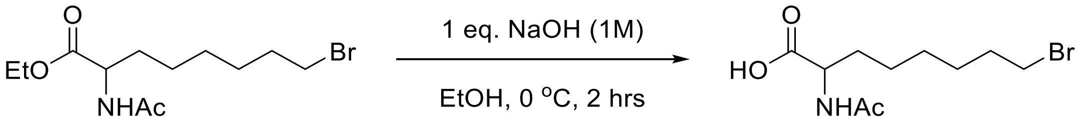

Ethyl 2-acetamido-8-bromooctanoate dissolved in absolute EtOH (10 ml) and cooled on ice for 20 minutes. A cooled solution of aqueous NaOH (1M, 5 ml) was added slowly at 0 °C over 2 hours in 1 ml portions. Once the reaction was complete, the reaction mixture was diluted with H_2O (15 ml) and the EtOH removed *in vacuo*. The aqueous mixture was then washed with Et_2O (25 ml x 1), acidified with 1M citric acid solution to pH 2, and extracted with EtOAc (20 ml x 3). The combined organic layers were washed with H_2O (20 ml), brine (20 ml), dried over $MgSO_4$, filtered under reduced

pressure, and solvent removed *in vacuo* to give a yellow oil. Trituration with cold Et_2O gave the pure product as a white powder (605 mg, 61% over two steps).

^{1}H NMR (500 MHz, $CDCl_3$) δ 6.26 (d, J = 7.7 Hz, 1H, NH), 4.58 (q, J = 6.8 Hz, 1H, CH), 3.40 (t, J = 6.8 Hz, 2H, CH_2Br), 2.06 (s, 3H, NH$\underline{Ac}$), 1.86 (m, 3H, $C\underline{H}_2CH_2Br$ + $CHC\underline{H}H$), 1.71 (m, 1H, $CHCH\underline{H}$), 1.53 – 1.40 (m, 2H, CH_2), 1.39 – 1.25 (m, 4H, CH_2CH_2). ^{13}C NMR (126 MHz, $CDCl_3$) δ 175.6 (C=O), 171.3 (C=O), 52.5 (CH), 34.0 (CH_2), 32.7 (CH_2), 32.0 (CH_2), 28.4 (CH_2), 28.0 (CH_2), 25.2 (CH_2), 23.1 (CH_3). These data are consistent with the reported values. [5]

2-amino-8-chlorooctanoic acid hydrochloride- 1f

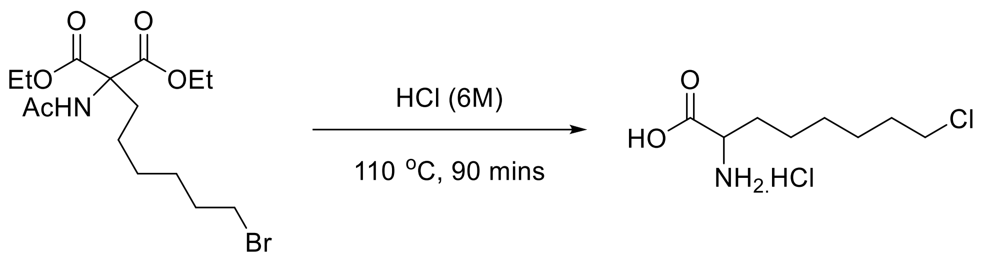

Diethyl-2-acetamido-2-(6-bromohexyl)malonate (1.36 g, 3.58 mmol,) was charged into a MW vial along with a stirrer bar, and suspended in HCl (6M, 4 ml). The reaction mixture was heated in the MW at 110 °C for 90 minutes to give a yellow solution. HCl was then removed *in vacuo* to give a yellow solid. Crude product was purified by reverse-phase column chromatography using H_2O (1% v/v TFA): MeOH (gradient 0 – 100%) to give a white solid (353 mg, 42%).

^{1}H NMR (400 MHz, MeOD) δ 3.86 (t, J = 6.2 Hz, 1H, CH), 3.57 (t, J = 6.6 Hz, 2H, CH_2Cl), 2.05 – 1.83 (m, 2H, CH_2), 1.84 – 1.70 (m, 2H, CH_2), 1.57 – 1.34 (m, 6H, $CH_2CH_2CH_2$). ^{13}C NMR (101 MHz, MeOD) δ 173.4 (COOH), 54.5 (CH), 46.0 (CH_2), 33.2 (CH_2), 31.3 (CH_2), 29.1 (CH_2), 27.2 (CH_2), 25.5 (CH_2).

LCMS-ESI-MS. Positive Mode. Expected mass of $[C_8H_{17}O_2N^{35}Cl]^+$ 193.0942, m/z of sample 194.0938. Expected mass of $[C_8H_{17}O_2N^{37}Cl]^+$ 195.0913, m/z of sample 196.0910. These data are consistent with the reported values.[5]

FTIR (neat) cm^{-1}: 3013 (OH, br), 2914 (CH, m), 2854 (NH, br) 1730 (CO, s), 1487 (CH, m), 1458 (CH, m), 1420 (CH, m), 762 (CCl, m)

Diethyl 2-((tert-butoxycarbonyl)amino)malonate

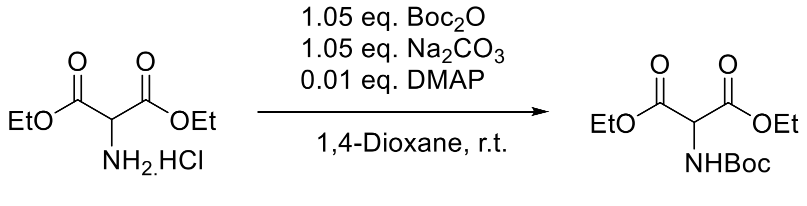

Diethylamino malonate hydrochloride (1.06 g, 5 mmol) was dissolved in dioxane (10 ml) and H_2O (6 ml). Sodium carbonate (452 mg, 5.25 mmol) was added slowly with stirring at room temperature until the reaction mixture became clear. DMAP (6.1 mg, 0.05 mmol) was added, followed by the dropwise addition of a solution of di-tertbutyl dicarbonate (1.15 g, 5.25 mmol) in dioxane (5 ml). The reaction mixture was stirred at room temperature and followed by TLC (DCM: 1% MeOH). Once the reaction was complete the solvent was removed *in vacuu*, and the residue dissolved in EtOAc (30 ml), washed with 5% $KHSO_4$ (20 ml), saturated $NaHCO_3$ (20 ml), H_2O (20 ml), brine (20 ml), dried over Na_2SO_4, filtered under reduced pressure, and concentrated *in vacuo*, to give the product as a colourless liquid (1.04 g, 66%)

^{1}H NMR (400 MHz, $CDCl_3$) δ 5.55 (d, J = 7.7 Hz, 1H, NH), 4.94 (d, J = 7.7 Hz, 1H, CH), 4.26 (ddp, J = 14.2, 7.1, 3.7 Hz, 4H, OCH_2), 1.44 (s, 9H, NHBoc), 1.29 (t, J = 7.1 Hz, 6H, OCH_2CH_3). ^{13}C NMR (126 MHz, $CDCl_3$) δ 166.8 (COOEt), 154.9 (COONH), 80.8 ($C(CH_3)_3$) 62.6 (CH_2CH_3), 57.7 (CH), 28.4 (CH_2CH_3), 14.2 ($C(CH_3)_3$) These data are consistent with the reported values.[6]

8-bromo-2-((tert-butoxycarbonyl)amino)-2-(ethoxycarbonyl)octanoic acid

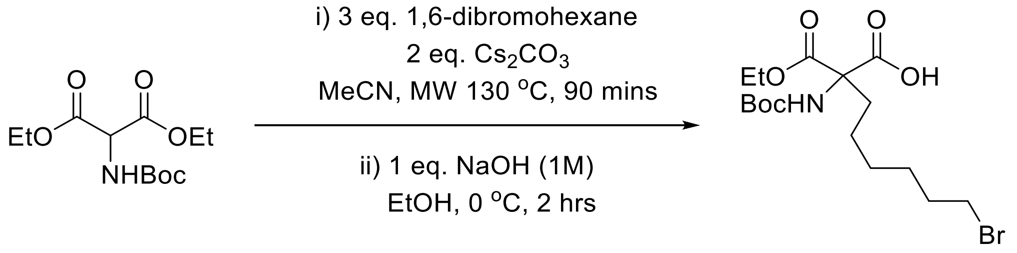

Diethyl 2-((tert-butoxycarbonyl)amino)malonate (1.03 g, 3.32 mmol) and caesium carbonate (2.16 g, 6.64 mmol) was added to a 20 ml MW vial and charged with a stirrer bar. Dry MeCN (10 ml) was added, followed by 1,6-dibromohexane (1.53 ml, 9.96 mmol). The vial was sealed and heated in the MW for 90 minutes at 130 °C. The reaction mixture was then filtered under reduced pressure, and the filtrate concentrated *in vacuo* to give a yellow oil. Crude oil was dissolved in absolute EtOH (10 ml) and cooled on ice for 20 minutes. A cooled solution of NaOH (1M, 4 ml) was added slowly at 0 °C over 2 hours in 1 ml portions. Once the reaction was complete, the reaction mixture was diluted H_2O (15 ml) and the EtOH removed *in vacuo*. The aqueous mixture was then washed with Et_2O (25 ml x 1), acidified with 1M citric acid solution to pH 2, and extracted with EtOAc (20 ml x 3). The combined organic layers were washed with H_2O (20 ml), brine (20 ml), dried over $MgSO_4$, filtered under reduced pressure, and solvent removed *in vacuo* to give thee product as a yellow oil (1.054 g, 77% over two steps)

^{1}H NMR (400 MHz, $CDCl_3$) δ 5.72 (s, 1H, NH), 4.32 (q, J = 7.2, 2H, OCH_2CH_3), 3.41 (t, J = 6.7 Hz, 2H, CH_2Br), 2.19 (m, 2H, CH_2), 1.86 (p, J = 6.9 Hz, 2H, CH_2), 1.46 (s, 9H, NHBoc), 1.43 – 1.24 (m, 9H,

$CH_2CH_2CH_2$ + $OCH_2C\underline{H_3}$). ^{13}C NMR (126 MHz, $CDCl_3$) δ 171.0 (COOH), 169.7 (COOEt), 155.1 (NHCO), 66.2 ($\underline{C}(CH_3)$), 63.1 (OCH_2), 33.9 (CH_2), 33.5 (CH_2), 32.7 (CH_2), 28.5 ($C(CH_3)_3$), 28.3 (CH_3), 27.9 (CH_2), 23.4 (CH_2), 14.1 ($OCH_2\underline{C}H_3$)

LCMS-ESI-MS. Positive Mode. Expected mass of sodium adduct $[C_{16}H_{28}{}^{79}BrNNaO_6]^+$ 432.0992, m/z found 432.0983, expected mass of sodium adduct $[C_{16}H_{28}{}^{81}BrNNaO_6]^+$ 434.0972, m/z of sample 434.0966.

8-bromo-2-((tert-butoxycarbonyl)amino)octanoic acid-1d

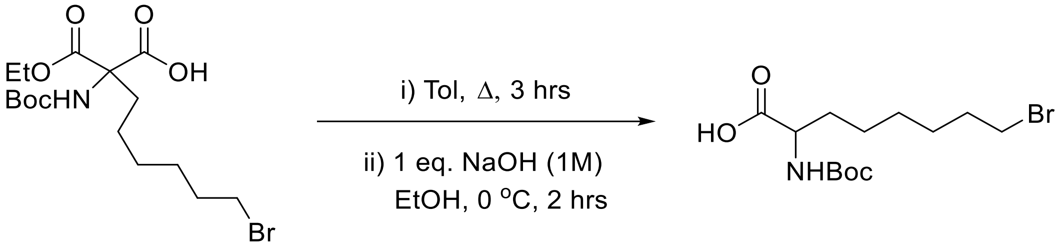

8-bromo-2-((tert-butoxycarbonyl)amino)-2-(ethoxycarbonyl)octanoic acid (1.054 g, 2.57 mmol) was suspended in toluene (20 ml), and refluxed at 115 °C for 3 hours to give a clear yellow solution. Solvent was removed *in vacuo* to give quantitative conversion to product yellow oil. Oil was dissolved in absolute EtOH (10 ml) and cooled on ice for 20 minutes. A cooled solution of NaOH (1M, 5 ml) was added slowly at 0 °C over 2 hours in 1 ml portions. Once the reaction was complete, the reaction mixture was diluted with H_2O (15 ml) and the EtOH removed *in vacuo*. The aqueous mixture was then washed with ether (25 ml x 1), acidified with 1M citric acid solution to pH 2, and extracted with EtOAc (20 ml x 3). The combined organic layers were washed with H_2O (20 ml), brine (20 ml), dried over $MgSO_4$, filtered under reduced pressure, and solvent removed *in vacuo* to give a yellow oil. Trituration with cold Et_2O gave the product as a yellow residue (346 mg, 39% over two steps).

^1H NMR (400 MHz, $CDCl_3$) δ 8.37 (s, 1H), 5.13 – 4.93 (m, 1H, NH), 4.30 (d, J = 6.4 Hz, 1H, CH), 3.40 (t, J = 6.8 Hz, 2H, CH_2Br), 1.85 (m, 3H, $C\underline{H_2}CH_2Br$ + $CHC\underline{H}H$), 1.74 – 1.58 (m, 1H, $CHCH\underline{H}$), 1.43 (s, 9H, NH<u>Boc)</u> 1.54 – 1.22 (m, 6H). ^{13}C NMR (126 MHz, $CDCl_3$) δ 177.5 (COOH), 155.8 (NHCO), 80.4 ($\underline{C}(CH_3)_3$), 53.4 (CH), 34.0 (CH_2), 32.7 (CH_2), 32.4 (CH_2), 28.5 ($C(\underline{C}H_3)_3$), 28.0 (CH_2), 25.2 (CH_2).

LCMS-ESI-MS. Positive Mode. Expected mass of sodium adduct $[C_{13}H_{24}{}^{79}BrNNaO_4]^+$ 360.0781, m/z of sample 360.0777. Expected mass of sodium adduct $[C_{13}H_{24}{}^{81}BrNNaO_4]^+$ 362.0760, m/z of sample 362.0755.

2-((tert-butoxycarbonyl)amino)-8-iodooctanoic acid- 1e

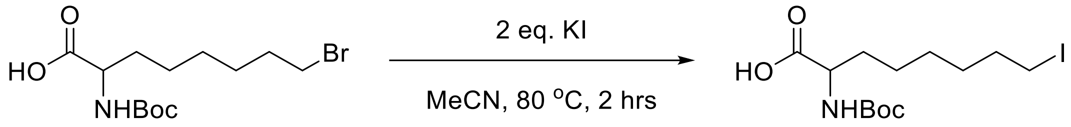

Solid 8-bromo-2-((tert-butoxycarbonyl)amino)octanoic acid (**1d**) (67.6 mg, 0.2 mmol) and potassium iodide (66.4 mg, 0.4 mmol) dissolved in dry MeCN (1 ml), and heated in the MW at 80 °C for 2 hours. Reaction mixture was then diluted with H_2O (1 ml), and product extracted with EtOAc (4 x 2 ml), dried over $MgSO_4$ and solvent removed *in vacuo* to give a yellow oil product (53.6 mg, 69.5%)

^{1}H NMR (400 MHz, $CDCl_3$) δ 9.35 (s, 1H, OH), 5.03 (d, J = 8.4 Hz, 1H, NH), 4.30 (q, J = 7.5 Hz, 1H, CH), 3.17 (t, J = 7.0 Hz, 2H, CH_2I), 1.80 (p, J = 7.0 Hz, 3H, $CH_2CH\underline{H}$), 1.71 – 1.56 (m, 1H, $CH_2C\underline{H}H$), 1.44 (s, 9H, $C(CH_3)_3$), 1.41 – 1.20 (m, 4H, CH_2CH_2). ^{13}C NMR (101 MHz, $CDCl_3$) δ 177.8 (COOH), 155.7 (COOtBu), 80.3 ($C(CH_3)$), 53.4 (CH), 33.4 (CH_2), 32.4 (CH_2), 30.3 (CH_2), 28.4 (CH_3), 28.2 (CH_2), 25.2 (CH_2), 7.1 (CH_2I)

LCMS-ESI-MS. Positive Mode. Expected mass of sodium adduct $[C_{13}H_{24}INNaO_4]^+$ 408.0642. m/z of sample 408.0641.

2-Amino-8-bromohexanoic acid TFA salt- 1a

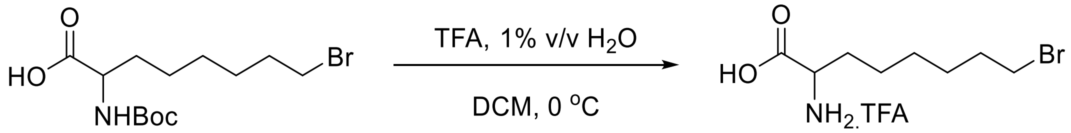

8-bromo-2-((tert-butoxycarbonyl)amino)octanoic acid (**1d**) (346 mg, 1.02 mmol) was dissolved in DCM (3 ml) and cooled to 0 °C. H_2O (1% v/v) was added, followed by the dropwise addition of TFA (12 ml) at 0 °C. Reaction was stirred at 0 °C for 15 minutes, then at room temperature until the reaction was complete. Solvent was then removed *in vacuo*, and the orange residue dissolved in DCM (5 ml), which was removed *in vacuo*. This was repeated three times. The residue was then triturated with cold Et_2O to give the product as a white solid (212 mg, 59%).

^{1}H NMR (300 MHz, MeOD) δ 3.94 (t, J = 6.3 Hz, 1H, CH), 3.45 (t, J=6.9 Hz, 2H, $C\underline{H_2}Br$), 1.88 (m, 4H), 1.48 (m, 6H). ^{13}C NMR (101 MHz, MeOD) δ 172.2 (C=O), 54.1 (CH), 34.24 (CH_2), 33.7 (CH_2), 31.5 (CH_2), 29.3 (CH_2), 28.8 (CH_2), 25.8 (CH_2).

LCMS-ESI-MS. Positive Mode. Expected mass of $[C_8H_{17}O_2N^{79}Br]^+$ 237.0437, m/z of sample 238.0433. Expected mass of $[C_8H_{17}O_2N^{81}Br]^+$ 240.0417, m/z of sample 238.0412 These data are consistent with reported data.[5]

FTIR (neat) cm^{-1}: 3008 (OH, br), 2932 (CH, m), 2855 (NH, m), 1732 (CO, s), 1668 (CO, s), 1466 (CH, m), 1414 (CH, m), 521 (CBr, m)

***N*-Boc-Tyr-OMe**

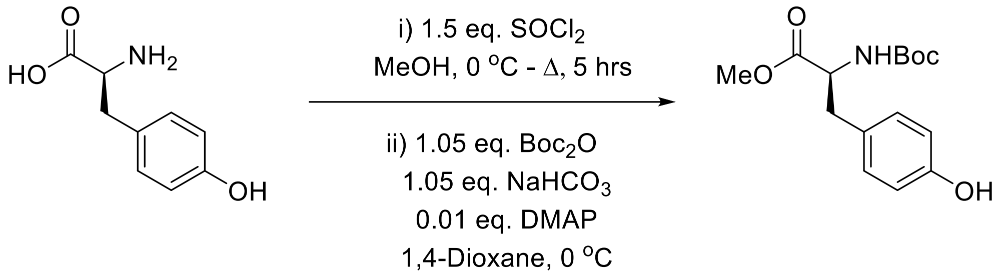

L-Tyrosine (1.087 g, 6 mmol) was dissolved in anhydrous MeOH (20 ml) and cooled to 0 °C. $SOCl_2$ (0.65 ml, 1.07 g, 9 mmol) added slowly, stirred at 0 °C for 10 minutes, followed by reflux at 75 °C for 5 hours. MeOH removed *in vacuo* to give a white solid (1.43 g). Solid was dissolved in dioxane (10 ml) and H_2O (5 ml). $NaHCO_3$ (529 mg, 6.3 mmol) added slowly at room temperature. Once all solid was dissolved, DMAP (7.3 mg, 0.06 mmol) was added, followed by Di-*tert*-butyl dicarbonate (1.37 g, 6.3 mmol) in dioxane (5 ml). Reaction mixture was stirred for 4 hours, monitored by TLC (Hexane: EtOAc, 1:1). Solvent was removed *in vacuo* and residue was suspended in EtOAc (20 ml). This EtOAc suspension was washed with dilute citric acid (25 ml), H_2O (25 ml), dilute $NaHCO_3$ (25 ml) and brine (25 ml). Organic layer was dried over sodium sulphate, filtered, and solvent removed *in vacuo* to give pure product as a yellow oil (1.847 g, 97%).

^{1}H NMR (400 MHz, $CDCl_3$) δ 6.97 (d, J = 8.1 Hz, 2H), 6.74 (d, J = 8.1 Hz, 2H), 4.99 (d, J = 8.4 Hz, 1H), 4.54 (q, J = 6.4 Hz, 1H), 3.71 (s, 3H), 3.00 (qd, J = 13.9, 5.9 Hz, 2H), 1.42 (s, 9H). ^{13}C NMR (126 MHz, CDCl) δ 172.7 (COOMe), 155.3 (C), 154.9 (COON), 130.6 (CH), 128.0 (C), 115.6 (CH), 80.2 (C), 54.7 (CH_3), 52.4 (CH), 37.7 (CH_2), 28.4 (CH_3). These data are consistent with the reported values.[7]

Methyl-(S)-2-amino-3-(4-(4-bromobutoxy)phenyl)propanoate TFA salt- 1g

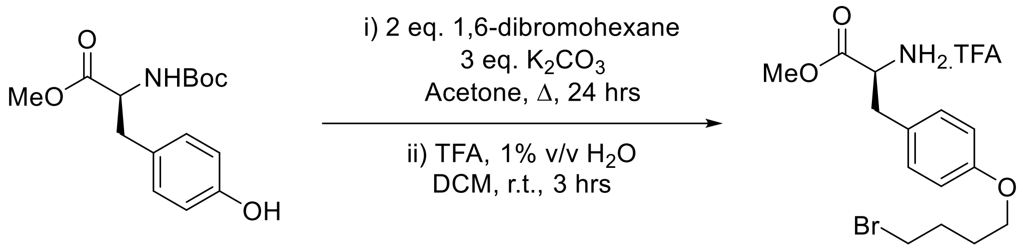

N-Boc-Tyr-OMe (1.85 g, 6 mmol) and potassium carbonate (2.49g, 18 mmol) dissolved in acetone (50 ml). 1,4-Dibromobutane (1.4 ml, 12 mmol) was added and refluxed for 24 hours. Reaction was cooled to room temperature and insoluble salts were filtered out and washed with EtOAc (10 ml x 3). Filtrate was concentrated on rotary evaporator to give crude white solid, which was purified by flash column chromatography using Hexane: EtOAc (gradient 0 – 20%) (20% EtOAc in Hexane, Rf = 0.2) to give the O-(4-bromobutyl) intermediate as a white solid. This solid then dissolved in DCM (3 ml) and cooled to 0 °C. TFA (15 ml) added slowly. The mixture was stirred at 0 °C for 2 hours, then at room temperature for 3 hours. Solvent was removed *in vacuo*, residue was left to stand and crystallise to as a brown crystalline solid (1.66 g, 62% over two steps)

^{1}H NMR (500 MHz, $CDCl_3$) δ 7.15 (d, J = 8.6 Hz, 2H, Ar-H), 6.92 (d, J = 8.7 Hz, 2H, Ar-H), 4.26 (dd, J = 7.5, 5.9 Hz, 1H, CH), 4.01 (t, J = 6.1 Hz, 2H, OCH_2), 3.82 (s, 3H, OCH_3), 3.52 (t, J = 6.6 Hz, 2H, CH_2Br), 3.19 (dd, J = 14.5, 5.9 Hz, 1H, C<u>H</u>H), 3.09 (dd, J = 14.5, 7.5 Hz, 1H, CHU), 2.04 (m, 2H, CH_2), 1.96 – 1.85 (m, 2H, CH_2). ^{13}C NMR (126 MHz, $CDCl_3$) δ 169.3 (COOMe), 159.0 (C), 130.4 (CH), 124.4 (C), 115.4 (CH), 67.0 (CH_2), 54.6 (CH), 53.6 (OCH_3), 35.3 (CH_2), 33.6 (CH_2), 29.5 (CH_2), 27.9 (CH_2)

LCMS-ESI-MS. Positive Mode. Expected mass of $[C_{14}H_{21}{}^{79}BrO_3N]^+$ 330.0699, m/z of sample 330.0691. Expected mass of $[C_{14}H_{21}{}^{81}BrO_3N]^+$ 331.2295, m/z of sample 331.0729.

$[\alpha]^{20}_{D}$ + 10.1 (c = 1, $CHCl_3$)

Stereochemical elucidation performed using FDAA (see page 3), with substrates L-tyrosine and **1g**. Two peaks from product of reaction between **1g** and FDAA indicates the formation of a two diastereomers. However LC-MS analysis indicates that these two peaks are due to hydrolysis of methyl ester under reaction conditions, and that only one diastereomer of **1g.FDAA** ($[C_{23}H_{28}{}^{79}BrN_5O_8]H^+$, m/z 582.1195) and **1g-OH.FDAA** ($[C_{22}H_{26}{}^{79}BrN_5O_8]H^+$, m/z 568.1038) are present.

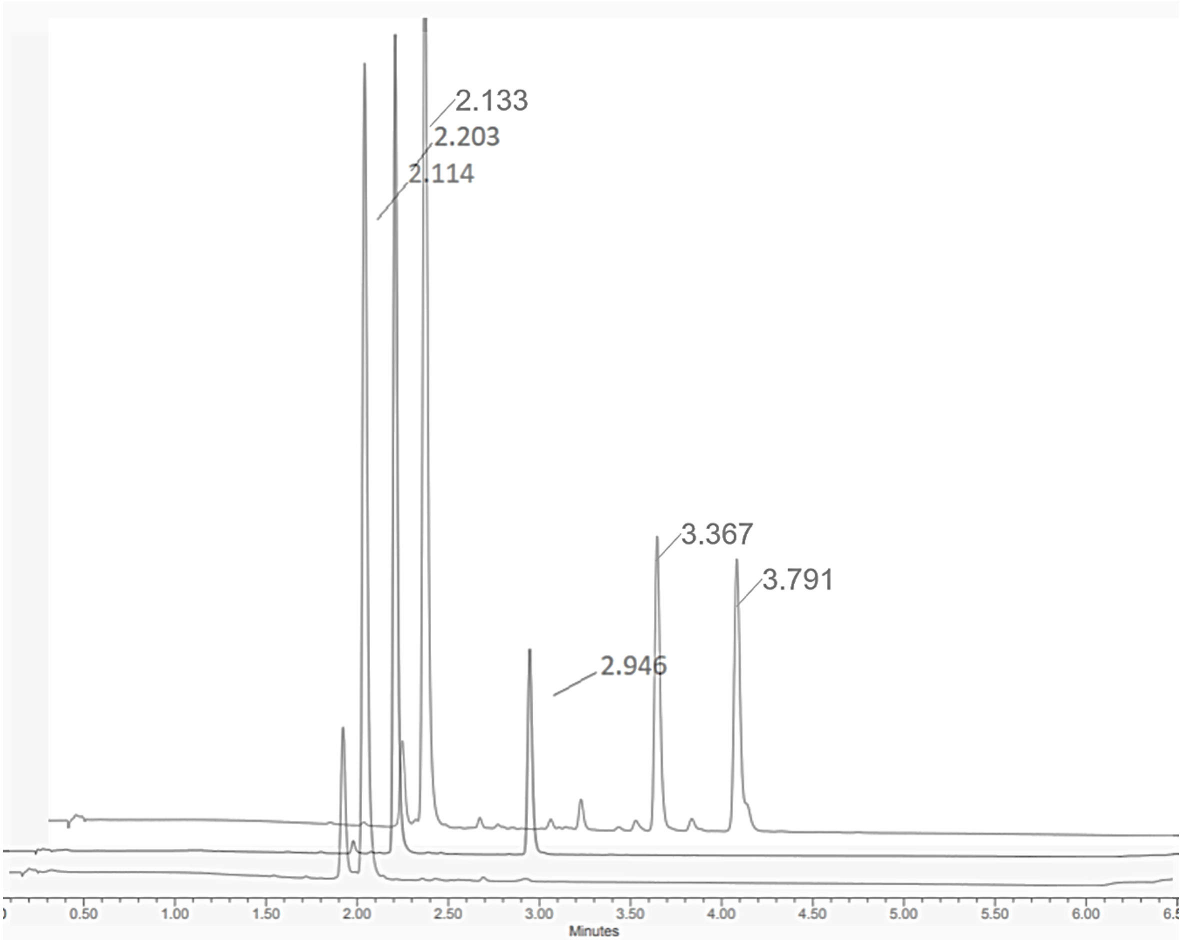

Supplementary Figure 6: Stereochemical elucidation of **1g** UPLC trace. Bottom line- control reaction containing no substrate. FDAA (subjected to reaction conditions) retention time 2.1 min. Middle line- L-Tyrosine.FDAA, retention time 2.9 min. Top line- **1g.FDAA,** retention time 3.8 min. **1g-OH.FDAA**, retention time 3.4 min.

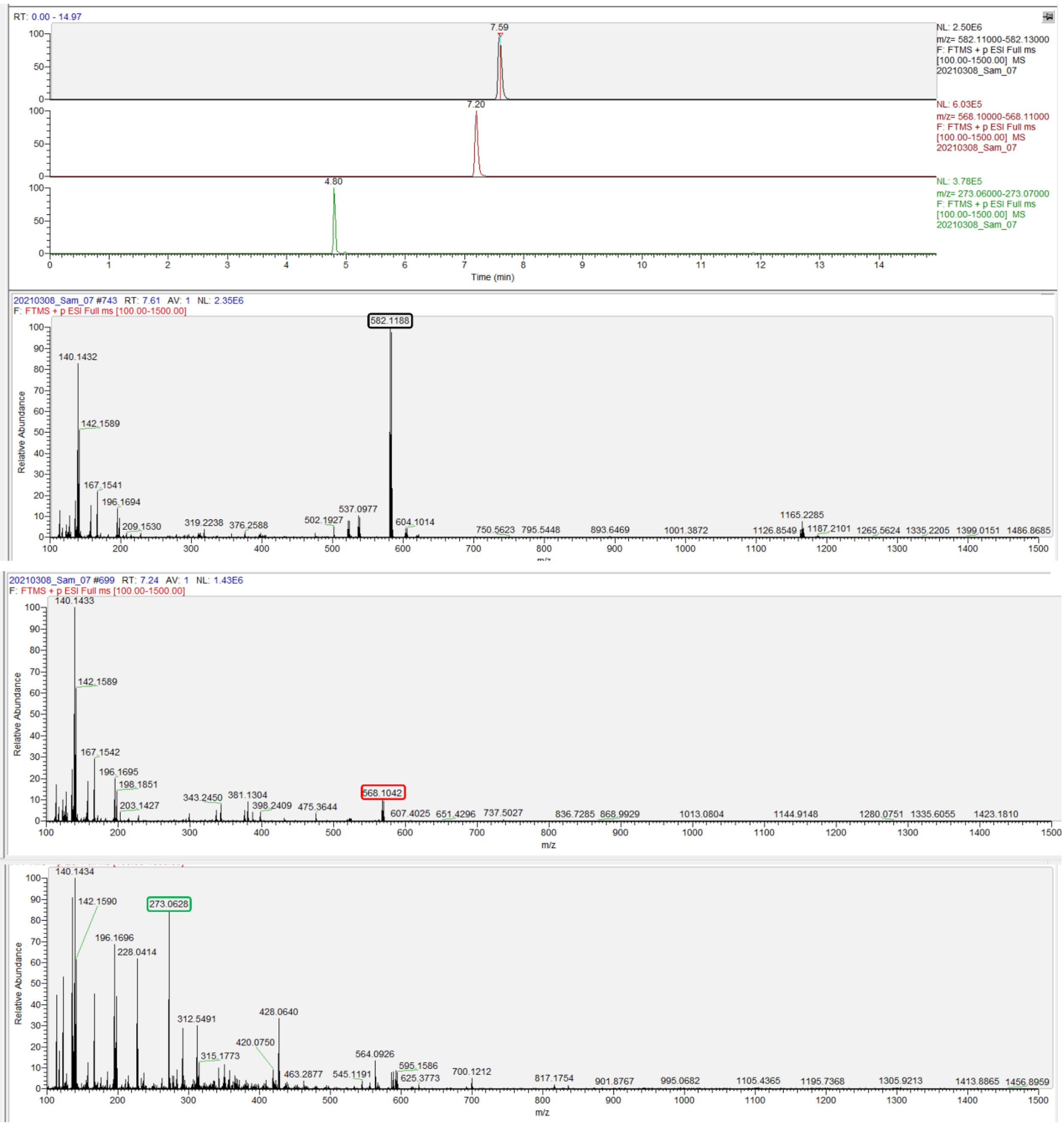

Supplementary Figure 7: LCMS trace of **1g.FDAA**. Green line- FDAA ($[C_9H_9FN_4O_5]H^+$, m/z 273.0630) retention time 4.80 min. Red line- **1g-OH.FDAA** ($[C_{22}H_{26}{}^{79}BrN_5O_8]H^+$, m/z 568.1038), retention time 7.20 min. Black line- **1g.FDAA** ($[C_{23}H_{28}{}^{79}BrN_5O_8]H^+$, m/z 582.1195), retention time 7.59 min.

2-amino-3-(4-(4-bromobutoxy)phenyl)propanoic acid hydrochloride- 1n

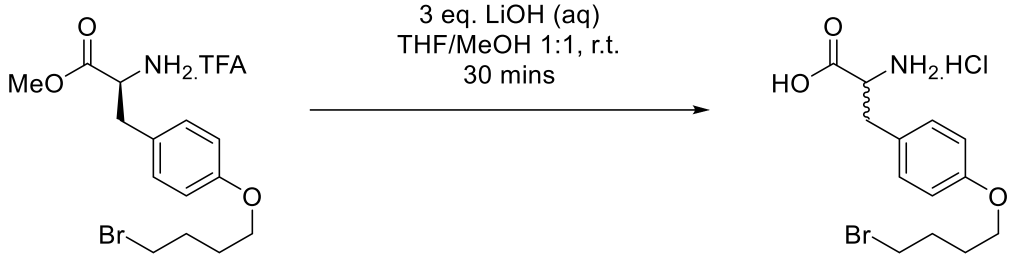

1g (1.7 mmol) was dissolved in THF/MeOH 1:1 (4 ml) at room temperature. Lithium hydroxide (214 mg, 5.1 mmol) in 2.5 mL of water was added to the solution. The reaction mixture was stirred for 30 min, reaction followed by TLC. The crude reaction mixture was dried *in vacuo* to give a white solid, which was redissolved in MeOH/H_2O 1:1 (25 ml) and acidified to pH 3 with HCl (1M). The resulting filtrate was collected, dried *in vacuo*, to give pure product (432 mg, 72%).

^{1}H NMR (500 MHz, MeOD) δ 7.20 (d, J = 8.6 Hz, 2H, Ar-H), 6.92 (d, J = 8.6 Hz, 2H, Ar-H), 4.19 (dd, J = 7.5, 5.3 Hz, 1H, CH), 4.01 (t, J = 6.1 Hz, 2H, OCH_2), 3.52 (t, J = 6.6 Hz, 2H, CH_2Br), 3.27 – 3.04 (m, 2H, Ar-C$\underline{H_2}$), 2.06 – 2.01 (m, 2H, CH_2) 1.96 – 1.89 (m, 2H, CH_2). ^{13}C NMR (126 MHz, MeOD) δ 171.3 (COOH), 160.1 (C), 131.6 (CH), 127.3 (C), 116.1 (CH), 68.1 (CH_2), 55.2 (CH), 36.5 (CH_2), 34.1 (CH_2), 30.7 (CH_2), 29.1 (CH_2).

LCMS-ESI-MS. Positive Mode. Expected mass of $[C_{13}H_{19}{}^{79}BrNO_3]^+$ 316.0543, m/z of sample 316.0544. Expected mass of $[C_{13}H_{19}{}^{79}BrNO_3]^+$ 318.0523, m/z of sample 318.0523

Stereochemical elucidation performed using FDAA (see page 3). Analysed using general LC-MS protocol. Upon reaction with FDAA, **1n** split into two peaks. Expected mass of **1n.FDAA** $[C_{22}H_{27}{}^{79}BrN_5O_8]^+$ 568.1038. m/z of peak retention time 7.21-7.24 min, 468.1043. m/z of peak retention time 7.26-7.31 min, 468.1044. (SI Figure 5)

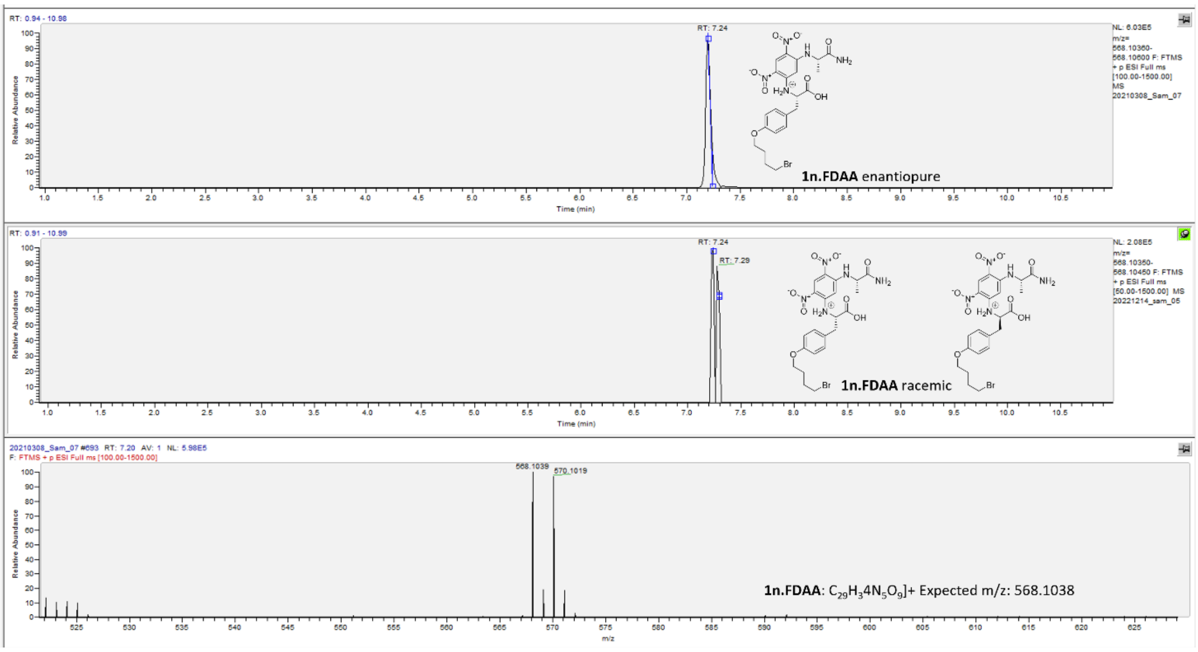

Supplementary Figure 8 Top: LC-MS trace of enantiopure **1n.FDAA**. Expected mass of **1n.FDAA** $[C_{22}H_{27}{}^{79}BrN_5O_8]^+$ 568.1038. m/z of peak retention time 7.24 min, 568.1039. Middle: LC-MS trace of racemic **1n.FDAA**. Expected mass of **1n.FDAA** $[C_{22}H_{27}{}^{79}BrN_5O_8]^+$ 568.1038. m/z of peak retention time 7.24 min, 568.1043, m/z of peak retention time 7.29 min, 568.1044. Bottom: m/z of **1n.FDAA**

7-(4-bromobutoxy)-2H-chromen-2-one- 1i

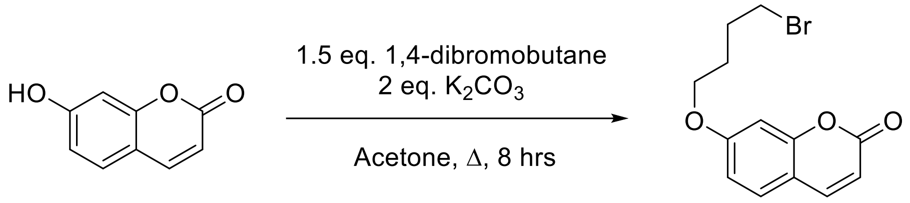

7-Hydroxycoumarin (303.2 mg, 1.5 mmol) and potassium carbonate (414.6 mg, 3 mmol) were dissolved in acetone (50 ml), and 1,4-dibromobutane (0.24 ml, 2 mmol) was added, followed by reflux for 8 hours. Reaction mixture was allowed to cool to room temperature, and insoluble salts filtered and washed with acetone (20 ml x 3), the organic solvent was then removed *in vacuo*. The crude mixture was purified by flash column chromatography using Hexane: EtOAc (gradient 0 – 20%) (20% EtOAc in Hexane, Rf = 0.27) to give a greyish white crystalline solid (354 mg, 81%)

^{1}H NMR (500 MHz, $CDCl_3$) δ 7.63 (dd, J = 9.5, 0.6 Hz, 1H, Ar-H), 7.37 (d, J = 8.5 Hz, 1H, Ar-H), 6.86 – 6.77 (m, 2H, Ar-H), 6.25 (d, J = 9.5 Hz, 1H, Ar-H), 4.05 (t, J = 6.0 Hz, 2H, CH_2O), 3.49 (t, J = 6.5 Hz, 2H, CH_2Br), 2.13 – 2.04 (m, 2H, CH_2), 2.04 – 1.94 (m, 2H, CH_2). ^{13}C NMR (126 MHz, $CDCl_3$) δ 162.2 (C), 161.4 (C), 156.0 (C), 143.5 (CH), 128.9 (CH), 113.3 (CH), 113.0 (CH), 112.7 (C), 101.5 (CH), 67.6 (CH_2), 33.3 (CH_2), 29.4 (CH_2), 27.8 (CH_2).

These data are consistent with the reported values [8]

Methyl (2-amino-8-bromooctanoyl)-L-phenylalaninate- 1m

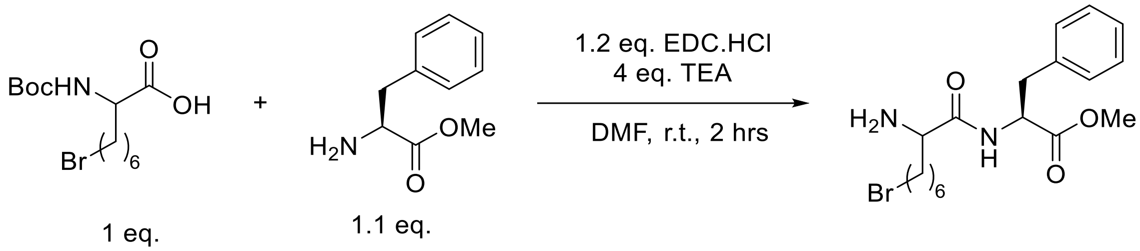

8-bromo-2-((tert-butoxycarbonyl)amino)octanoic acid (**1d**) (0.5 mmol. 169 mg), H-Phe-OMe-HCl (0.55 mmol, 118.6 mg) and EDC.HCl (0.6 mmol, 115 mg) dissolved in DMF (5 ml) and cooled to 0 °C. TEA (2 mmol, 0.28 ml) added, and reaction stirred at room temperature for 2 hours. Reaction mixture diluted with EtOAc (10 ml) and washed with dilute citric acid (10 ml x 2), H_2O (10 ml x 2), dilute $NaHCO_3$ (10 ml x 2) and brine (10 ml x 2). Organic layer was dried over $MgSO_4$, filtered, and concentrated to give white solid. Solid residue was dissolved in DCM (0.5 ml), TFA (3 ml) was added and the mixture was stirred for 3 hours at room temperature. Solvent was removed *in vacuo* to give yellow oil mixture of diastereomers (170 mg, 66%)

Based on ^{1}H NMR, two distinct methoxy peaks at δ 3.72 and 3.71 there are two diastereomeric forms present due to enantiomeric nature of starting material **1d**, separation was attempted but unsuccessful. Majority of peaks in ^{1}H NMR and ^{13}C NMR are overlapping, however distinct peaks are labelled as (dia).

^{1}H NMR (400 MHz ,$CDCl_3$) δ 7.33 – 7.21 (m, 3H, Ar-H), 7.16-7.09 (m, 2H, Ar-H), 4.98 – 4.77 (m, 1H, CH), 3.72 and 3.71 (s, 3H, OMe, dia) 3.41-3.37 (m, 2H, CH_2Br), 3.37-3.31 (m, 1H, CH) 3.28 – 3.00 (m, 2H), 1.87-1.72 (m, 4H), 1.61 – 1.11 (m, 6H). ^{13}C NMR (101 MHz, $CDCl_3$) δ 172.3 (COO), 171.8 (CON), 136.2 (C), 129.4 (CH), 128.6 (CH), 127.2 (CH), 55.1 (CH), 52.8 (CH), 52.4 (CH_3), 45.2 (CH_2), 38.3 (CH_2 dia), 38.1 (CH_2 dia), 34.0 (CH_2), 32.8 (CH_2), 28.7 (CH_2), 28.1 (CH_2), 25.5 (CH_2).

LCMS-ESI-MS. Positive Mode. Expected mass of $[C_{18}H_{28}{}^{79}BrN_2O_3]^+$ 399.1278, m/z of sample 399.1276. Expected mass of $[C_{18}H_{28}{}^{81}BrN_2O_3]^+$ 401.1257, m/z of sample 401.1255.

1-(2-bromoethyl)indoline-2,3-dione- 1j

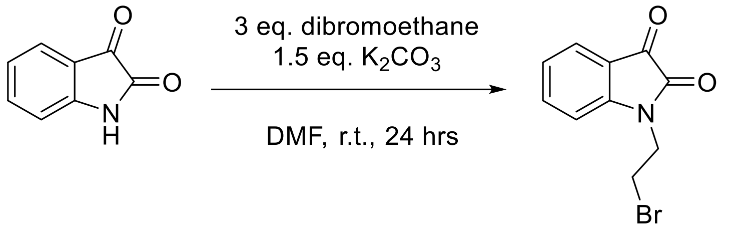

Isatin (1 g, 6.8 mmol) was dissolved in DMF (5 ml), and K_2CO_3 (1.41 g, 10.2 mmol) was added with stirring. Dibromoethane (5 ml, 58 mmol) was added dropwise and the mixture was stirred at room temperature for 24 hours. The reaction mixture was then filtered, and inorganic salts washed with DMF (3 x 2 ml). Filtrate was concentrated under reduced pressure to give a crude red solid product, which was recrystallised from hot EtOH to give pure red crystalline solid (684 mg, 40%)

^{1}H NMR (500 MHz, $CDCl_3$) δ 7.67 – 7.58 (m, 2H), 7.18 – 7.11 (m, 1H), 7.00 (d, J = 7.9 Hz, 1H), 4.15 (t, J = 6.8 Hz, 2H), 3.62 (t, J = 6.7 Hz, 2H). ^{13}C NMR (126 MHz, $CDCl_3$) δ 183.9 (CO), 182.8 (CO), 158.4 (C), 150.6 (C), 138.6 (CH), 125.9 (CH), 124.2 (CH), 110.4 (CH), 42.1 (CH_2), 27.2 (CH_2). These data are consistent with the reported values.[9]

N-(2-ethyl-6-methylphenyl)-2-iodo-N-(1-methoxypropan-2-yl)acetamide- 1k

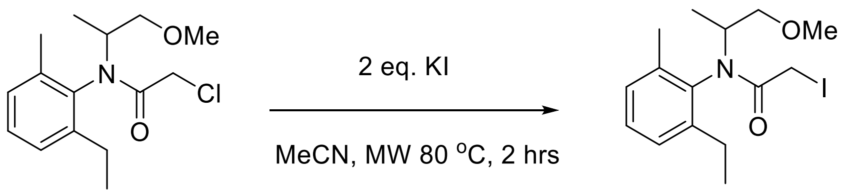

Metalachlor (0.15 mmol, 38 µl) and potassium iodide (0.30 mmol, 49.8 mg) were dissolved in MeCN (0.7 ml) and heated in the MW at 80 °C for 2 hours. The reaction mixture was then diluted with H_2O (2ml), extracted with EtOAc (4 x 3 ml), and the combined organic layers washed with H_2O (2 ml), brine (2 ml), dried over $MgSO_4$, and concentrated *in vacuo* to give a colourless oil (46.2 mg, 0.123 mmol, 82%).

Native metalachlor exists as four stable stereoisomers in solution at ambient conditions. aSS, aRS, aSR, and aRR.[3] Enantiomers due to chiral centre, and atropisomers due to limited rotation around the Ar-N bond- leading to separation of some peaks in NMR experiments of the aSS/aRR enantiomers (major, approximately 65%) and the aSR/aRS enantiomers (minor, approximately 35%).[4] Based on similar separation in **2j** peaks we can assume similar proportions of stereoisomers in solution. Where peaks are overlapping integrations are as expected, when peaks are separate their combined integrations are as expected.

^{1}H NMR (500 MHz, $CDCl_3$) δ 7.29 – 7.24 (m, 1H, Ar-H, overlapping isomers), 7.23 (dt, J = 7.9, 2.3 Hz, 1H, Ar-H, overlapping isomers), 7.14 (dt, J = 7.3, 2.6 Hz, 1H, Ar-H, overlapping isomers), 4.16 (qd, J = 6.9, 4.3 Hz, 1H, CH, overlapping isomers), 3.75 (dd, J = 9.4, 4.2 Hz, 1H, $C\underline{H}H$ major isomer), 3.72 (ddd, J = 26.3, 9.5, 4.3 Hz, 1H, $C\underline{H}H$ minor isomer), 3.51 (ddd, J = 9.4, 6.8, 5.5 Hz, 1H, $CH\underline{H}$, overlapping isomers), 3.48 – 3.41 (m, 2H, CH_2I overlapping isomers), 3.30 (s, 3H, OCH_3 major isomer) 3.28 (s, 3H, OCH_3 minor isomer), 2.70 – 2.57 (m, 2H, Ar-$C\underline{H_2}CH_3$ overlapping isomers), 2.33 (s, 3H, Ar-CH_3 minor isomer), 2.30 (s, 3H, Ar-CH_3 major isomer) 1.31-1.25 (m, 3H, $CHC\underline{H_3}$ overlapping isomers), 1.19-1.15 (m, 3H, Ar-$CH_2C\underline{H_3}$ overlapping isomers). ^{13}C NMR (126 MHz, $CDCl_3$) δ 168.5 (CON), 142.5 (Ar-N minor isomer), 142.4 (Ar-N major isomer), 138.7 (C, overlapping isomers), 136.9 (C, major isomer), 136.7 (C minor isomer), 129.2 (Ar-H minor isomer), 129.1 (Ar-H, major isomer), 128.8 (Ar-H, overlapping isomers), 127.0 (Ar-H, major isomer), 126.9 (Ar-H minor isomer), 74.7 ($\underline{C}H_2OCH_3$, overlapping isomers), 58.7 ($CH_2O\underline{C}H_3$, overlapping isomers), 55.8 (CH minor isomer), 55.6 (CH major isomer), 24.1 (Ar-$\underline{C}H_2CH_3$ minor isomer), 23.9 (Ar-$\underline{C}H_2CH_3$ major isomer), 19.4 (Ar-CH_3 overlapping isomers), 15.7 ($CH\underline{C}H_3$ minor isomer), 15.5 ($CH\underline{C}H_3$ major isomer), 14.4 (Ar-$CH_2\underline{C}H_3$ major isomer), 14.2 (Ar-$CH_2\underline{C}H_3$ minor isomer), -0.2 (CH_2I minor isomer), -0.3 (CH_2I major isomer)

LCMS-ESI-MS. Positive Mode. Expected mass of $[C_{15}H_{23}INO_3]^+$ 376.0768, m/z of sample 377.0768.

2-amino-8-hydroxyoctanoic acid- 1a-OH

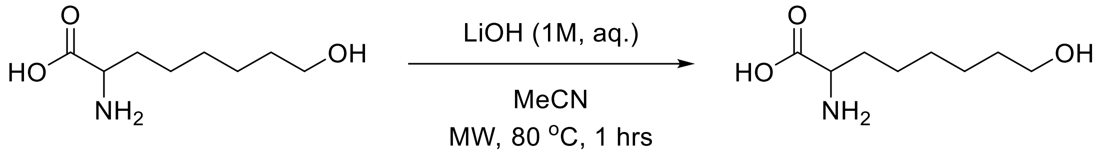

8-bromo-2-((tert-butoxycarbonyl)amino)octanoic acid (**1d**) (0.2 mmol, 67.6 mg) was dissolved in MeCN (1 ml), and LiOH (1M, 1 ml) added. Reaction mixture heated at 80 °C in MW for 1 hour. Reaction mixture was allowed to cool and MeCN removed *in vacuo*. Aqueous mixture was acidified to pH 4 using HCl (1M), and crude product extracted with EtOAc (3 x 5ml), dried over $MgSO_4$, and concentrated *in vacuo* to give a colourless oil. The oil was dissolved in DCM (1 ml) and cooled to 0 °C. TFA (2 ml) added slowly. The mixture was stirred at 0 °C for 2 hours, then at room temperature for 3 hours. Solvent was removed *in vacuo*, and purified by reverse-phase column chromatography on Biotage Isolera Four, using H_2O (1% v/v TFA): MeOH (gradient 0 – 100%) to give pure product. (45.6 mg, 79%)

^{1}H NMR (500 MHz, MeOD) δ 3.93 (t, J = 6.3 Hz, 1H, CH), 3.55 (t, J = 6.5 Hz, 2H, CH_2OH), 2.00 – 1.81 (m, 3H, $C\underline{H_2}C\underline{H}H$), 1.81-1.68 (m, 1H, $CH\underline{H}$), 1.58-1.50 (m, 2H, CH_2), 1.48 – 1.33 (m, 4H, CH_2CH_2). ^{13}C NMR (126 MHz, MeOD) δ 172.1 (COOH), 62.8 (CH_2OH), 54.0 (CH), 33.4 (CH_2), 31.5 (CH_2), 30.0 (CH_2), 26.6 (CH_2), 25.7 (CH_2).

LCMS-ESI-MS. Positive Mode. Expected mass of $[C_8H_{18}NO_3]^+$ 176.1281, m/z of sample 176.1283.

4. References

(1) Bhushan, R.; Brückner, H. Marfey's Reagent for Chiral Amino Acid Analysis: A Review. *Amino Acids* **2004**, *27* (3–4), 231–247. https://doi.org/10.1007/s00726-004-0118-0.

(2) Li, Y.; Chen, Q.; Yang, L.; Li, Y.; Zhang, Y.; Qiu, Y.; Ren, J.; Lu, C. Identification of Highly Potent N-Acylethanolamine Acid Amidase (NAAA) Inhibitors: Optimization of the Terminal Phenyl Moiety of Oxazolidone Derivatives. *Eur. J. Med. Chem.* **2017**, *139*, 214–221. https://doi.org/10.1016/j.ejmech.2017.08.004.

(3) Müller, M. D.; Poiger, T.; Buser, H. R. Isolation and Identification of the Metolachlor Stereoisomers Using High-Performance Liquid Chromatography, Polarimetric Measurements, and Enantioselective Gas Chromatography. *J. Agric. Food Chem.* **2001**, *49* (1), 42–49. https://doi.org/10.1021/jf000857f.

(4) Morton, M. D.; Walters, F. H.; Aga, D. S.; Thurman, E. M.; Larive, C. K. Nuclear Magnetic Resonance Identification of New Sulfonic Acid Metabolites of Chloroacetanilide Herbicides. **1997**, *8561* (96), 1240–1243.

(5) Liu, Y. E.; Lu, Z.; Li, B.; Tian, J.; Liu, F.; Zhao, J.; Hou, C.; Li, Y.; Niu, L.; Zhao, B. Enzyme-Inspired Axially Chiral Pyridoxamines Armed with a Cooperative Lateral Amine Chain for Enantioselective Biomimetic Transamination. *J. Am. Chem. Soc.* **2016**, *138* (34), 10730–10733. https://doi.org/10.1021/jacs.6b03930.

(6) Prakash, S.; Hazari, P. P.; Meena, V. K.; Jaswal, A.; Khurana, H.; Kukreti, S.; Mishra, A. K. Biotinidase Resistant 68Gallium-Radioligand Based on Biotin/Avidin Interaction for Pretargeting: Synthesis and Preclinical Evaluation. *Bioconjug. Chem.* **2016**, *27* (11), 2780–2790. https://doi.org/10.1021/acs.bioconjchem.6b00576.

(7) Bulman Page, P. C.; Buckley, B. R.; Farah, M. M.; John Blacker, A. Binaphthalene-Derived Iminium Salt Catalysts for Highly Enantioselective Asymmetrie Epoxidation. *European J. Org. Chem.* **2009**, No. 20, 3413–3426. https://doi.org/10.1002/ejoc.200900252.

(8) Xie, S. S.; Wang, X.; Jiang, N.; Yu, W.; Wang, K. D. G.; Lan, J. S.; Li, Z. R.; Kong, L. Y. Multi-Target Tacrine-Coumarin Hybrids: Cholinesterase and Monoamine Oxidase B Inhibition Properties against Alzheimer's Disease. *Eur. J. Med. Chem.* **2015**, *95*, 153–165. https://doi.org/10.1016/j.ejmech.2015.03.040.

(9) Satish, G.; Polu, A.; Ramar, T.; Ilangovan, A. Iodine-Mediated C-H Functionalization of Sp, Sp2, and Sp3 Carbon: A Unified Multisubstrate Domino Approach for Isatin Synthesis. *J. Org.*

Chem. **2015**, *80* (10), 5167–5175. https://doi.org/10.1021/acs.joc.5b00581.

5. NMR Spectra

2-amino-8-(4-methoxyphenyl)octanoic acid-3a

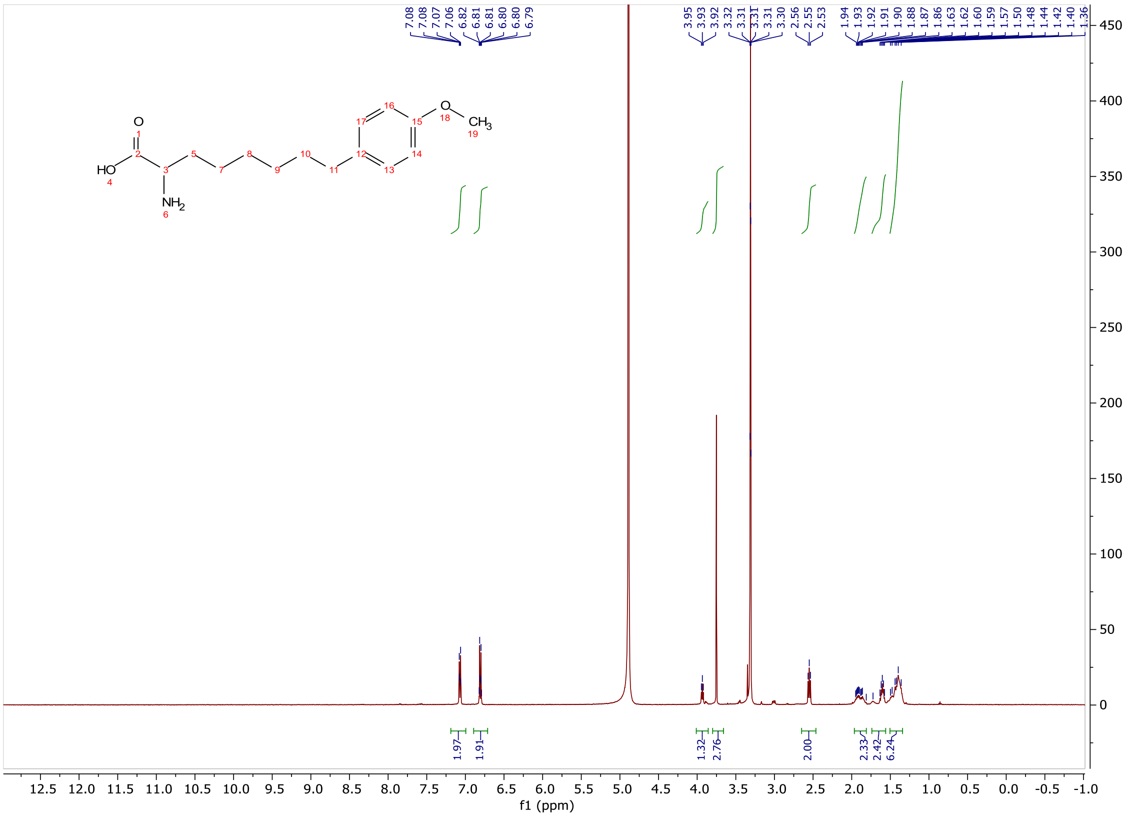

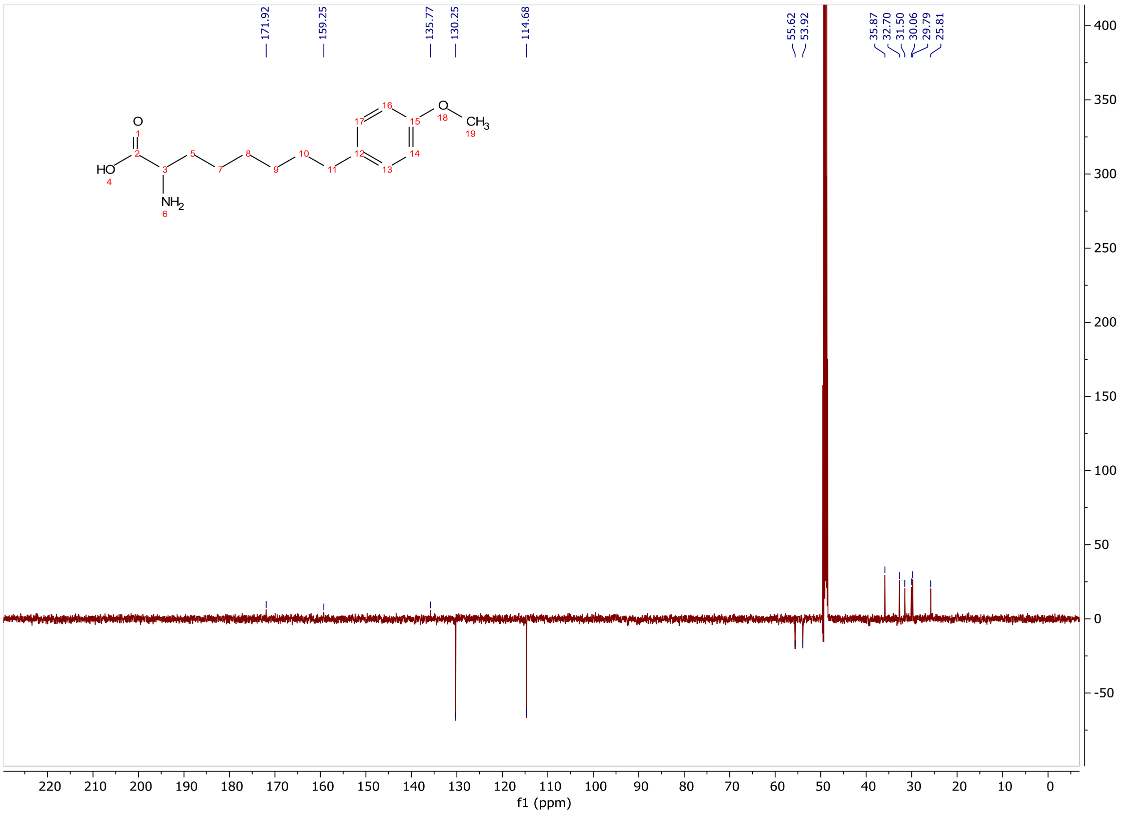

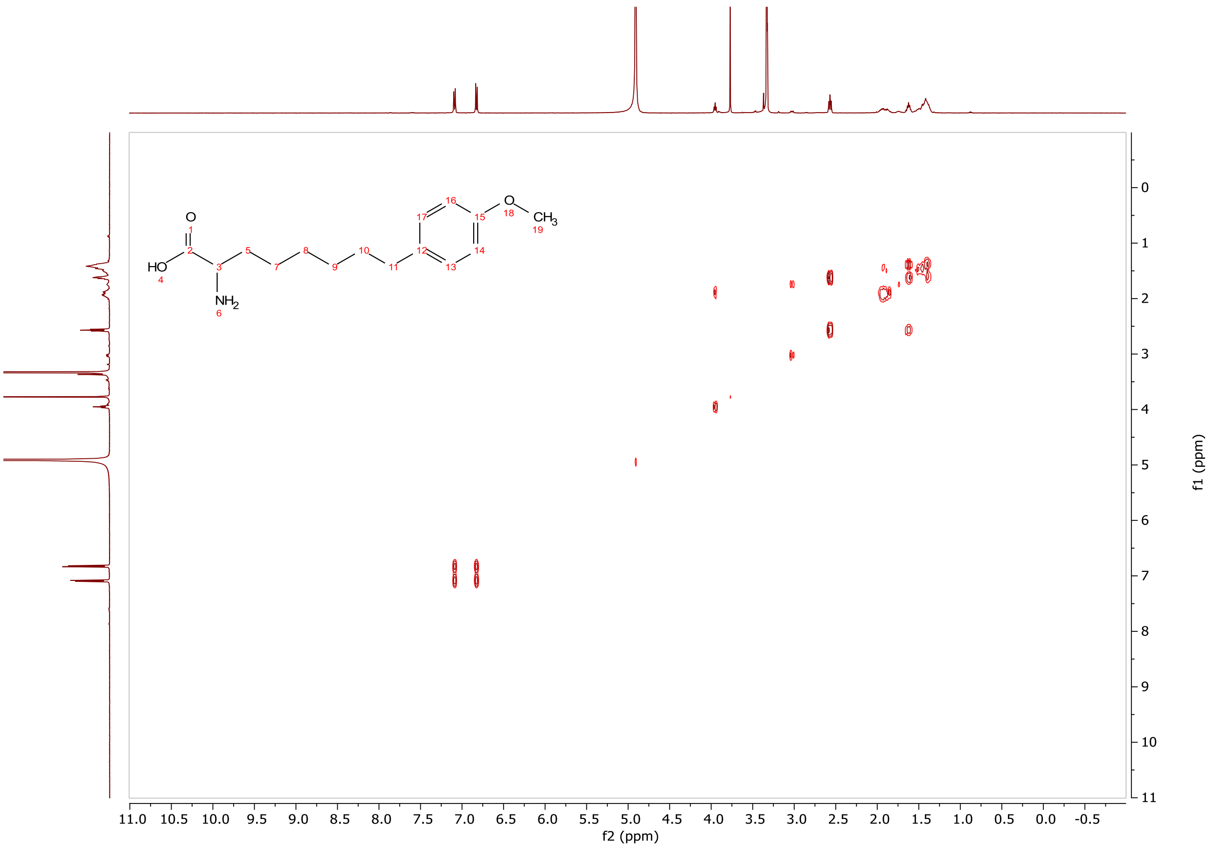
O
1
2
HO
4
3
NH2
6
5
7
8
9
10
11
12
13
14
15
16
17
O
18
CH3
19
f2 (ppm)
f1 (ppm)

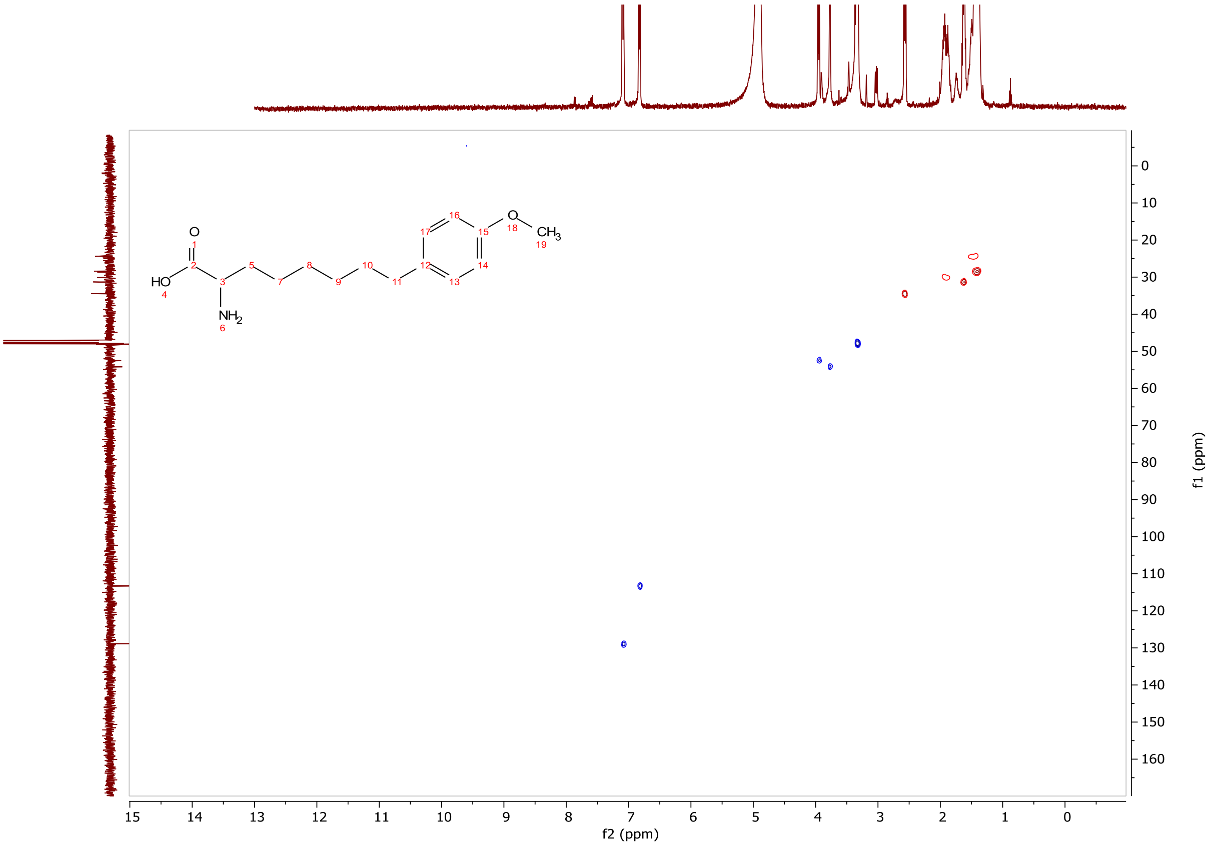
O
1
2
HO
4
3
NH2
6
5
7
8
9
10
11
12
13
14
15
16
17
O
18
CH3
19
f2 (ppm)
f1 (ppm)

±*N*-Acetyl-2-amino-8-(*p*-methoxyphenyl)-octanoic acid- 3b

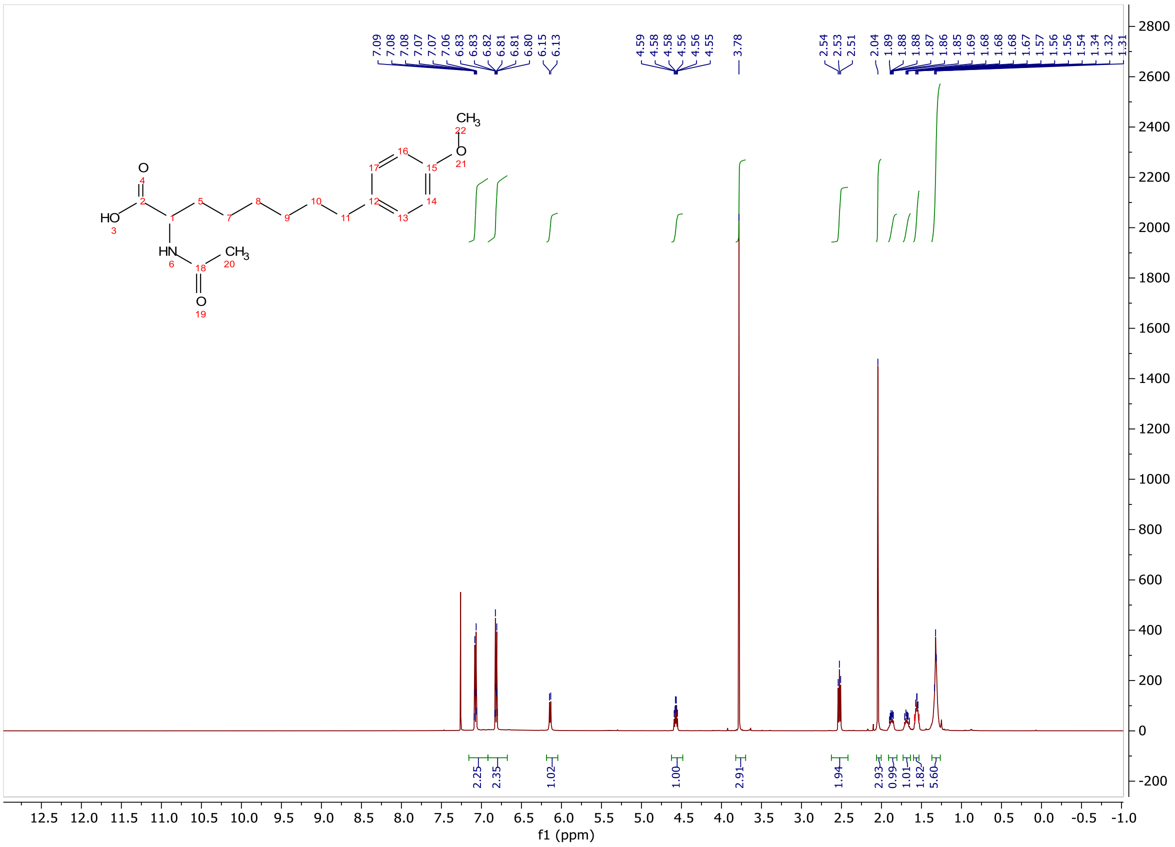

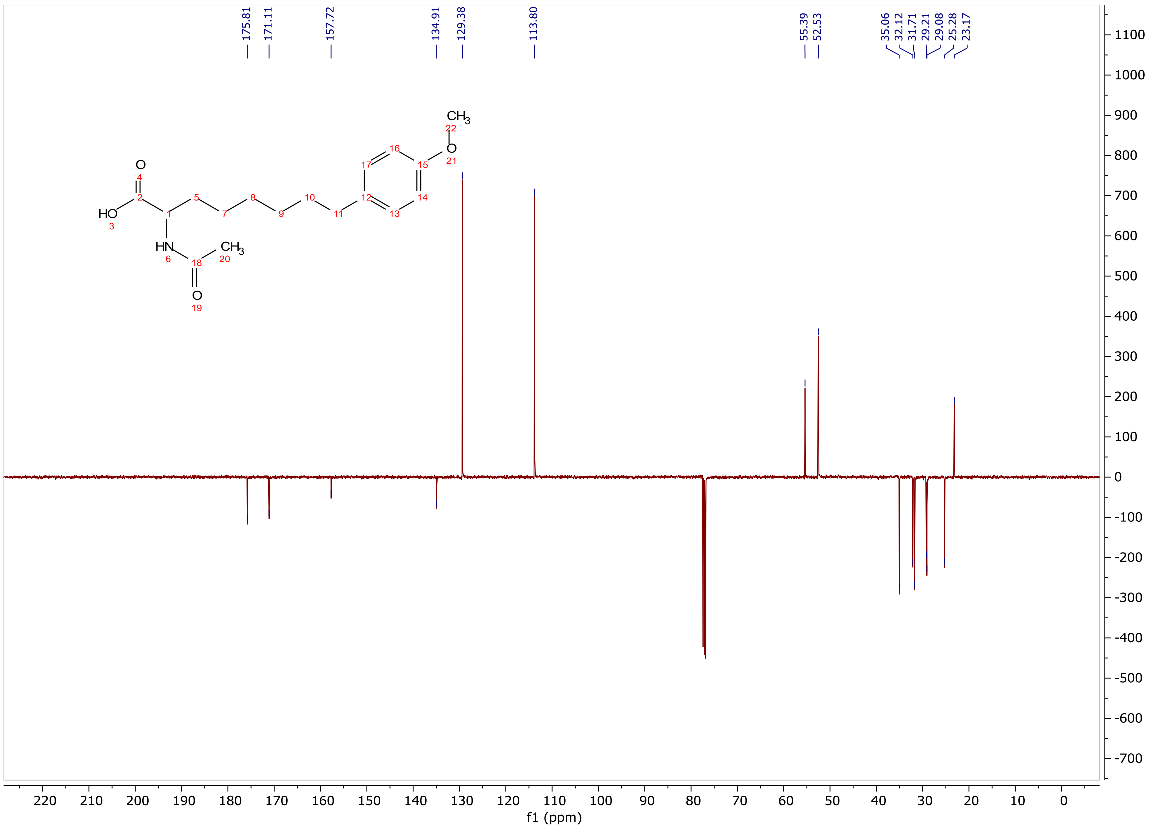

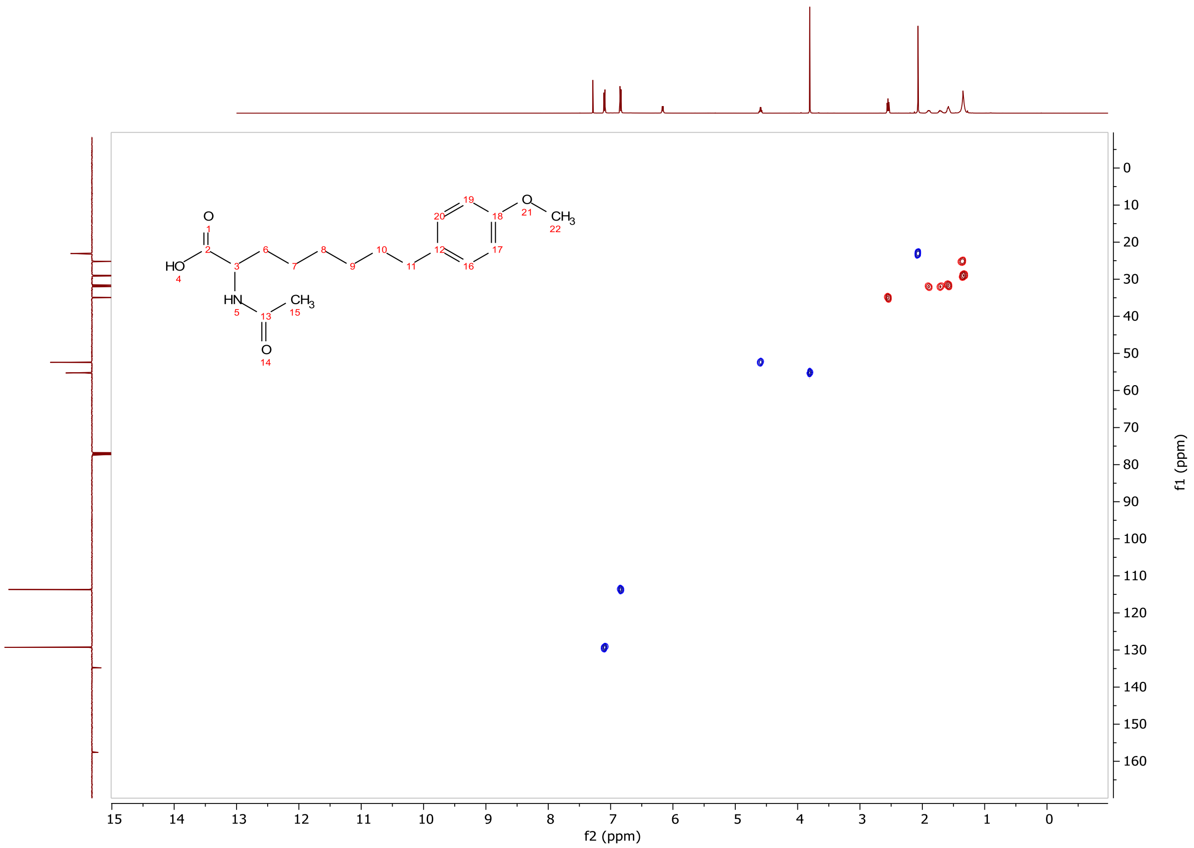

HO
HN
CH3
O
CH3
f2 (ppm)
f1 (ppm)

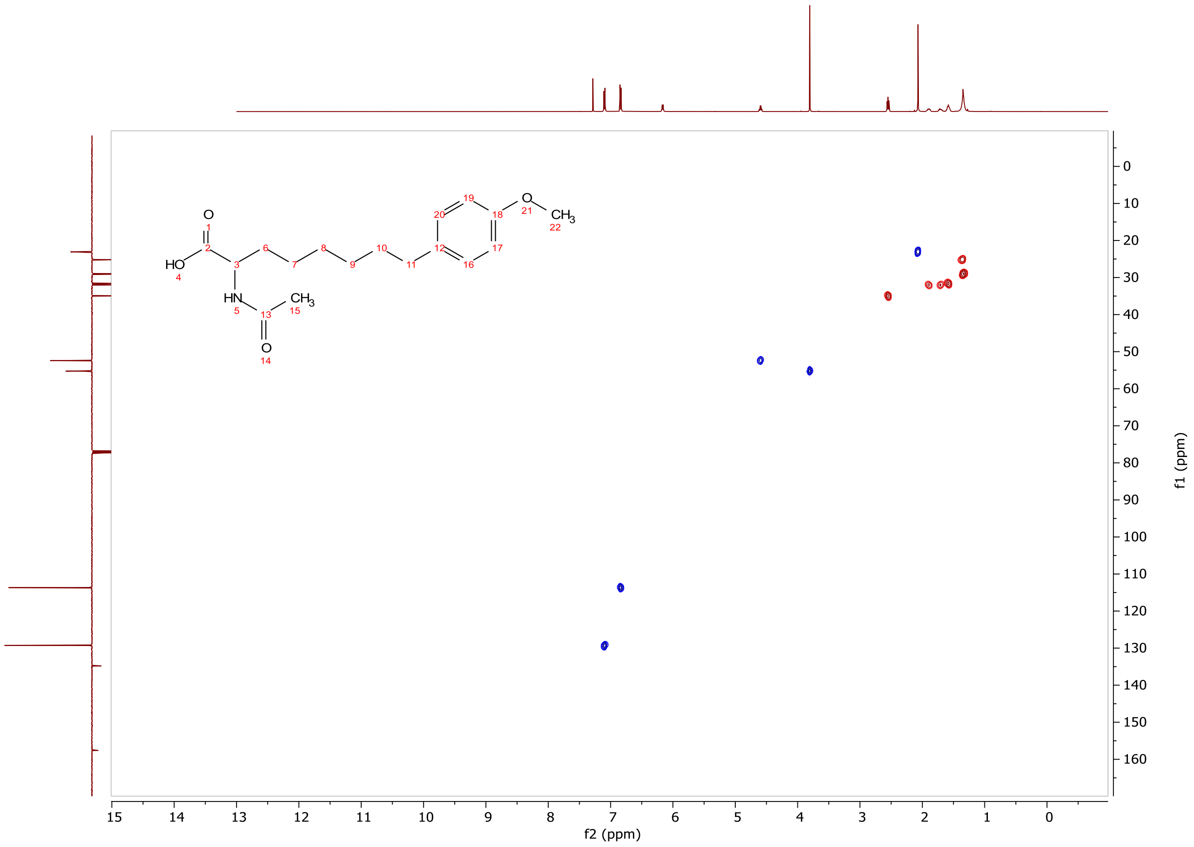

HO
HN
CH3
O
CH3
f2 (ppm)
f1 (ppm)

(4-methoxyphenyl)heptanoic acid-3c

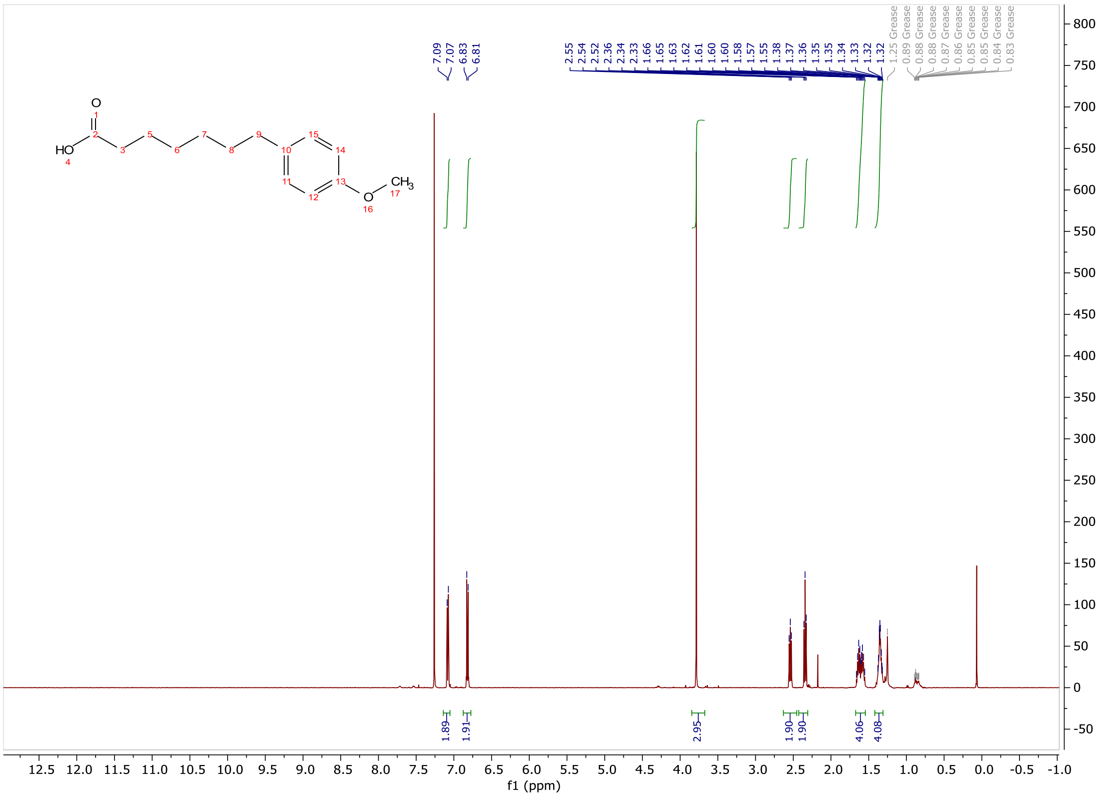

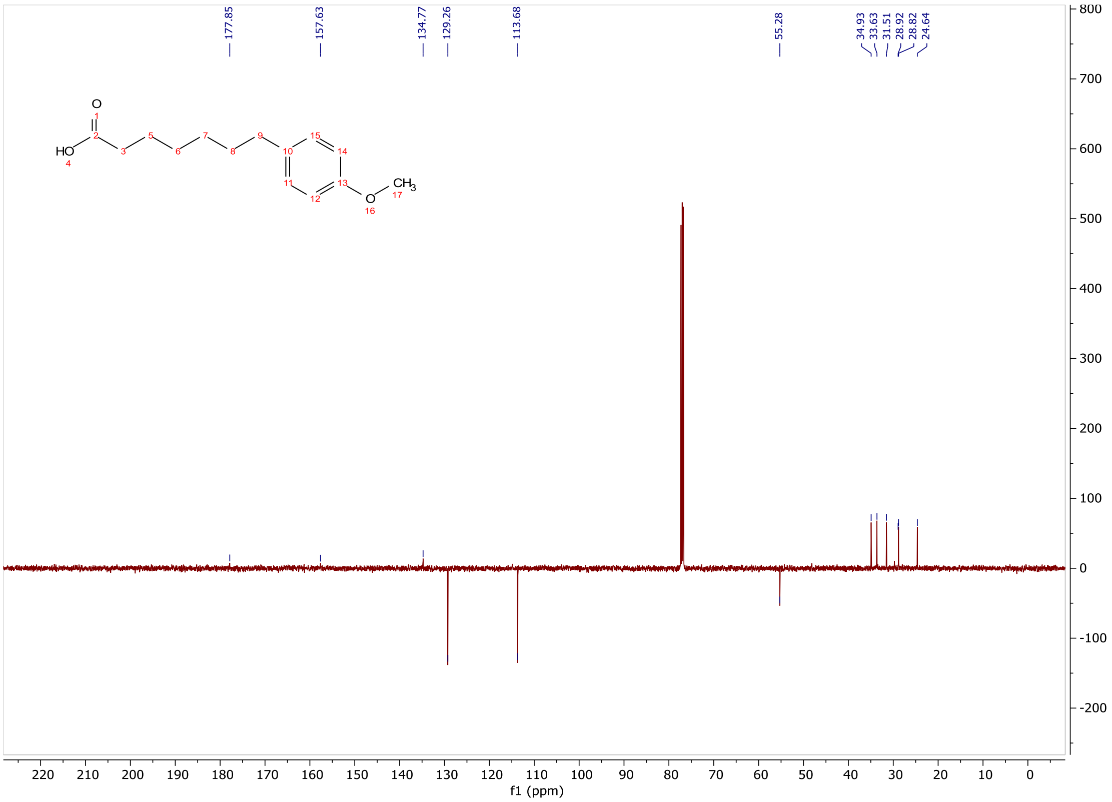

(S)-2-amino-3-(4-(4-(4-methoxyphenyl)butoxy)phenyl)propanoic acid- 3g

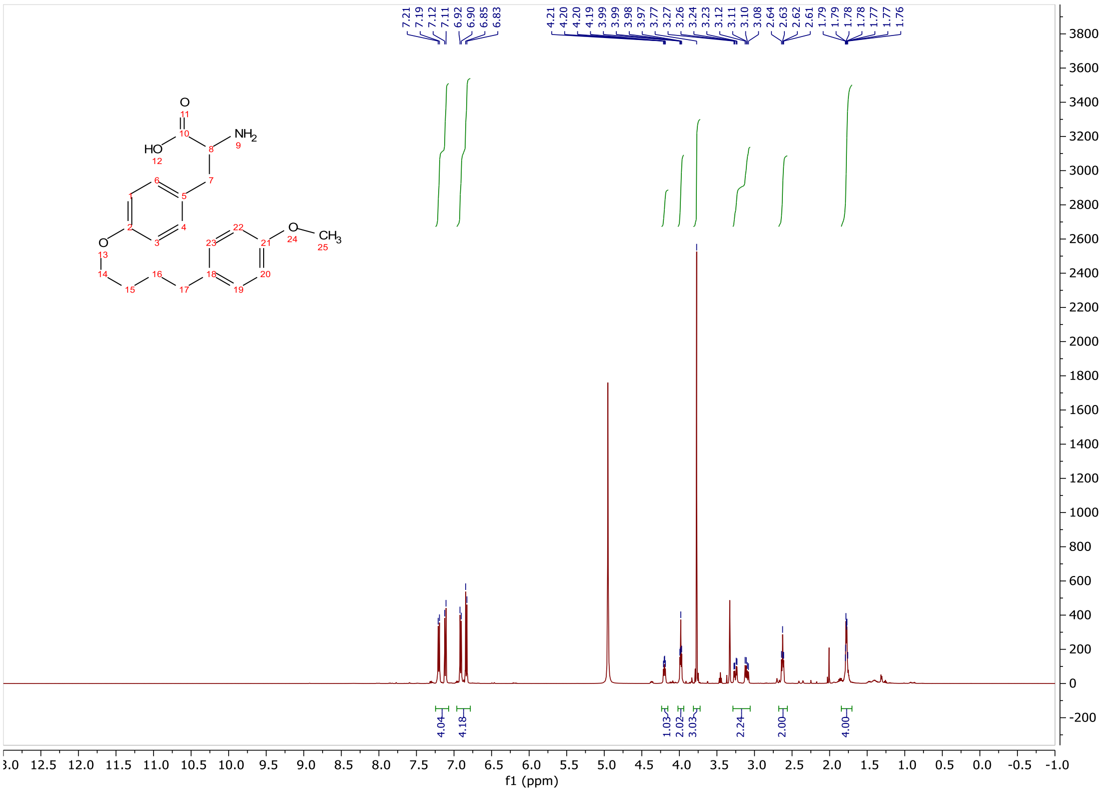

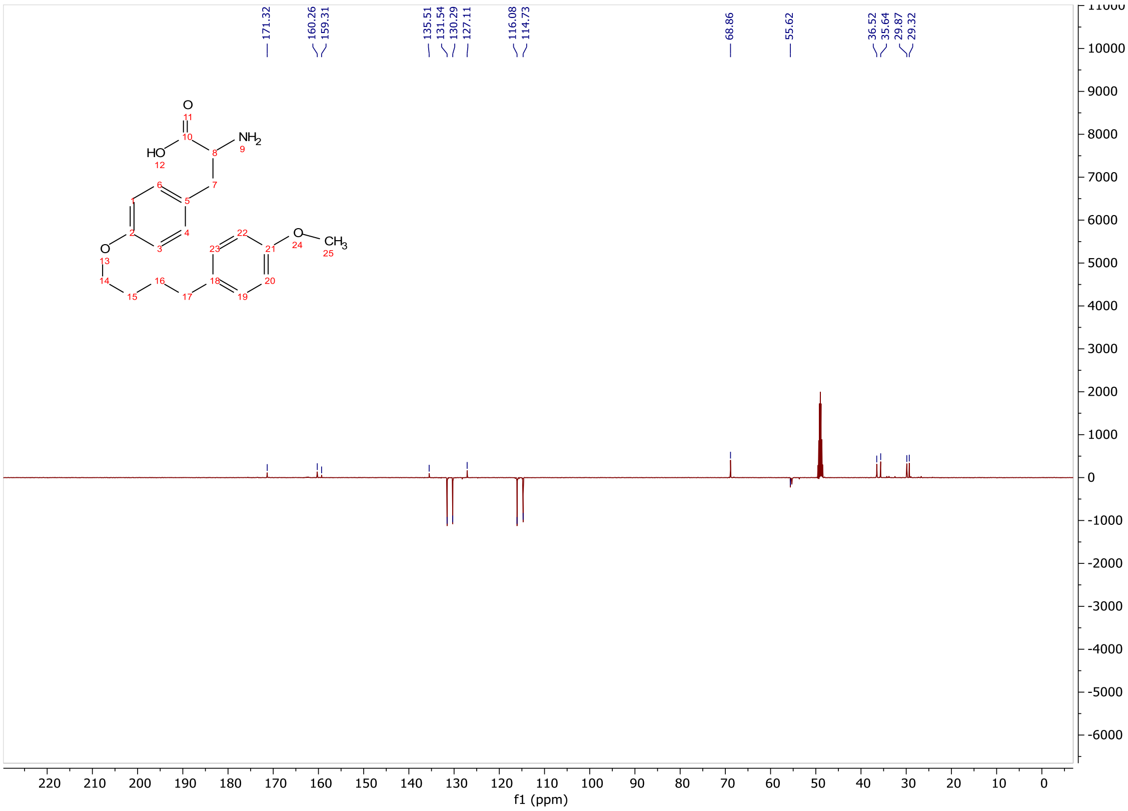

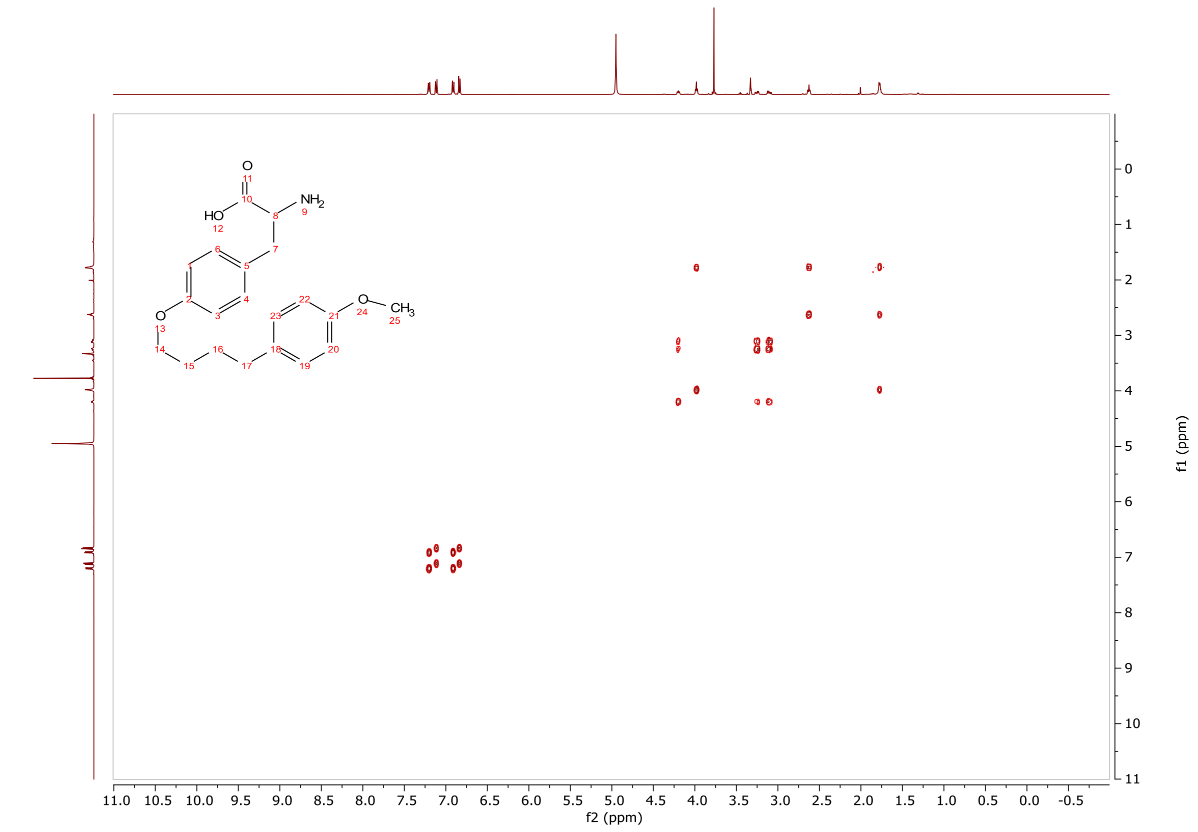

O
11
10
HO
12
8
NH2
9
7
6
5
4
3
2
1
O
13
14
15
16
17
18
19
20
21
22
23
O
24
CH3
25
f1 (ppm)
f2 (ppm)

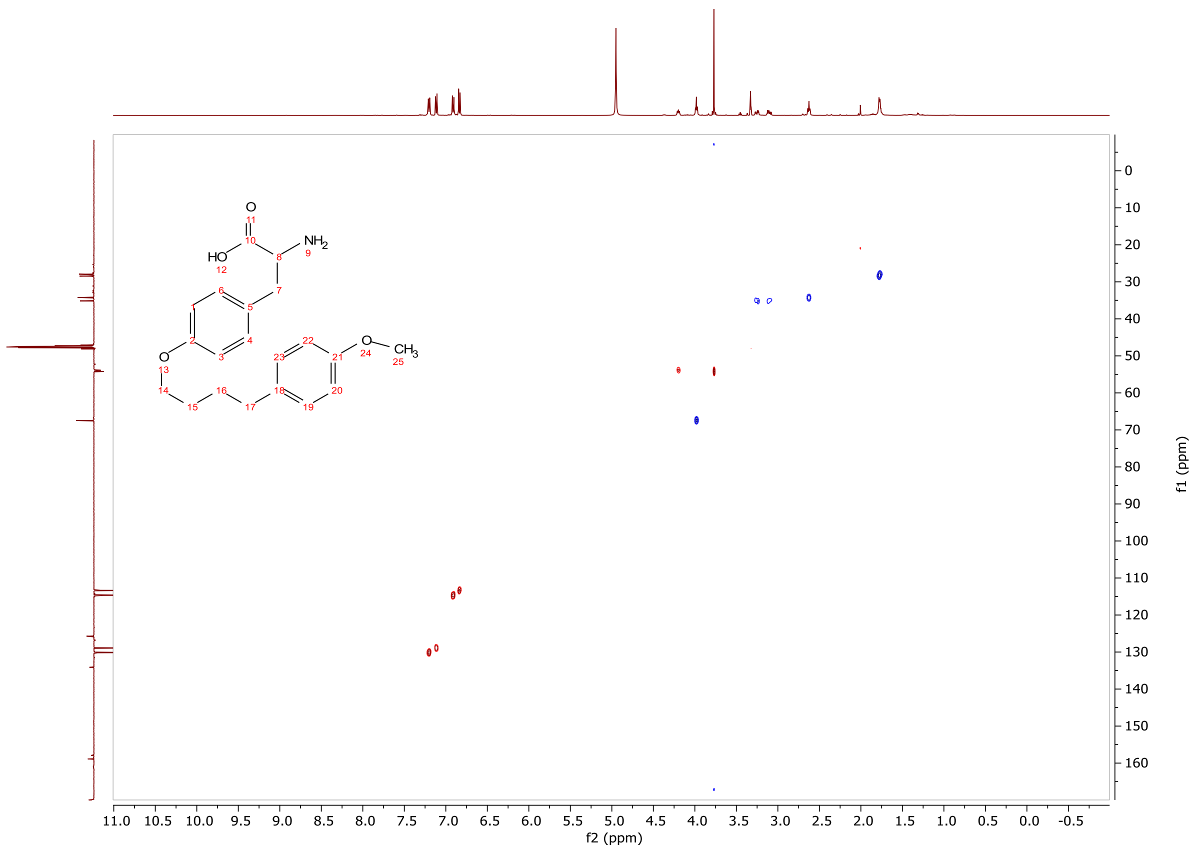

O
11
10
HO
12
8
NH2
9
7
6
5
4
3
2
O
13
14
15
16
17
18
19
20
21
22
23
O
24
CH3
25
f1 (ppm)
f2 (ppm)

3-(4-methoxyphenethyl)-1H-indole- 3h

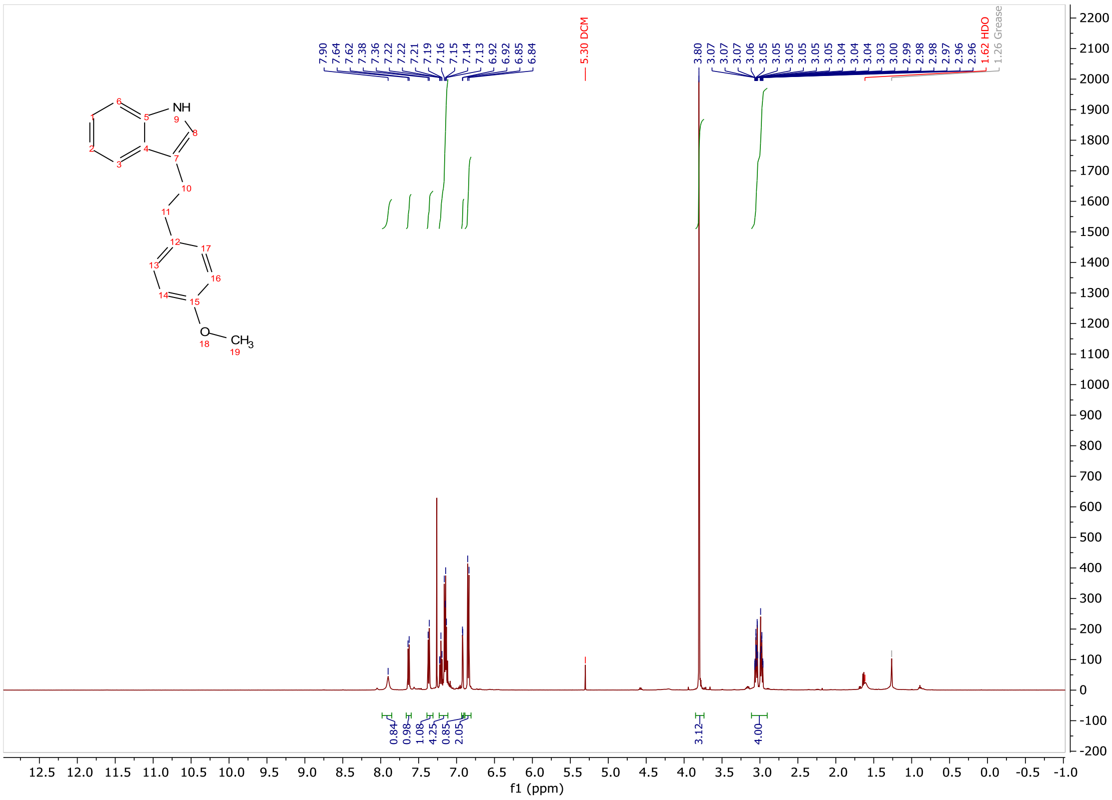

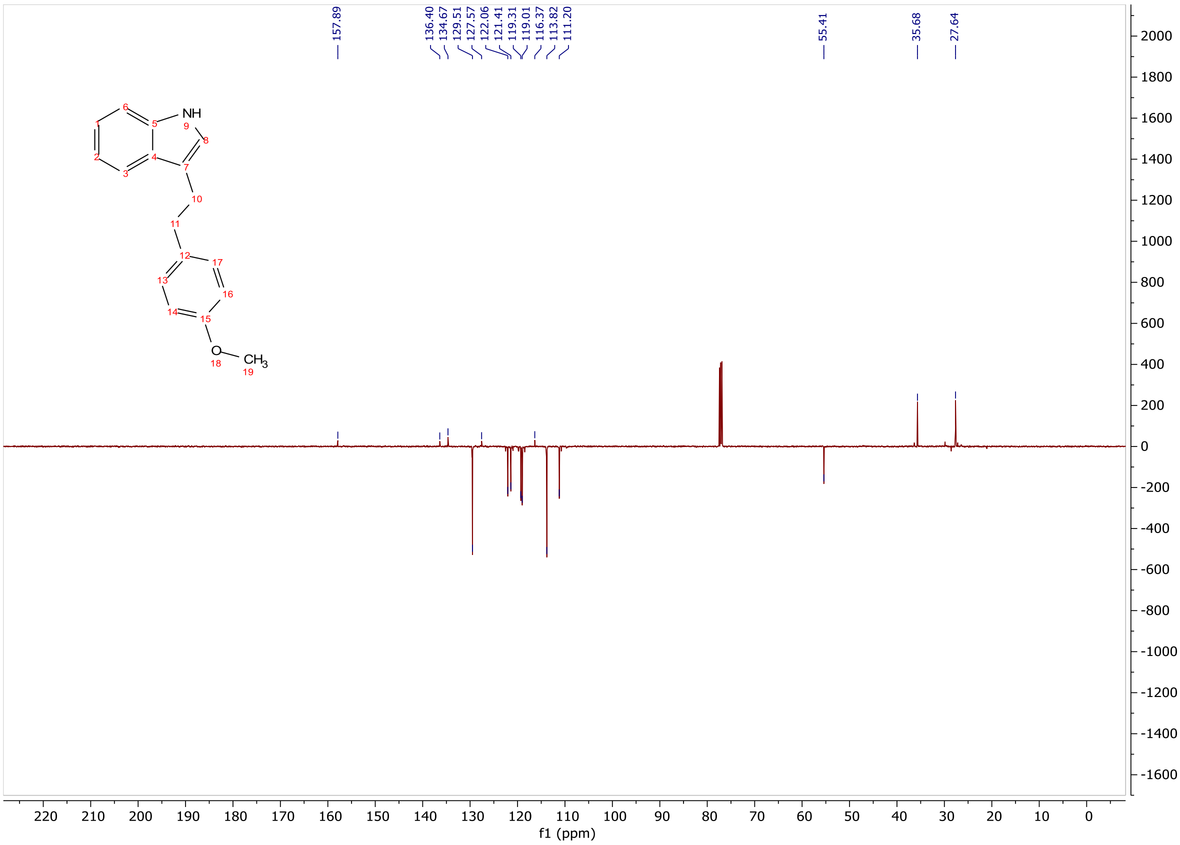

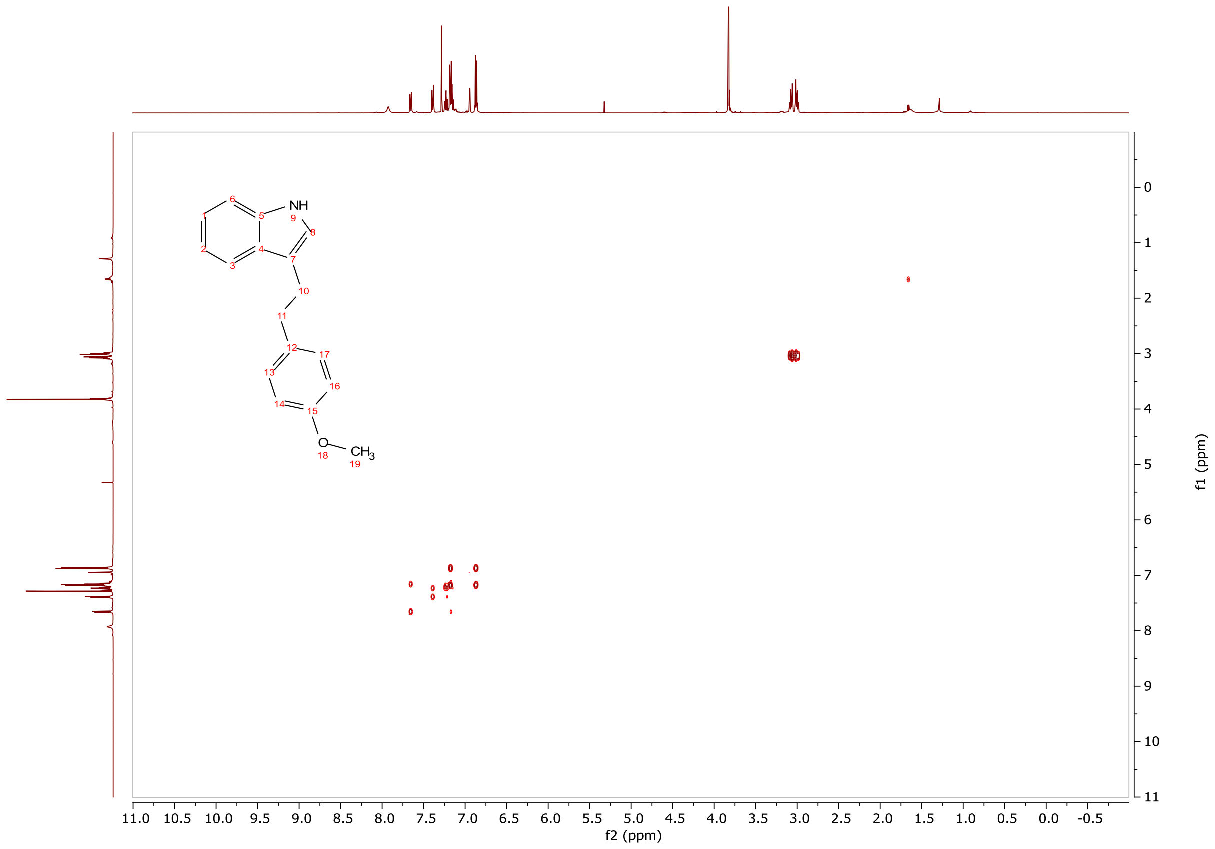

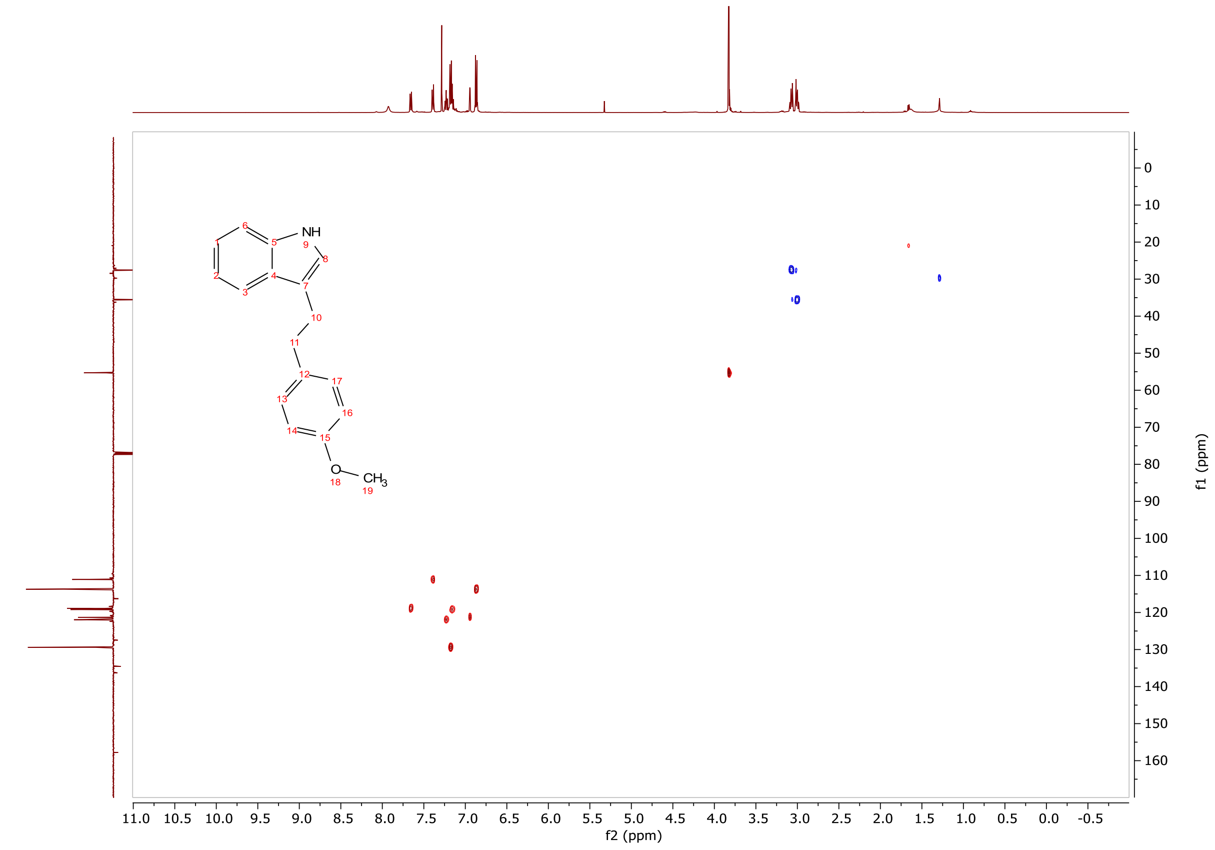

7-(4-(4-methoxyphenyl)butoxy)-2H-chromen-2-one- 4i

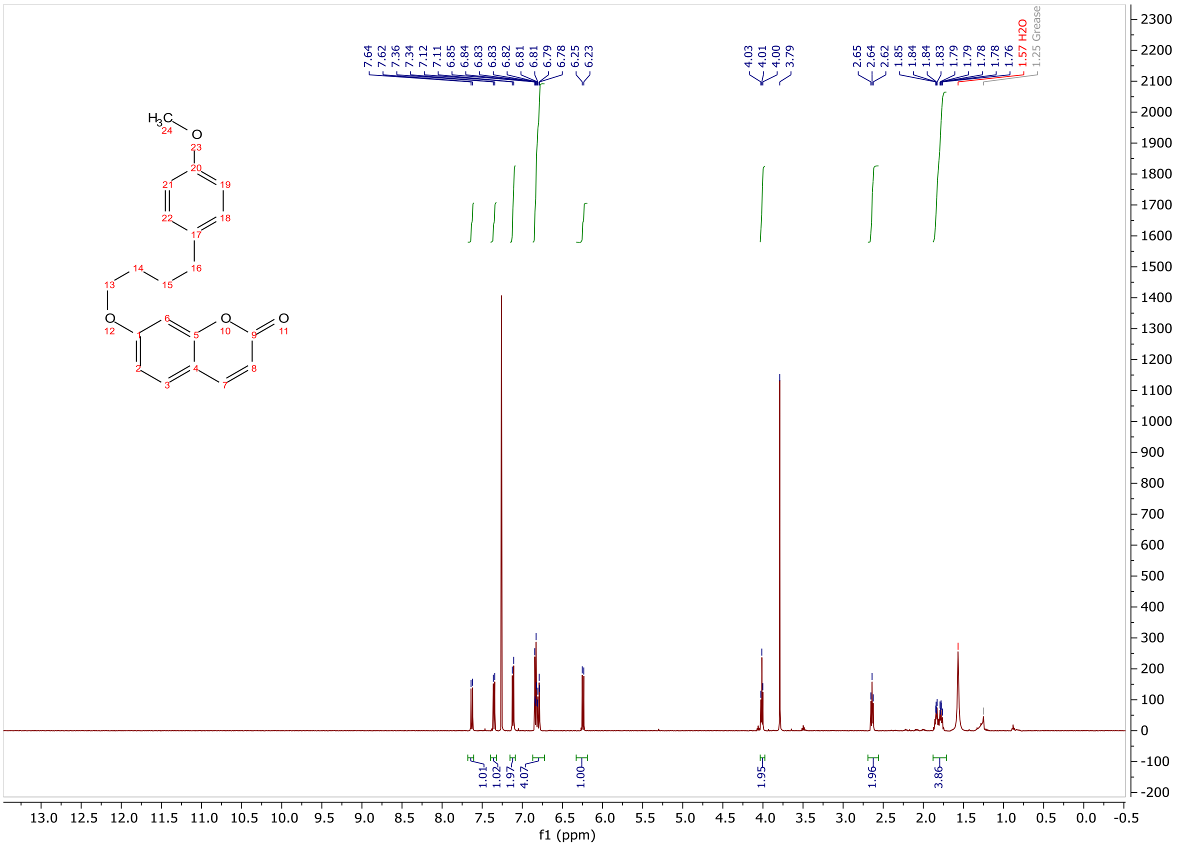

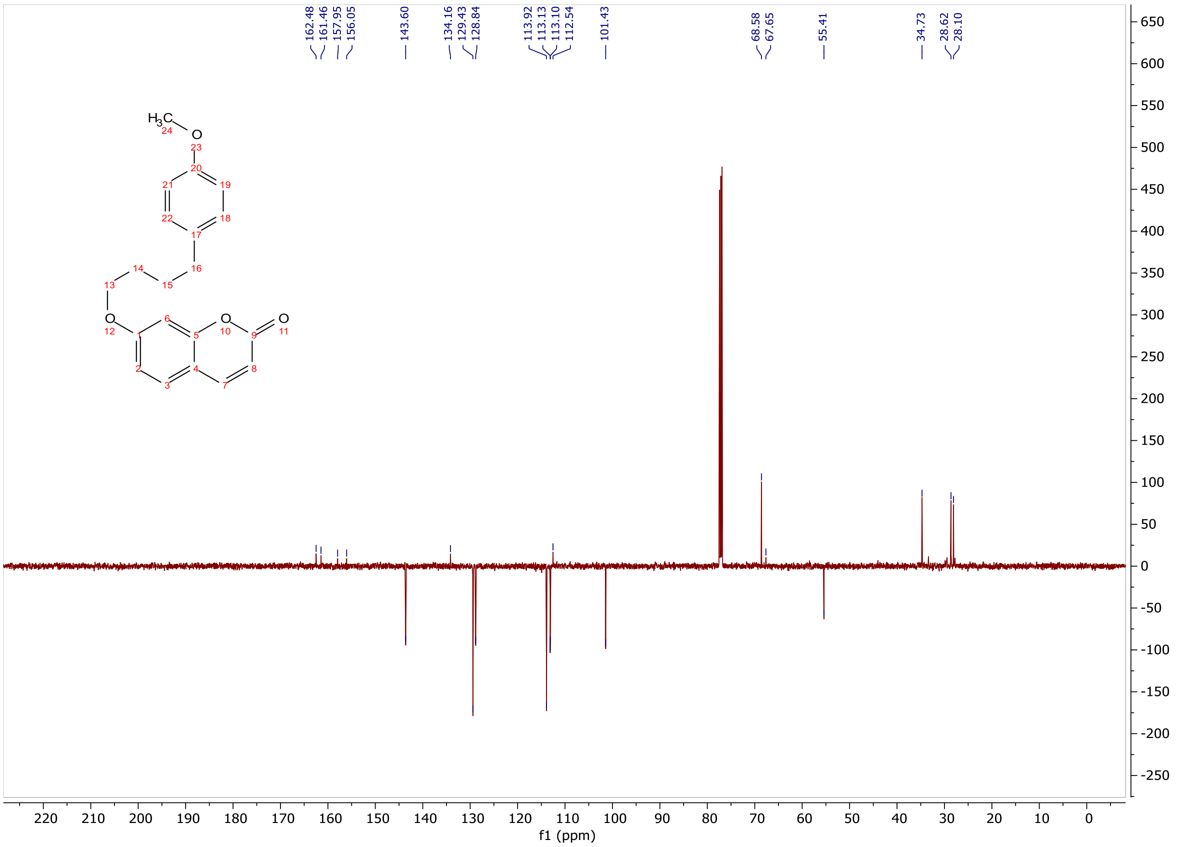

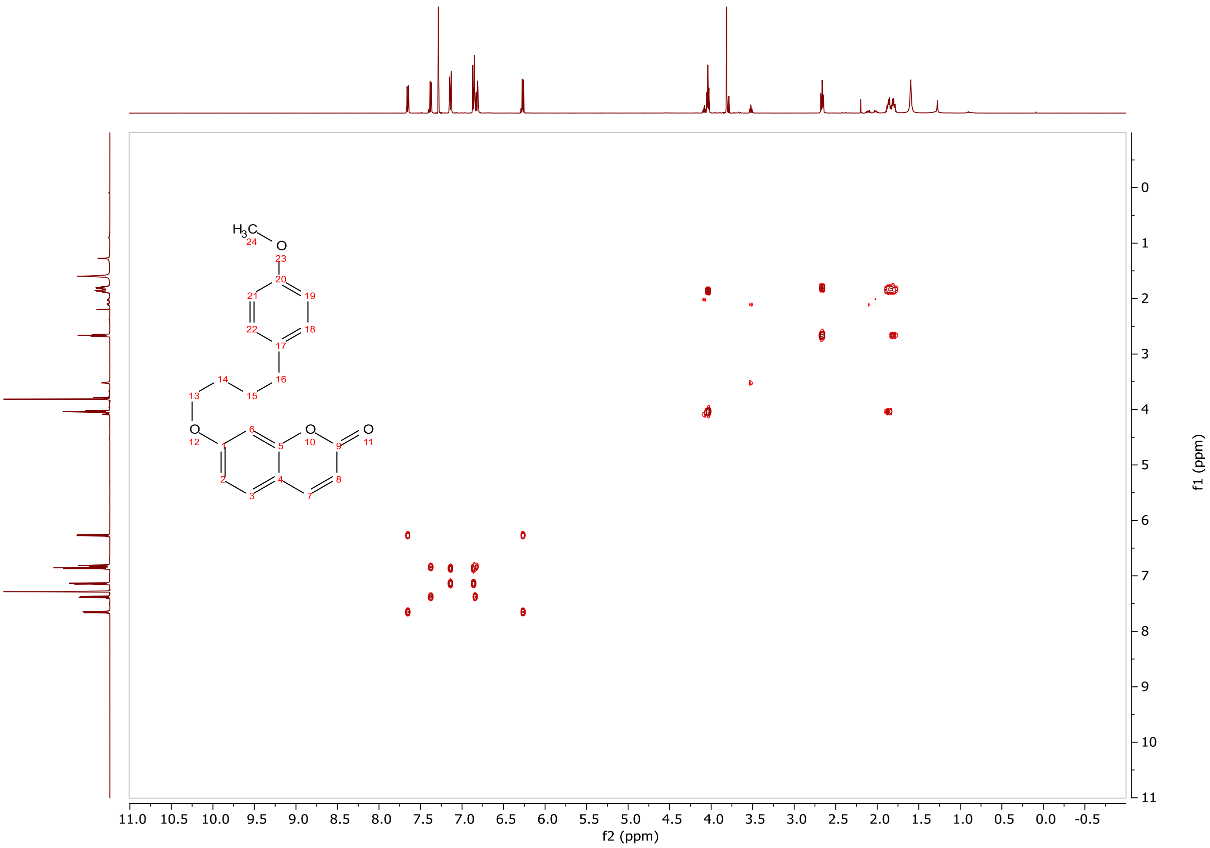
H3C
24
O
23
20
21
19
22
18
17
16
14
15
13
O
12
1
6
5
O
10
9
O
11
2
3
4
7
8
11.0 10.5 10.0 9.5 9.0 8.5 8.0 7.5 7.0 6.5 6.0 5.5 5.0 4.5 4.0 3.5 3.0 2.5 2.0 1.5 1.0 0.5 0.0 -0.5
f2 (ppm)
0 1 2 3 4 5 6 7 8 9 10 11
f1 (ppm)

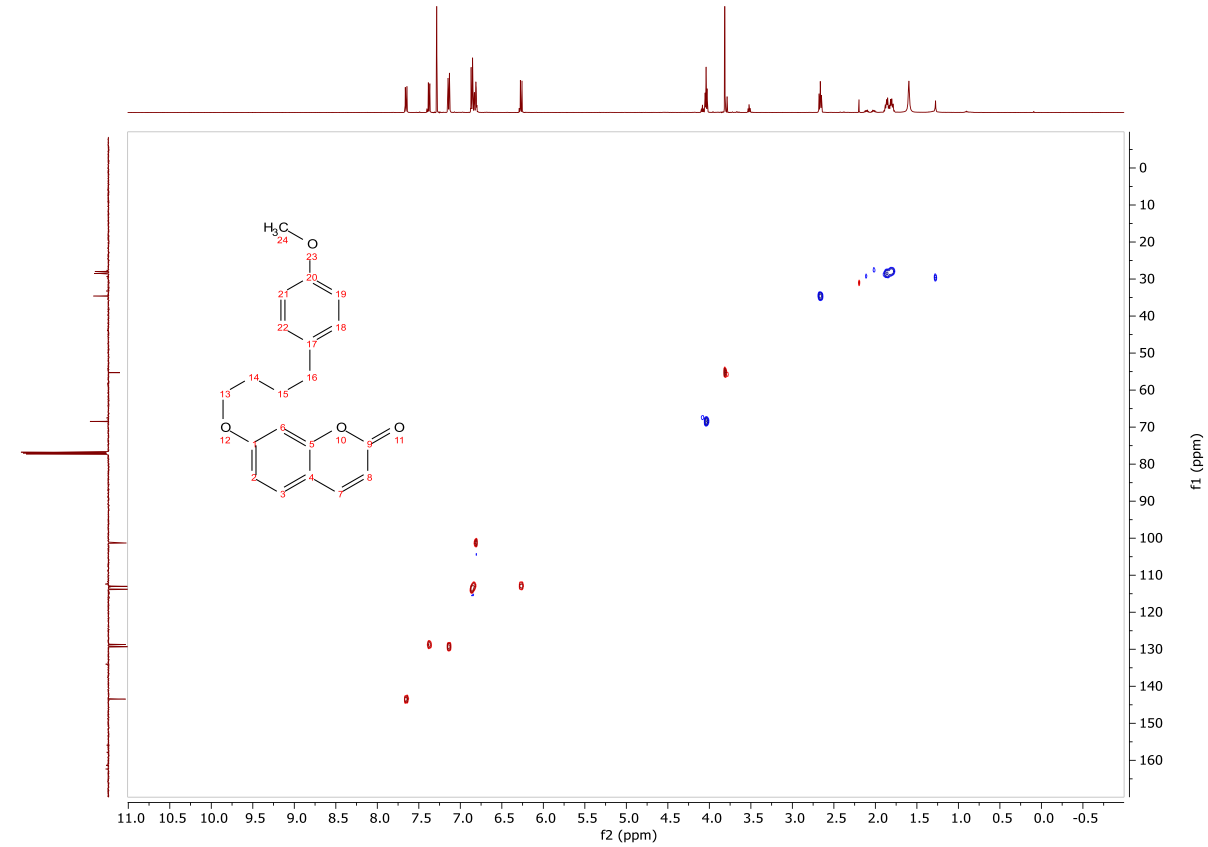
H3C
24
O
23
20
21
19
22
18
17
16
14
15
13
O
12
1
6
5
O
10
9
O
11
2
3
4
7
8
11.0 10.5 10.0 9.5 9.0 8.5 8.0 7.5 7.0 6.5 6.0 5.5 5.0 4.5 4.0 3.5 3.0 2.5 2.0 1.5 1.0 0.5 0.0 -0.5
f2 (ppm)
0 10 20 30 40 50 60 70 80 90 100 110 120 130 140 150 160
f1 (ppm)

1-(4-methoxyphenethyl)indoline-2,3-dione- 3j

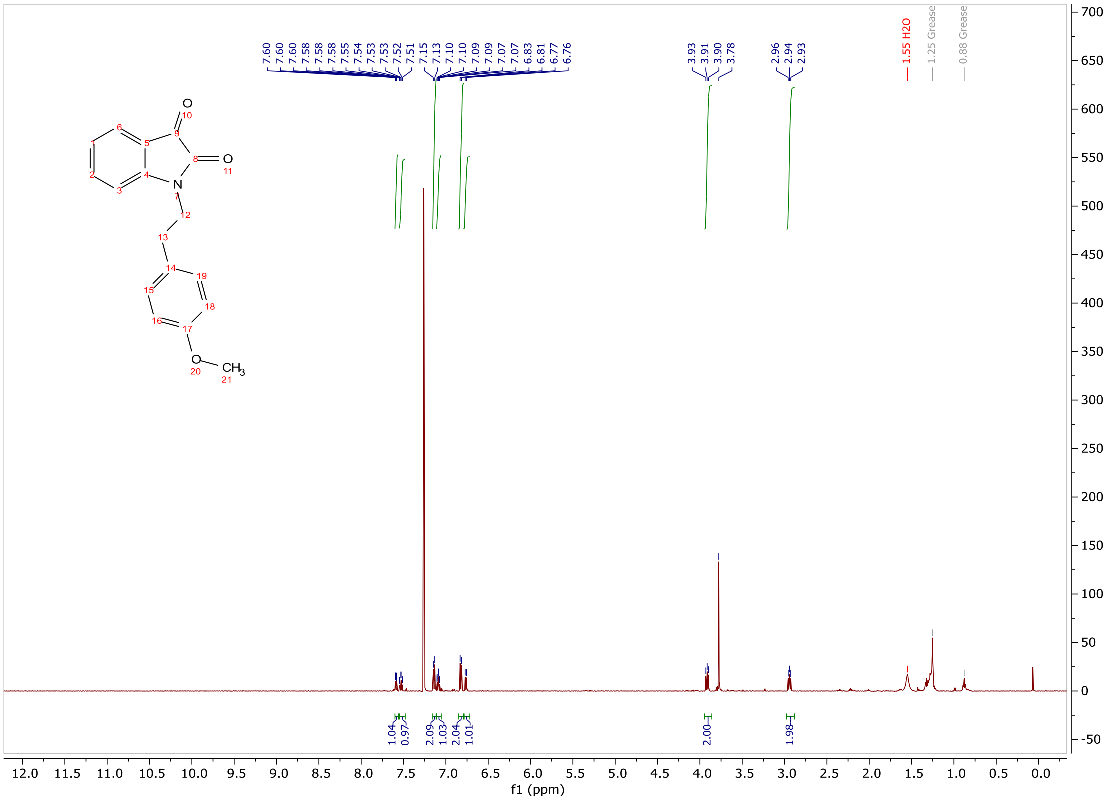

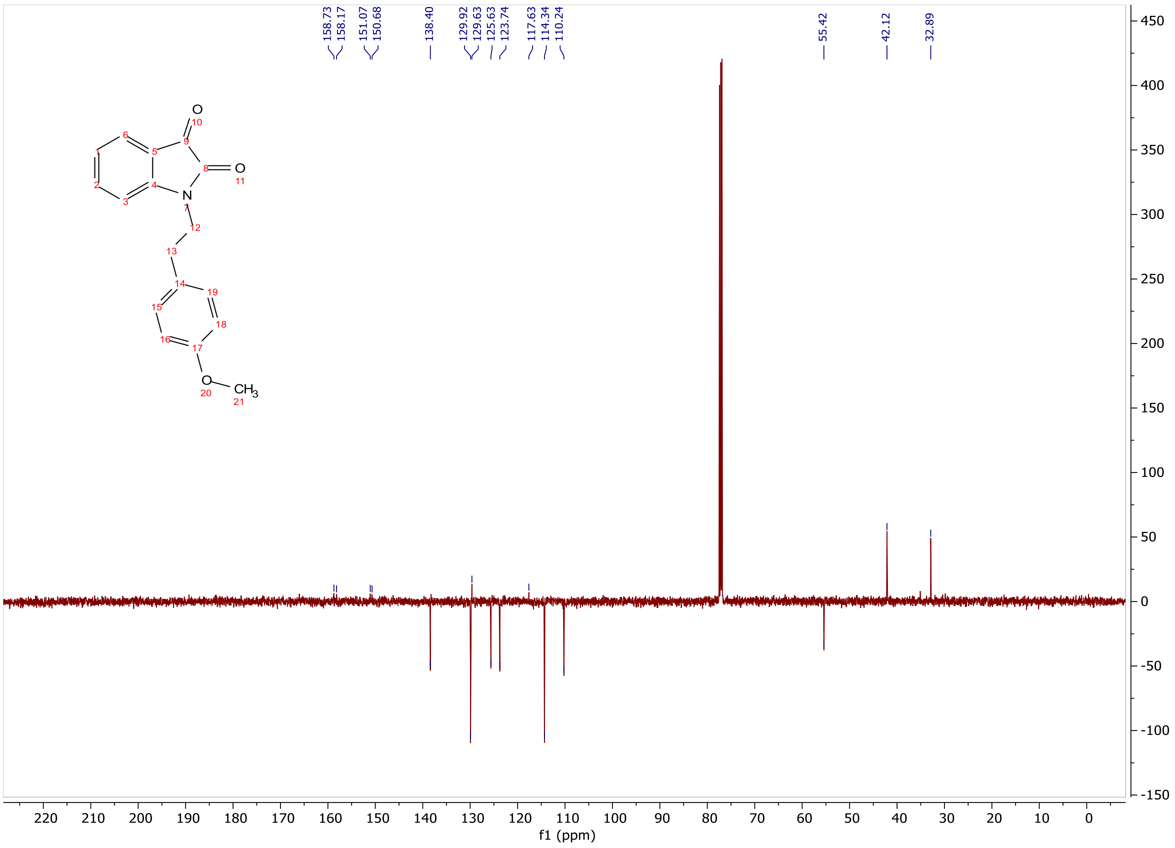

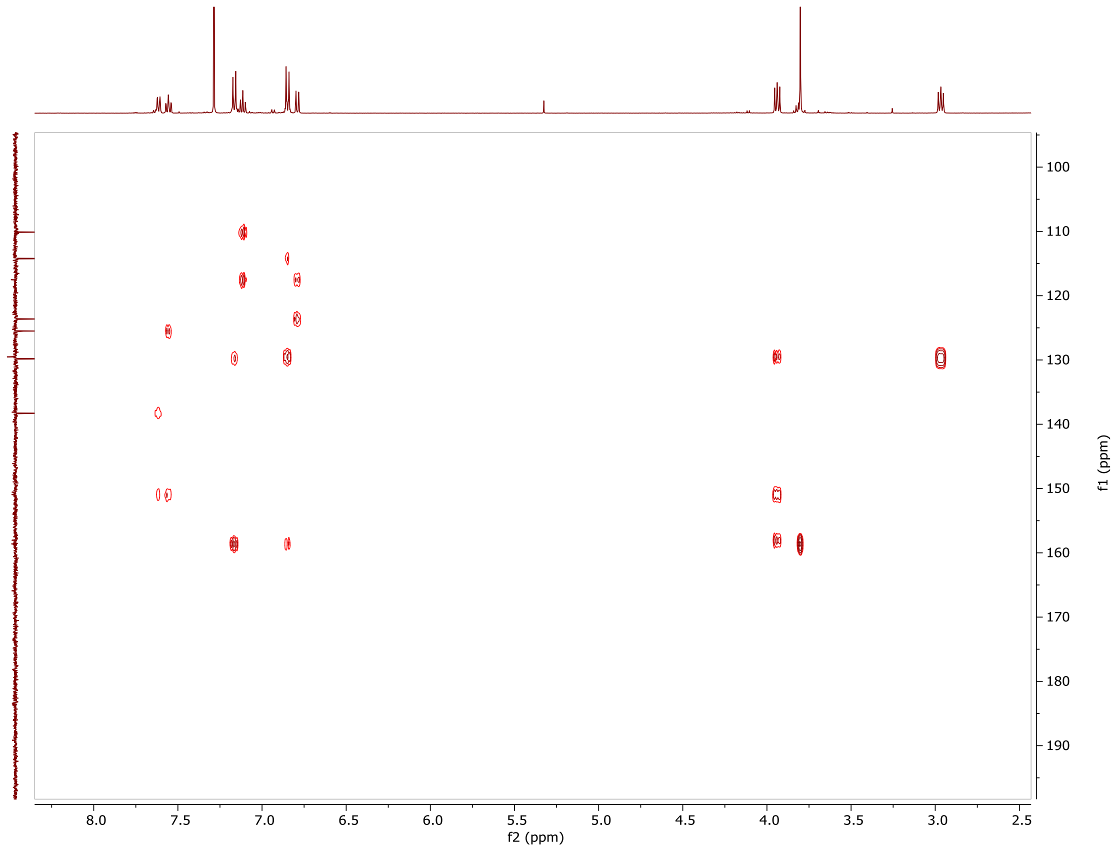
f2 (ppm)
f1 (ppm)

Methyl (2-amino-8-(*p*-methoxyphenyl) octanoyl)-L-phenylalaninate – 3m

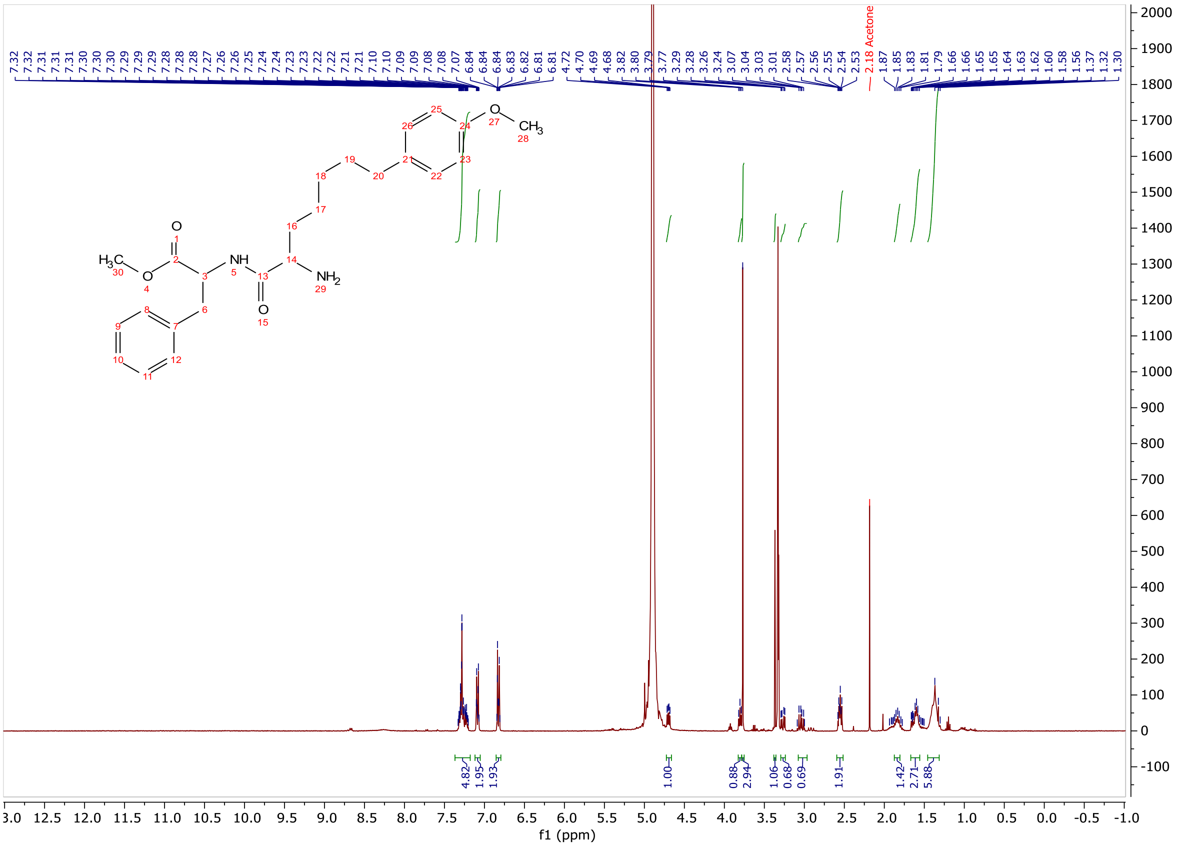

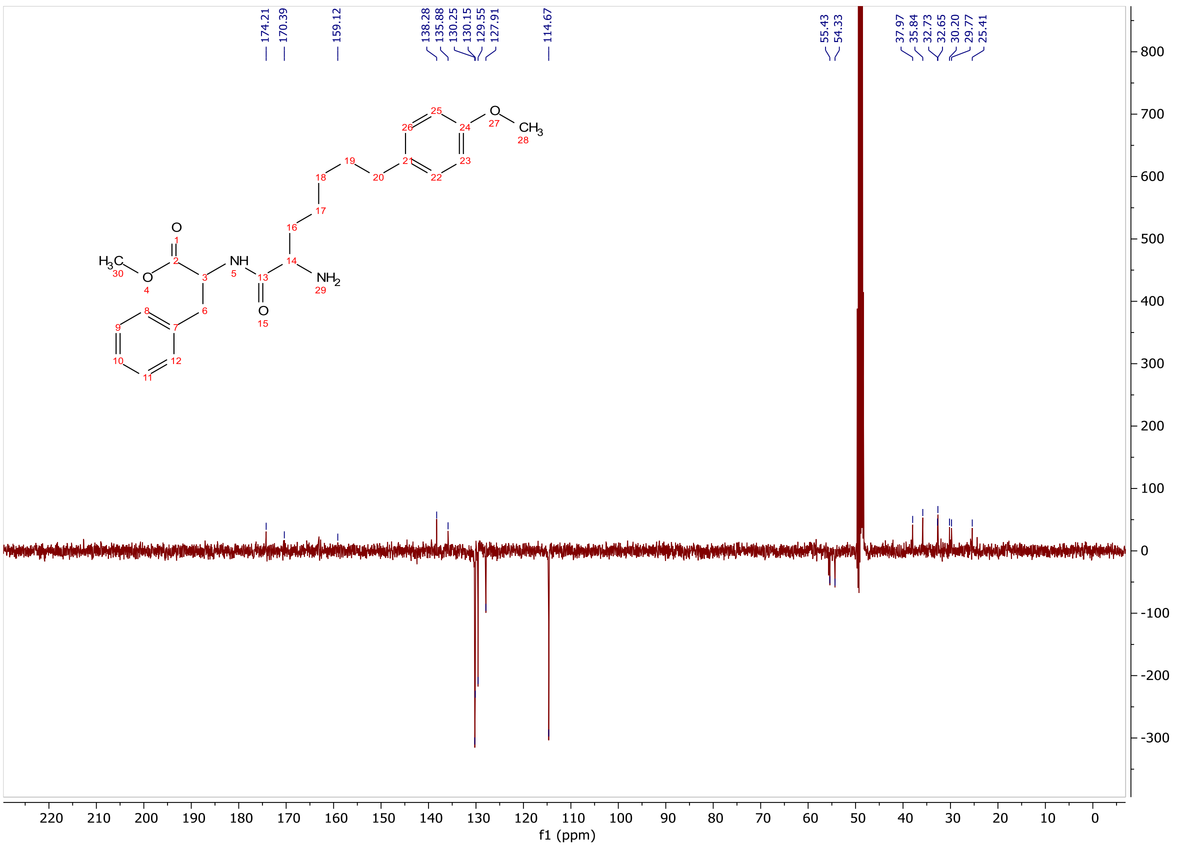

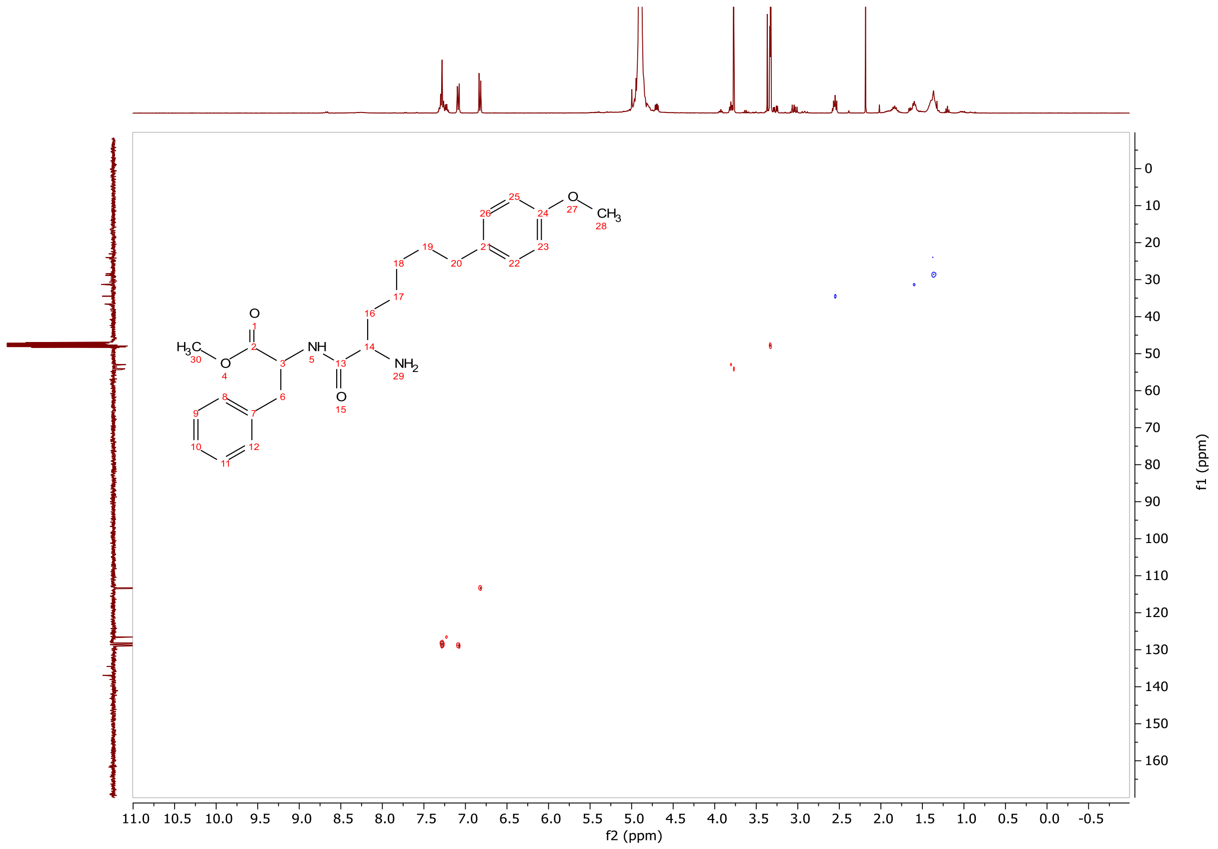

f2 (ppm)
f1 (ppm)

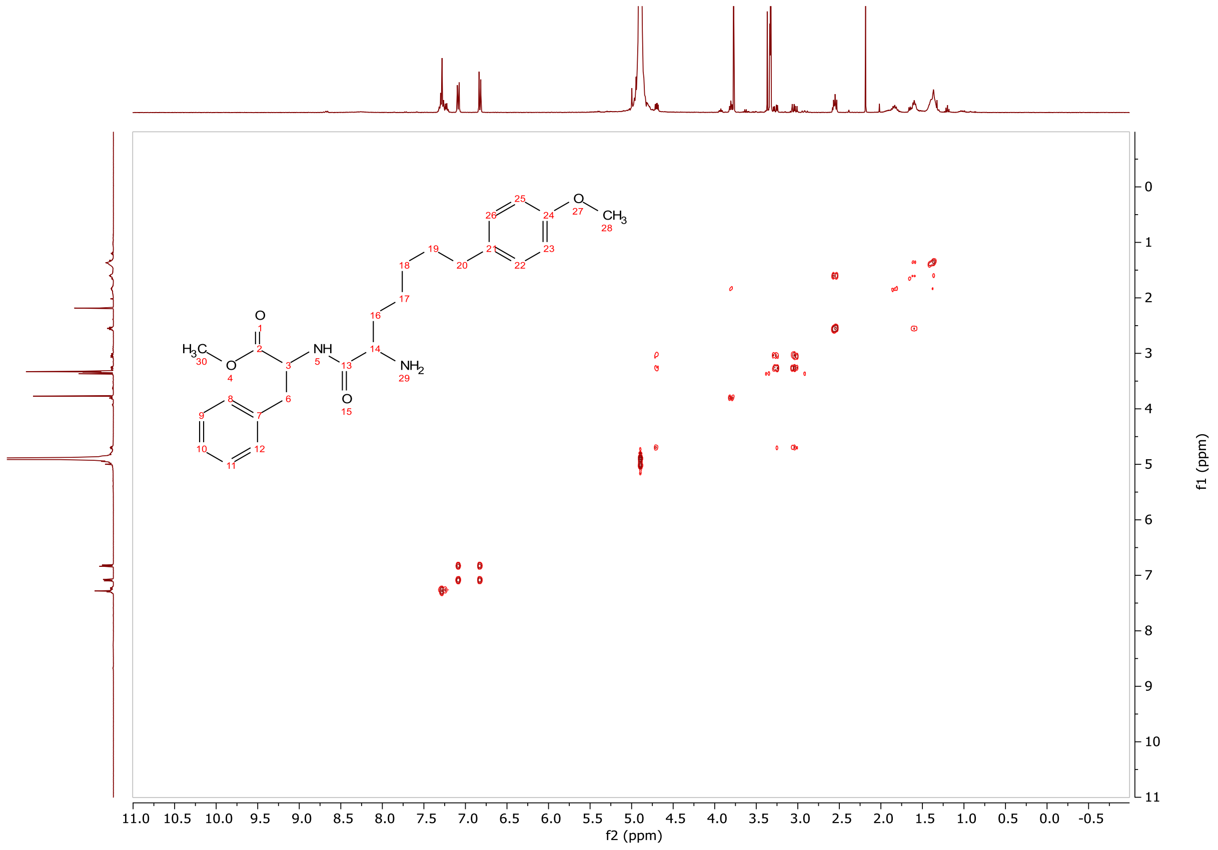

f2 (ppm)
f1 (ppm)

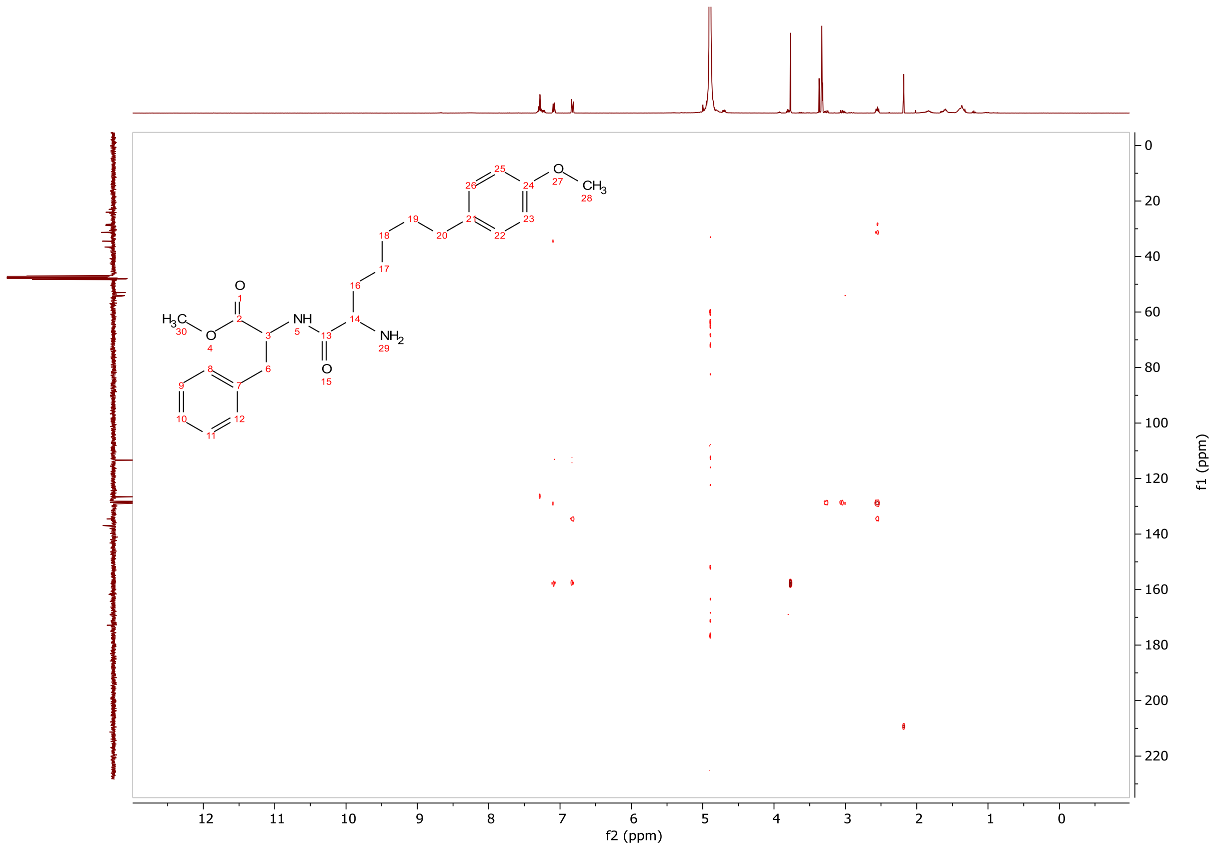
f2 (ppm)
f1 (ppm)

Coupled Metalachlor (N-(2-ethyl-6-methylphenyl)-2-(4-methoxyphenyl)-N-(1-methoxypropan-2-yl)acetamide)- 3k

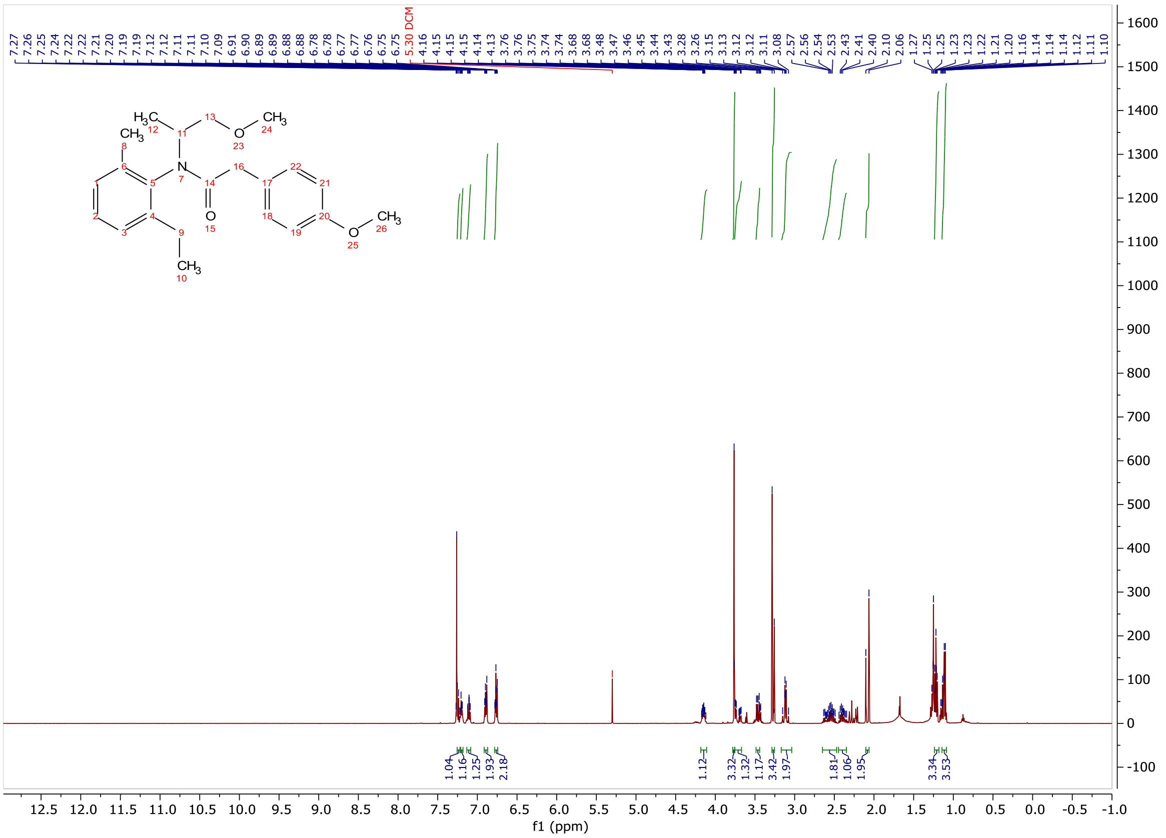

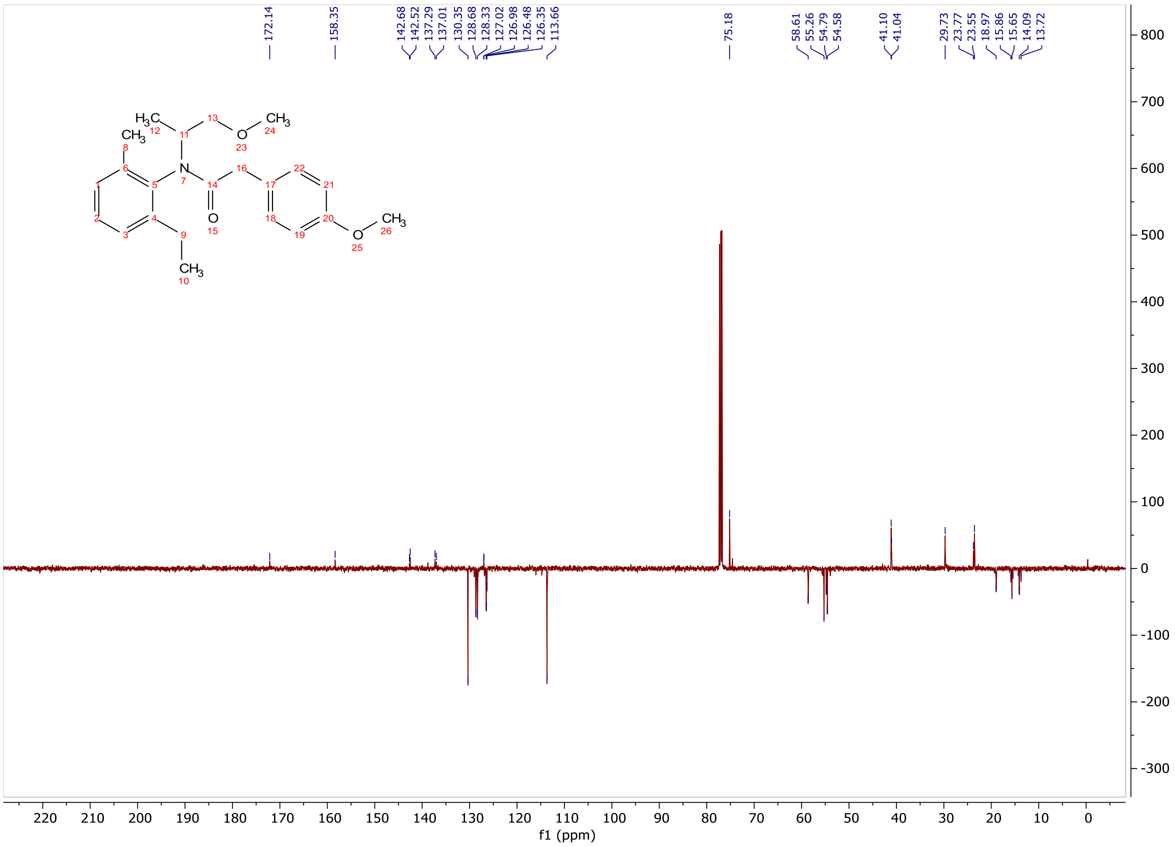

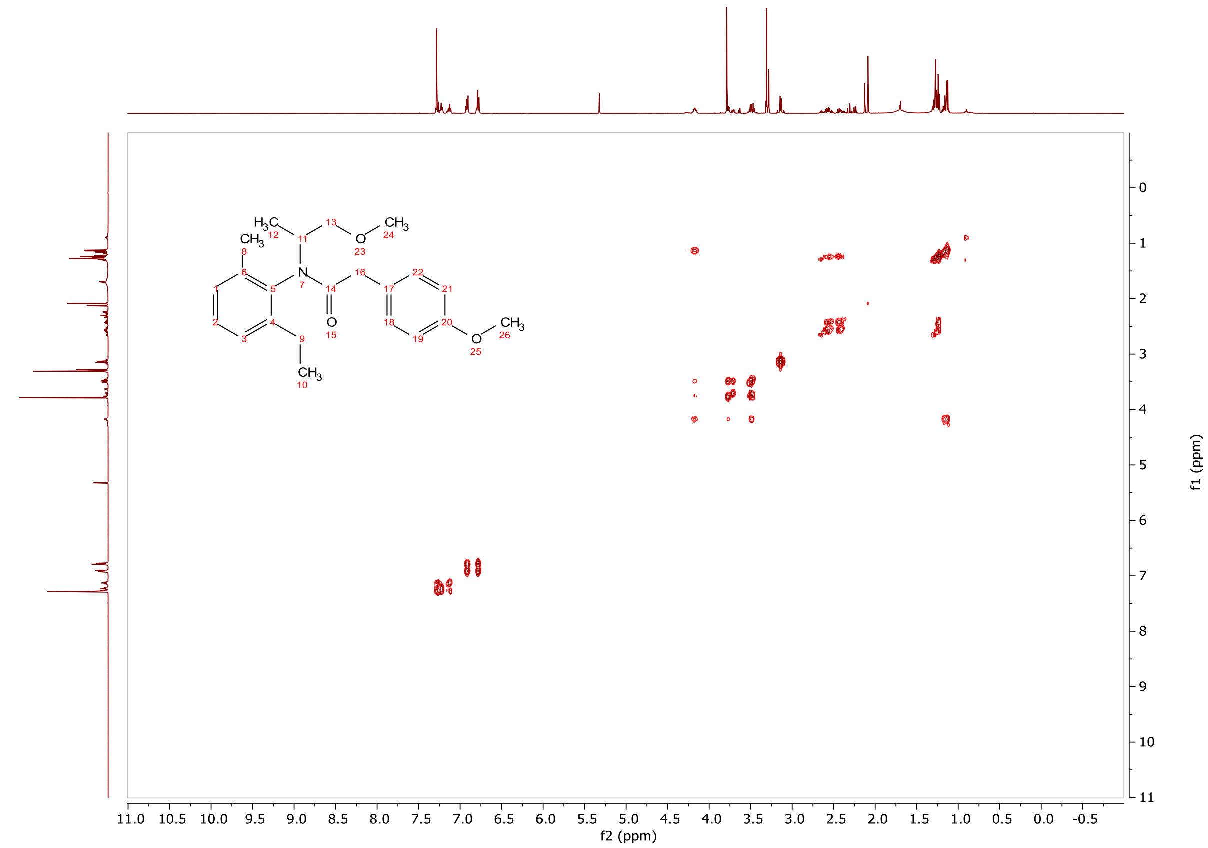

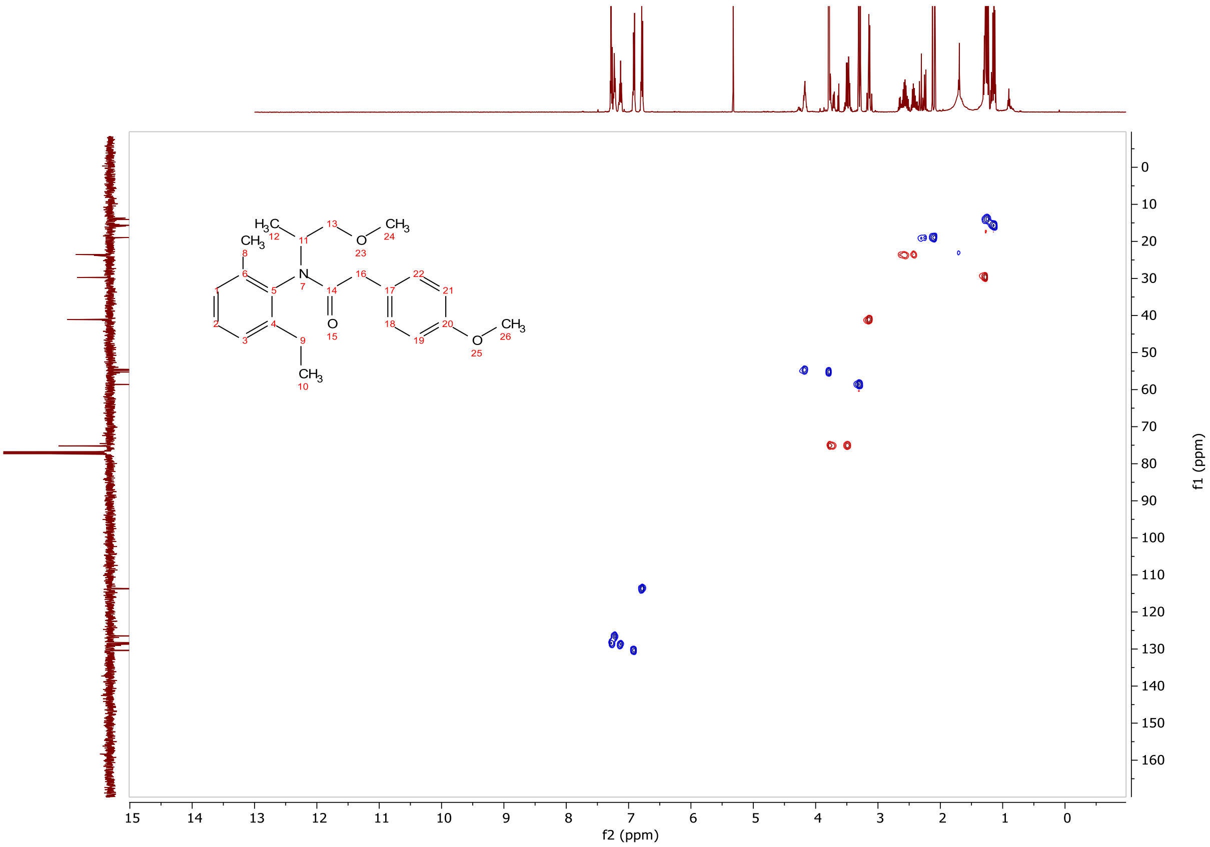

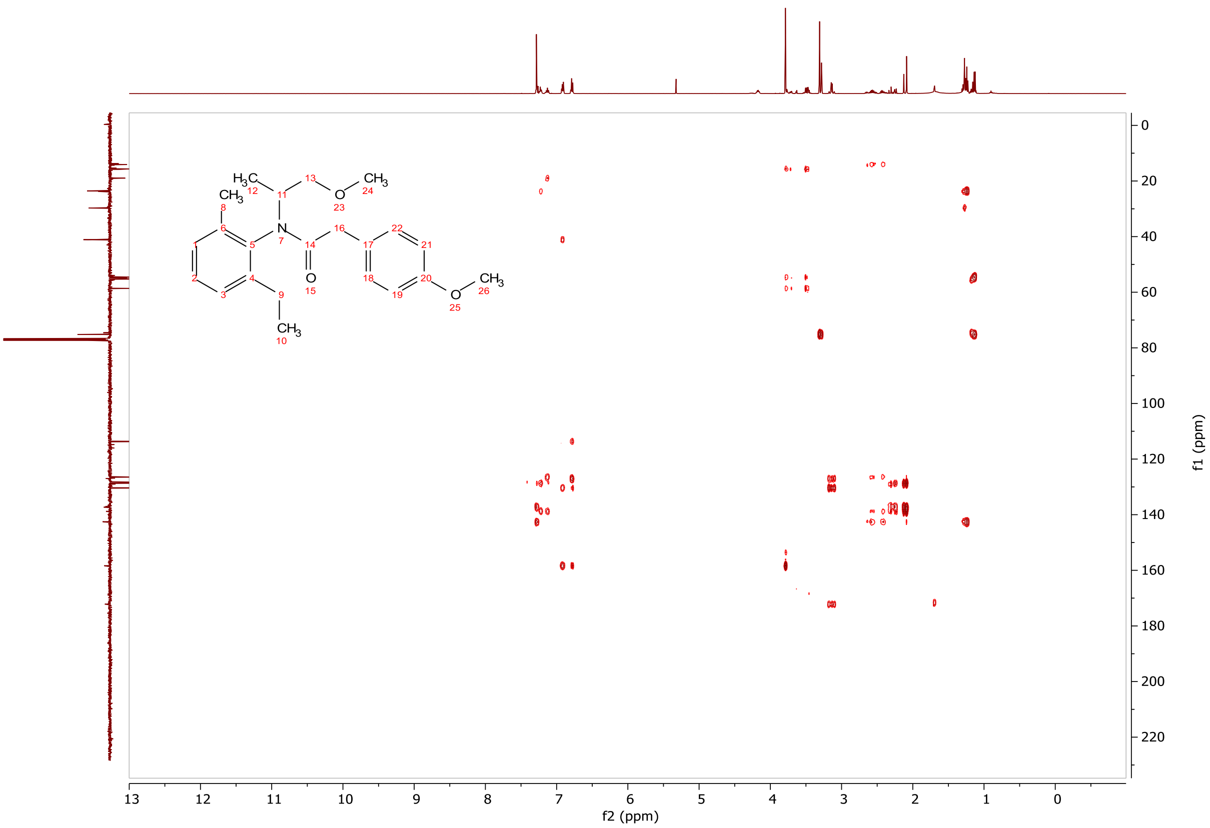
f2 (ppm)
f1 (ppm)

±2-((tert-butoxycarbonyl)amino)-8-(4-methoxyphenyl)octanoic acid- 3d

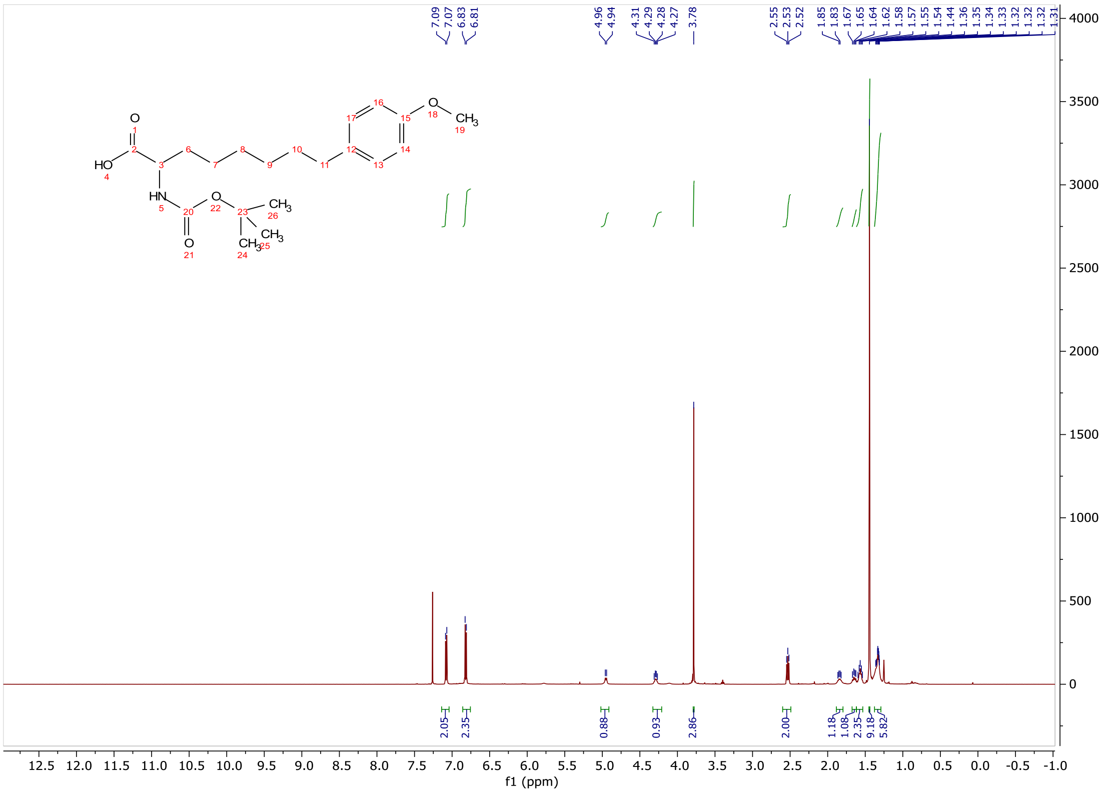

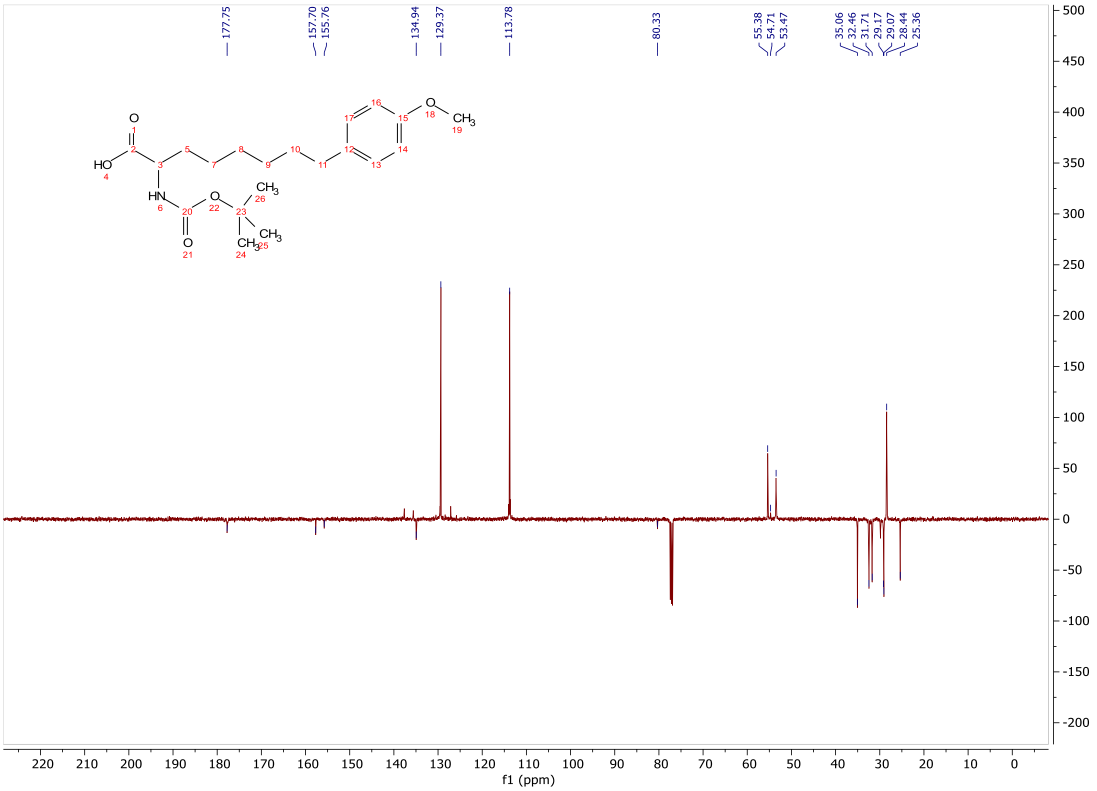

2-amino-8-(2-methoxyphenyl)octanoic acid- 4b

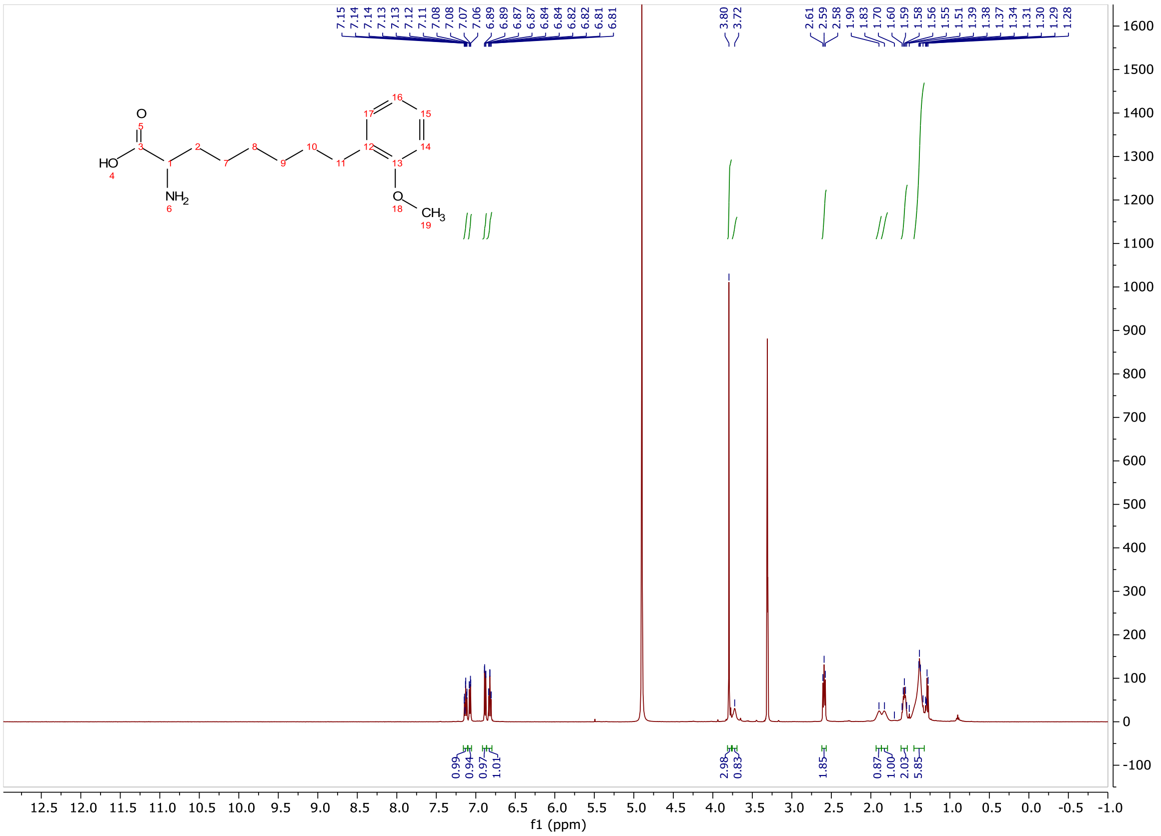

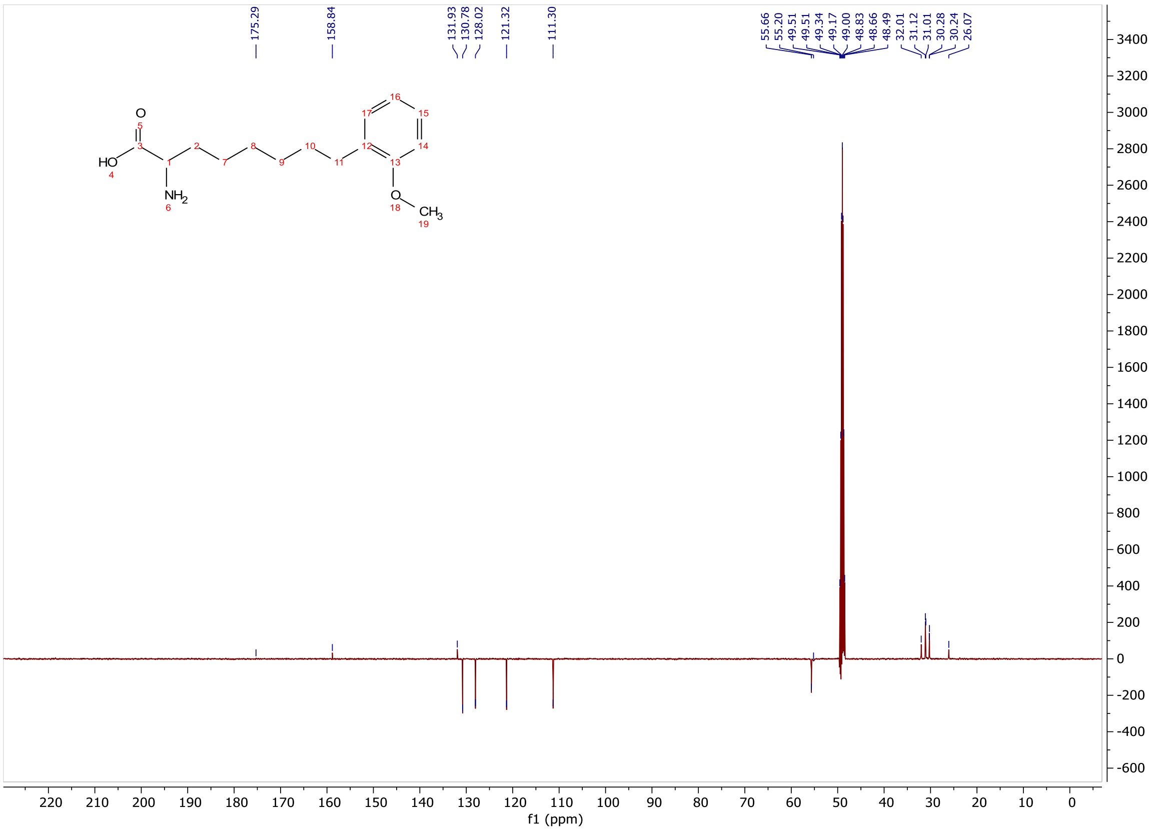

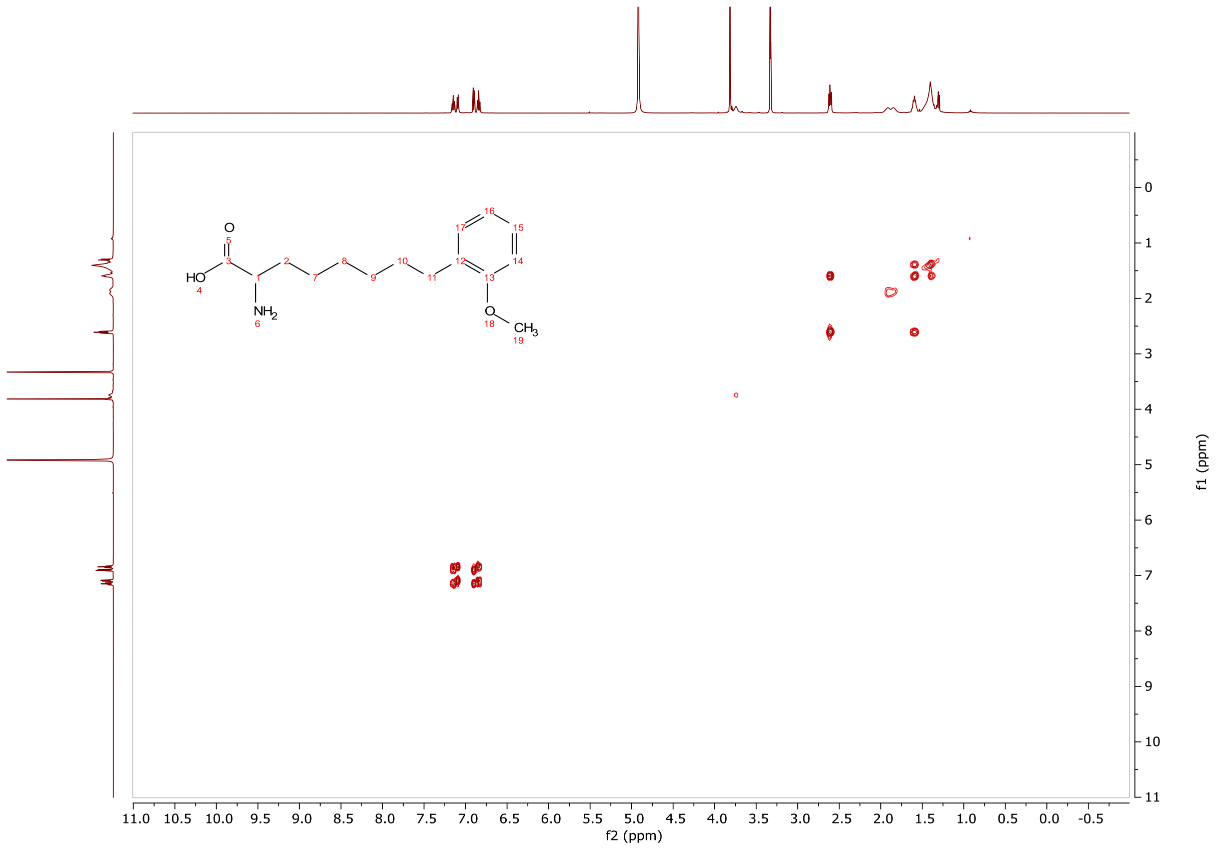

HO
4
O
5
3
1
2
7
8
9
10
11
12
13
14
15
16
17
NH2
6
O
18
CH3
19
f2 (ppm)
f1 (ppm)

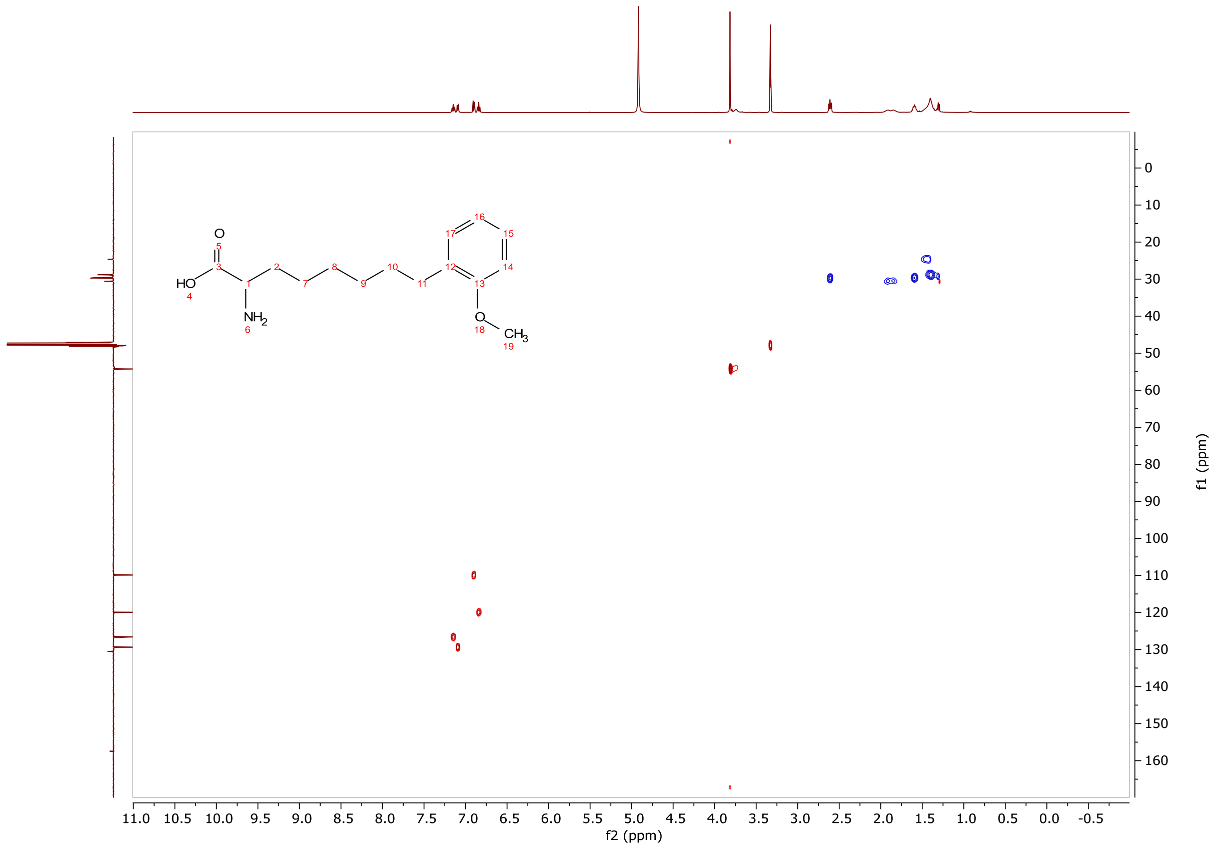

HO
4
O
5
3
1
2
7
8
9
10
11
12
13
14
15
16
17
NH2
6
O
18
CH3
19
f2 (ppm)
f1 (ppm)

2-amino-8-(p-tolyl)octanoic acid- 4c

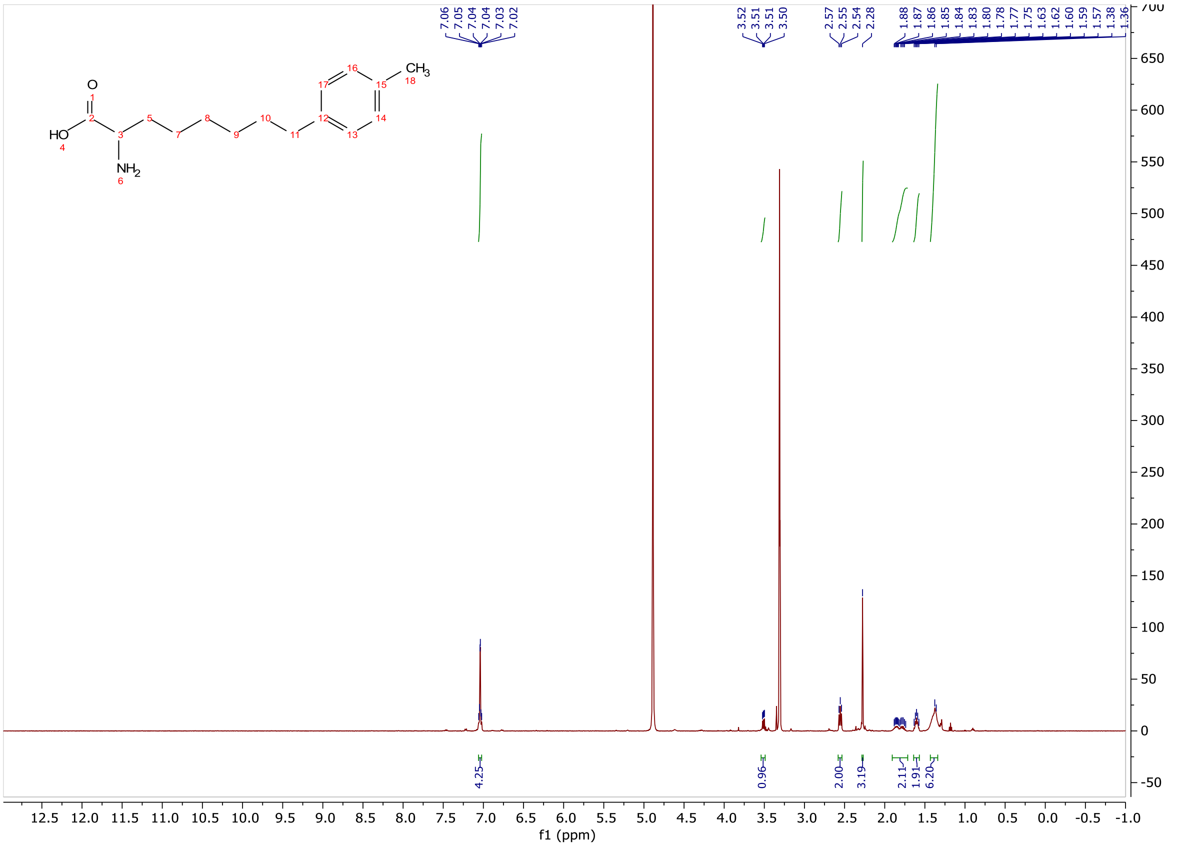

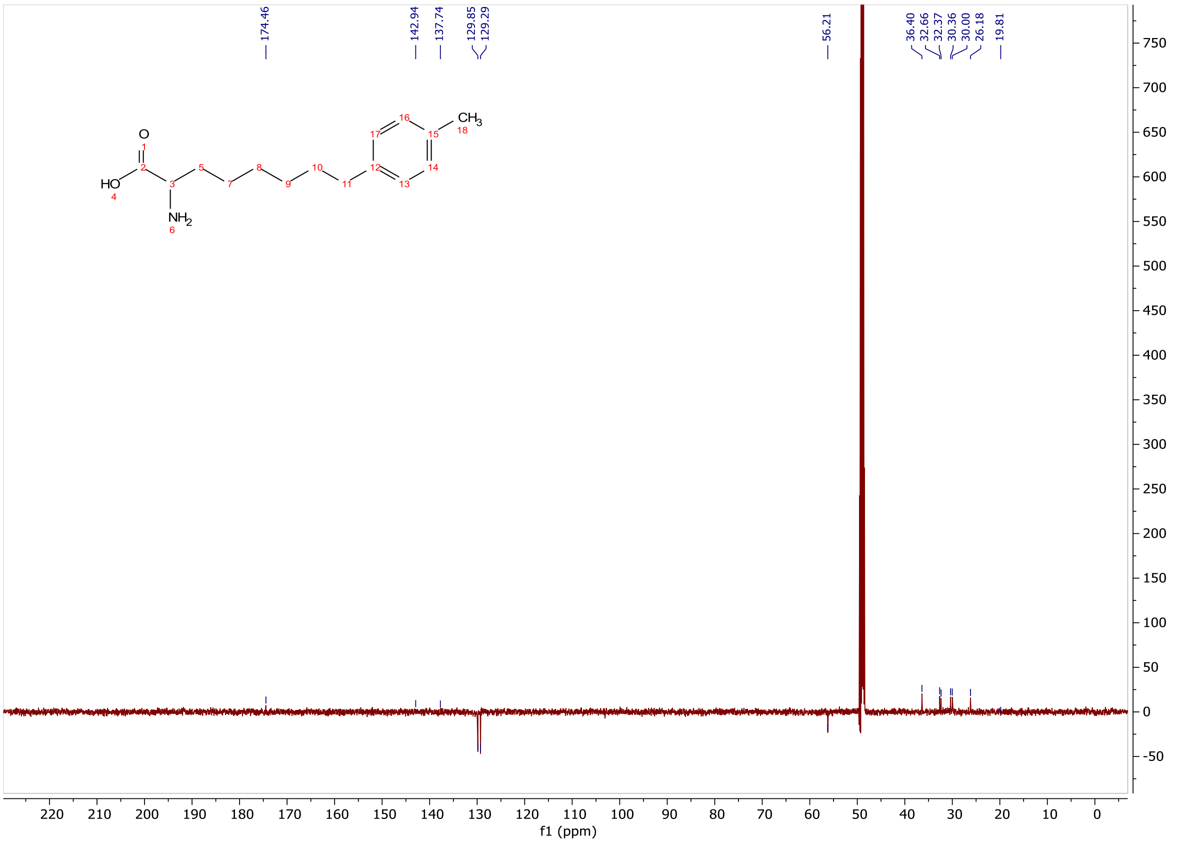

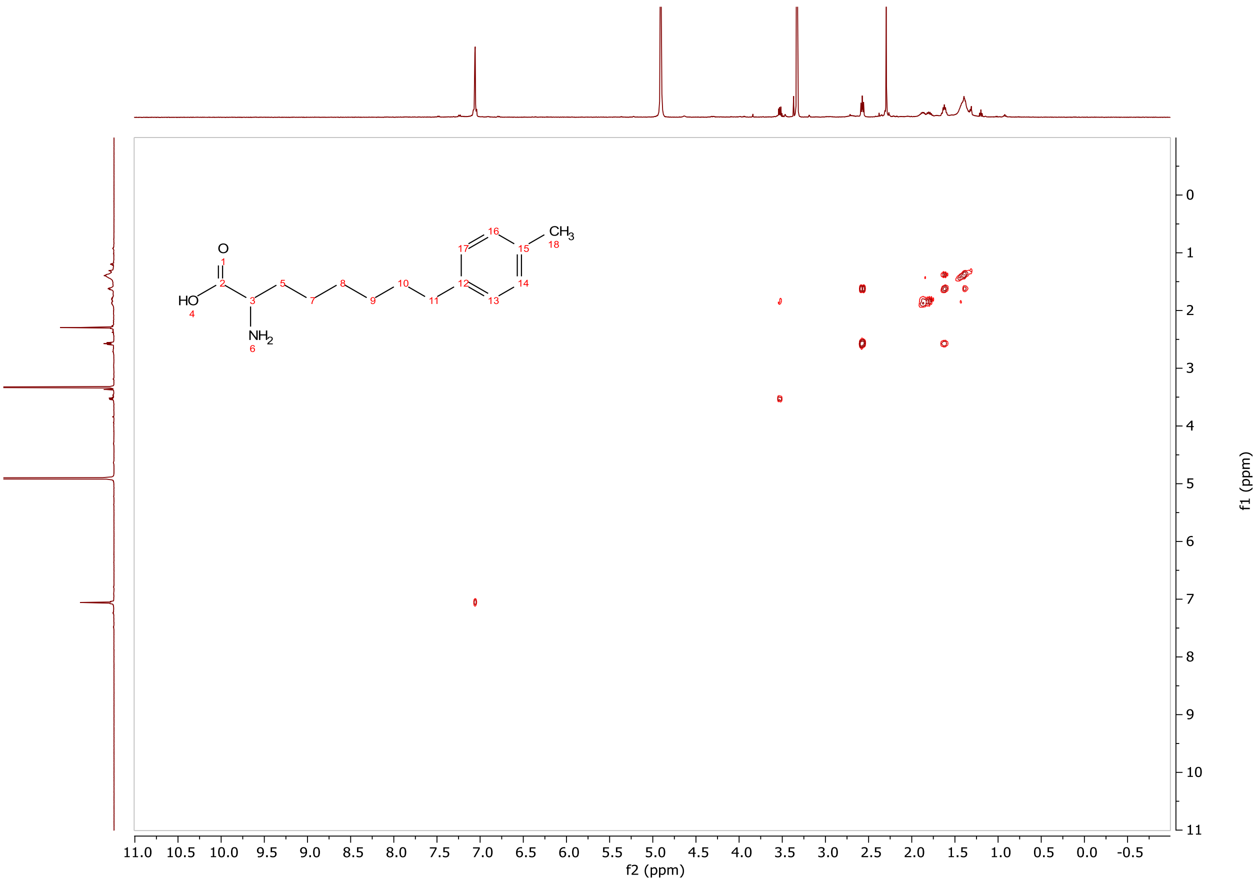

f2 (ppm)
f1 (ppm)
HO
NH2
CH3

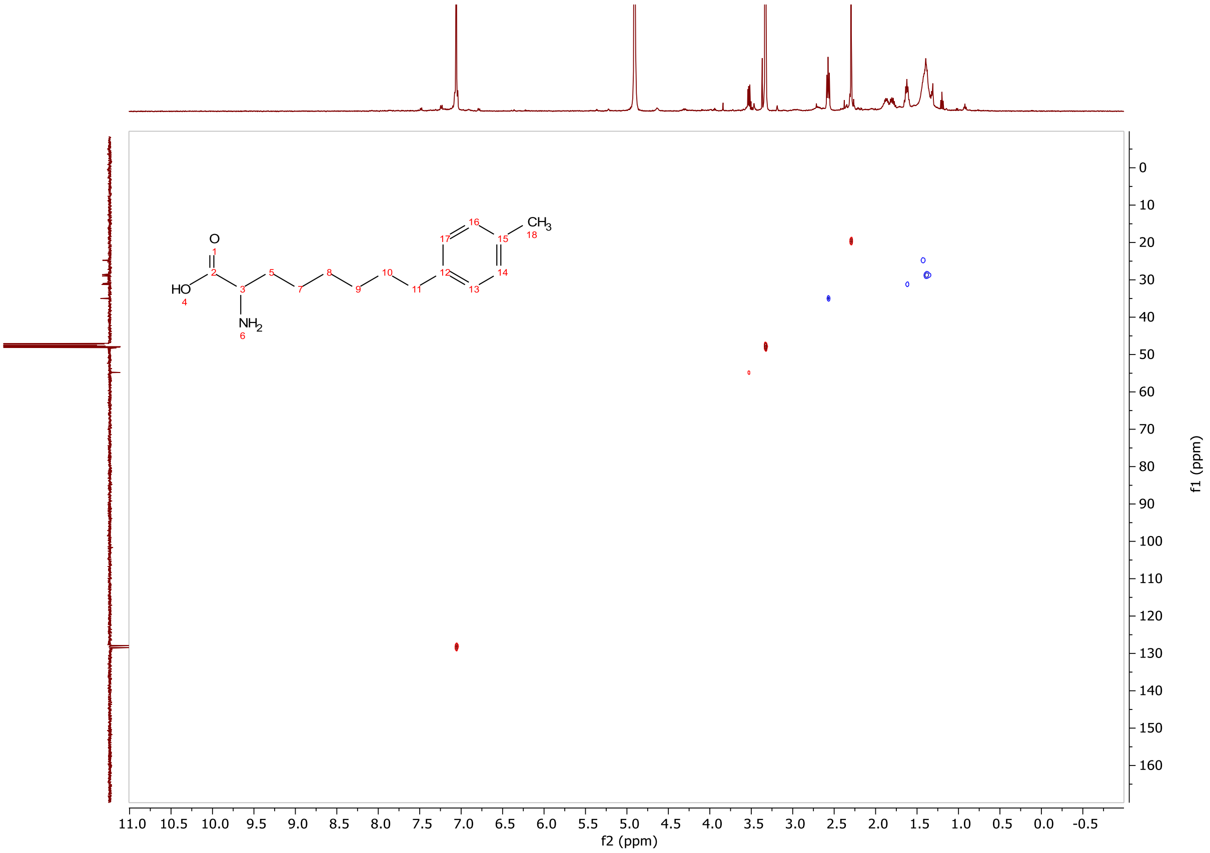

f2 (ppm)
f1 (ppm)
HO
NH2
CH3

2-amino-8-(σ-tolyl)-octanoic acid- 4d

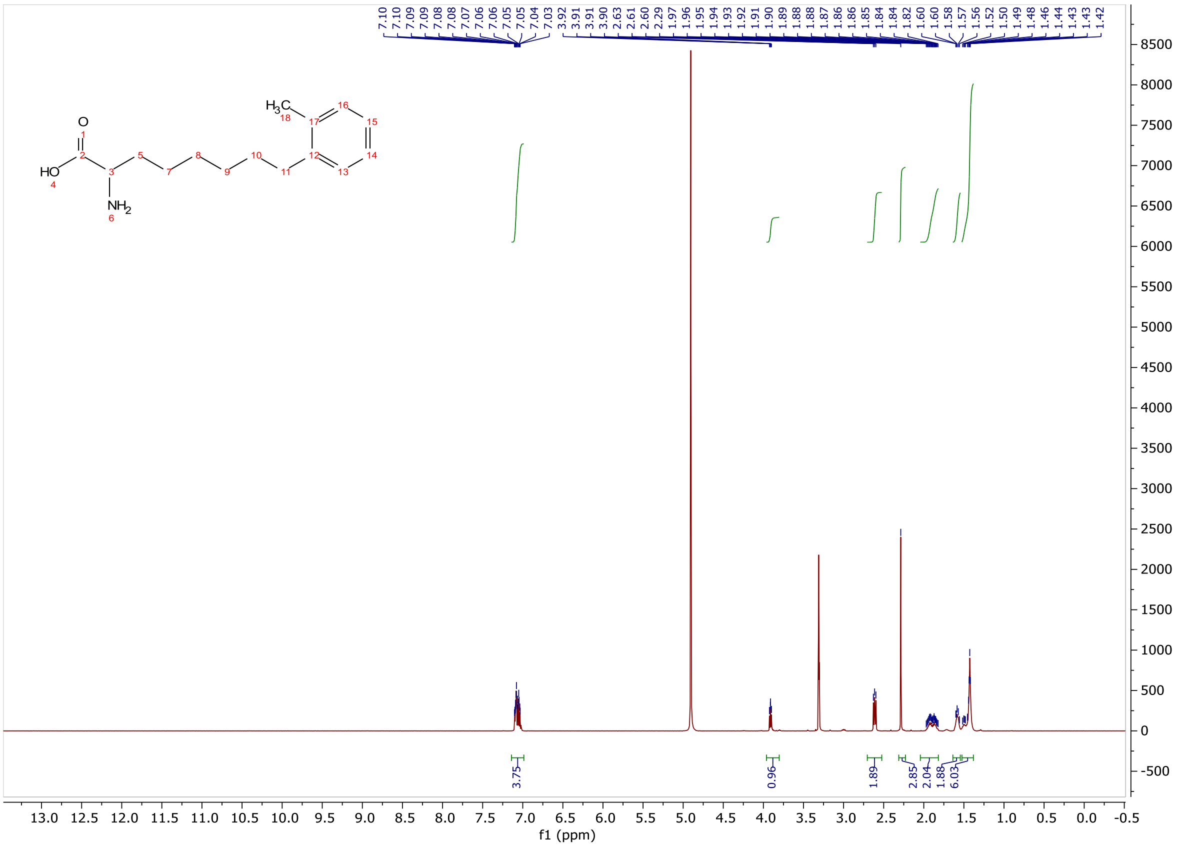

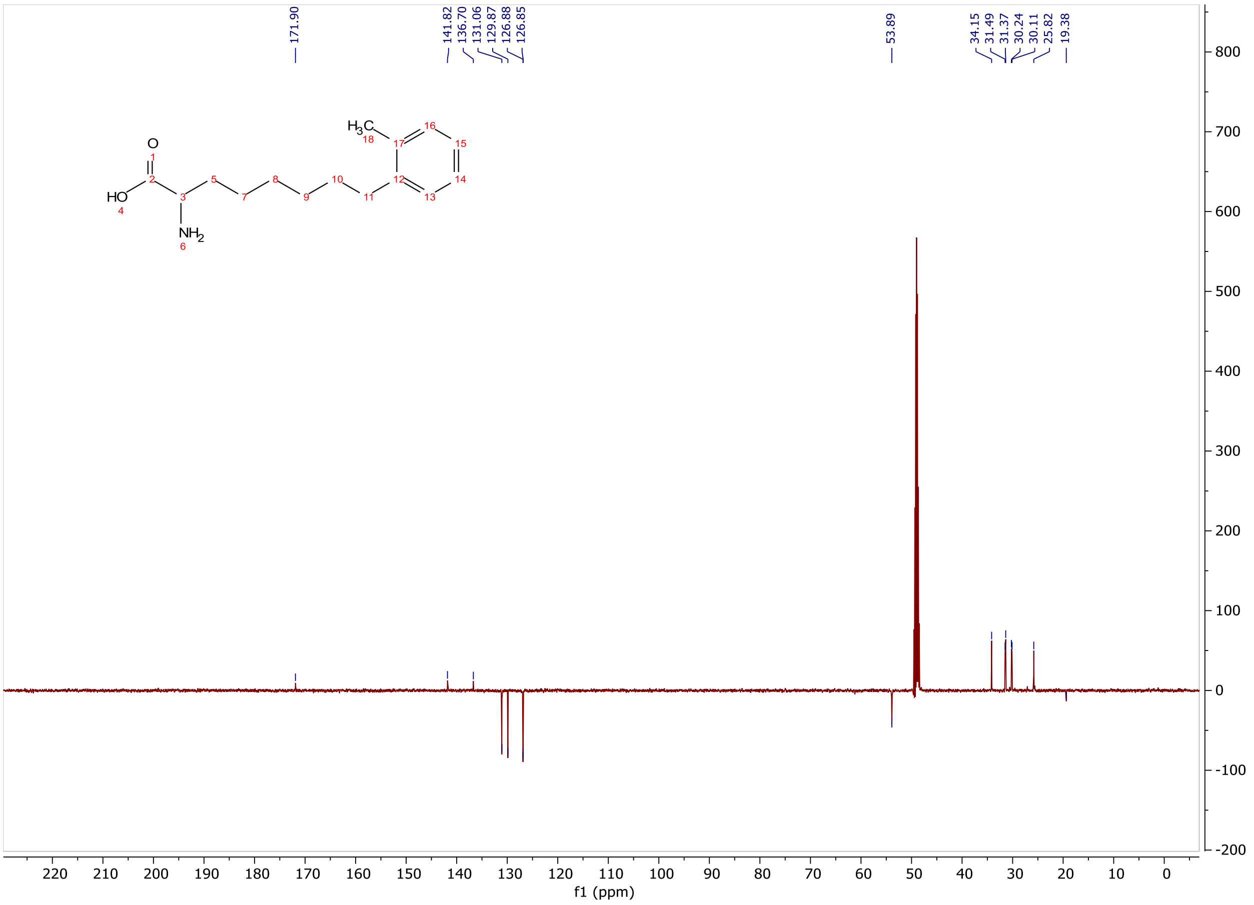

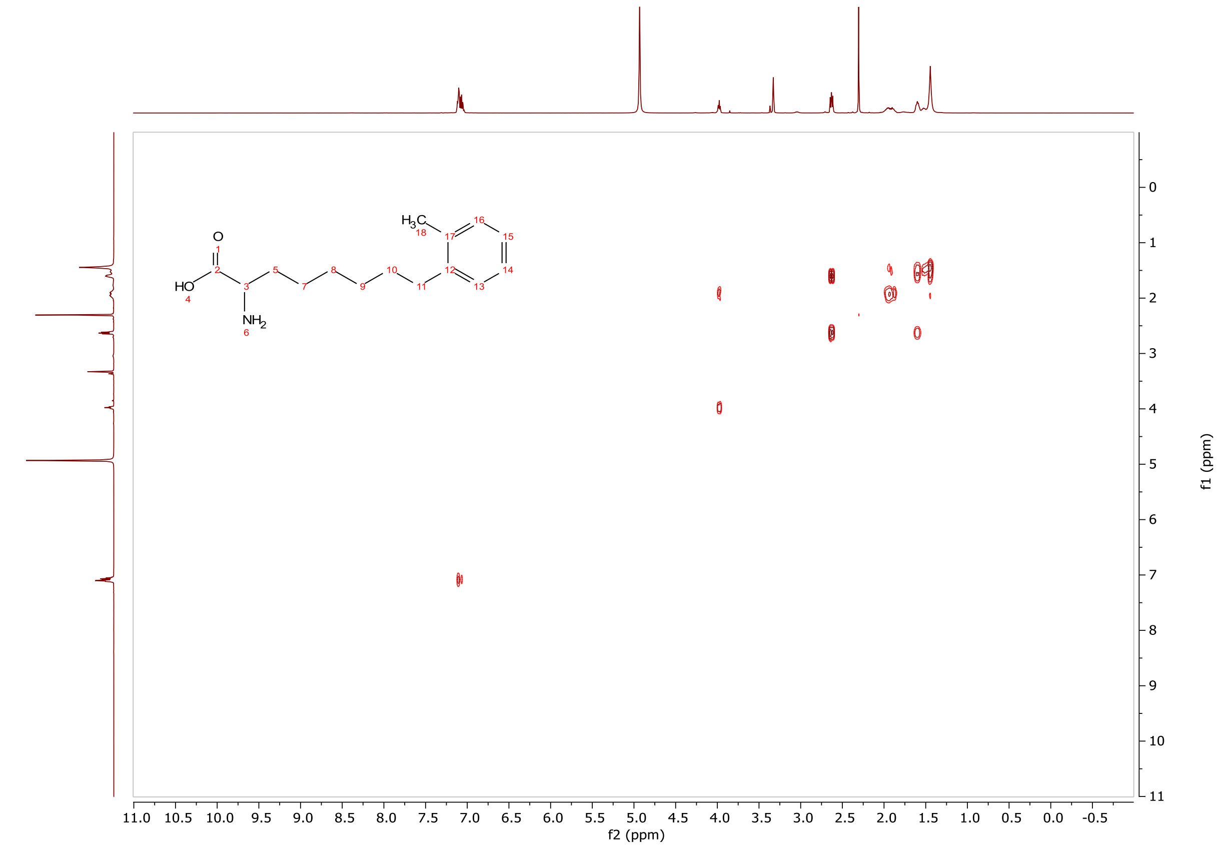

f2 (ppm)
f1 (ppm)

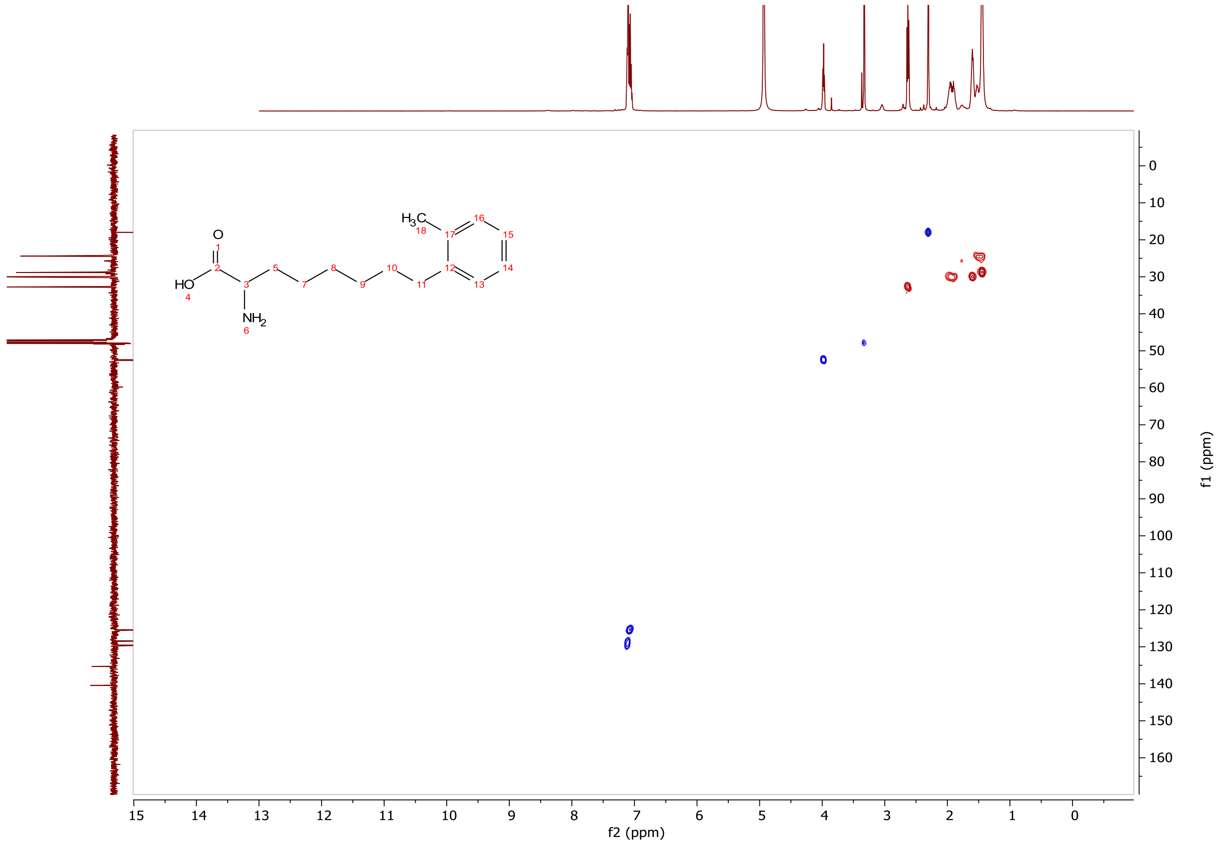

f2 (ppm)
f1 (ppm)

2-amino-8-phenyl-octanoic acid- 4e

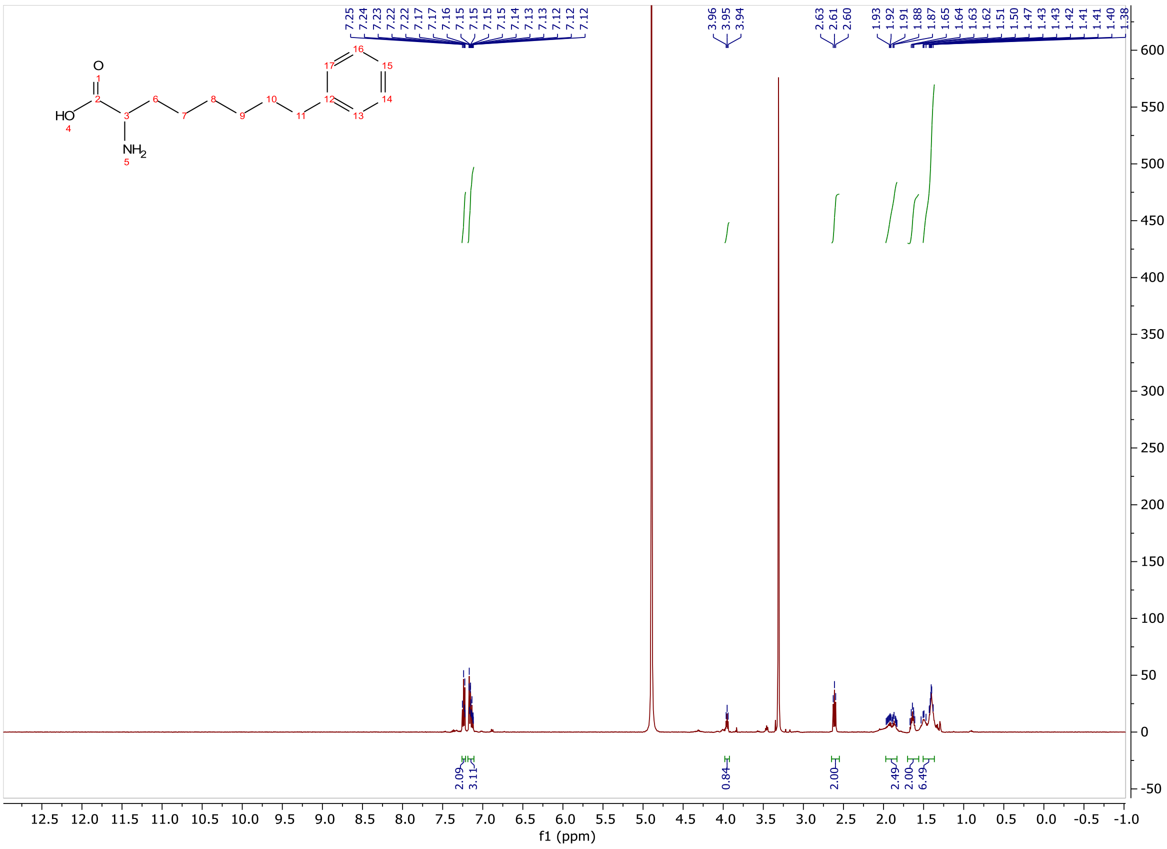

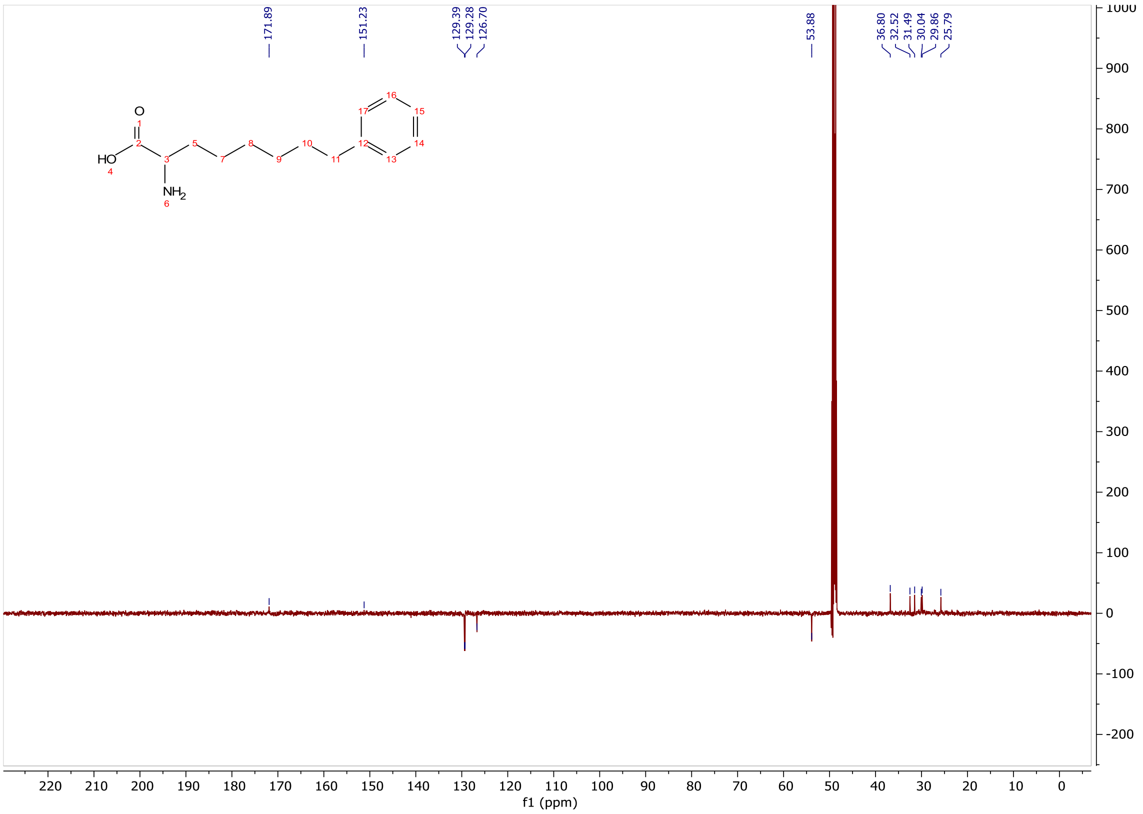

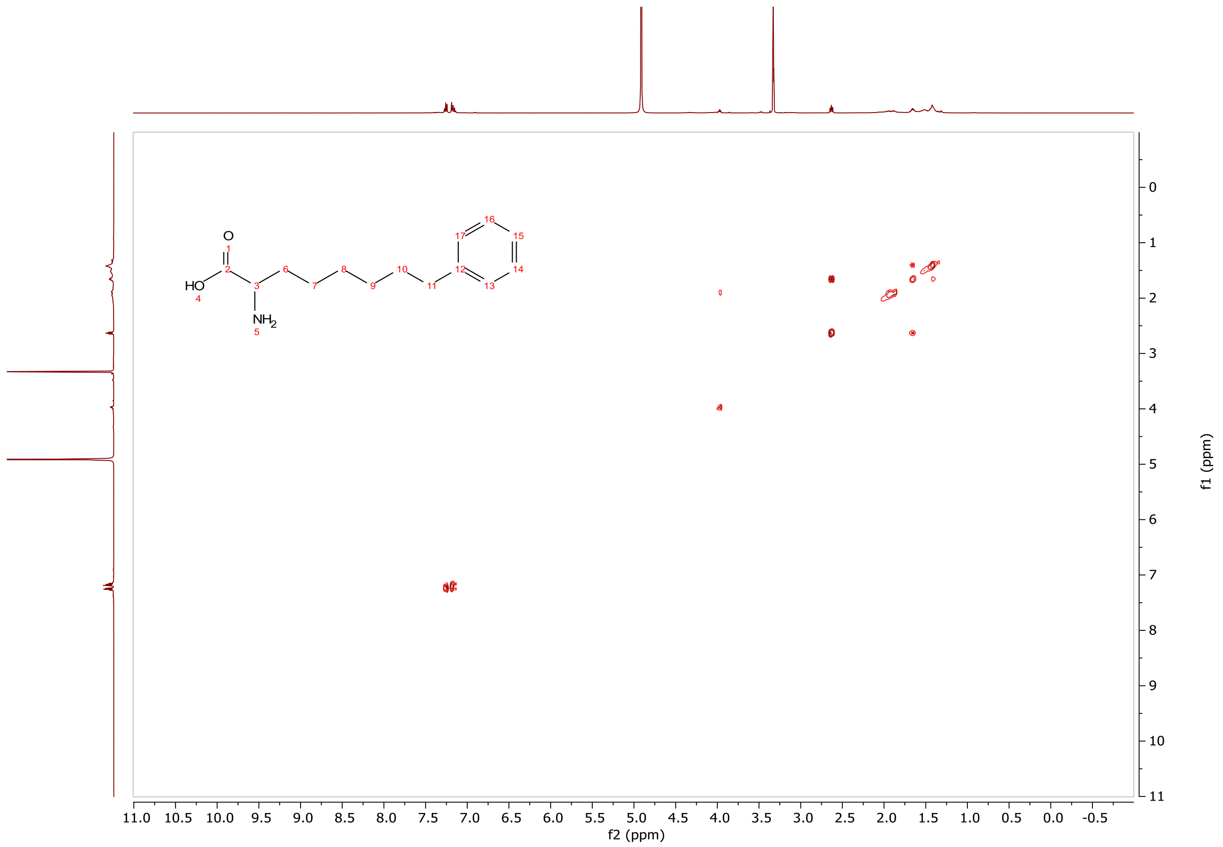

HO
NH2
O
f2 (ppm)
f1 (ppm)

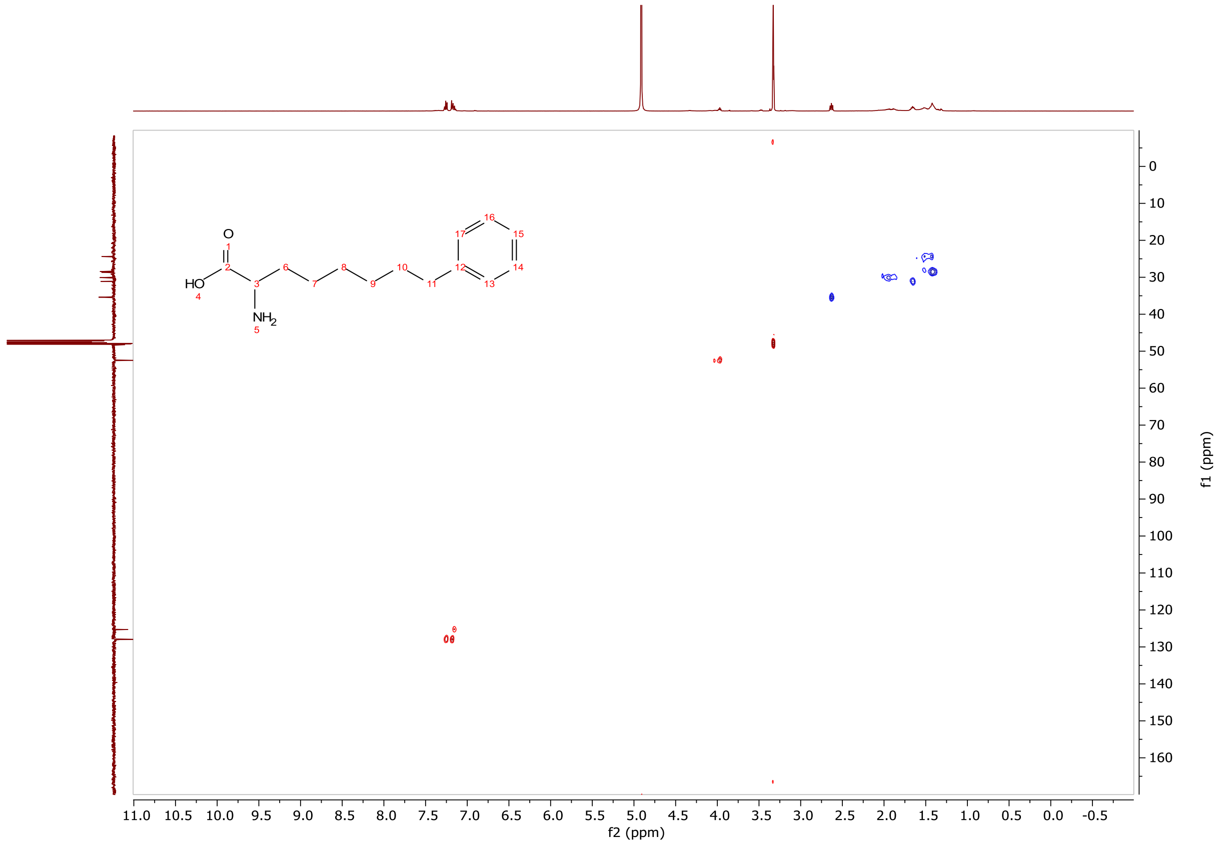

HO
NH2
O
f2 (ppm)
f1 (ppm)

2-amino-8-(4-chlorophenyl)octanoic acid- 4f

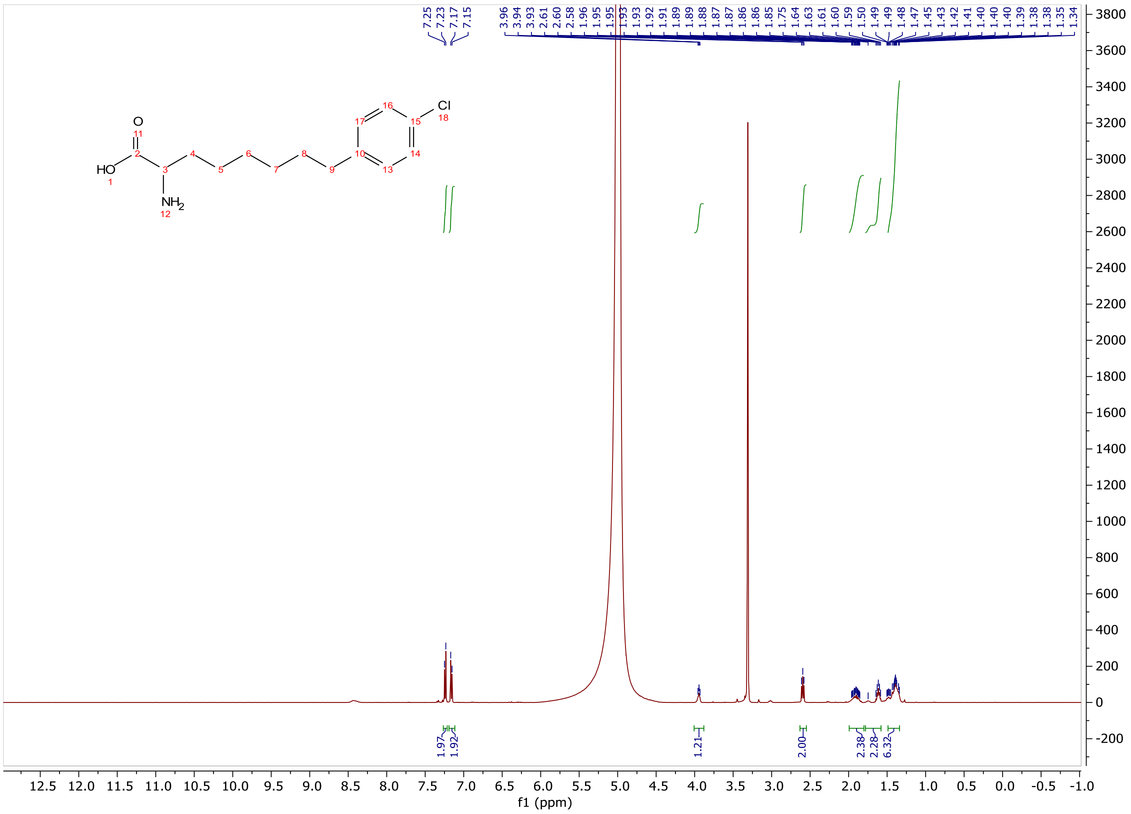

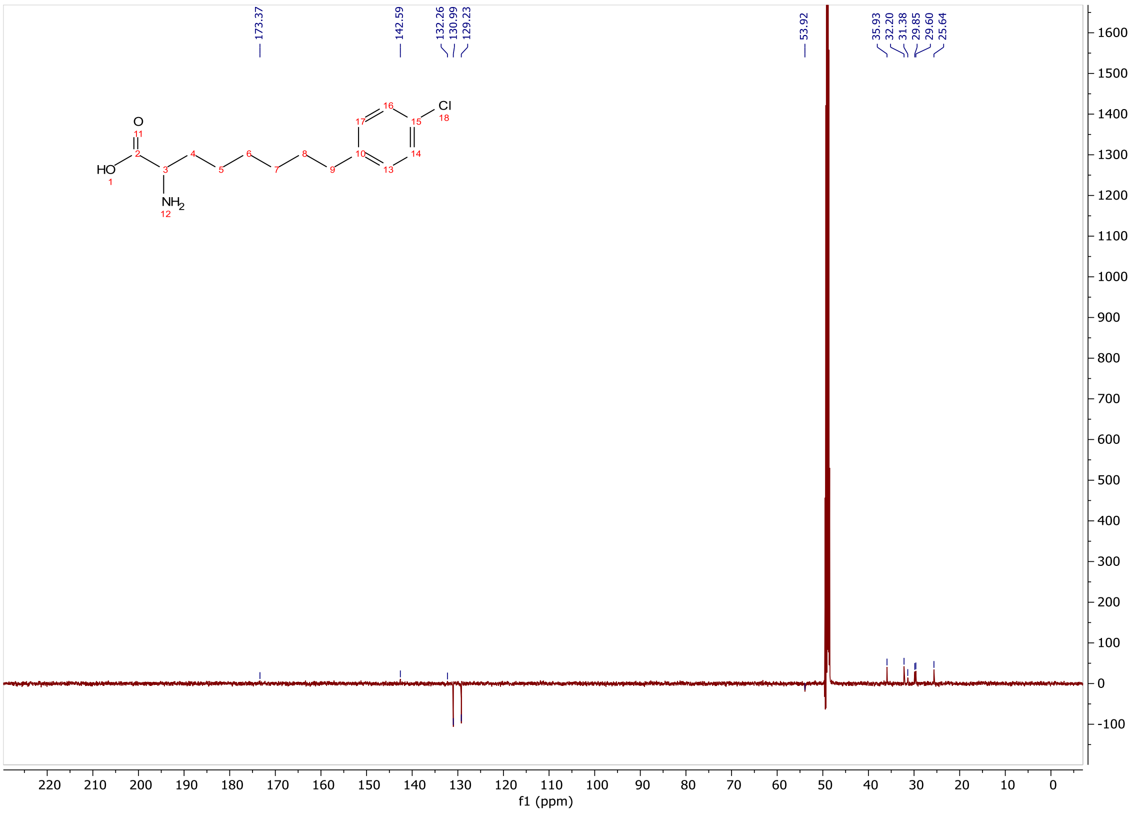

2-amino-8-(naphthalen-1-yl)octanoic acid- 4g

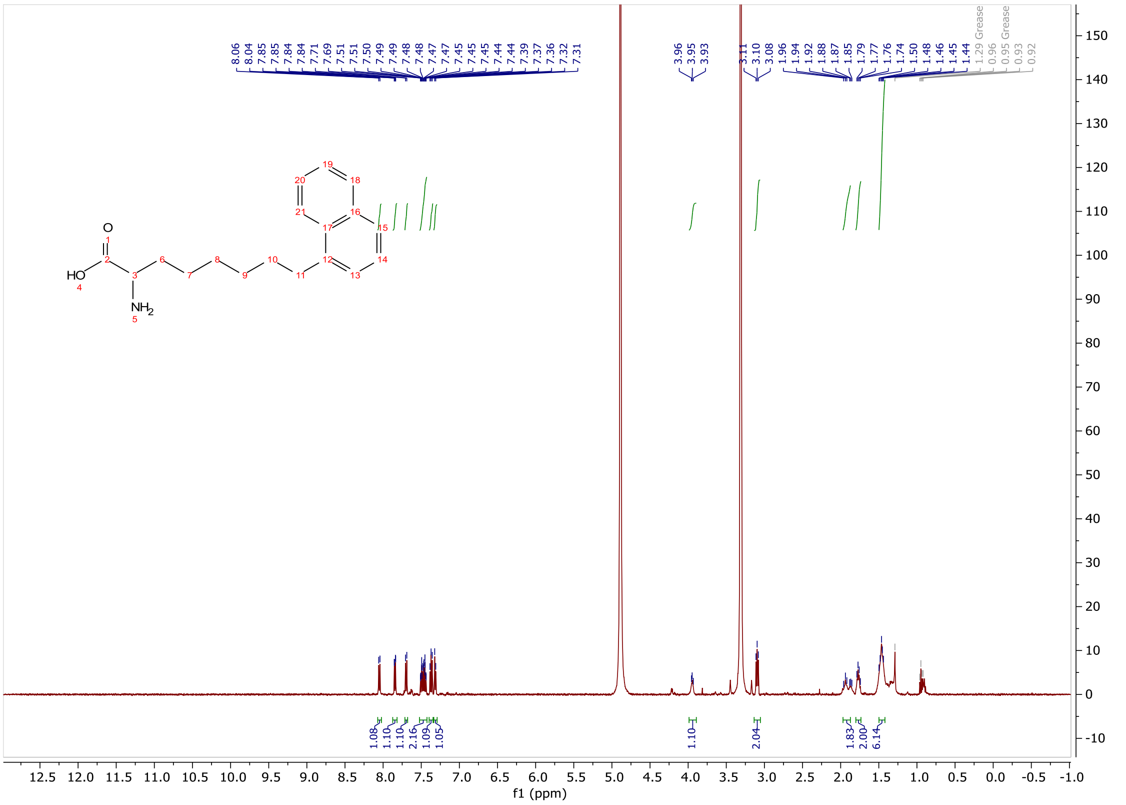

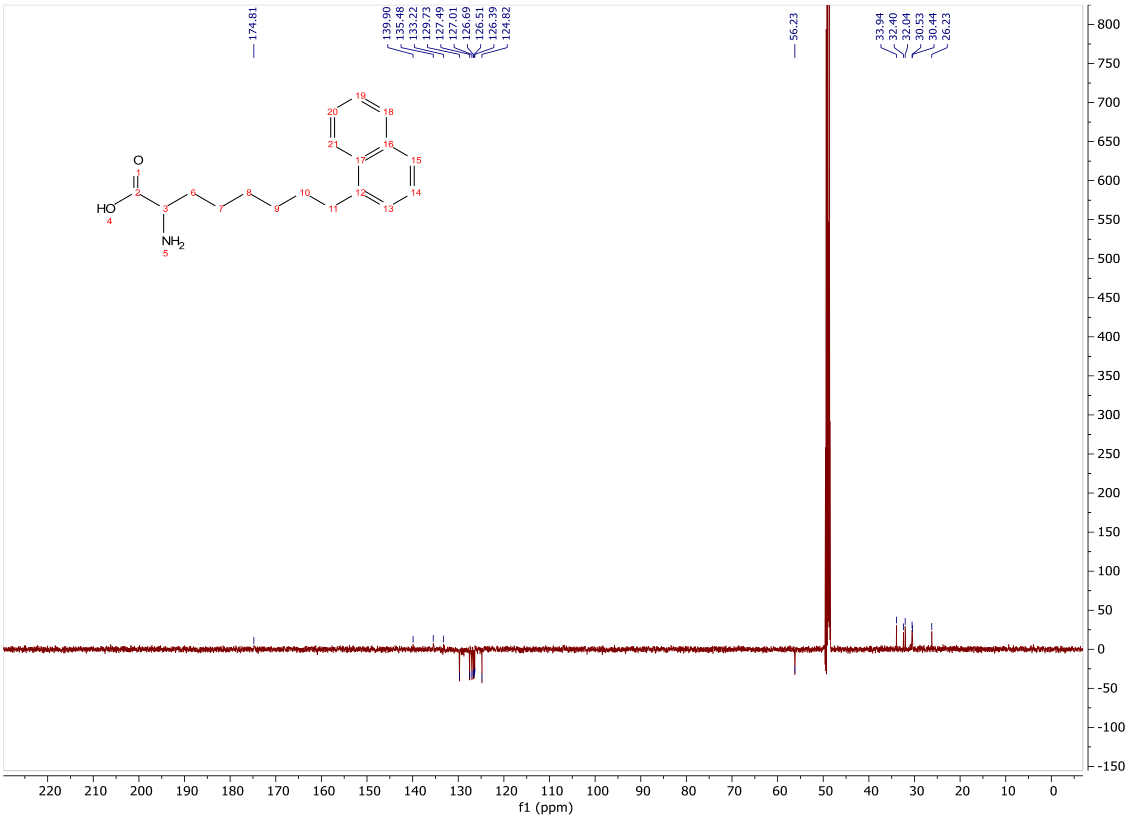

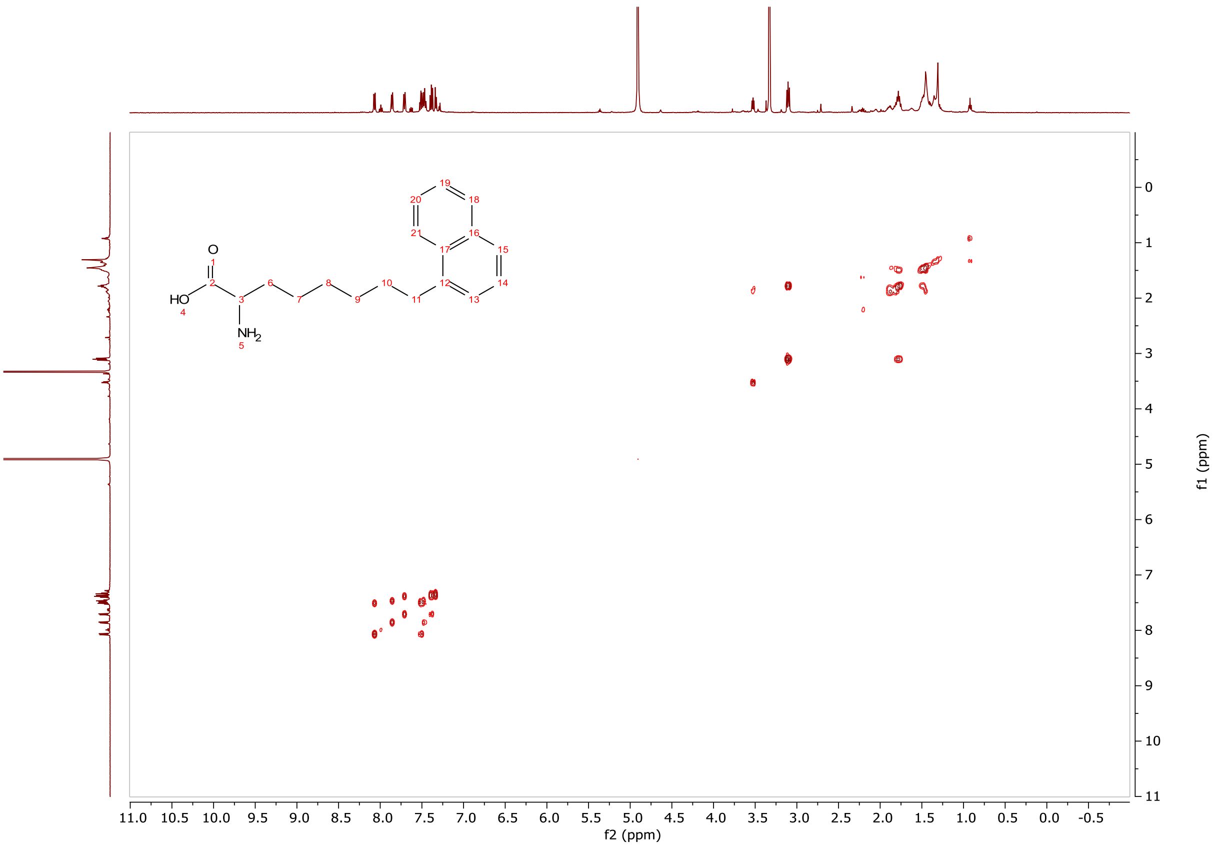

f2 (ppm)
f1 (ppm)

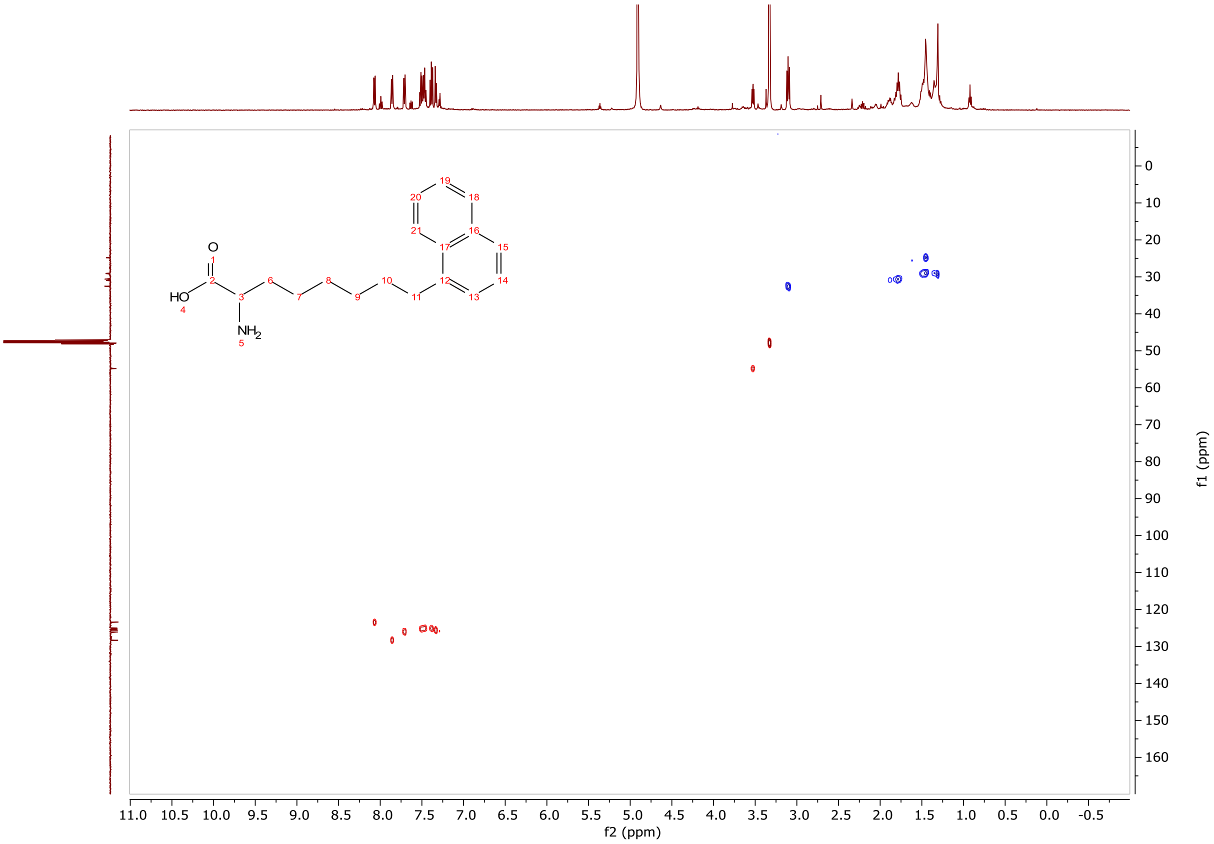

f2 (ppm)
f1 (ppm)

2-amino-8-(4-aminophenyl)octanoic acid- 4h

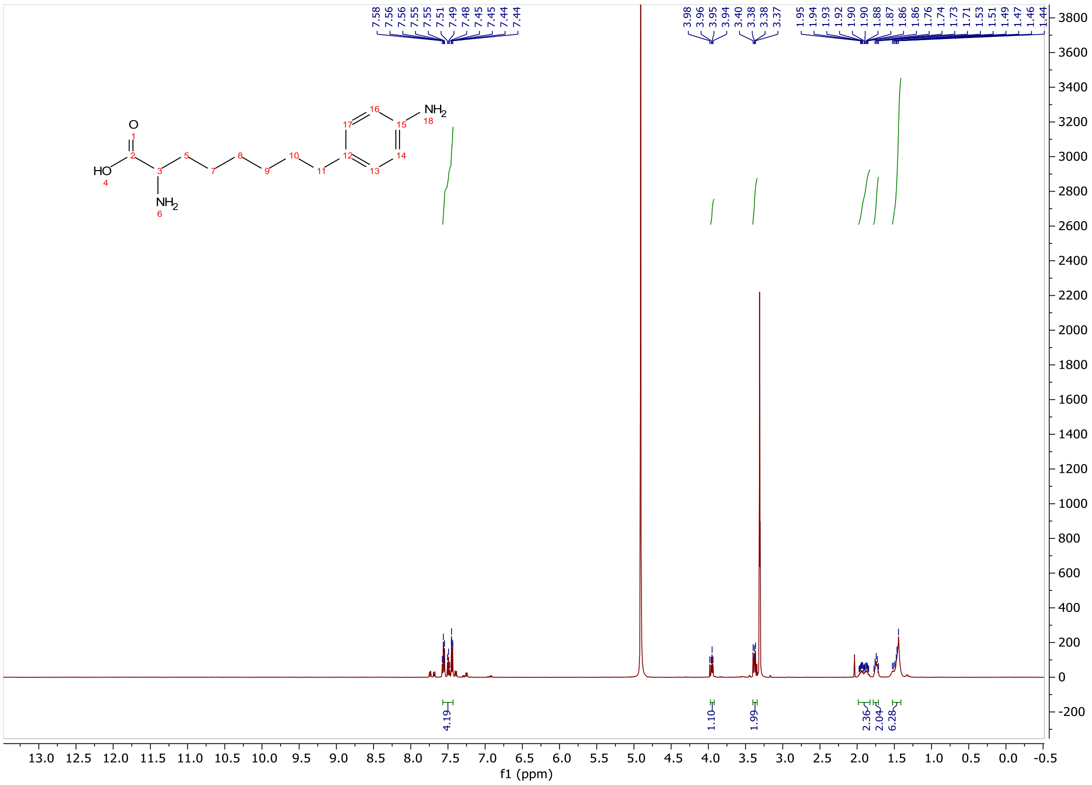

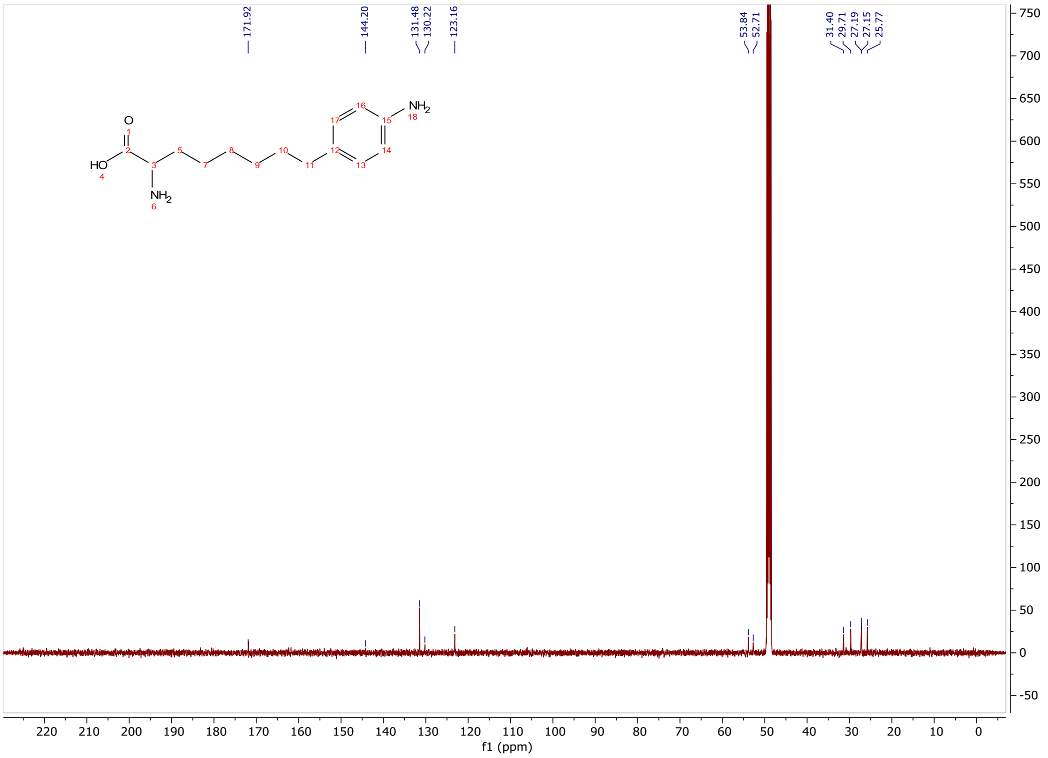

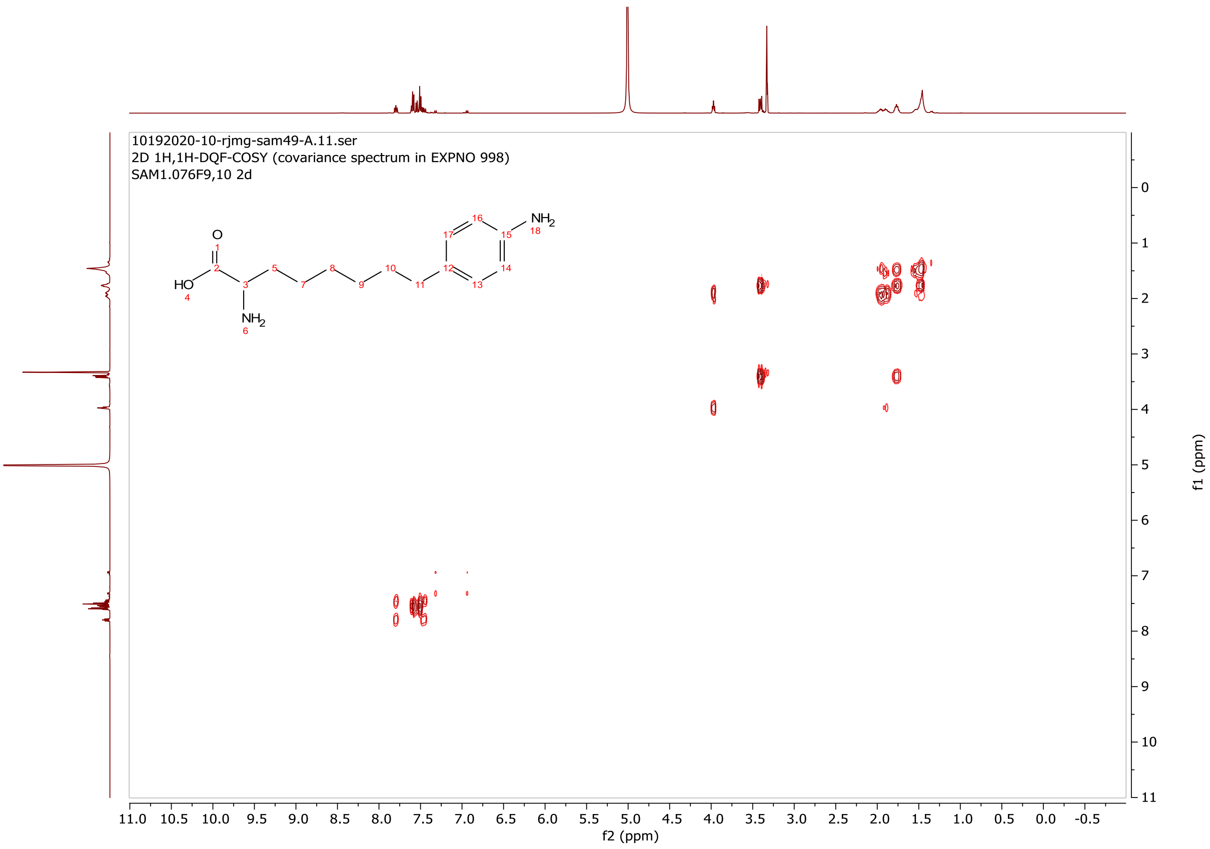
10192020-10-rjmg-sam49-A.11.ser
2D 1H,1H-DQF-COSY (covariance spectrum in EXPNO 998)
SAM1.076F9,10 2d
f2 (ppm)
f1 (ppm)

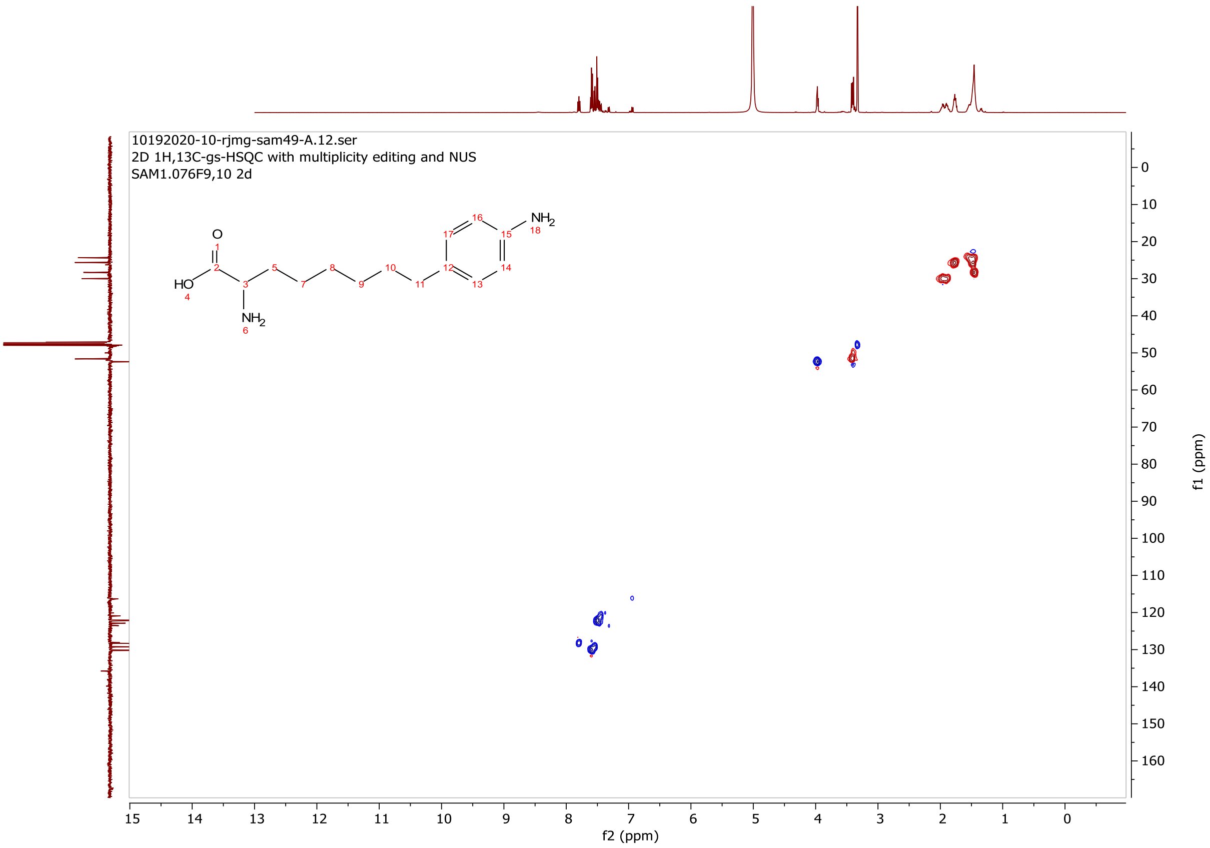
10192020-10-rjmg-sam49-A.12.ser
2D 1H,13C-gs-HSQC with multiplicity editing and NUS
SAM1.076F9,10 2d
f2 (ppm)
f1 (ppm)

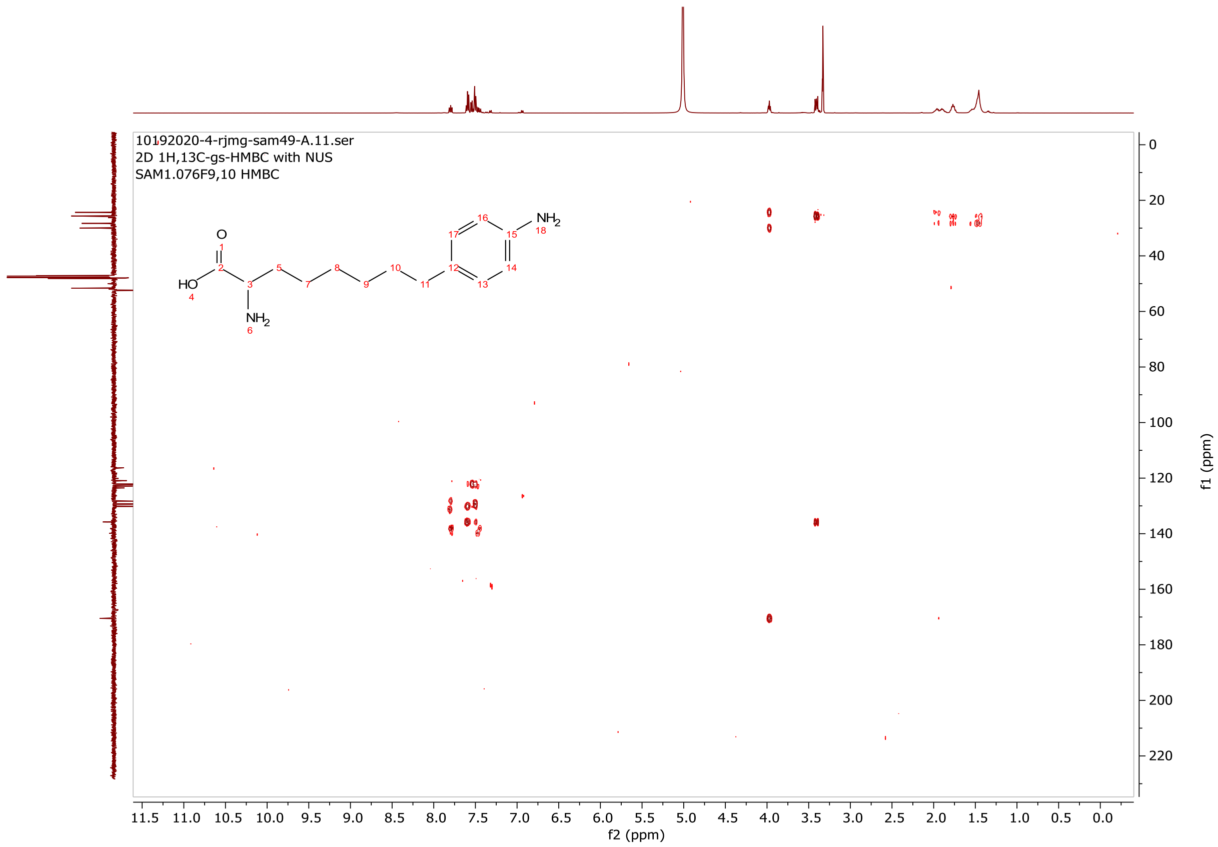
10192020-4-rjmg-sam49-A.11.ser
2D 1H,13C-gs-HMBC with NUS
SAM1.076F9,10 HMBC
f2 (ppm)
f1 (ppm)

±*4*-(7-((tert-butoxycarbonyl)amino)-7-carboxyheptyl)benzoic acid-*N*-Boc 4i

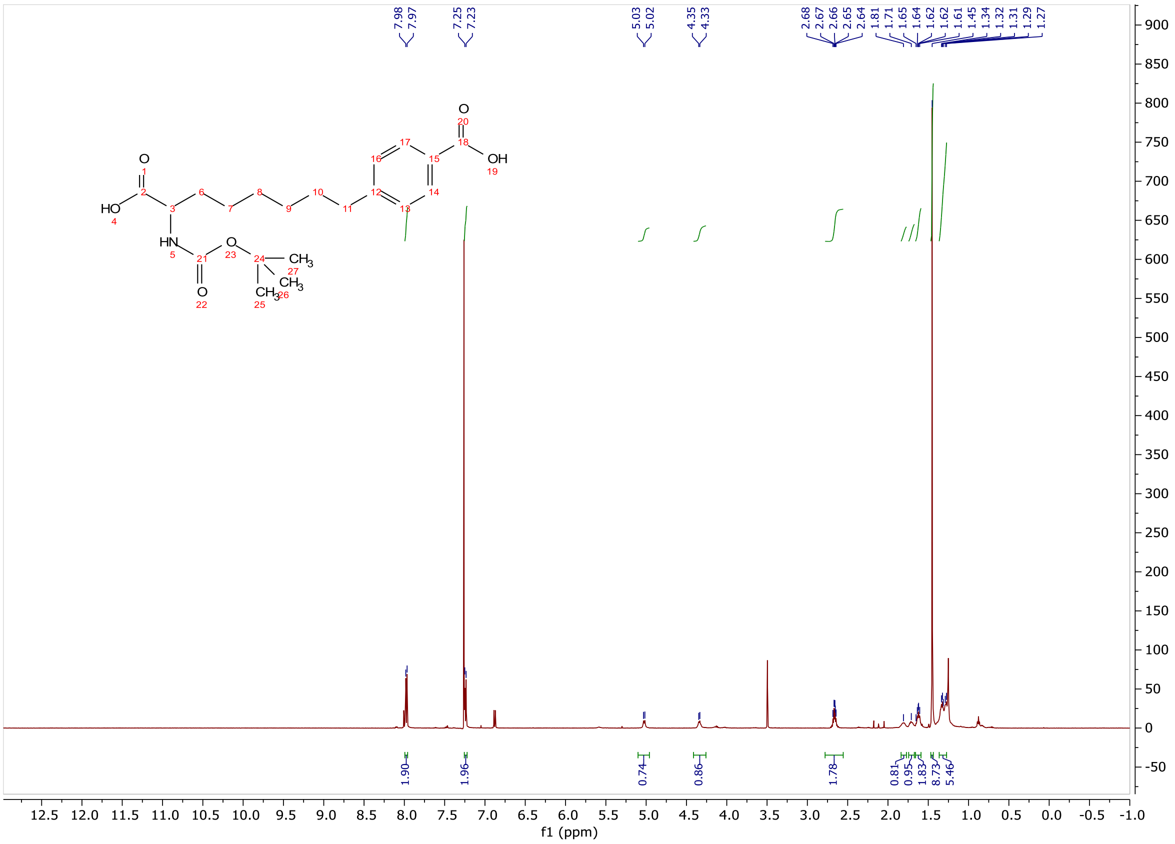

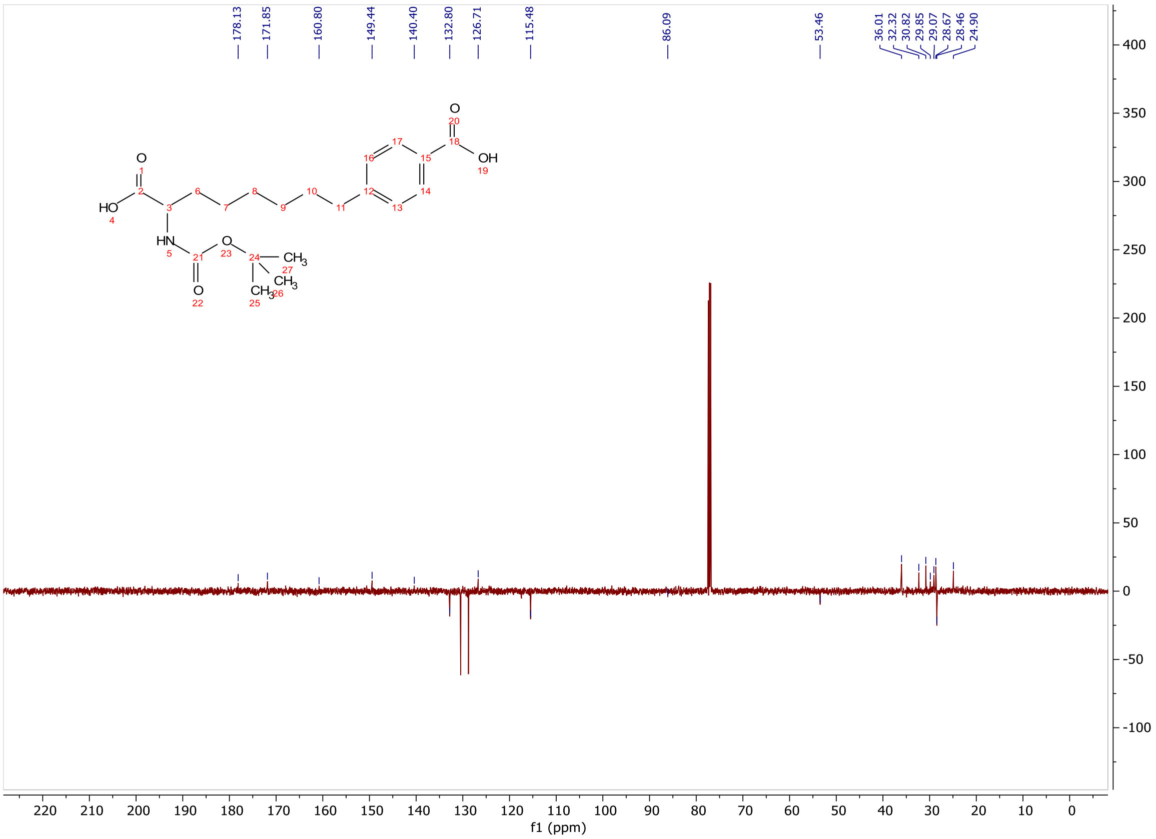

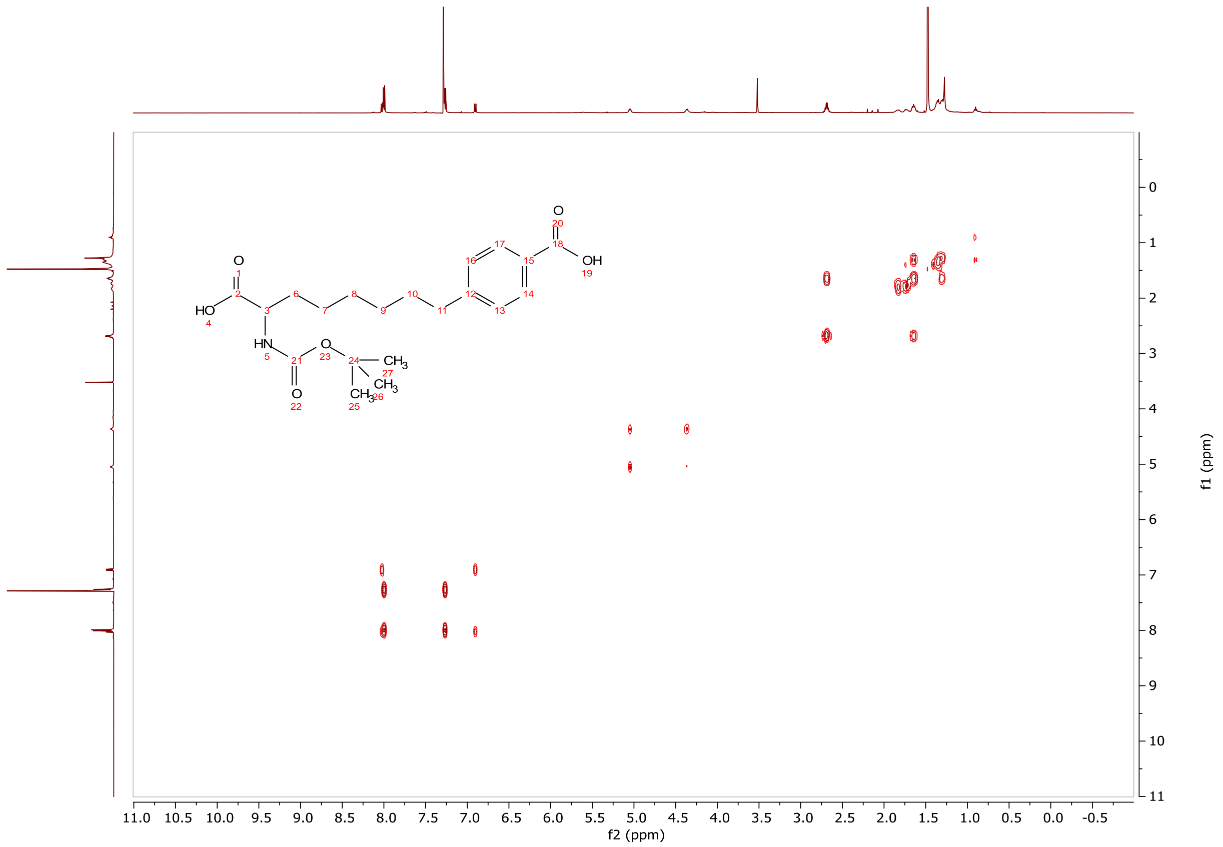
f2 (ppm)
f1 (ppm)

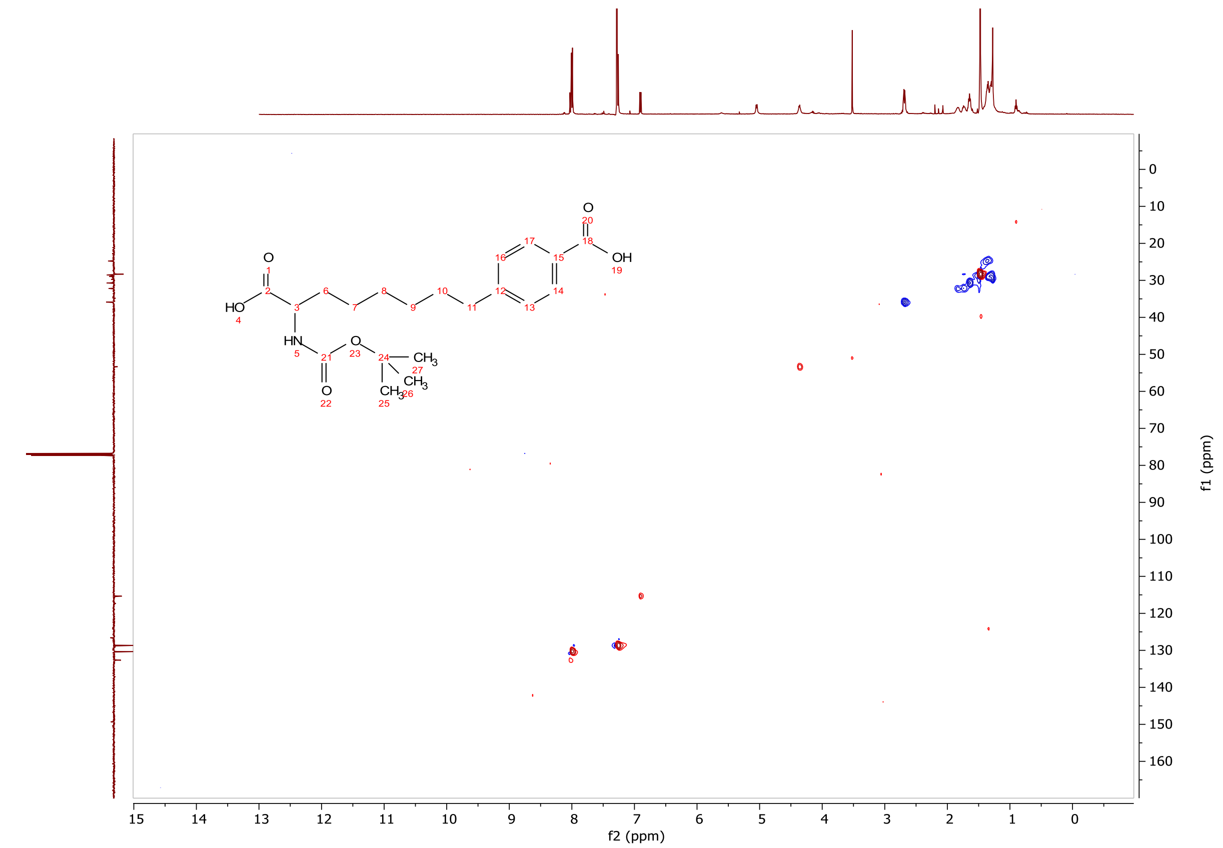
f2 (ppm)
f1 (ppm)

4-(7-amino-7-carboxyheptyl)benzoic acid- 4i

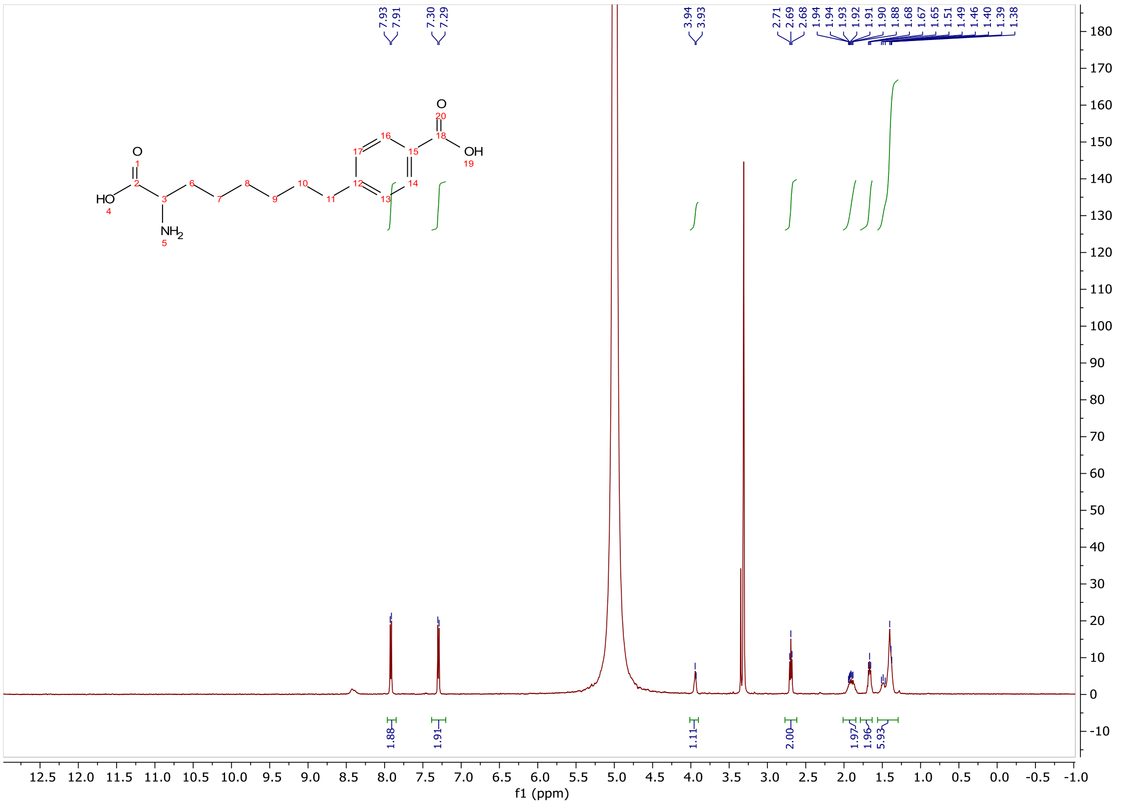

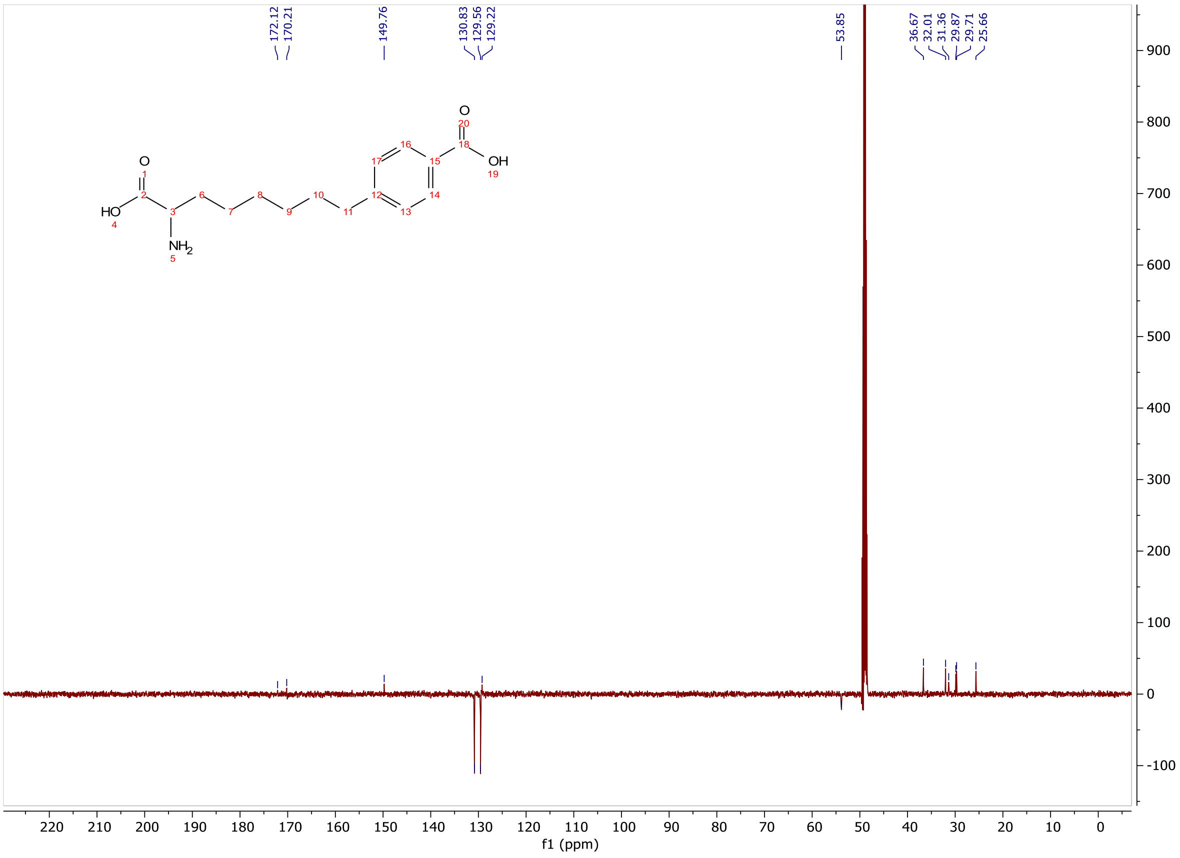

2-amino-8-(3-aminophenyl)octanoic acid- 4j

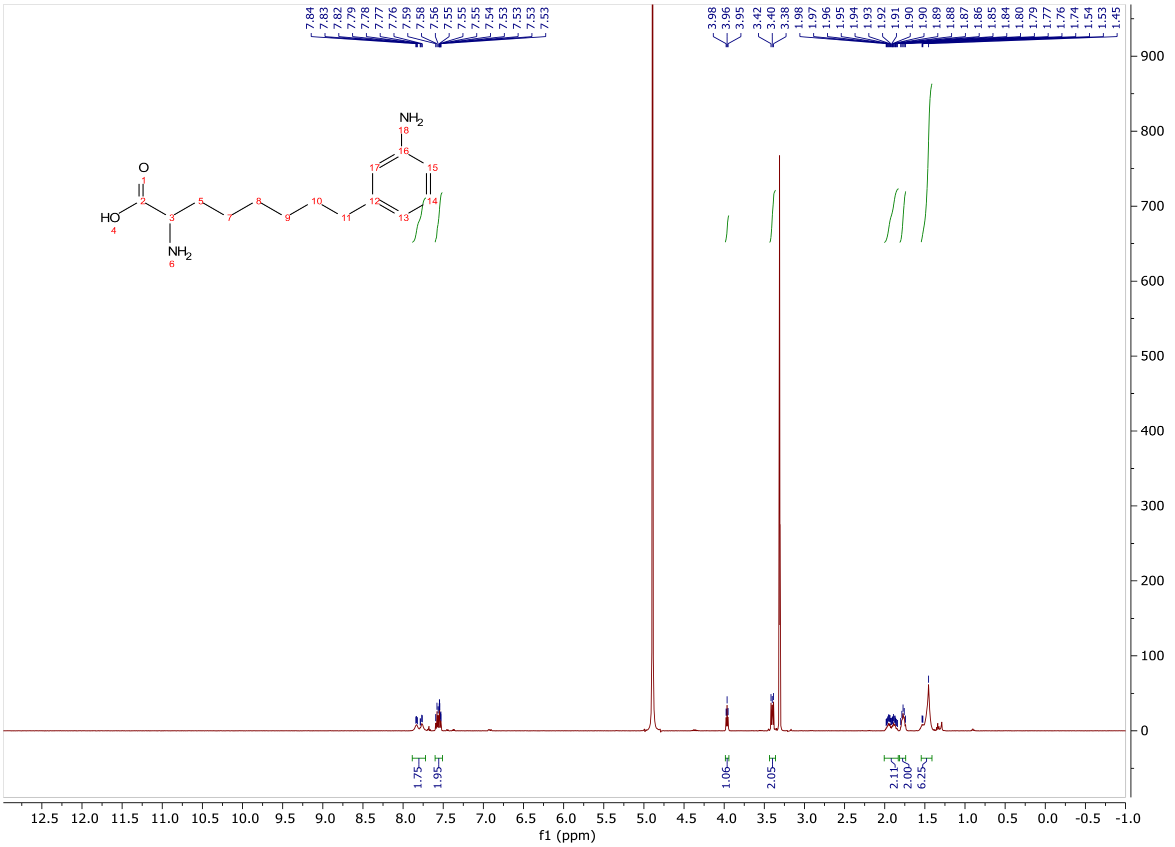

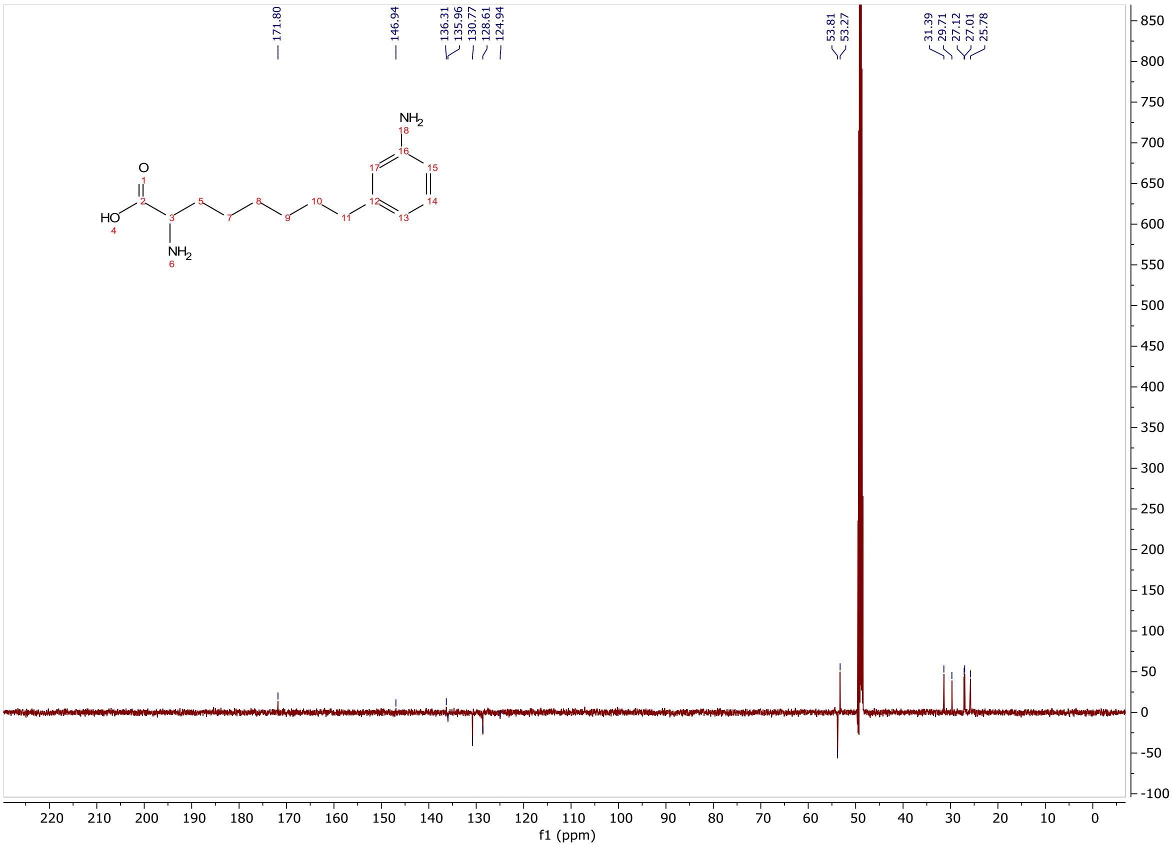

2-amino-8-(σ-fluorophenyl)-octanoic acid- 4k

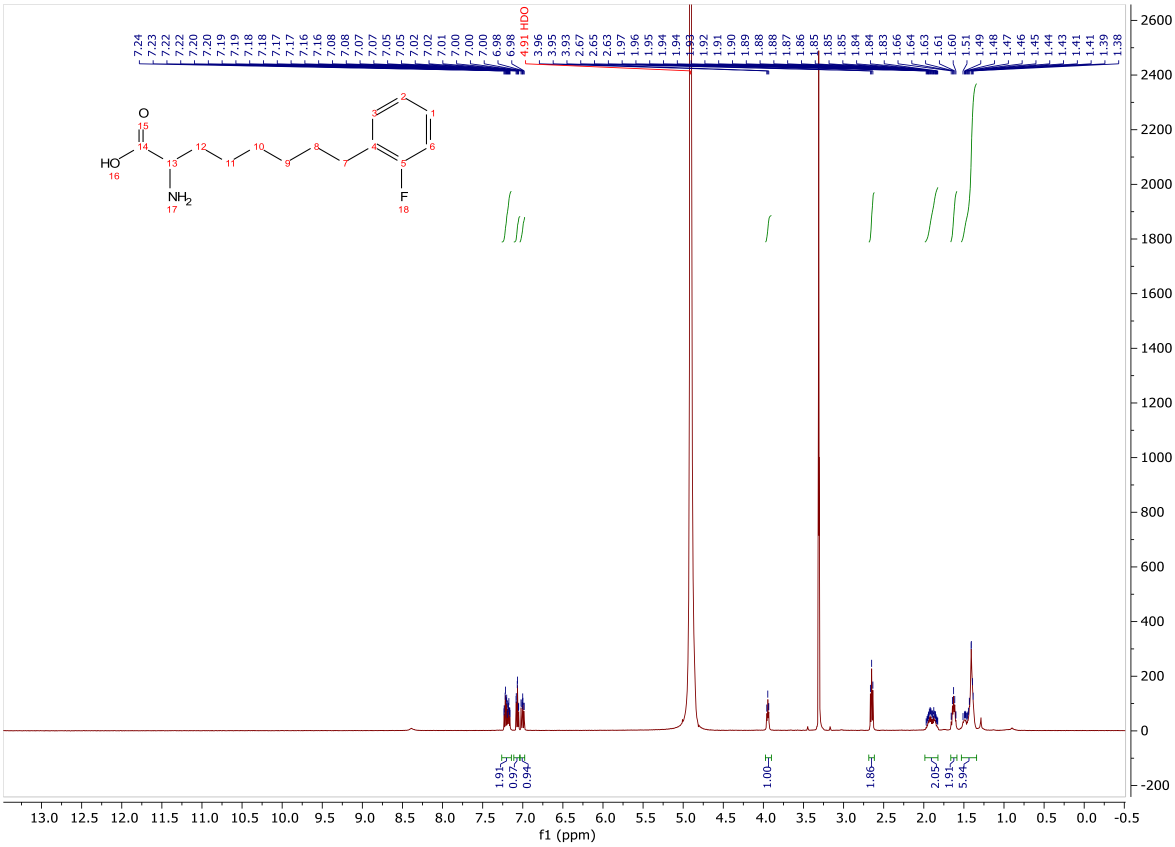

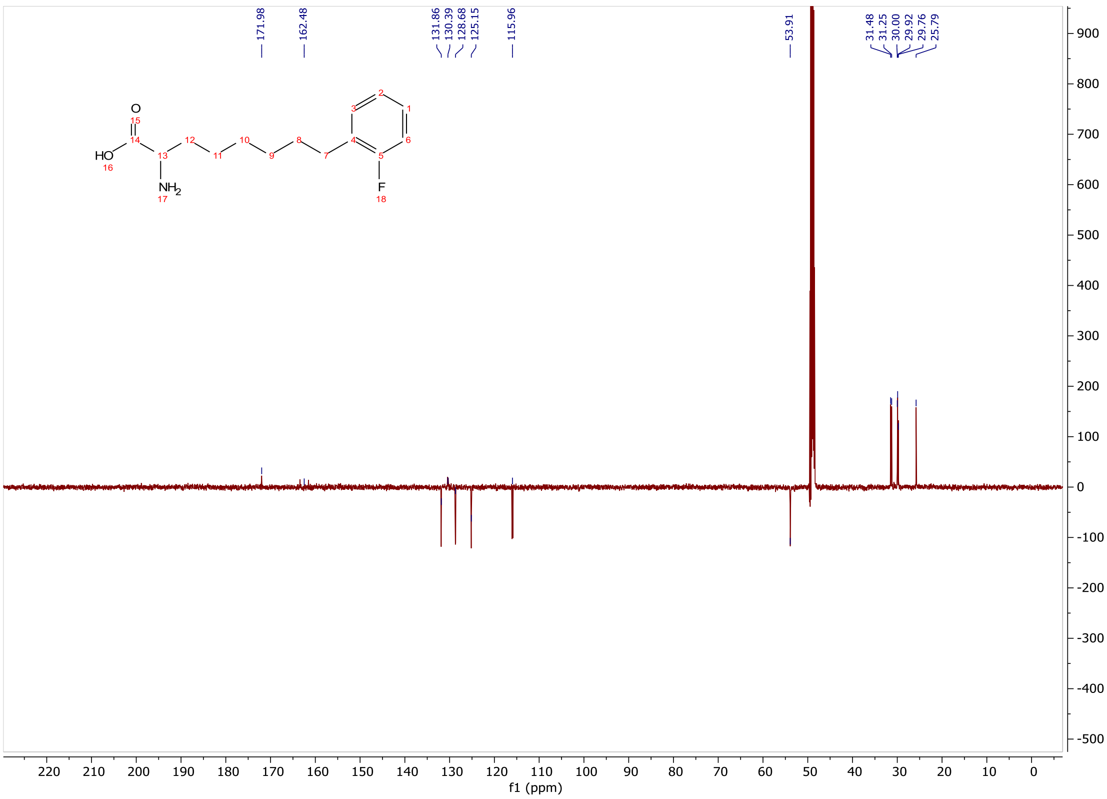

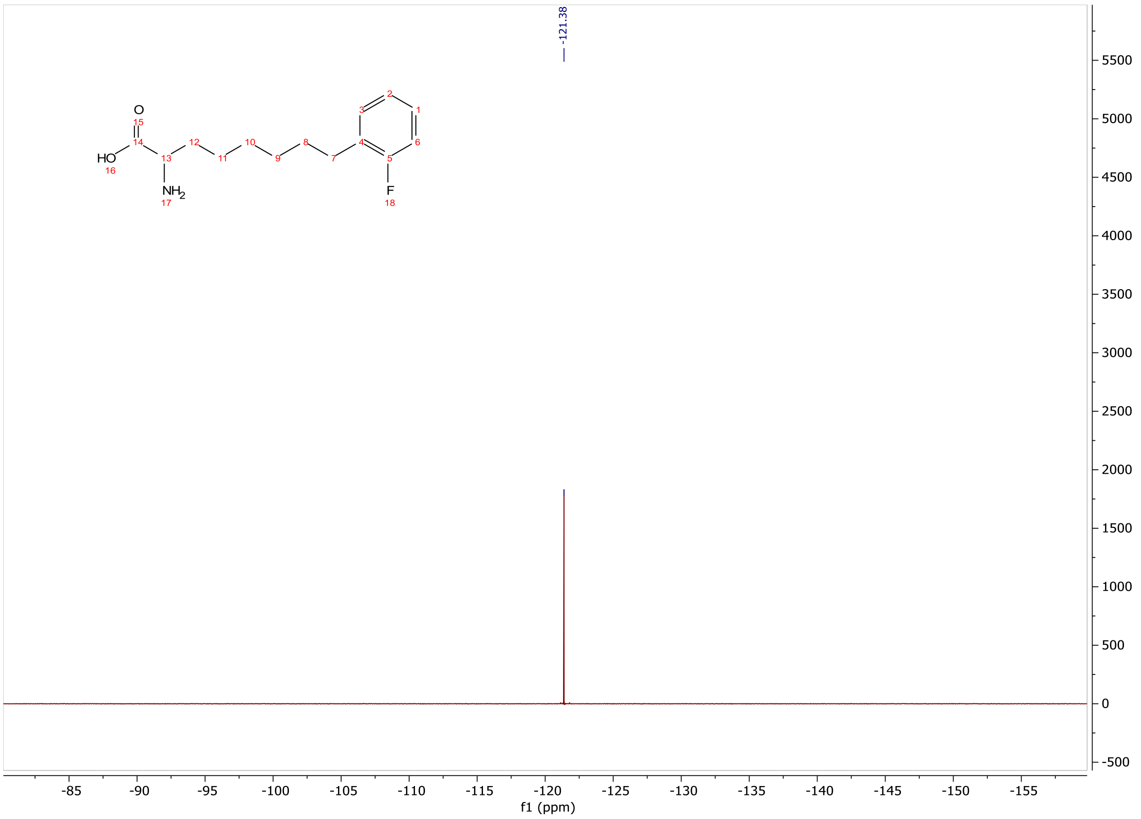

-121.38
O
15
14
HO
16
13
NH2
17
12
11
10
9
8
7
4
3
2
1
6
5
F
18
5500
5000
4500
4000
3500
3000
2500
2000
1500
1000
500
0
-500
-85 -90 -95 -100 -105 -110 -115 -120 -125 -130 -135 -140 -145 -150 -155
f1 (ppm)

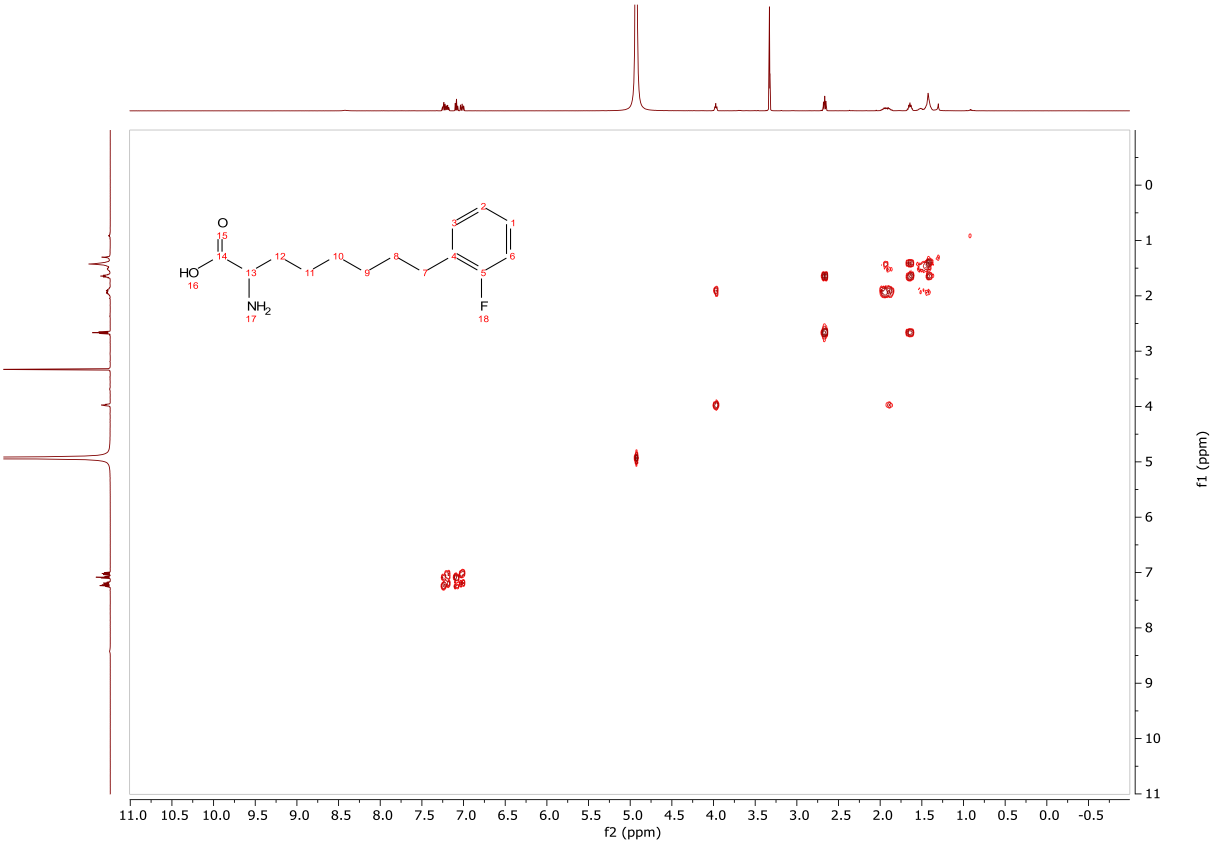

O
15
14
HO
16
13
NH2
17
12
11
10
9
8
7
4
3
2
1
6
5
F
18
0 1 2 3 4 5 6 7 8 9 10 11
f1 (ppm)
11.0 10.5 10.0 9.5 9.0 8.5 8.0 7.5 7.0 6.5 6.0 5.5 5.0 4.5 4.0 3.5 3.0 2.5 2.0 1.5 1.0 0.5 0.0 -0.5
f2 (ppm)

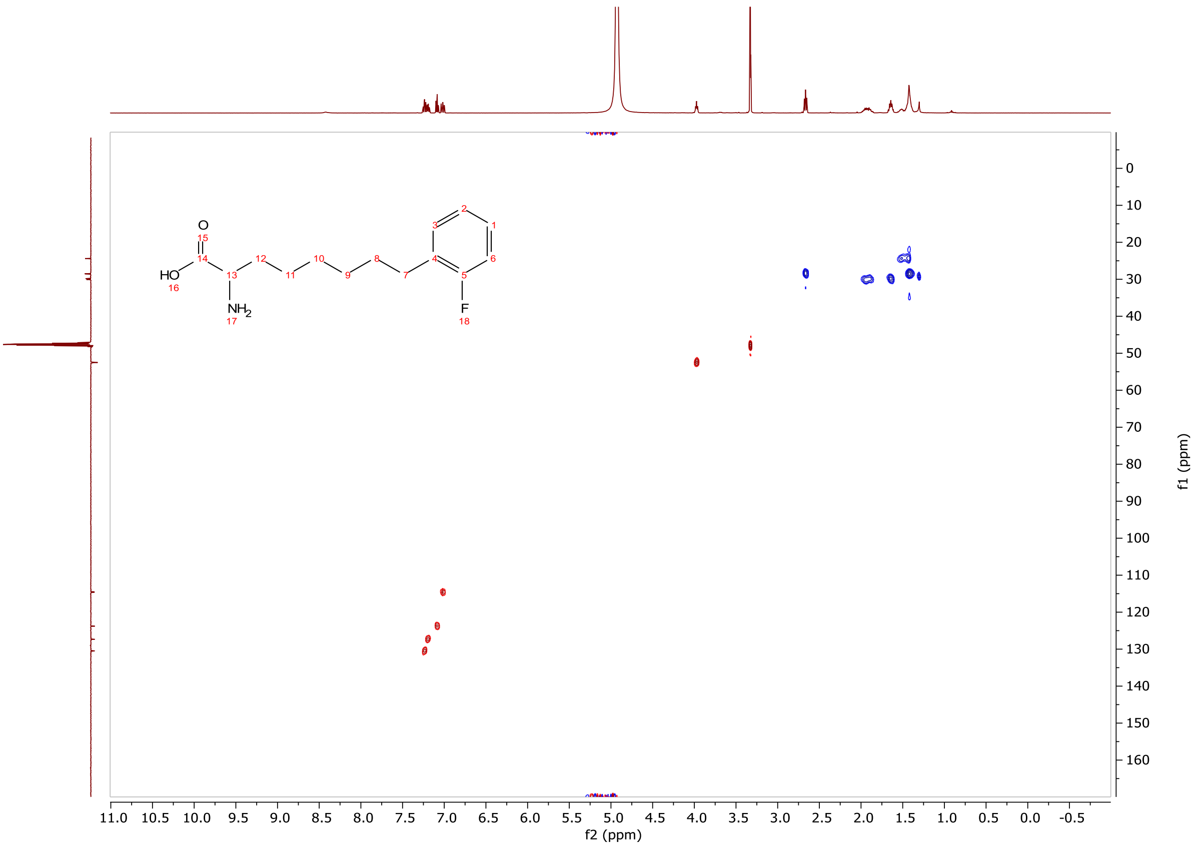

f2 (ppm)
f1 (ppm)
HO
O
NH2
F

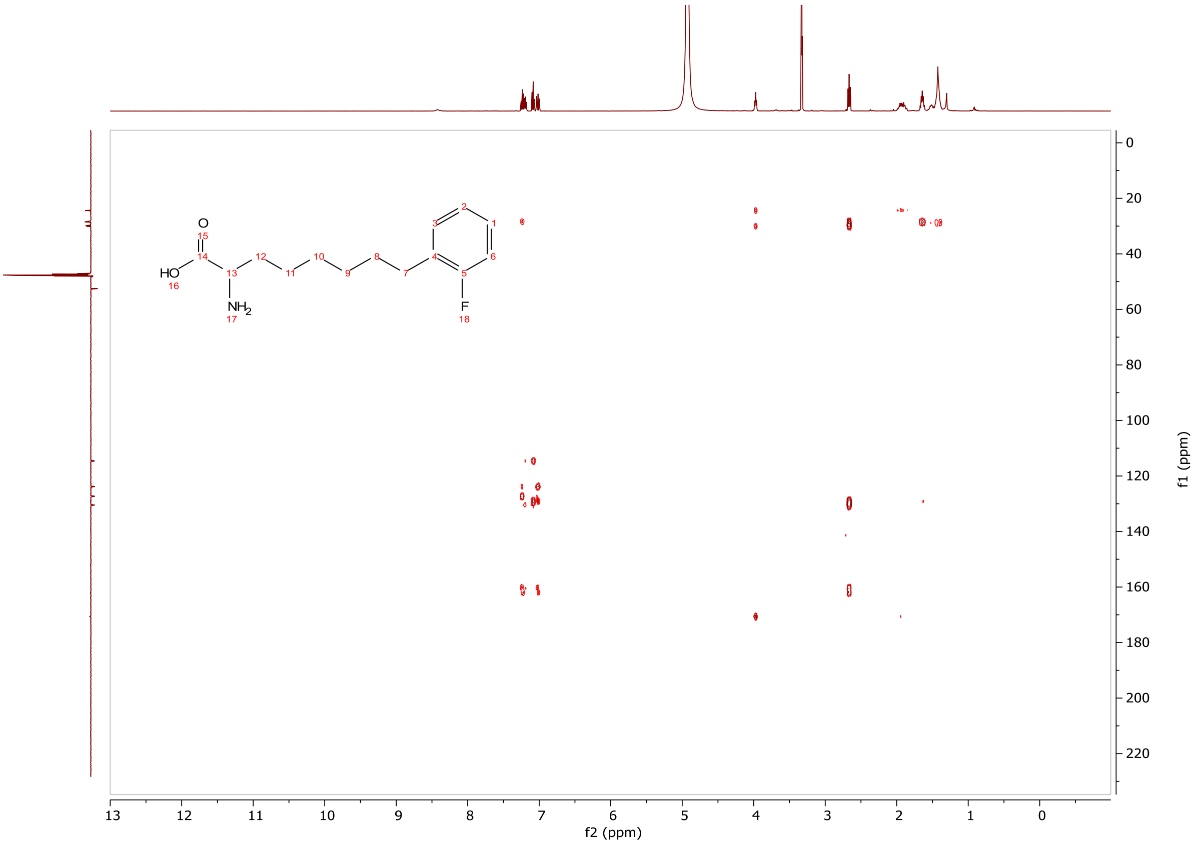

f2 (ppm)
f1 (ppm)
HO
O
NH2
F

2-amino-8-(3-((5-(dimethylamino)naphthalene)-1-sulfonamido)phenyl)octanoic acid- 4m

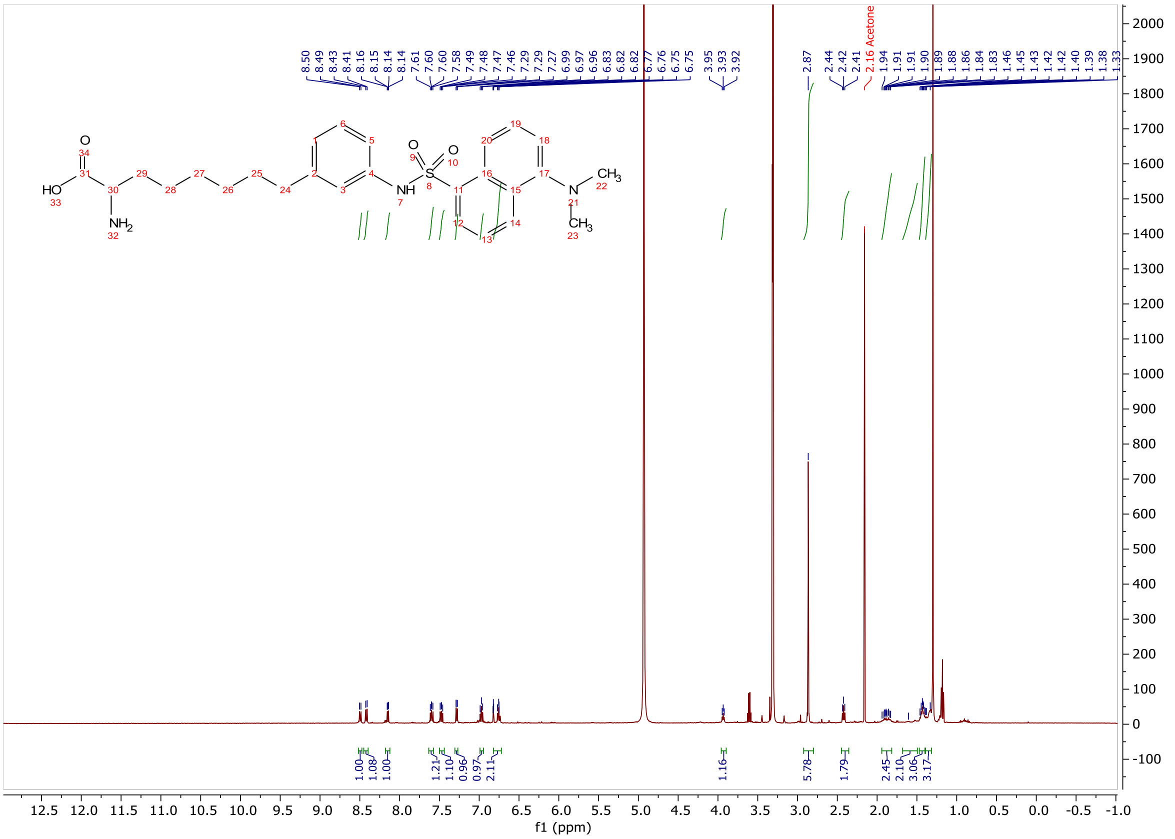

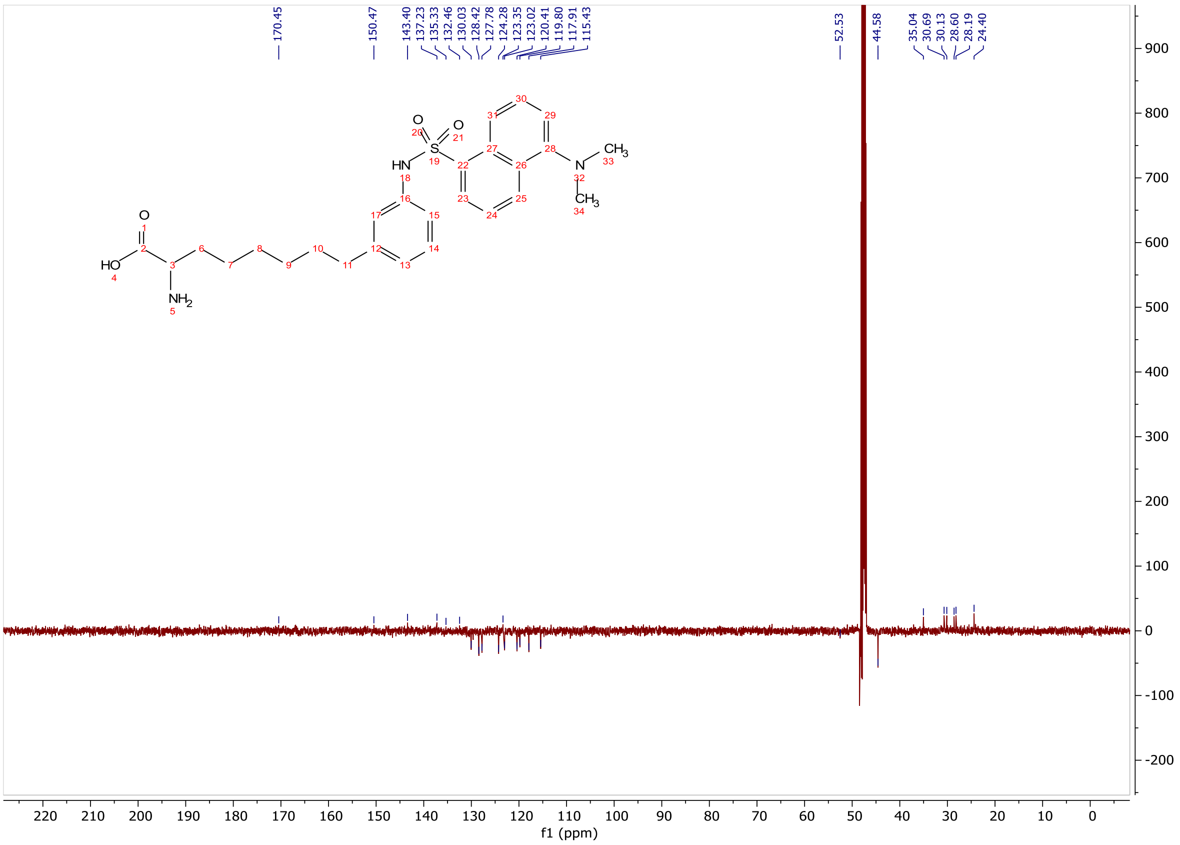

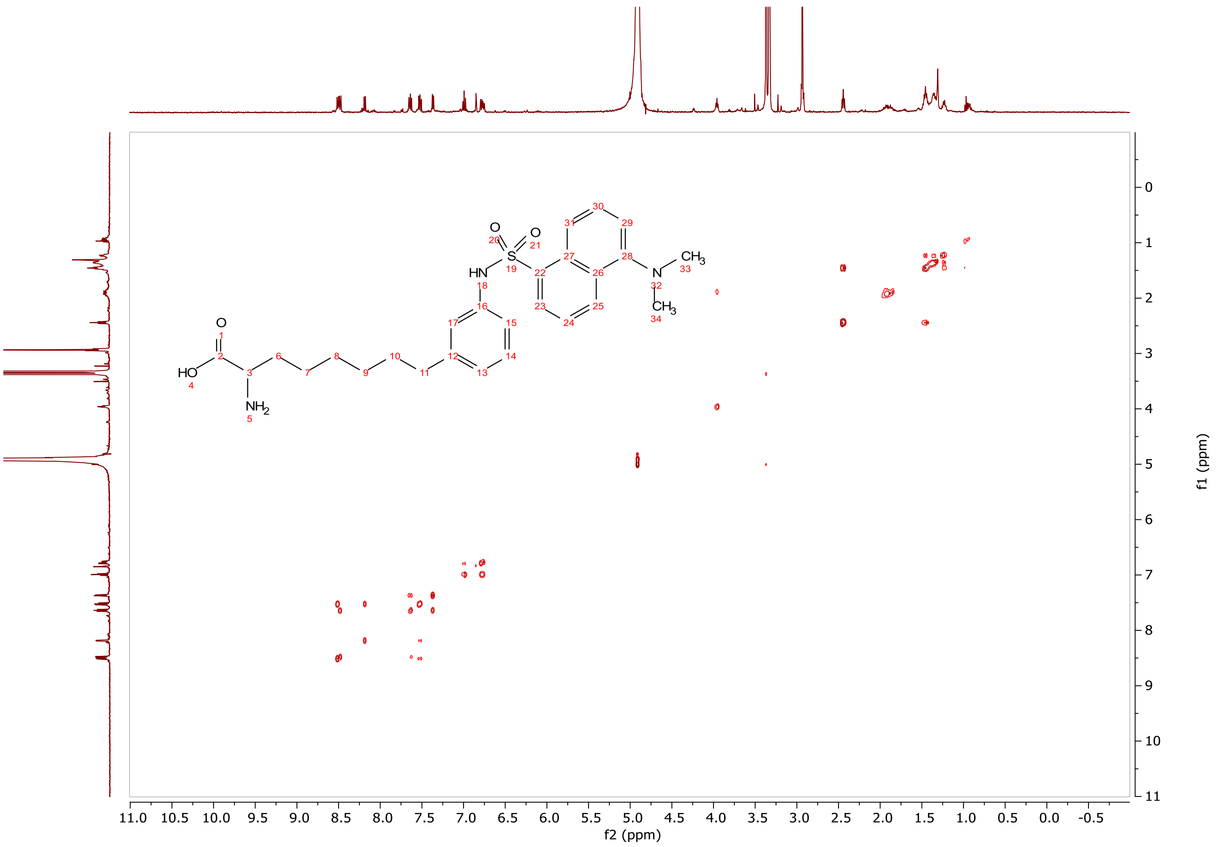
f2 (ppm)
f1 (ppm)
HO
NH2
HN
CH3
CH3

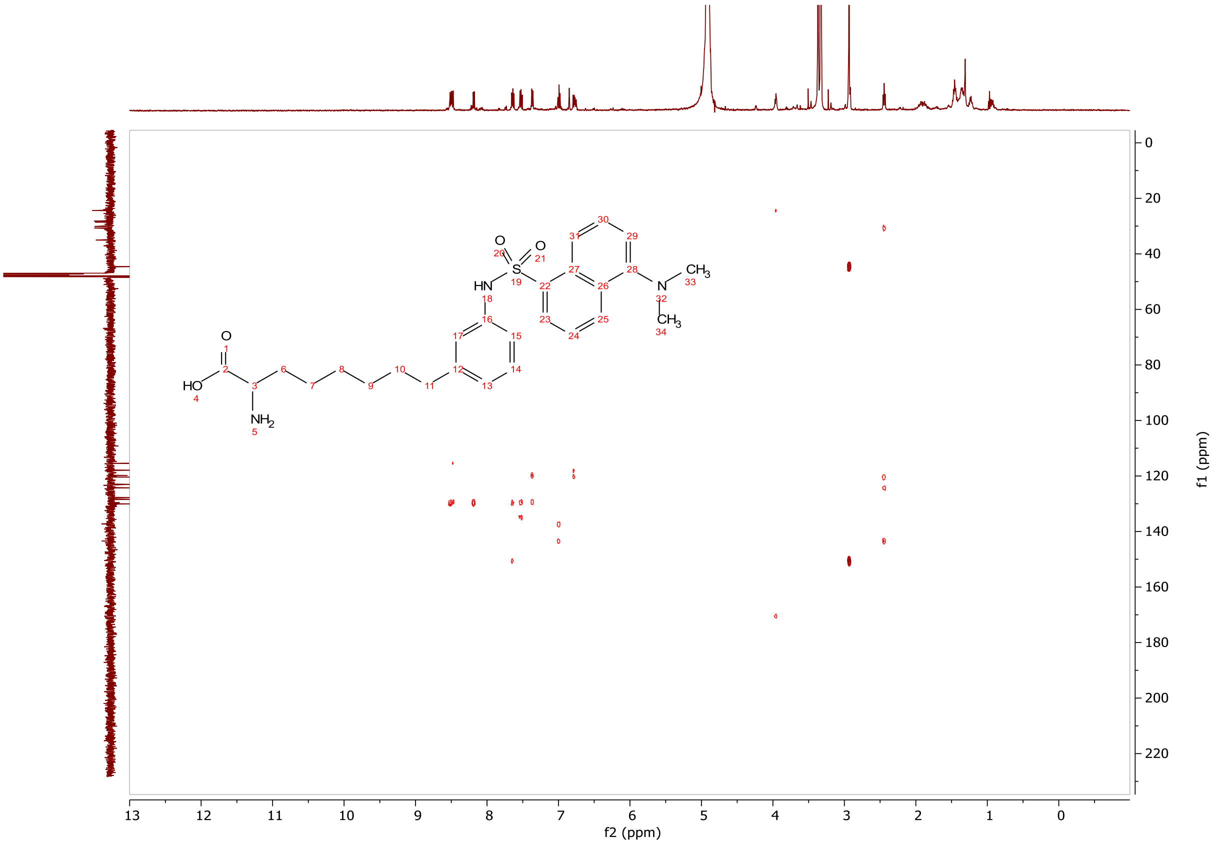
f2 (ppm)
f1 (ppm)
HO
NH2
HN
CH3
CH3

Diethyl-2-acetamido-2-(6-bromohexyl)malonate

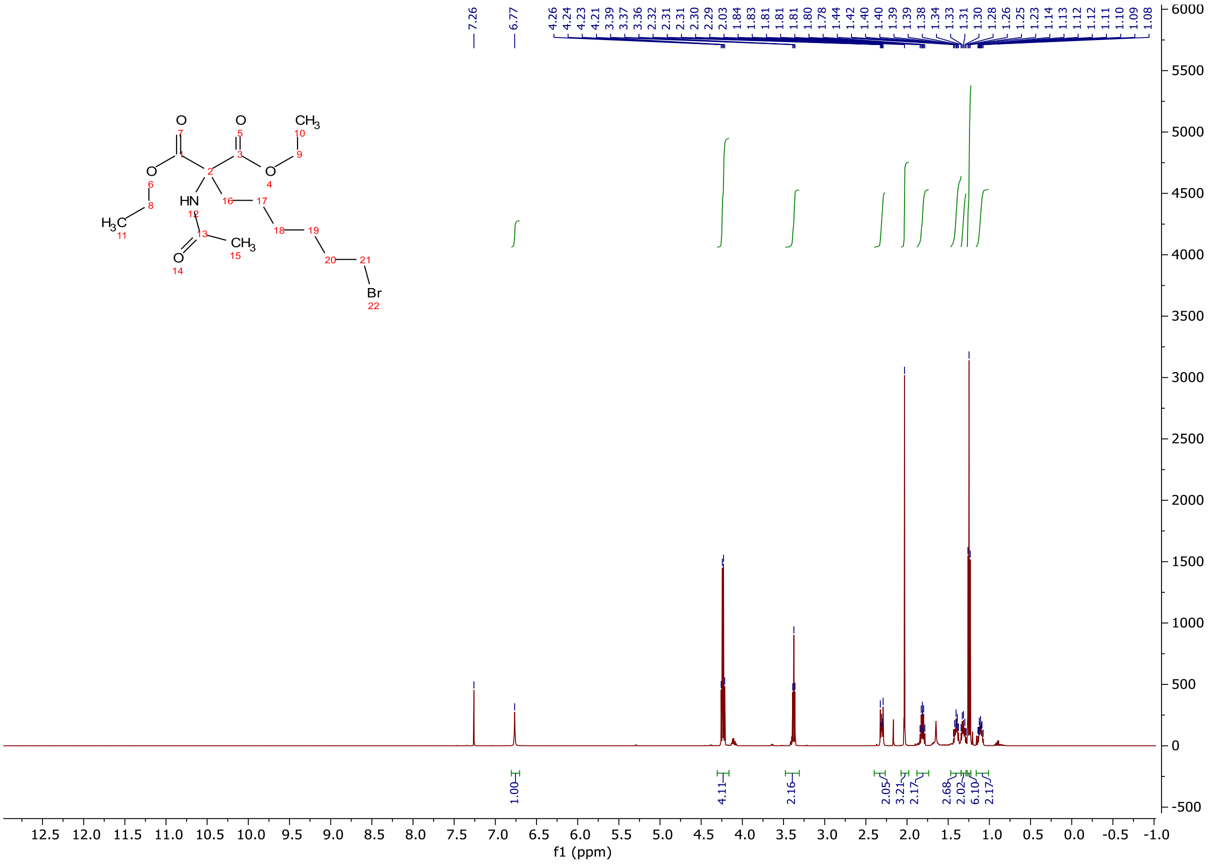

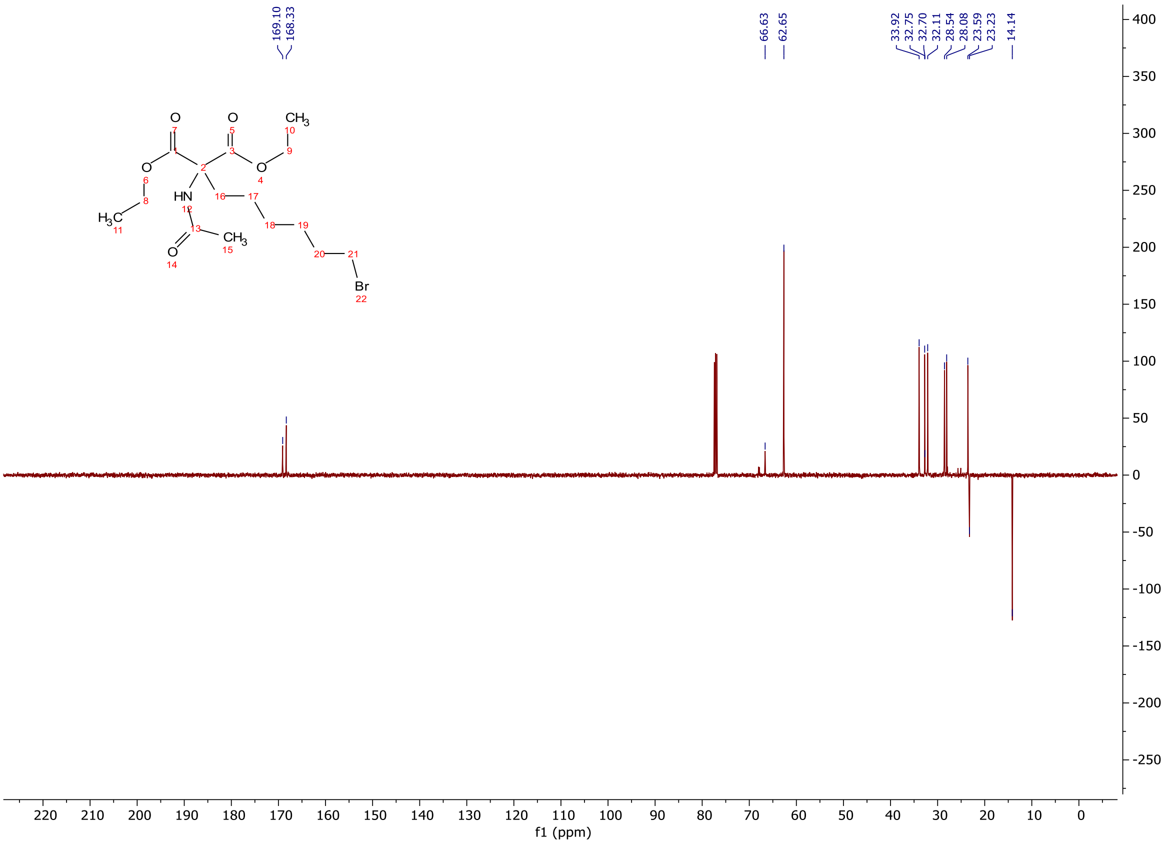

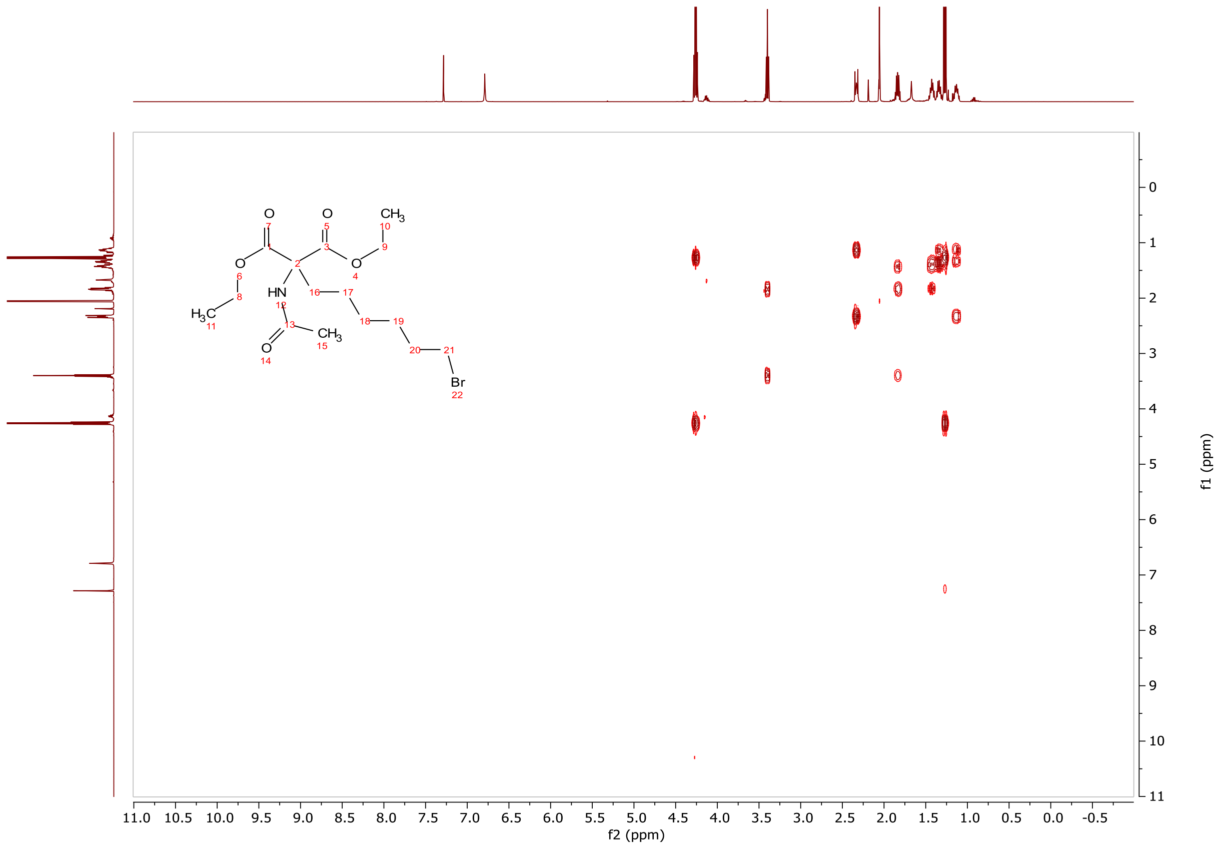

f2 (ppm)
f1 (ppm)
CH3
HN
H3C
Br

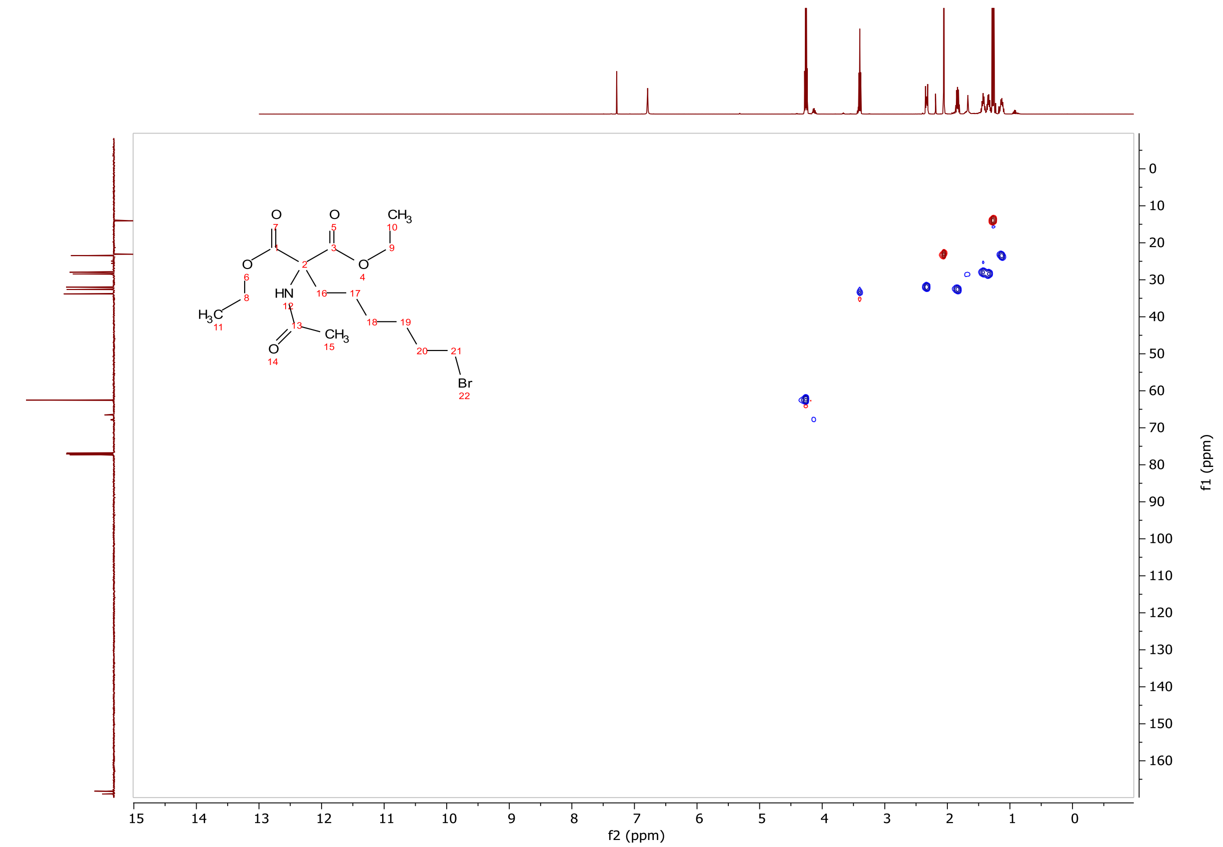

f2 (ppm)
f1 (ppm)
CH3
HN
H3C
Br

2-acetamido-8-bromo-2-(ethoxycarbonyl)octanoic acid

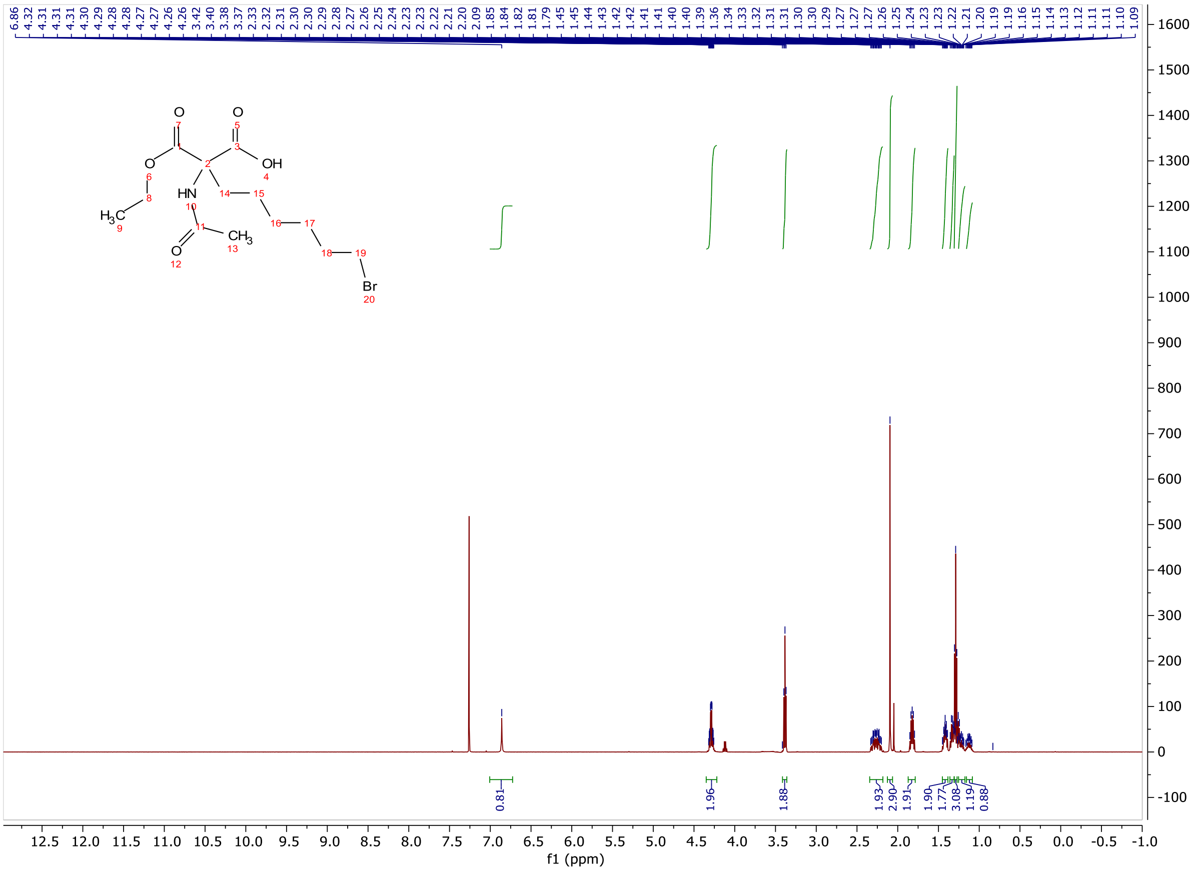

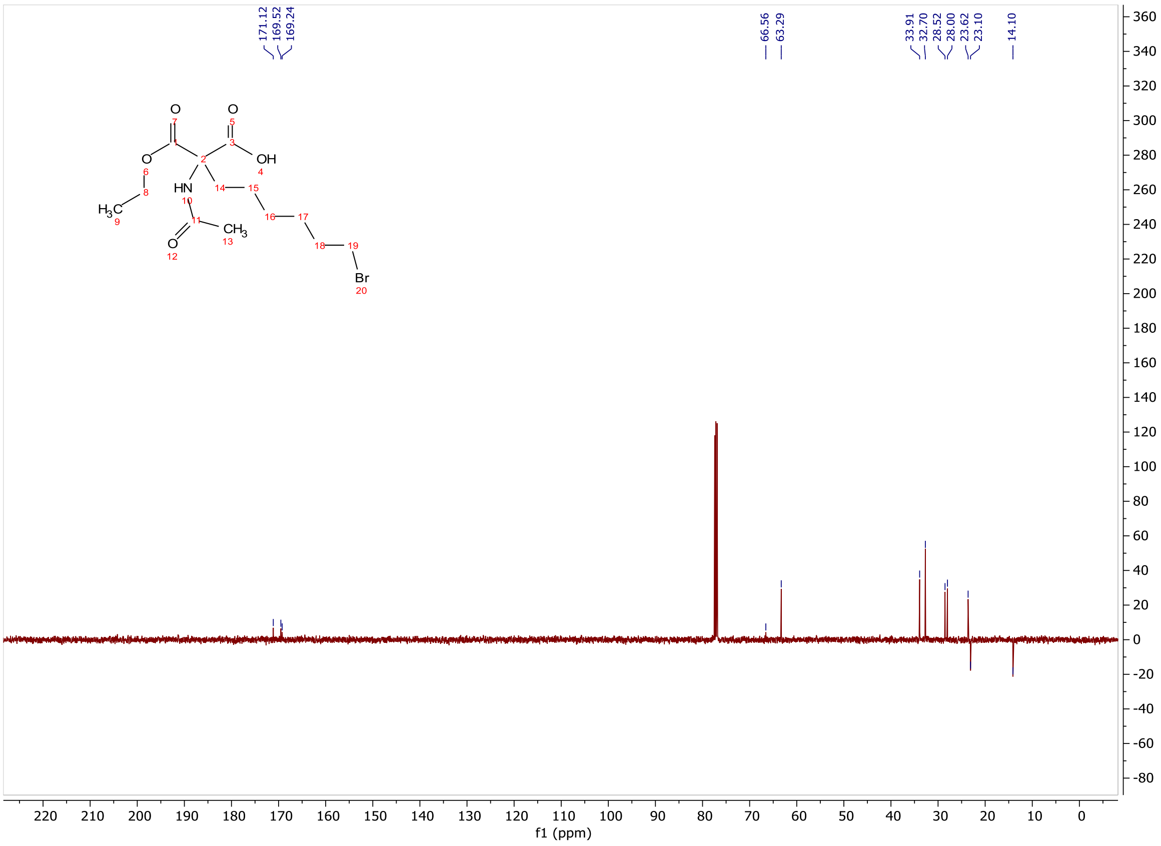

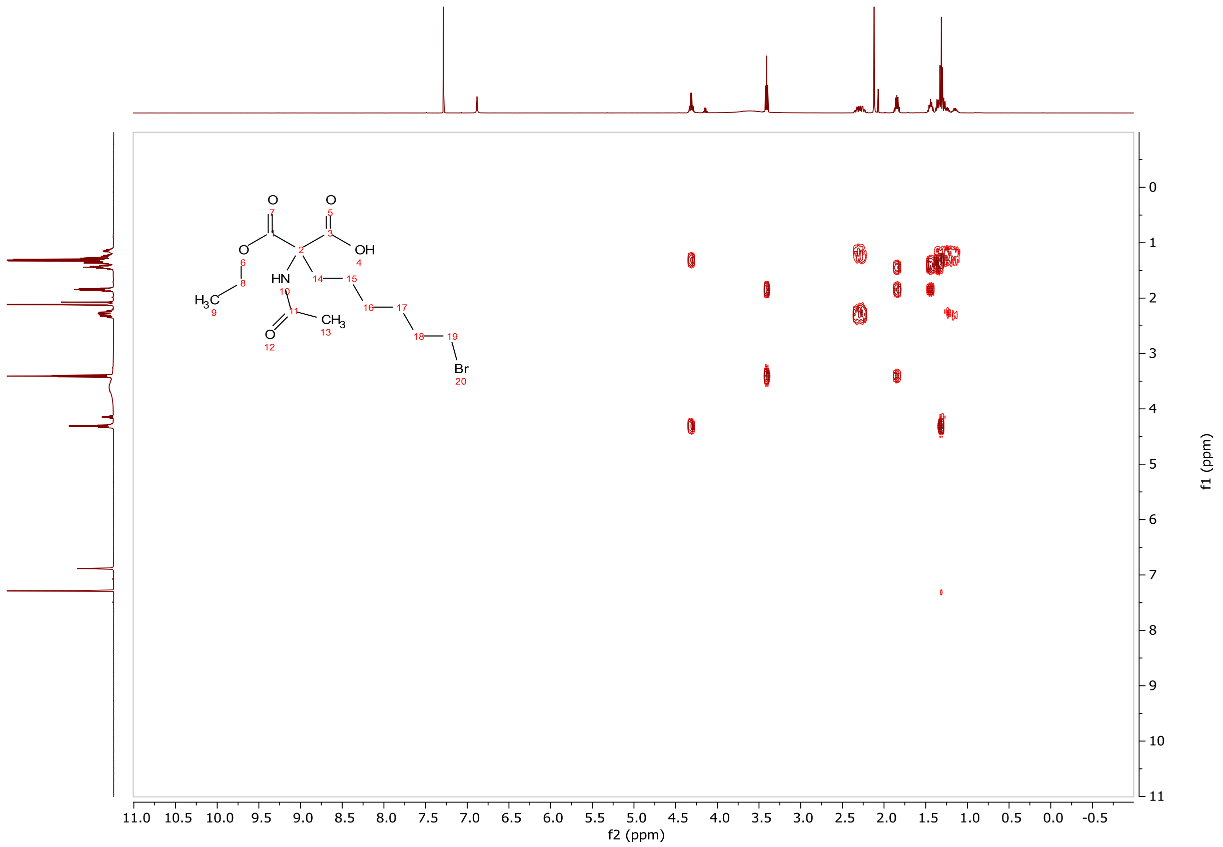

O
O
OH
O
HN
H3C
O
CH3
Br
f2 (ppm)
f1 (ppm)

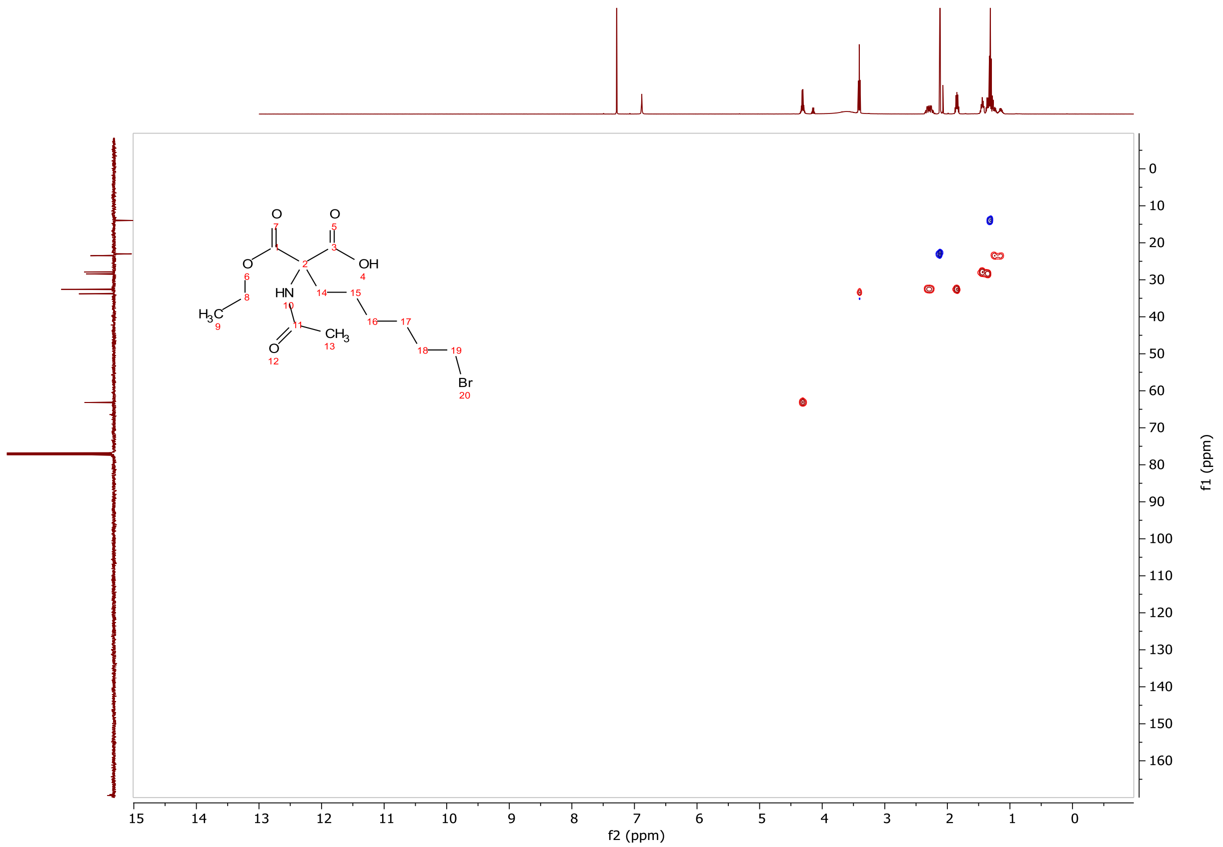

O
O
OH
O
HN
H3C
O
CH3
Br
f2 (ppm)
f1 (ppm)

Ethyl 2-acetamido-8-bromooctanoate

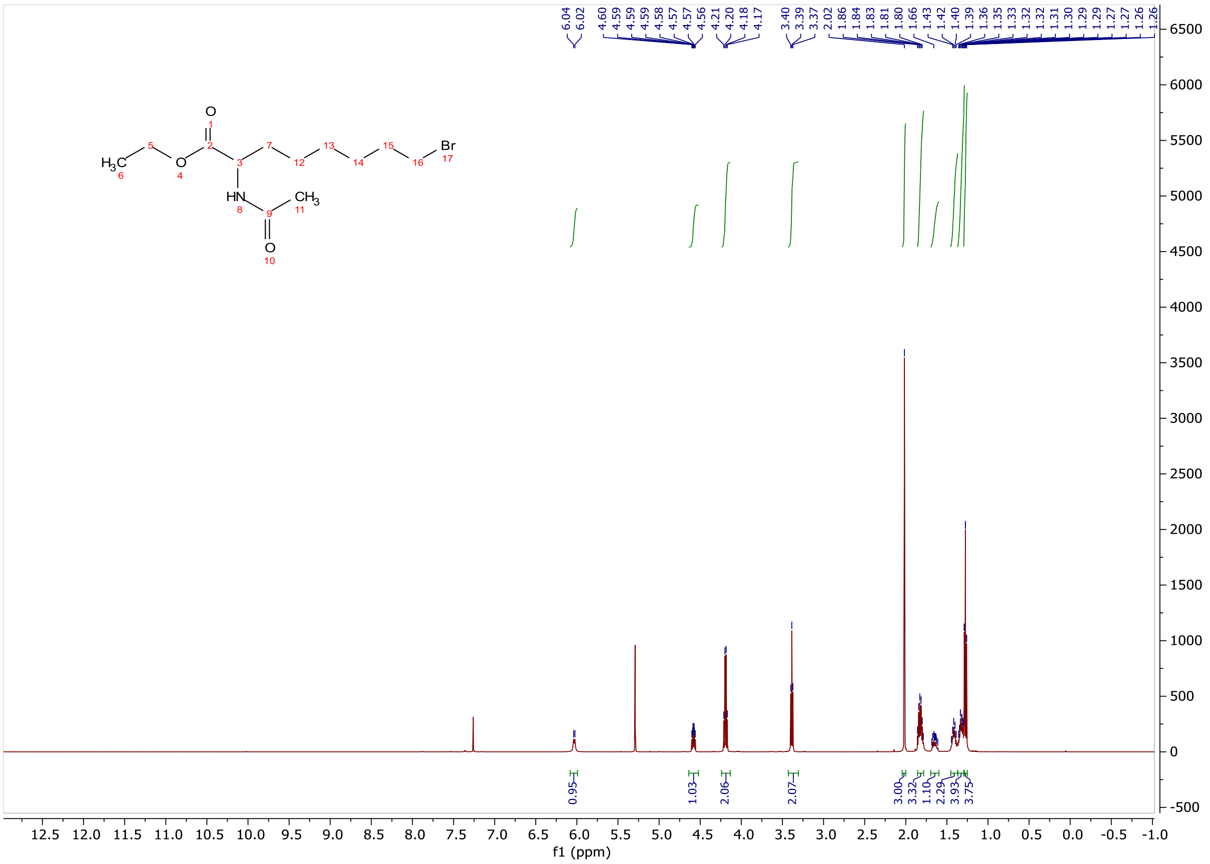

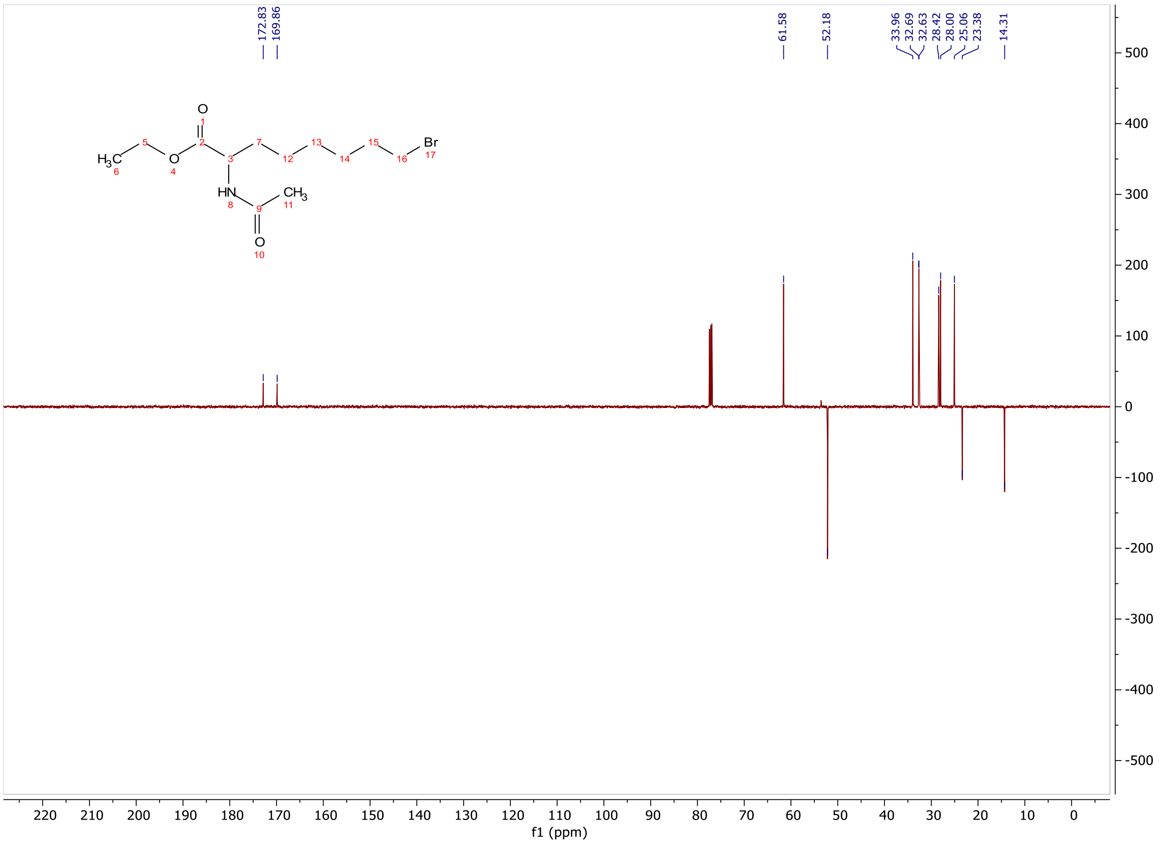

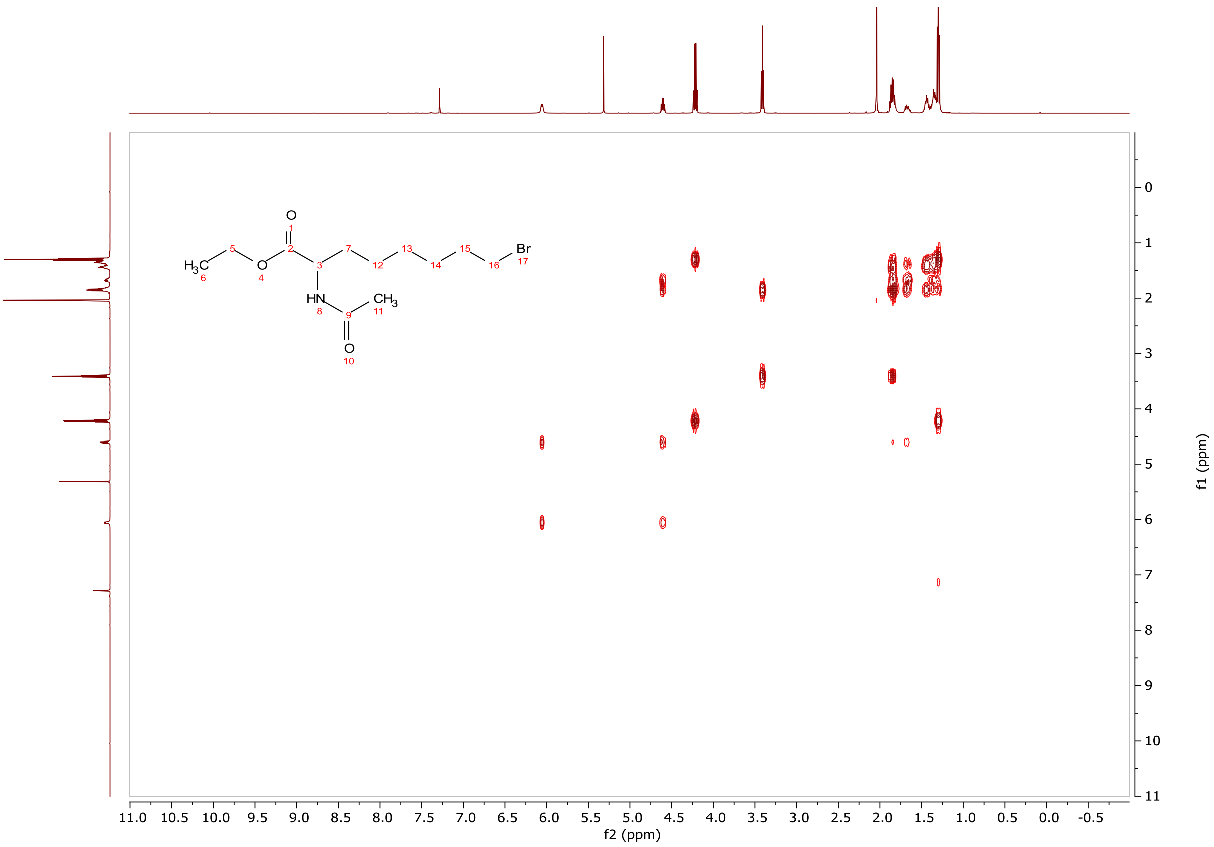

f2 (ppm)
f1 (ppm)

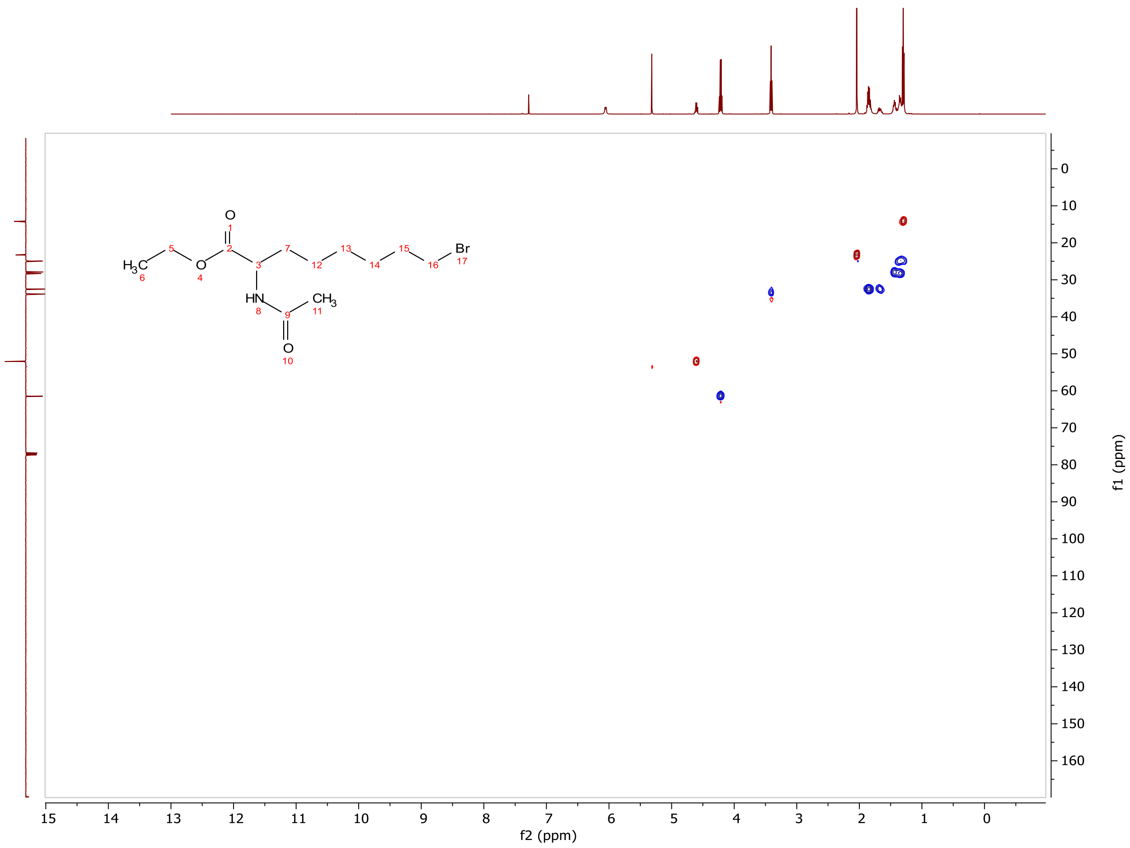

f2 (ppm)
f1 (ppm)

2-acetamido-8-bromooctanoic acid-1b

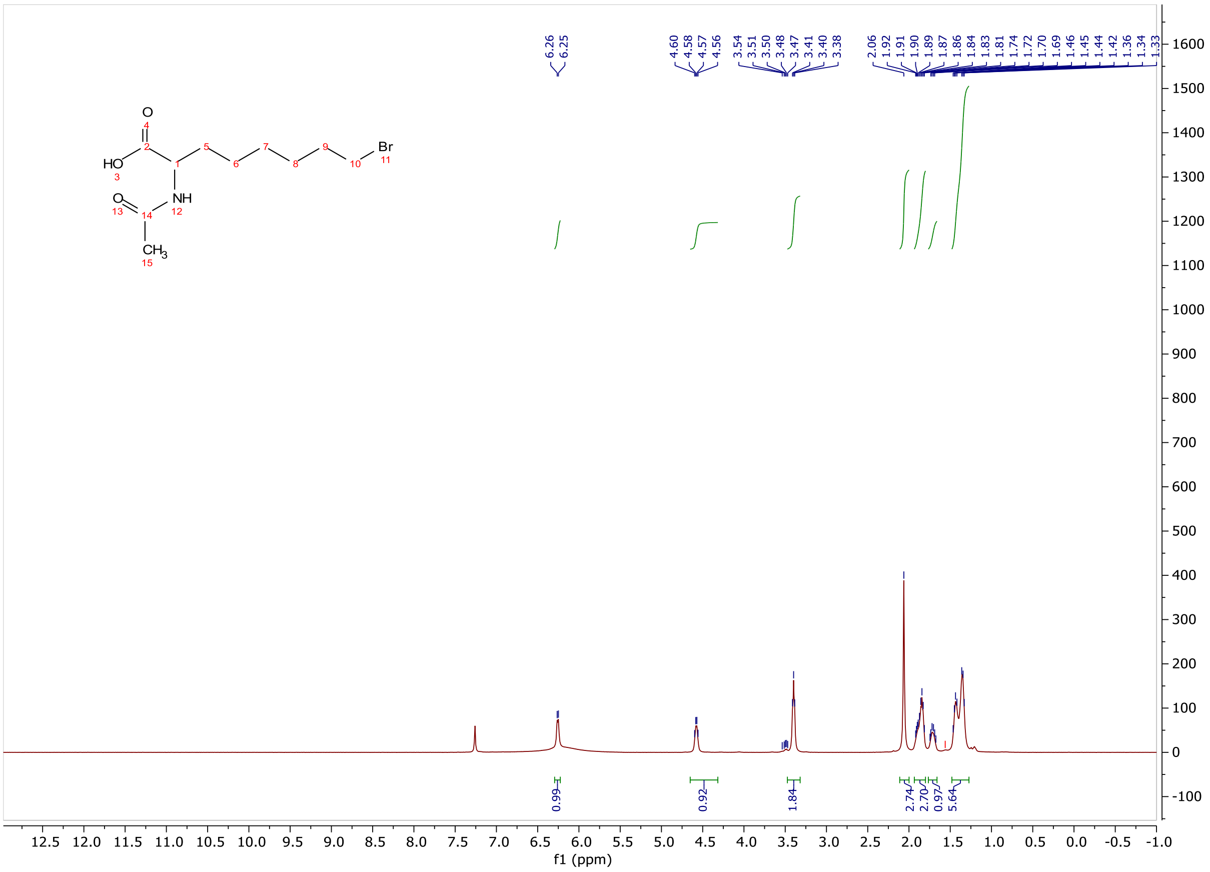

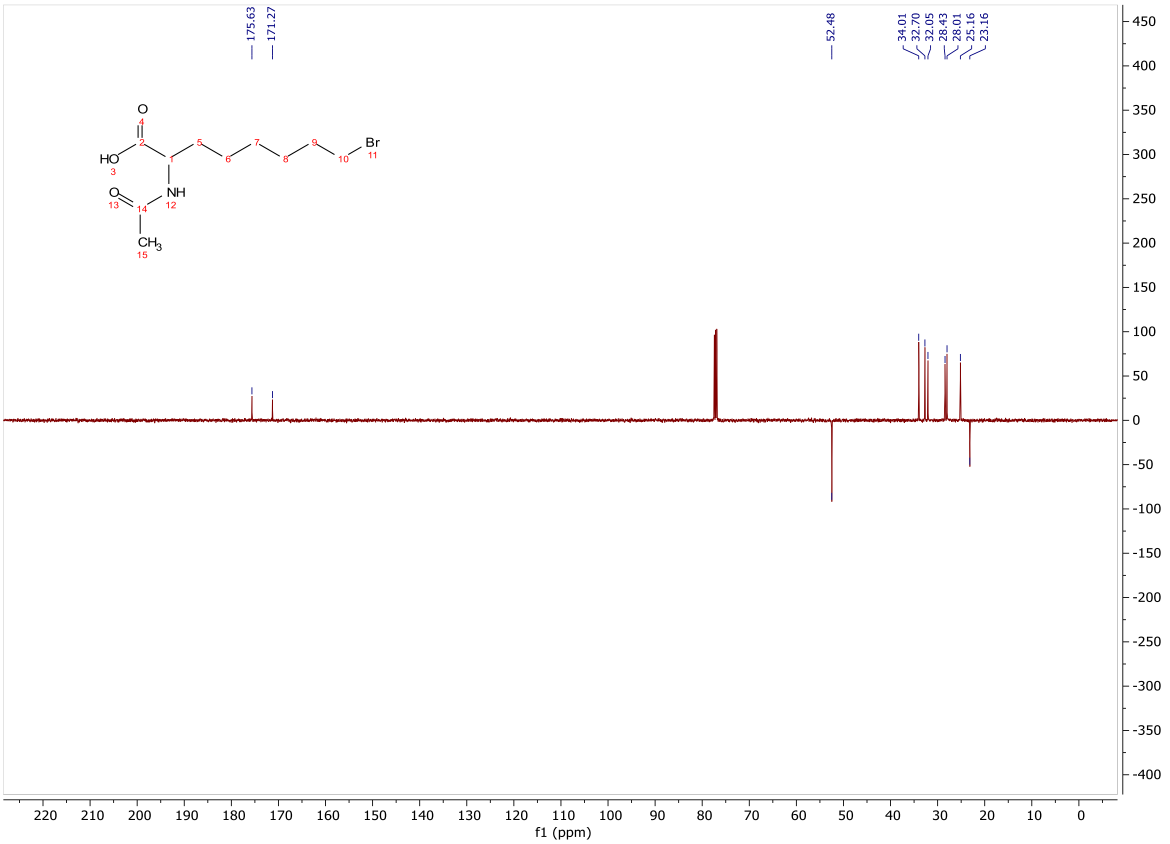

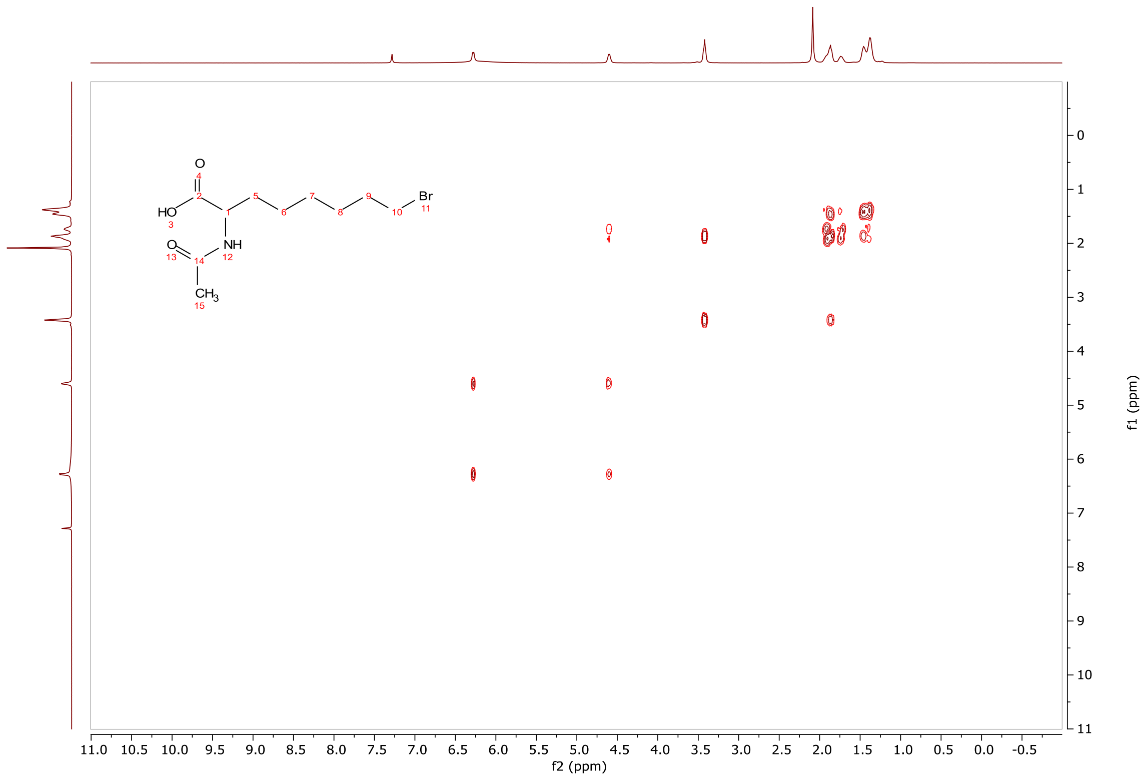

O
HO
NH
O
CH3
Br
f2 (ppm)
f1 (ppm)

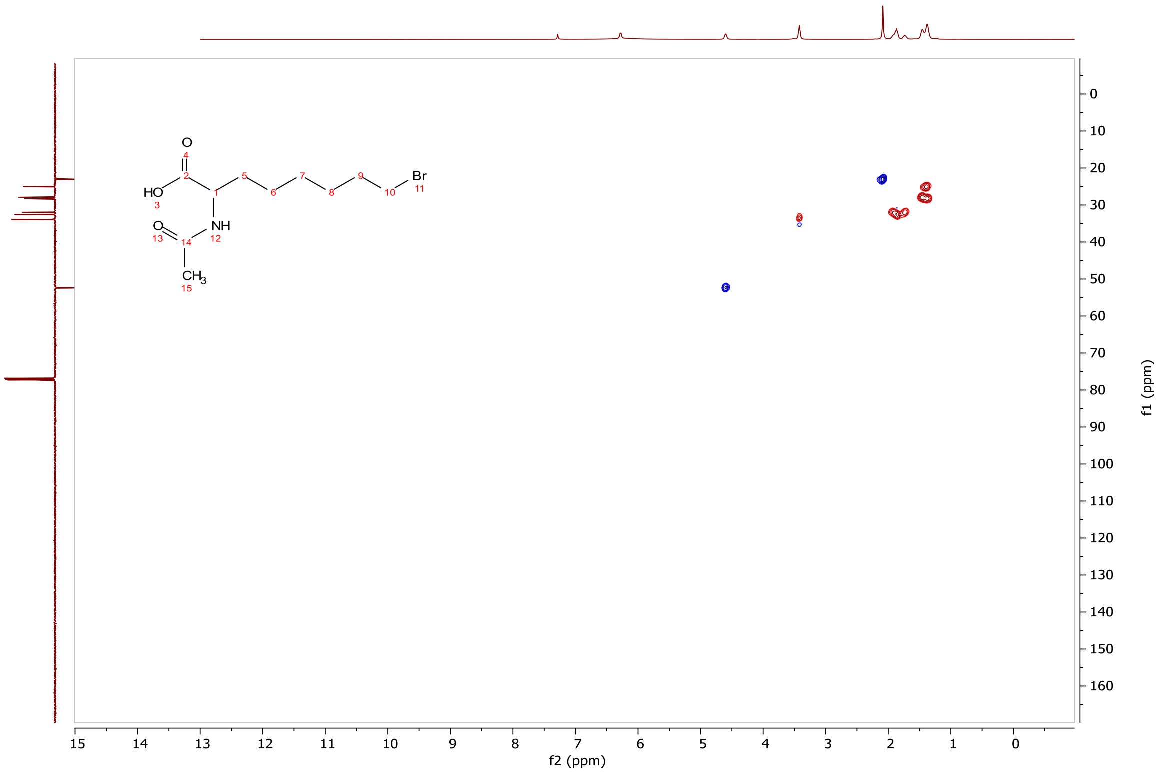

O
HO
NH
O
CH3
Br
f2 (ppm)
f1 (ppm)

2-amino-8-chlorooctanoic acid hydrochloride- 1f

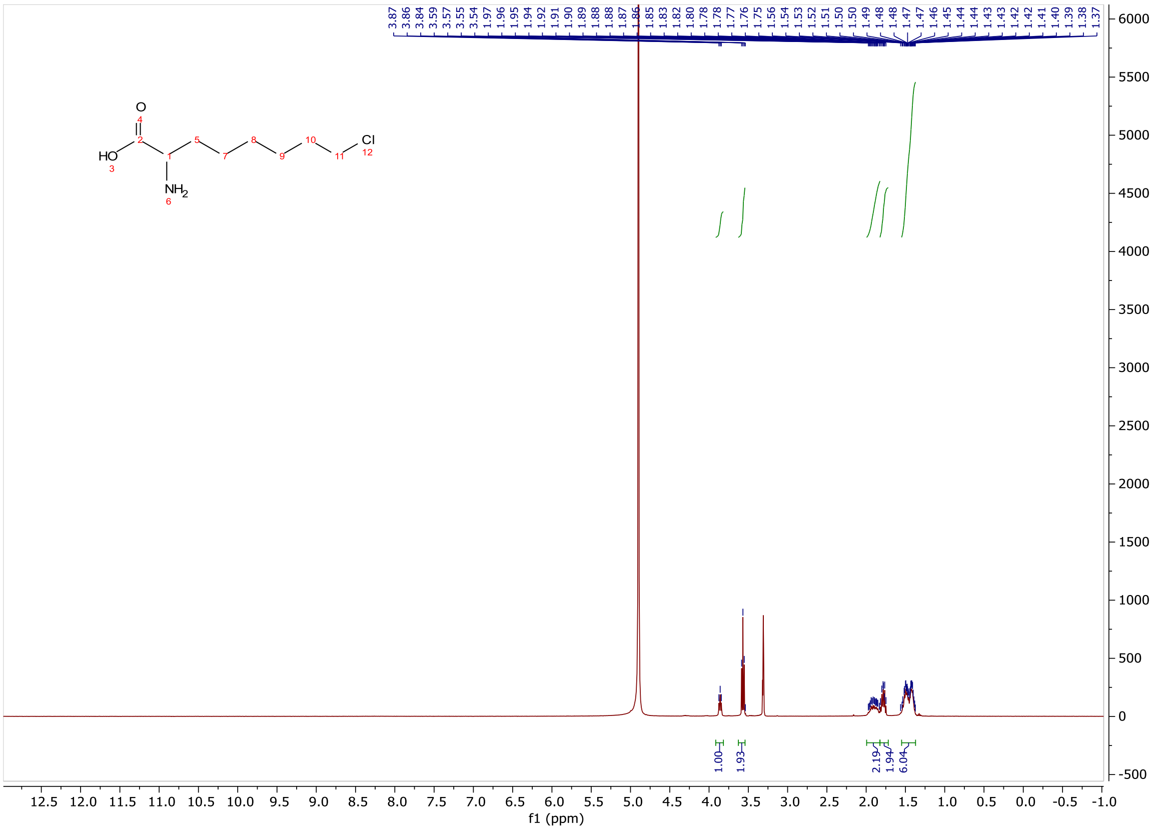

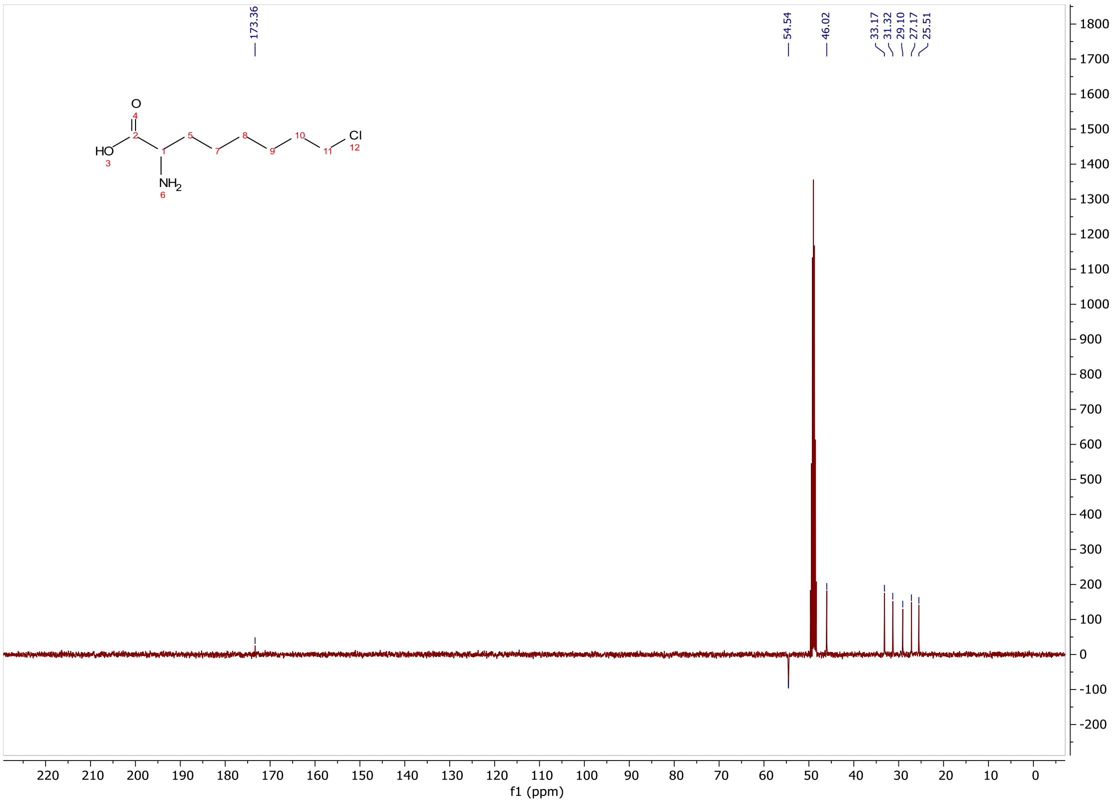

Diethyl 2-((tert-butoxycarbonyl)amino)malonate

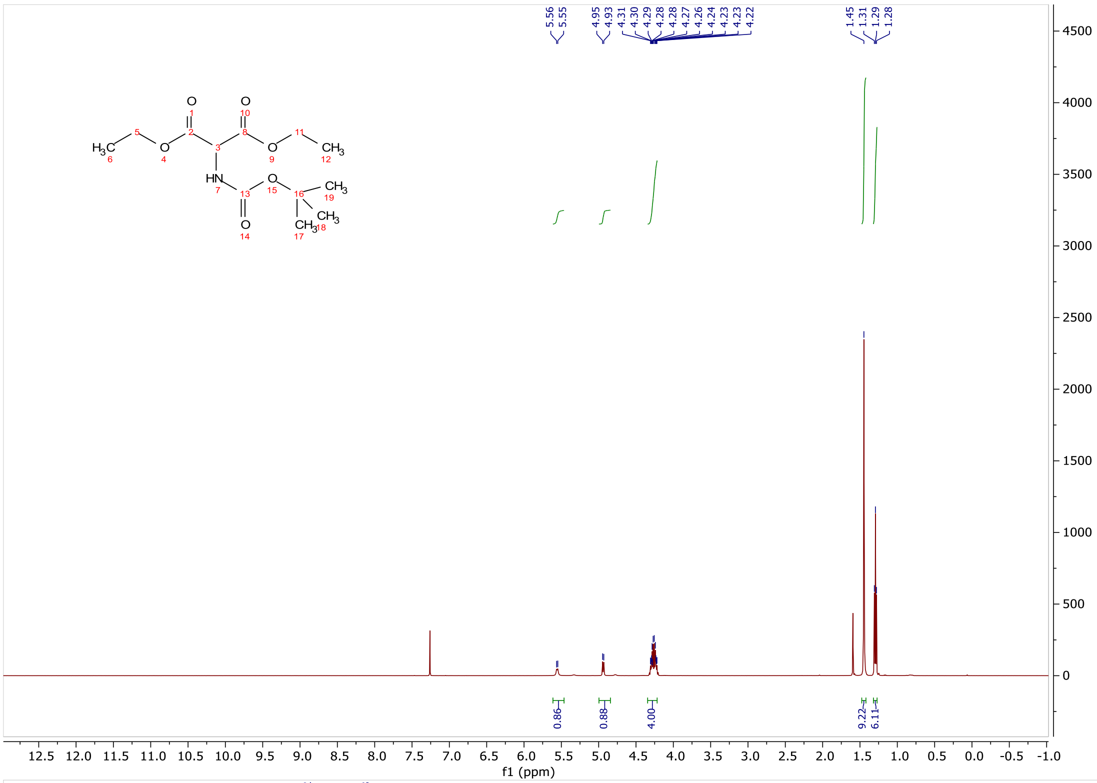

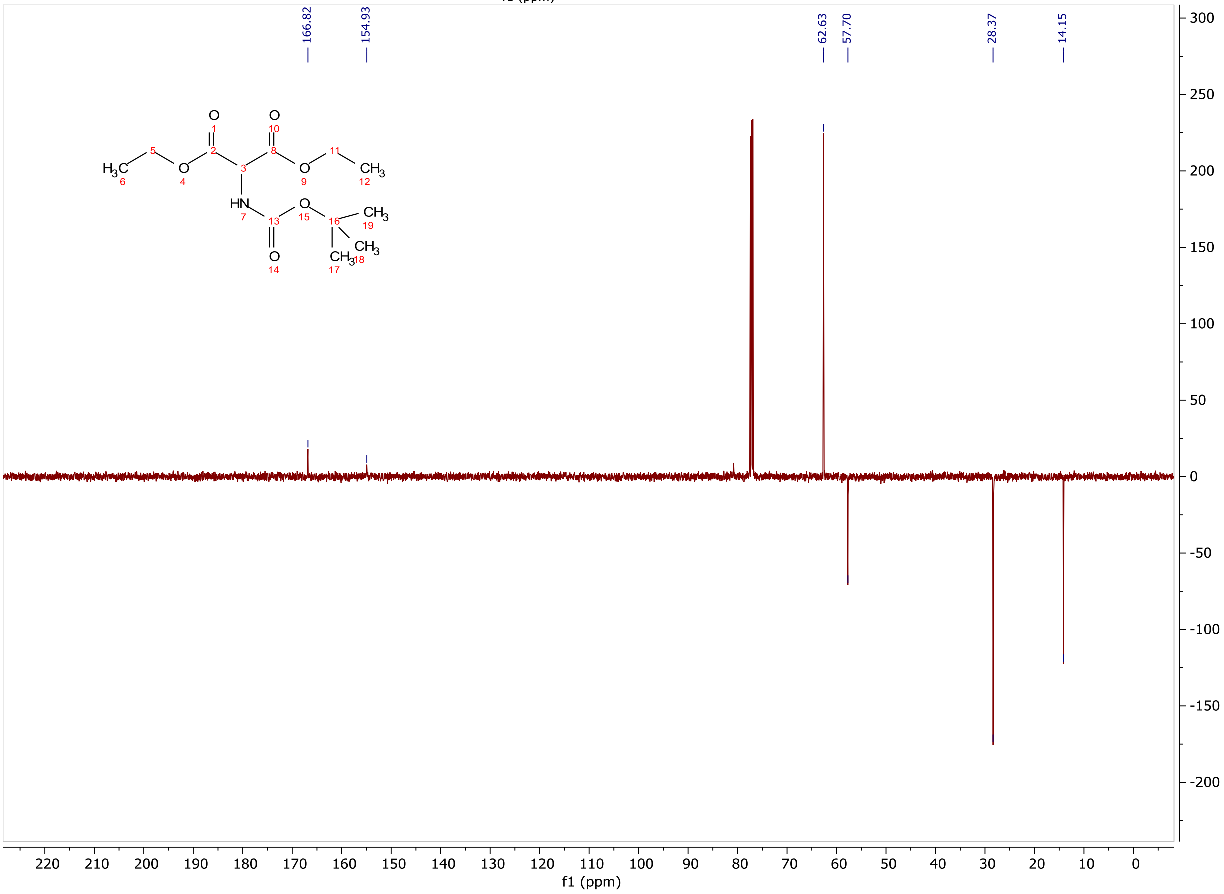

8-bromo-2-((tert-butoxycarbonyl)amino)-2-(ethoxycarbonyl)octanoic acid

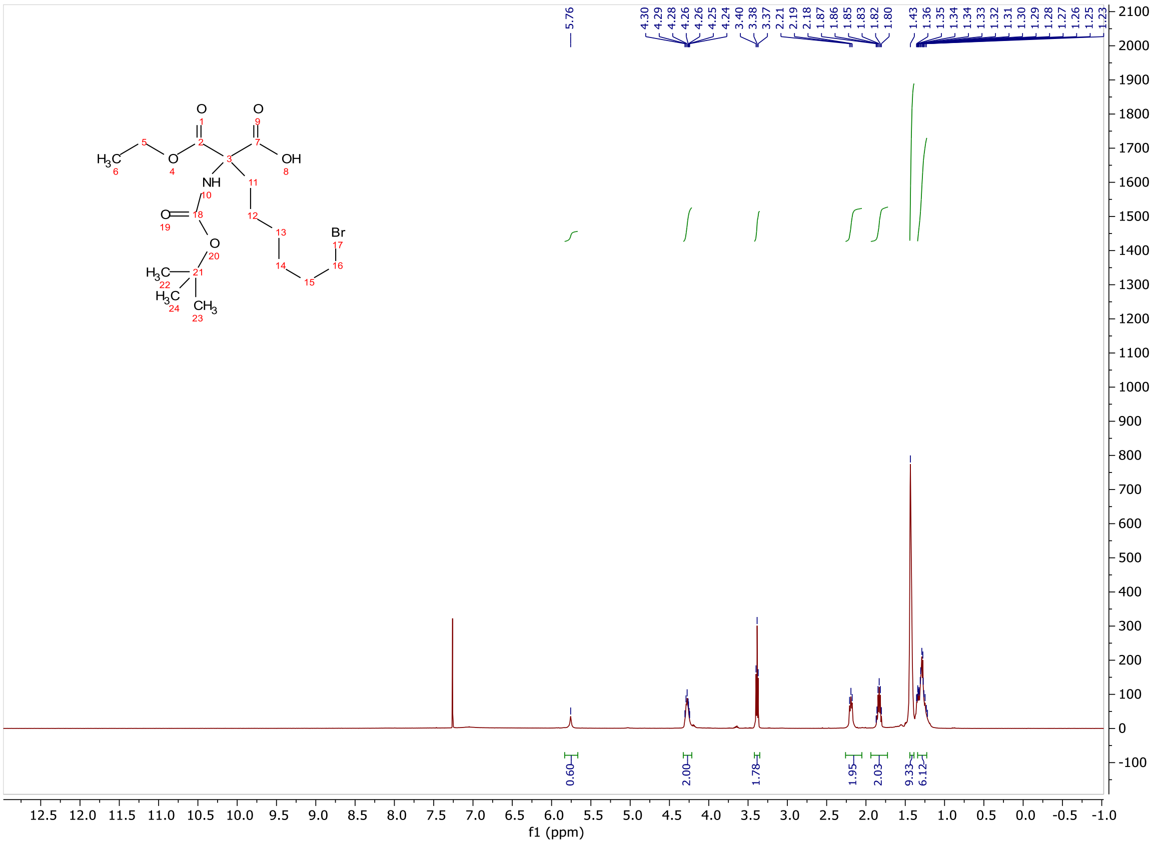

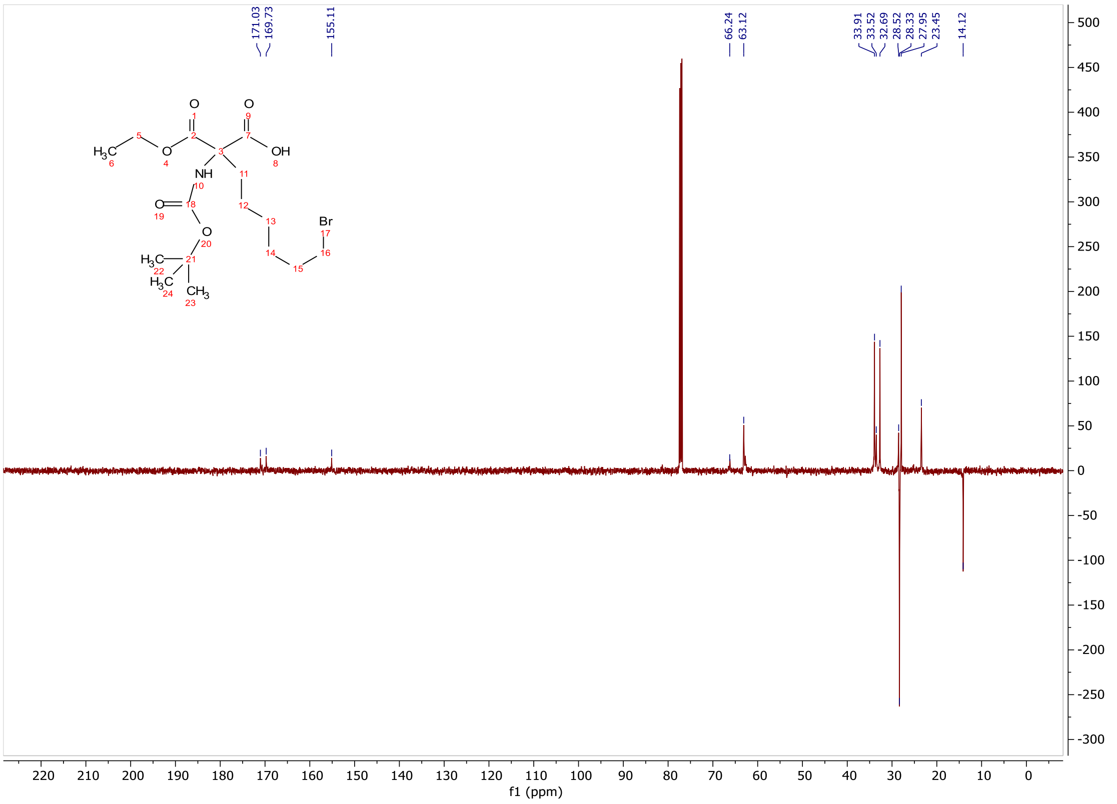

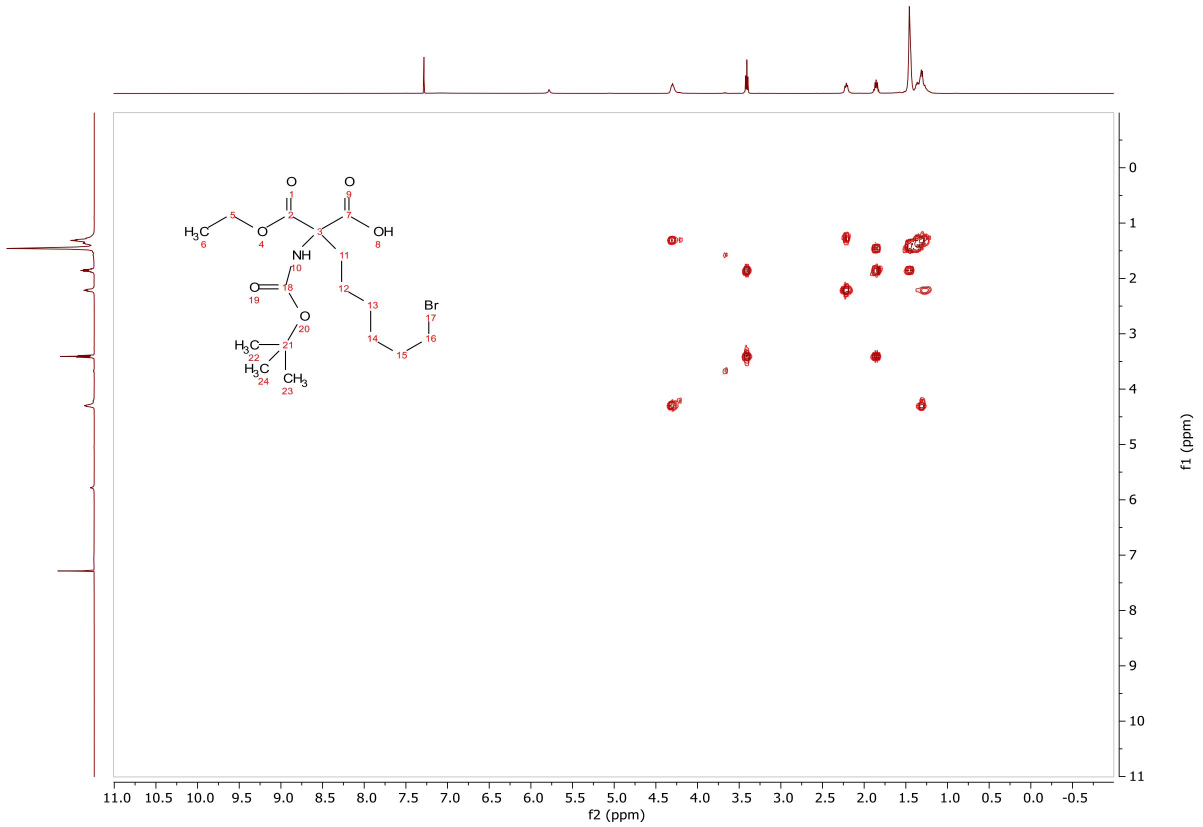

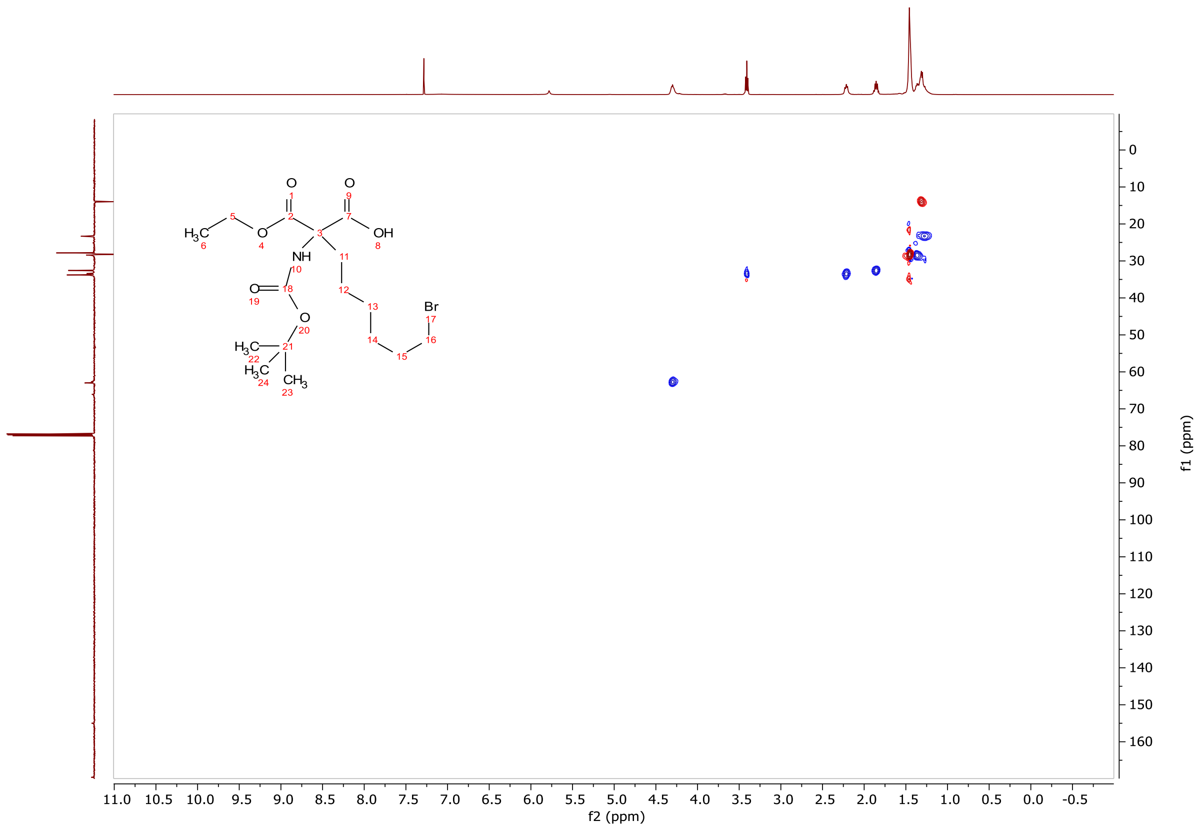

8-bromo-2-((tert-butoxycarbonyl)amino)octanoic acid- 1d

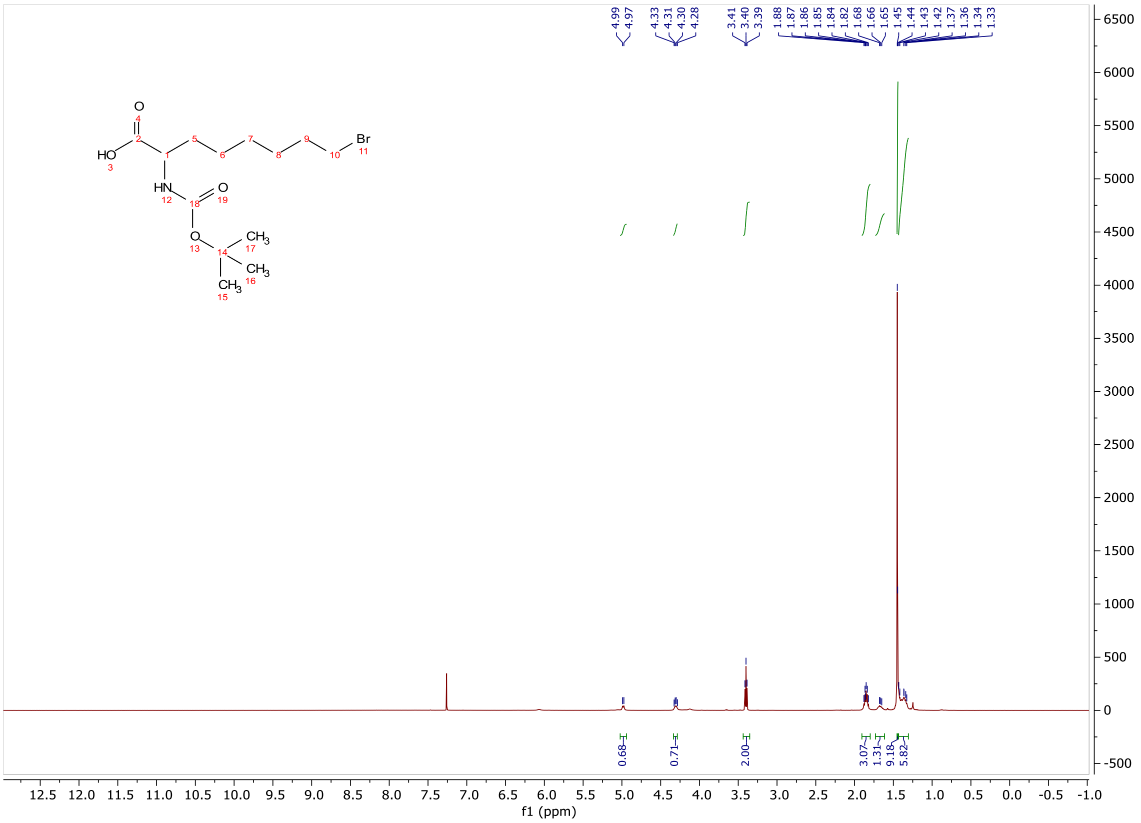

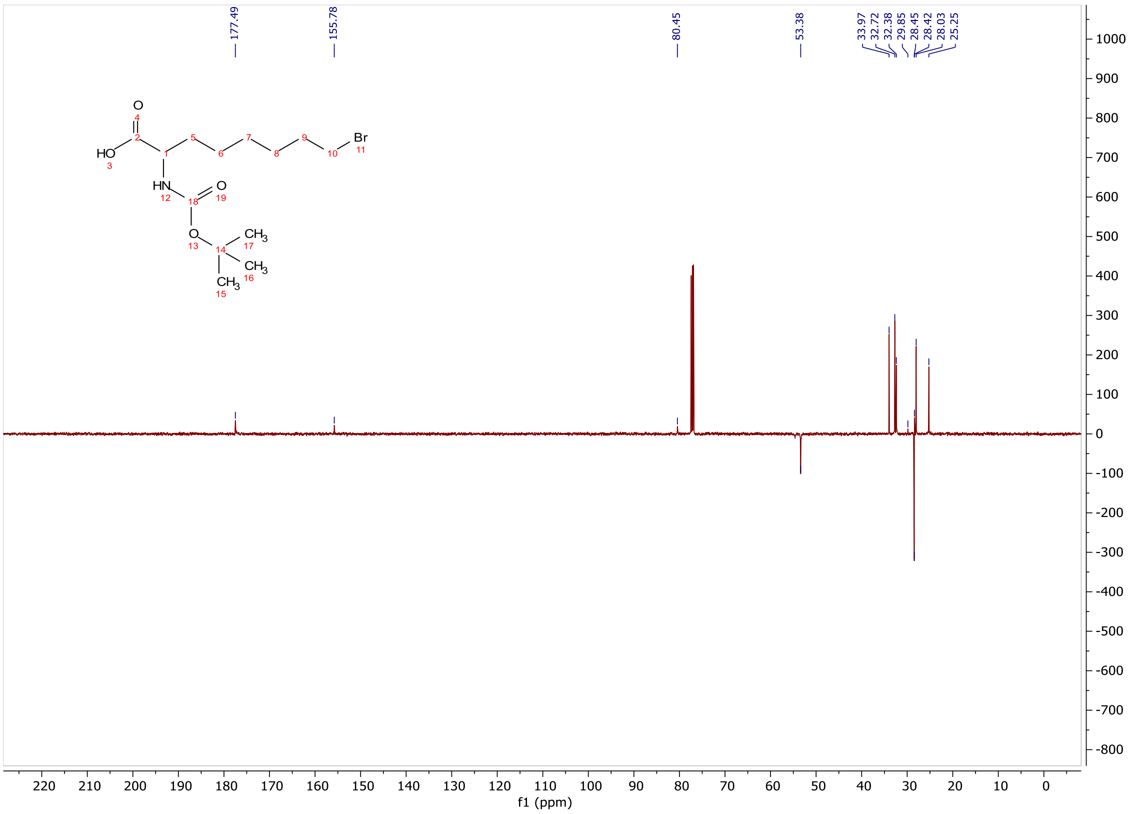

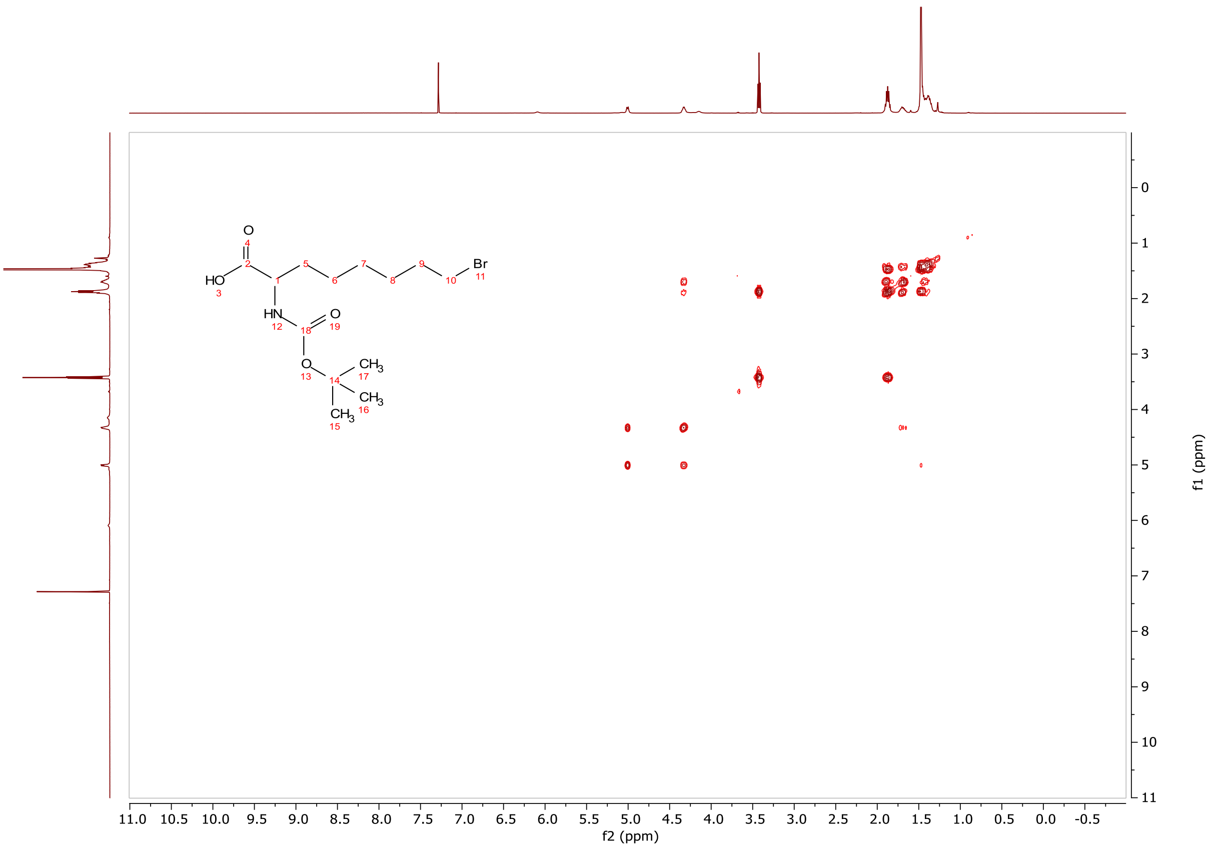

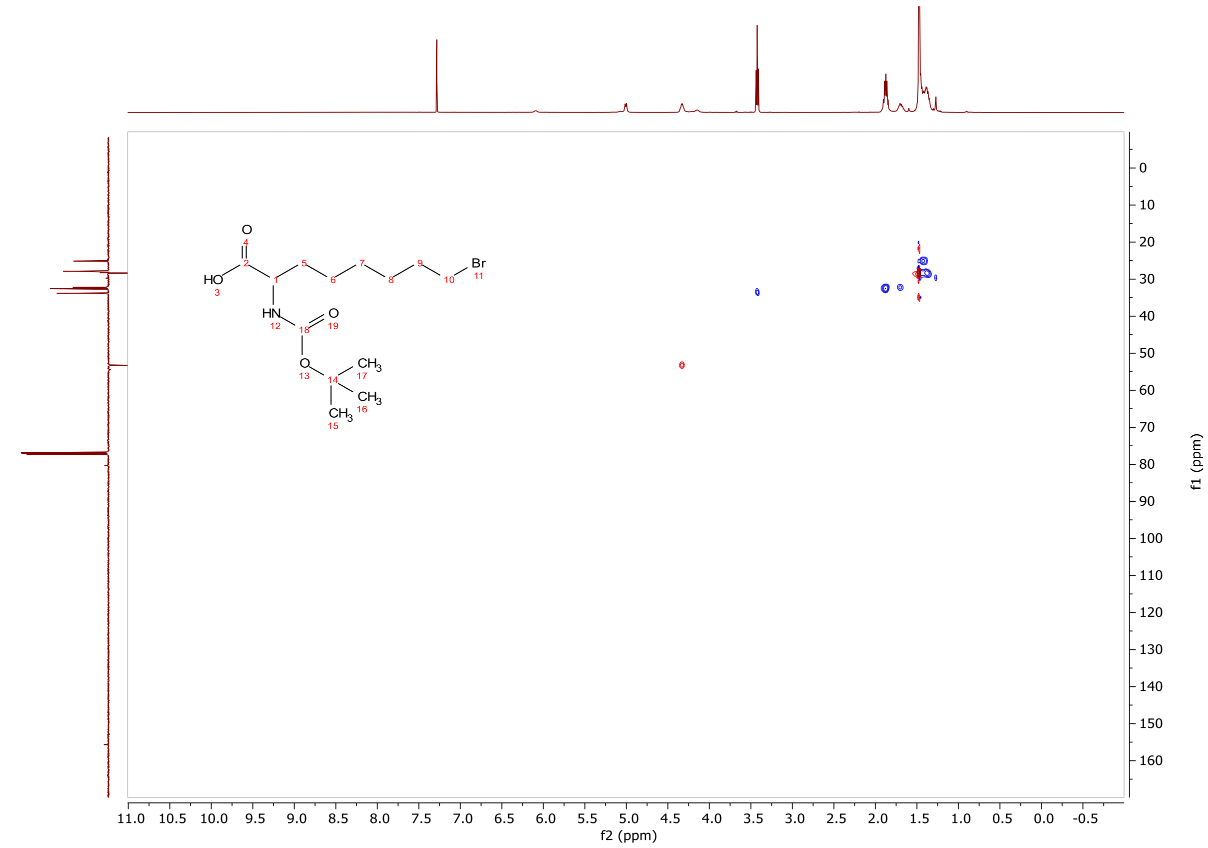

2-((tert-butoxycarbonyl)amino)-8-iodooctanoic acid- 1e

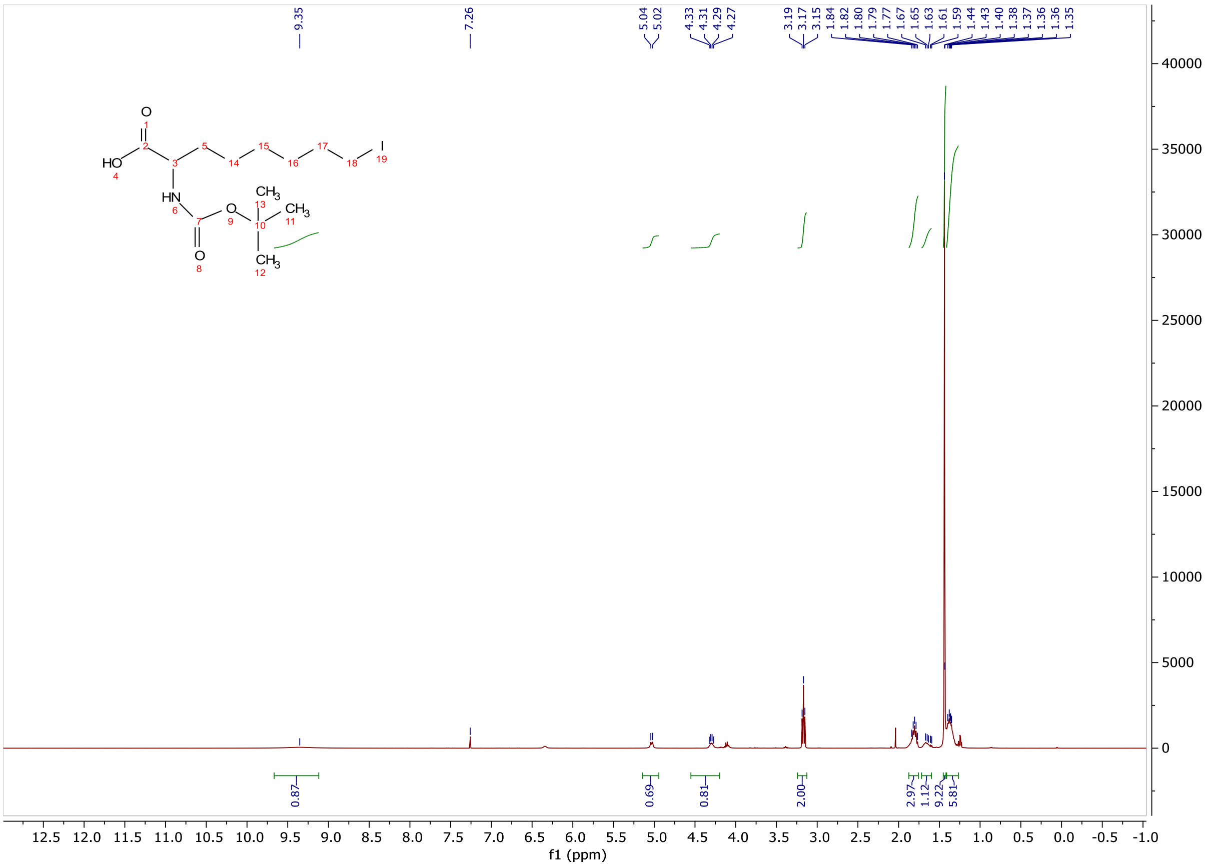

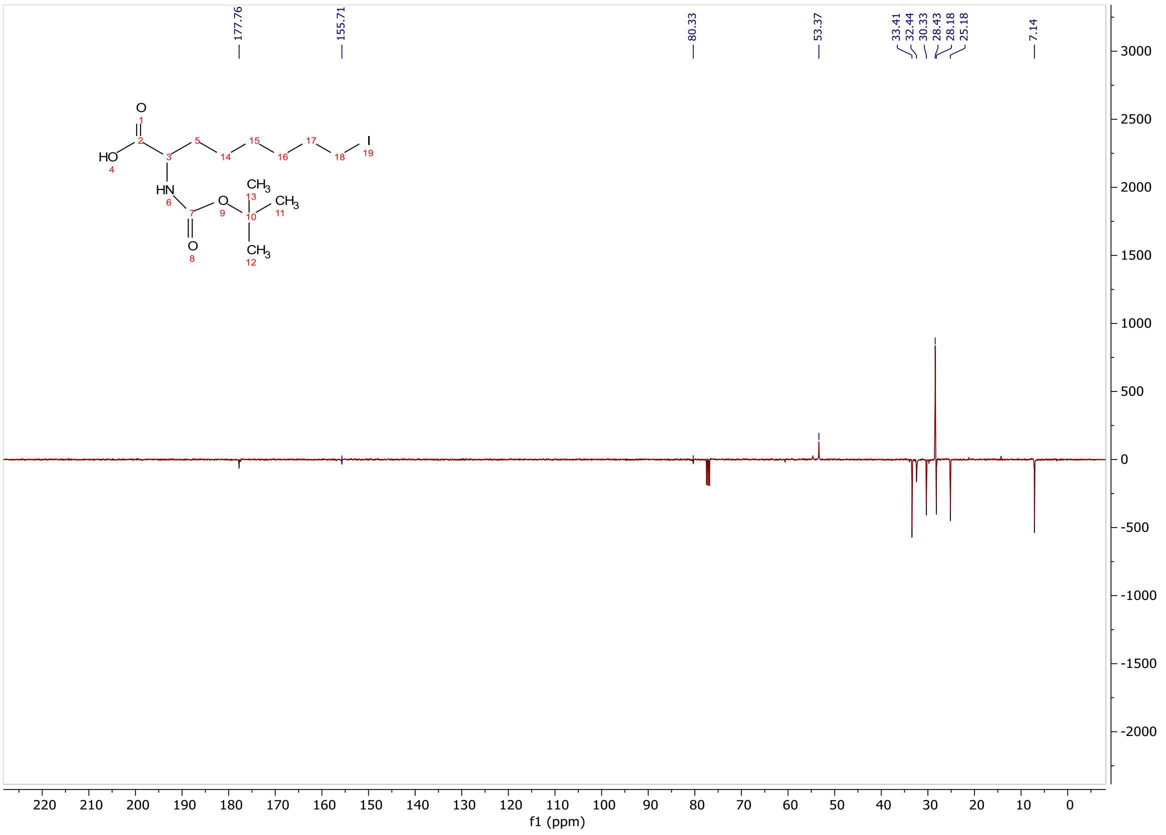

2-Amino-8-bromohexanoic acid TFA salt- 1a

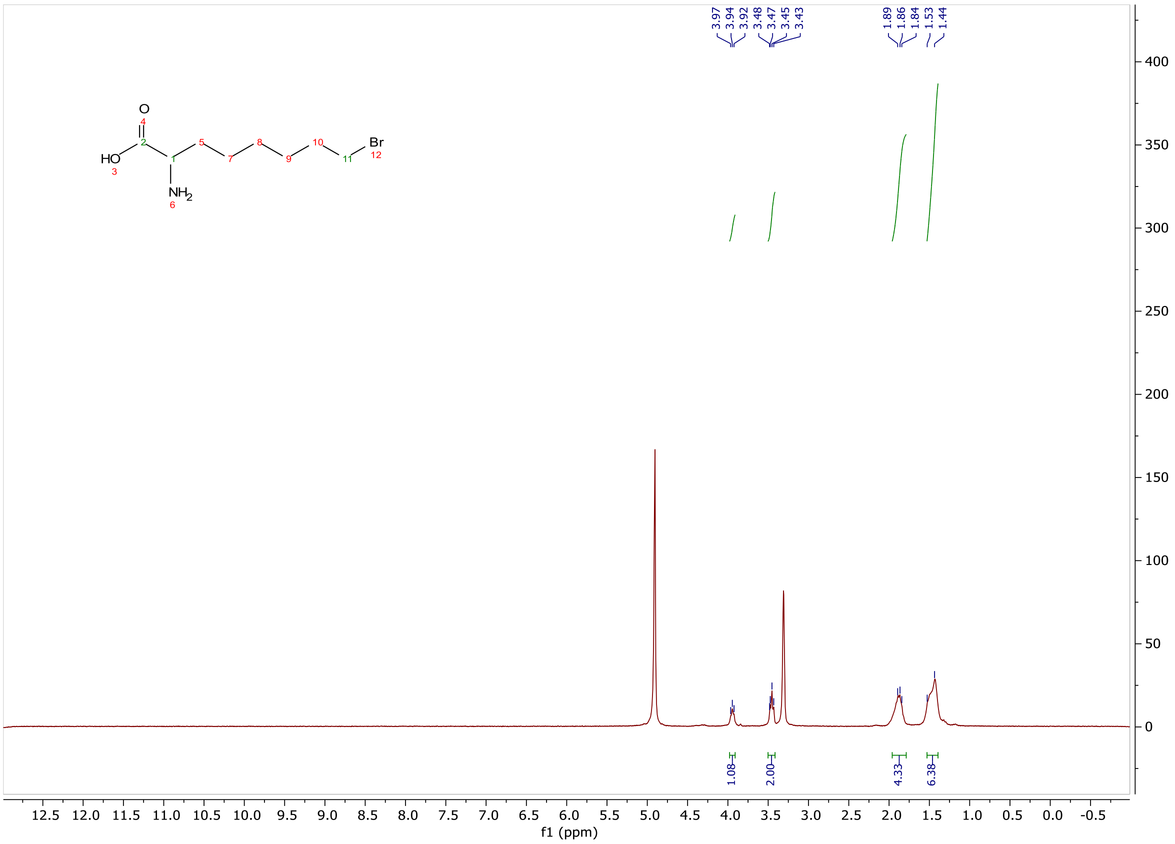

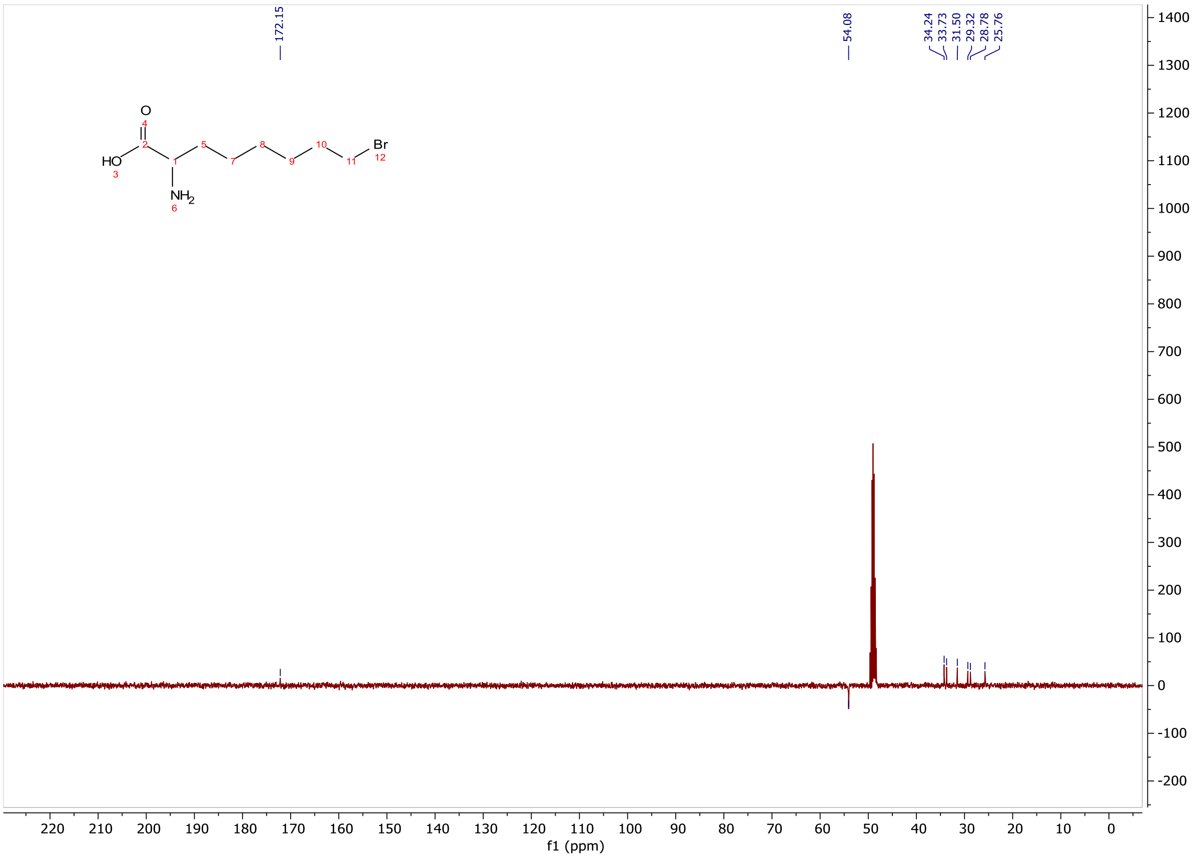

N-Boc-Tyr-OMe

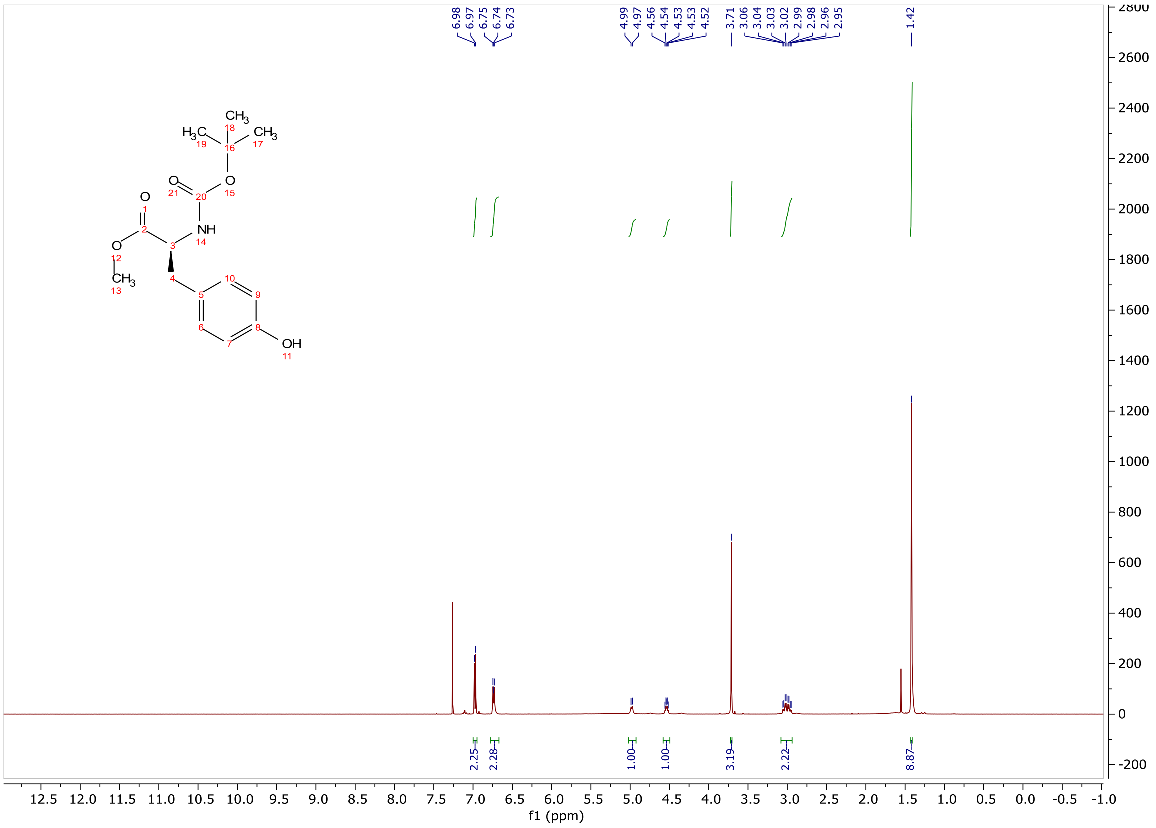

6.98
6.97
6.75
6.74
6.73
4.99
4.97
4.56
4.54
4.53
4.53
4.52
3.71
3.06
3.04
3.03
3.02
2.99
2.98
2.96
2.95
1.42
2.25
2.28
1.00
1.00
3.19
2.22
8.87
f1 (ppm)

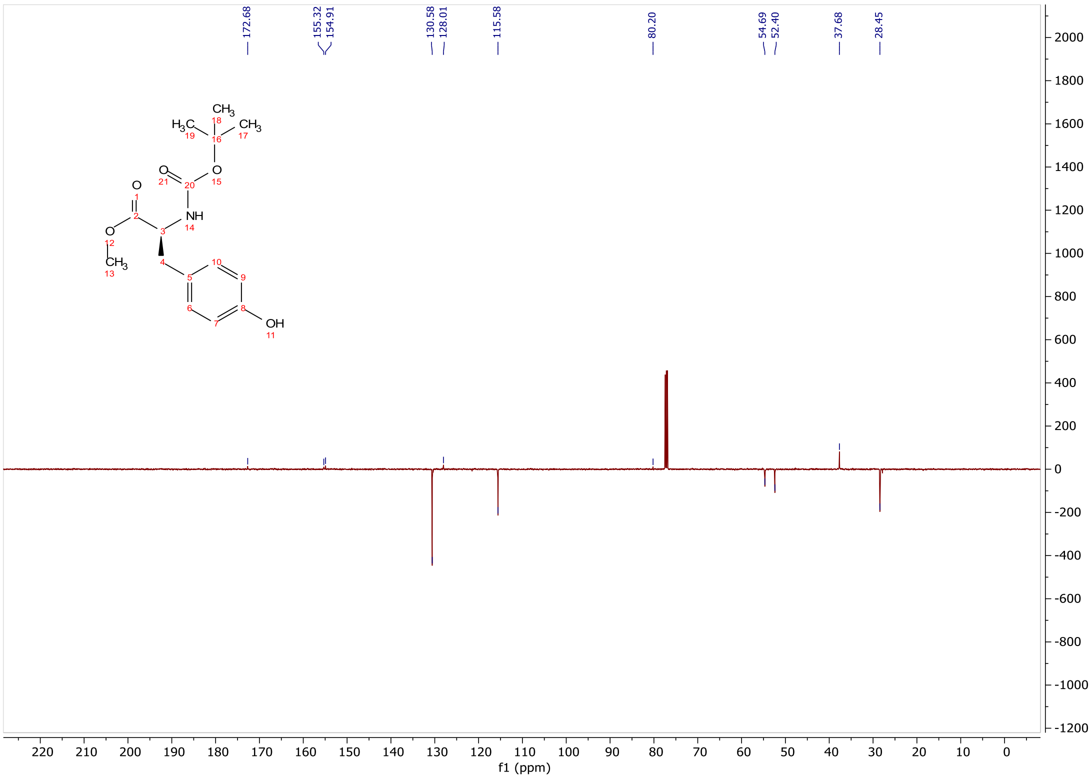

172.68
155.32
154.91
130.58
128.01
115.58
80.20
54.69
52.40
37.68
28.45
f1 (ppm)

Methyl-(S)-2-amino-3-(4-(4-bromobutoxy)phenyl)propanoate TFA salt- 1g

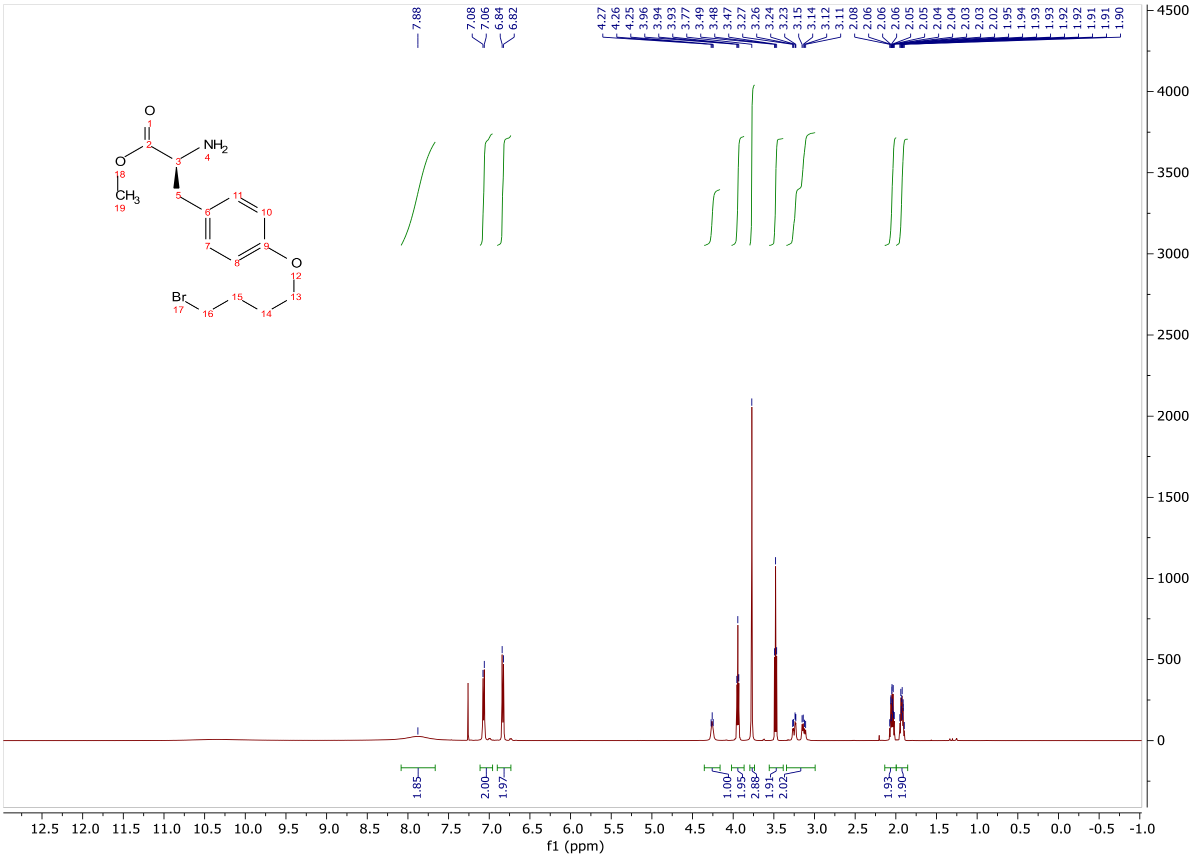

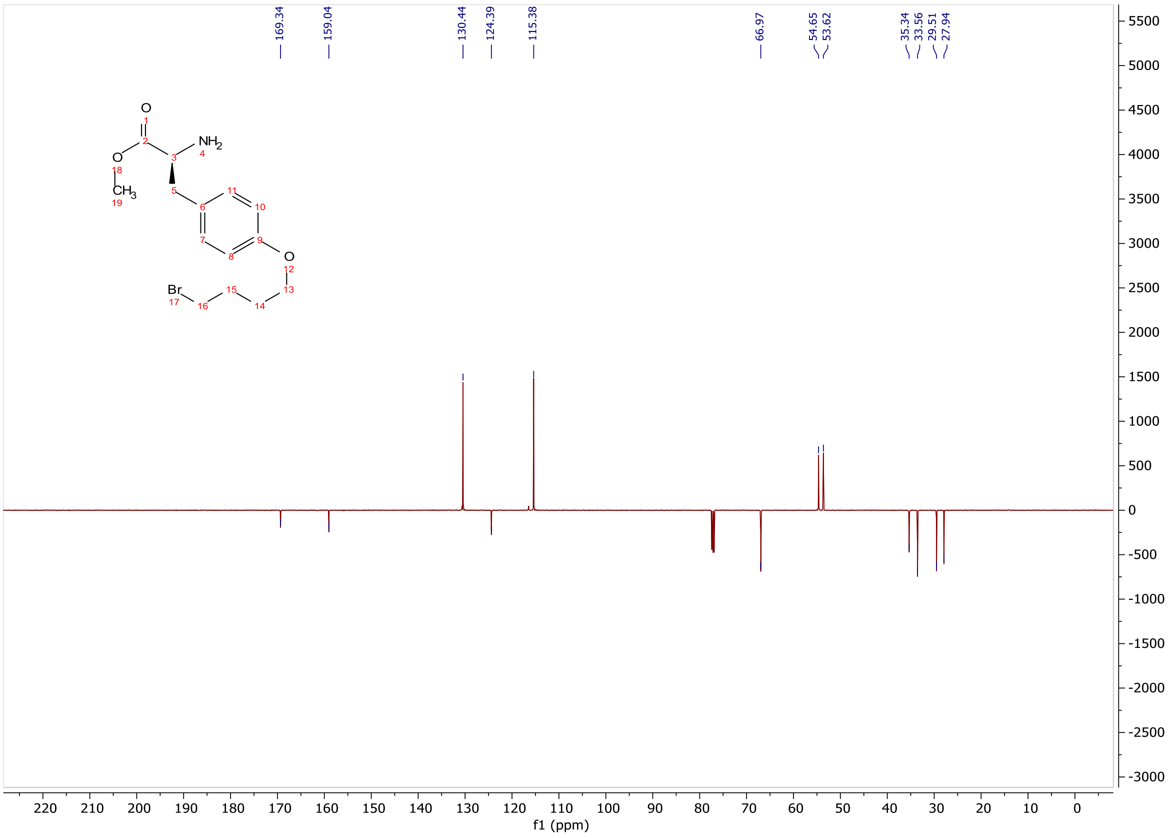

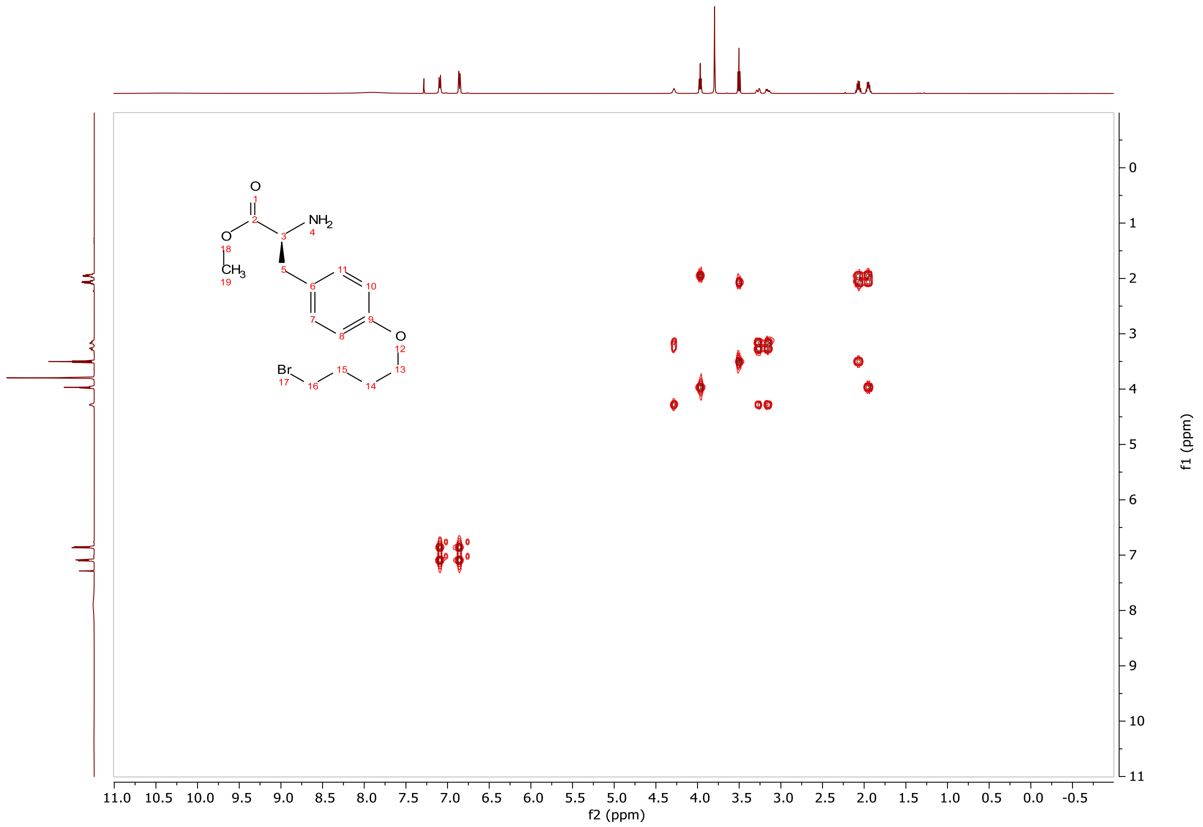

O
NH2
O
CH3
Br
O
f1 (ppm)
f2 (ppm)

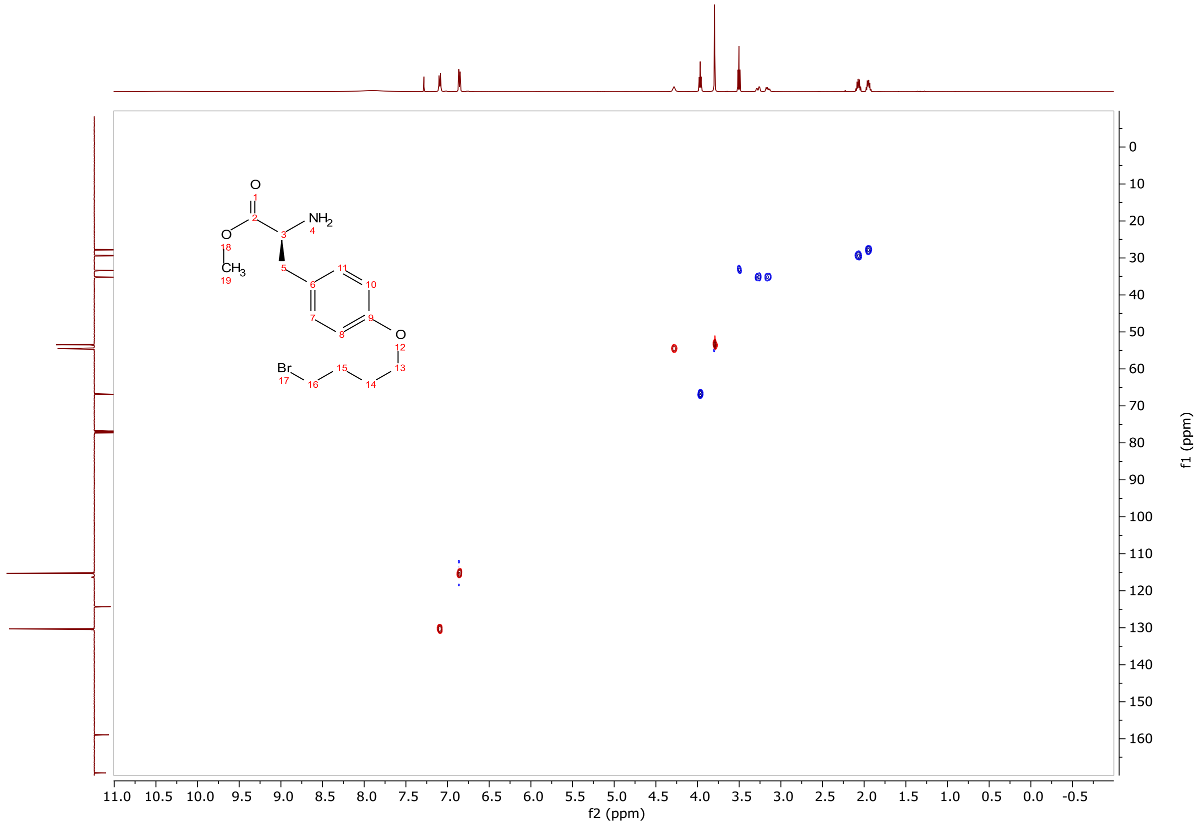

O
NH2
O
CH3
Br
O
f1 (ppm)
f2 (ppm)

2-amino-3-(4-(4-bromobutoxy)phenyl)propanoic acid hydrochloride- 1n

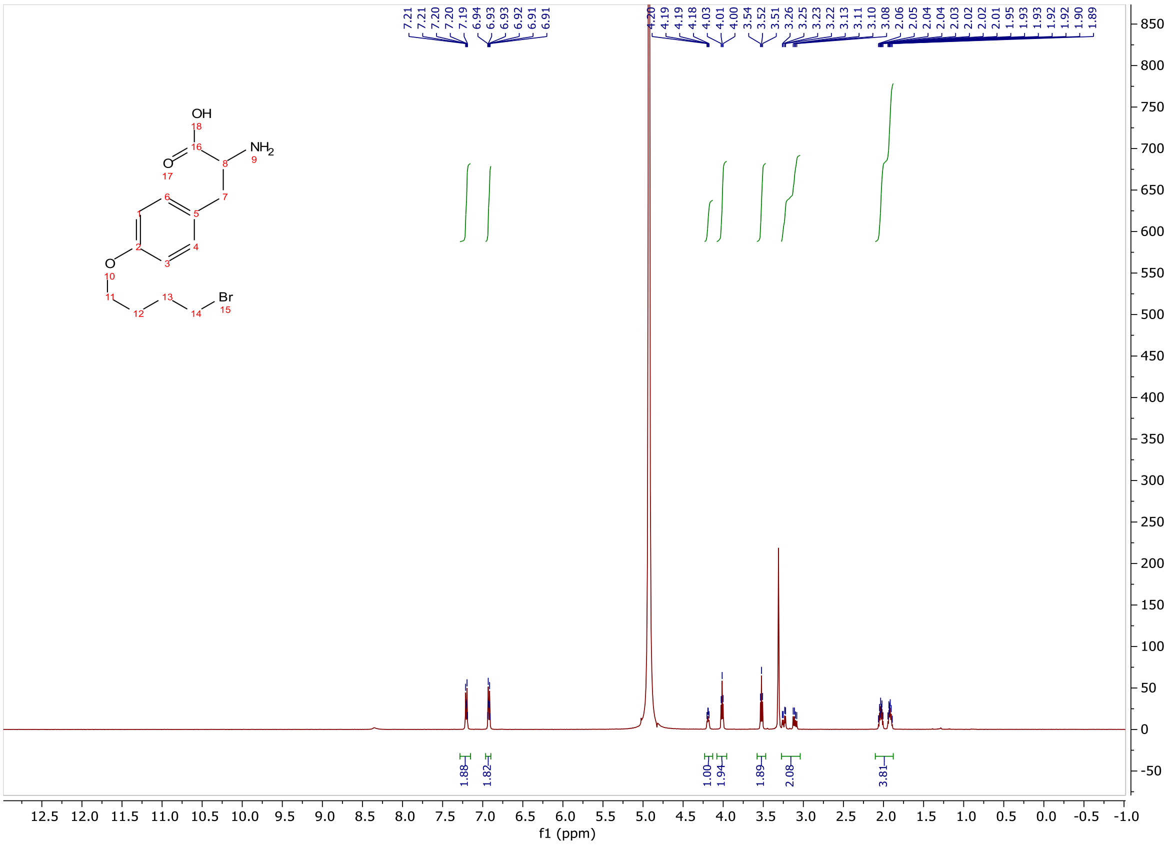

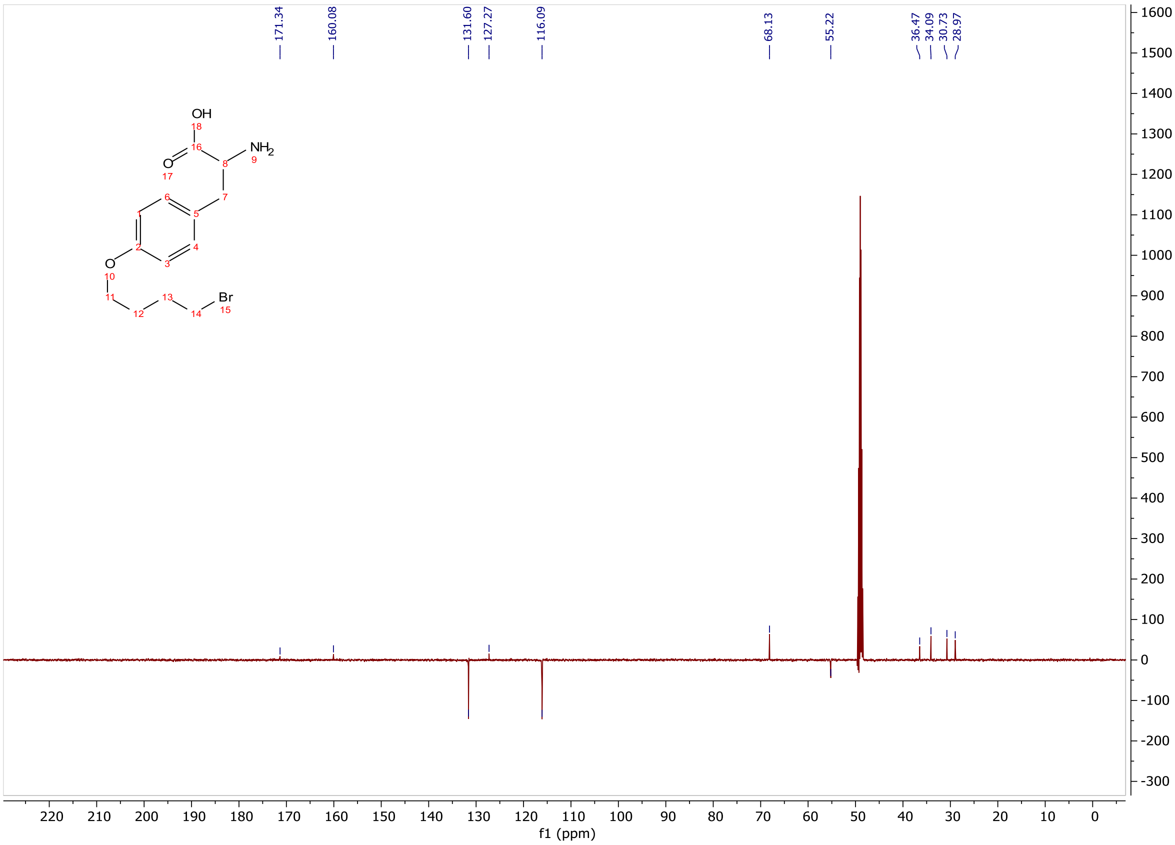

7-(4-bromobutoxy)-2H-chromen-2-one- 1i

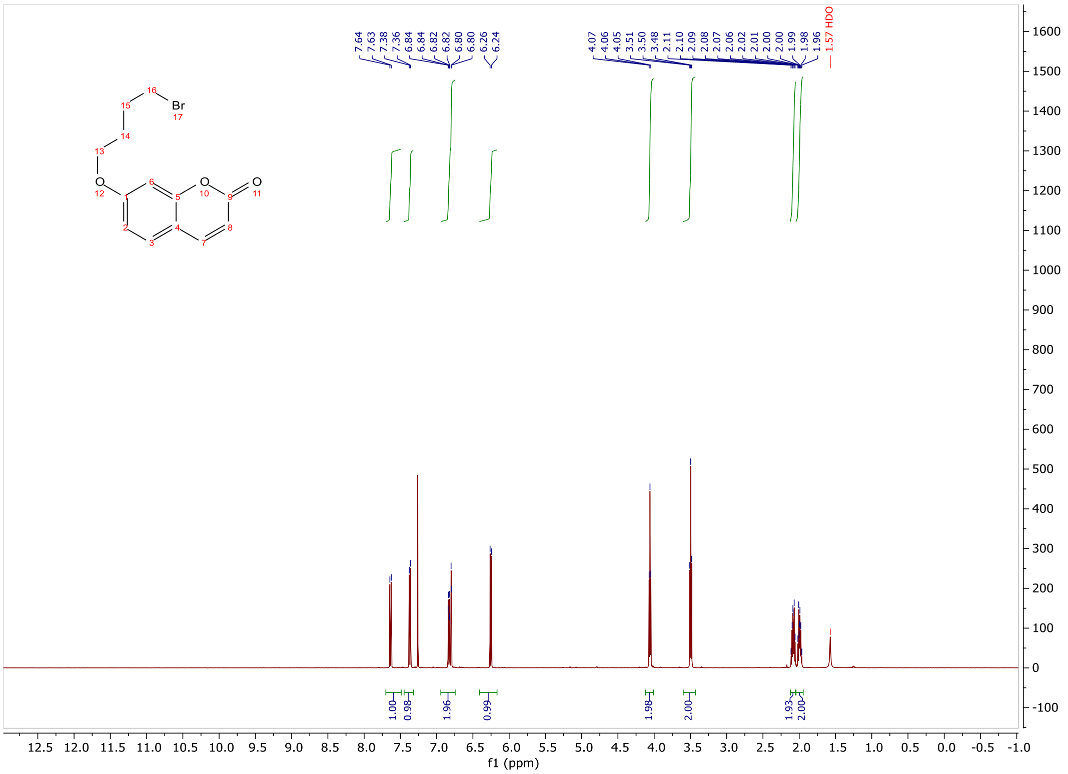

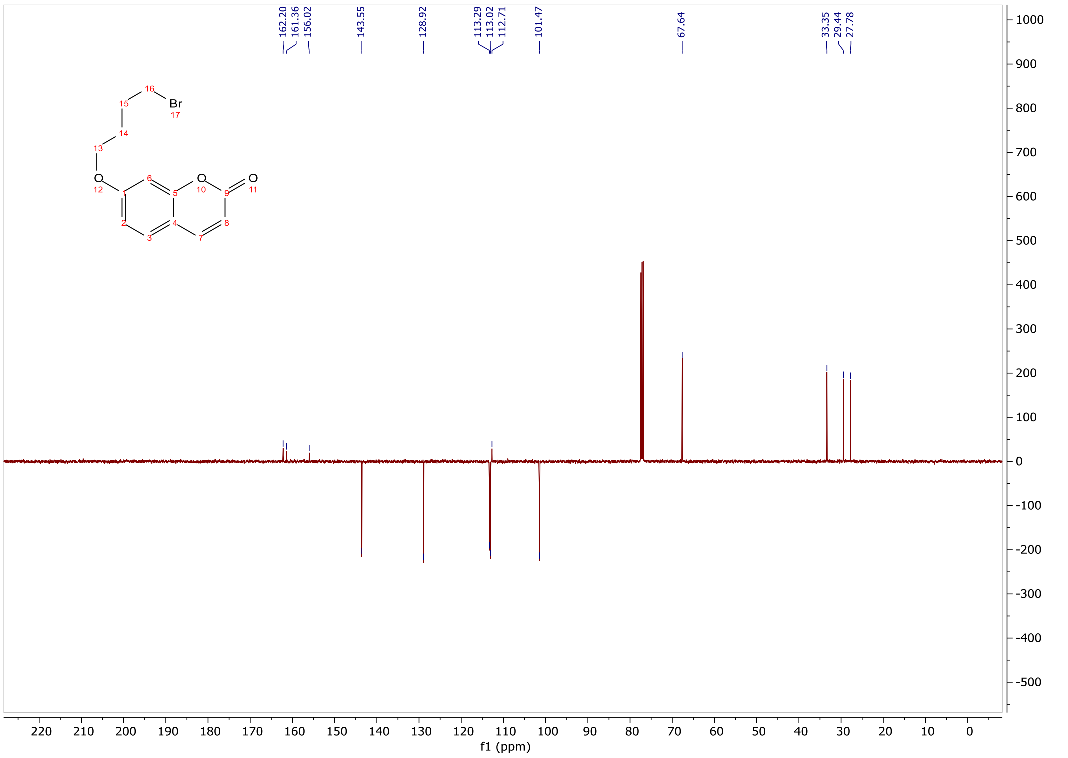

Methyl (2-amino-8-bromooctanoyl)-L-phenylalaninate- 1m

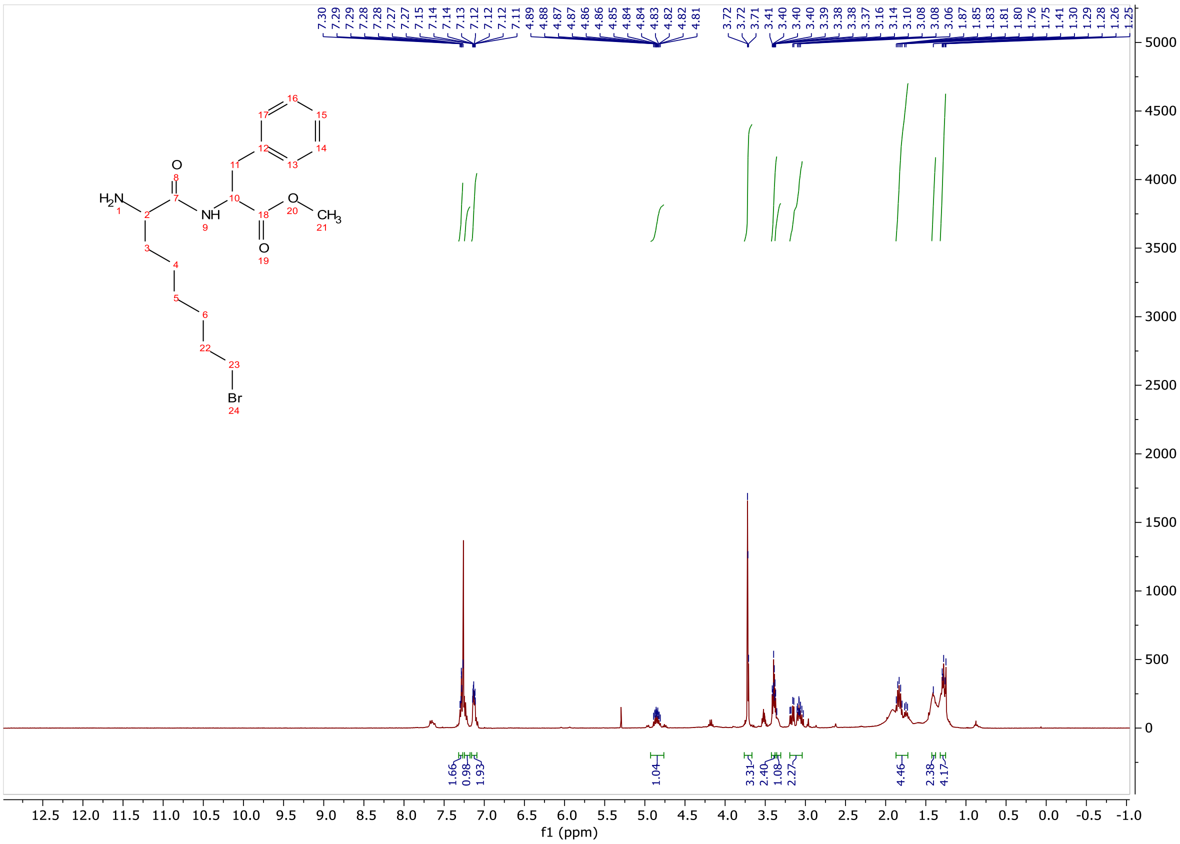

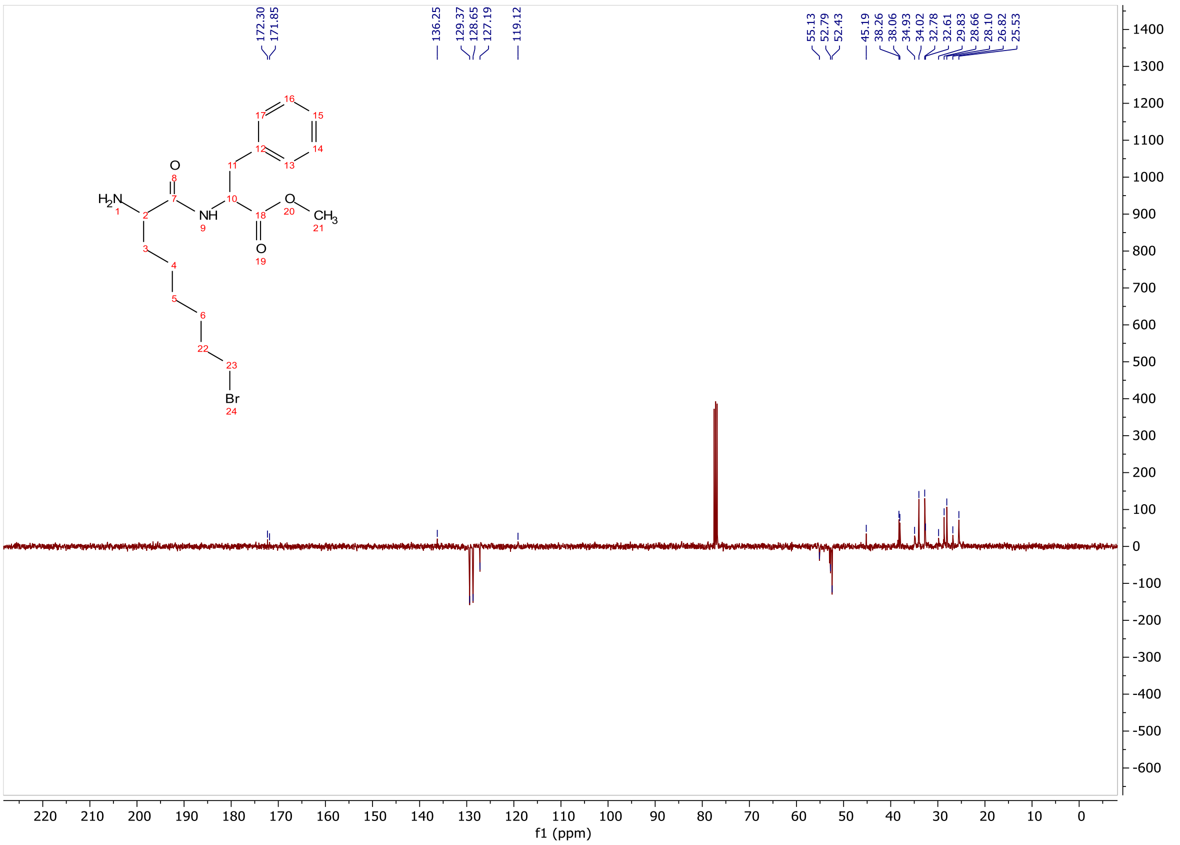

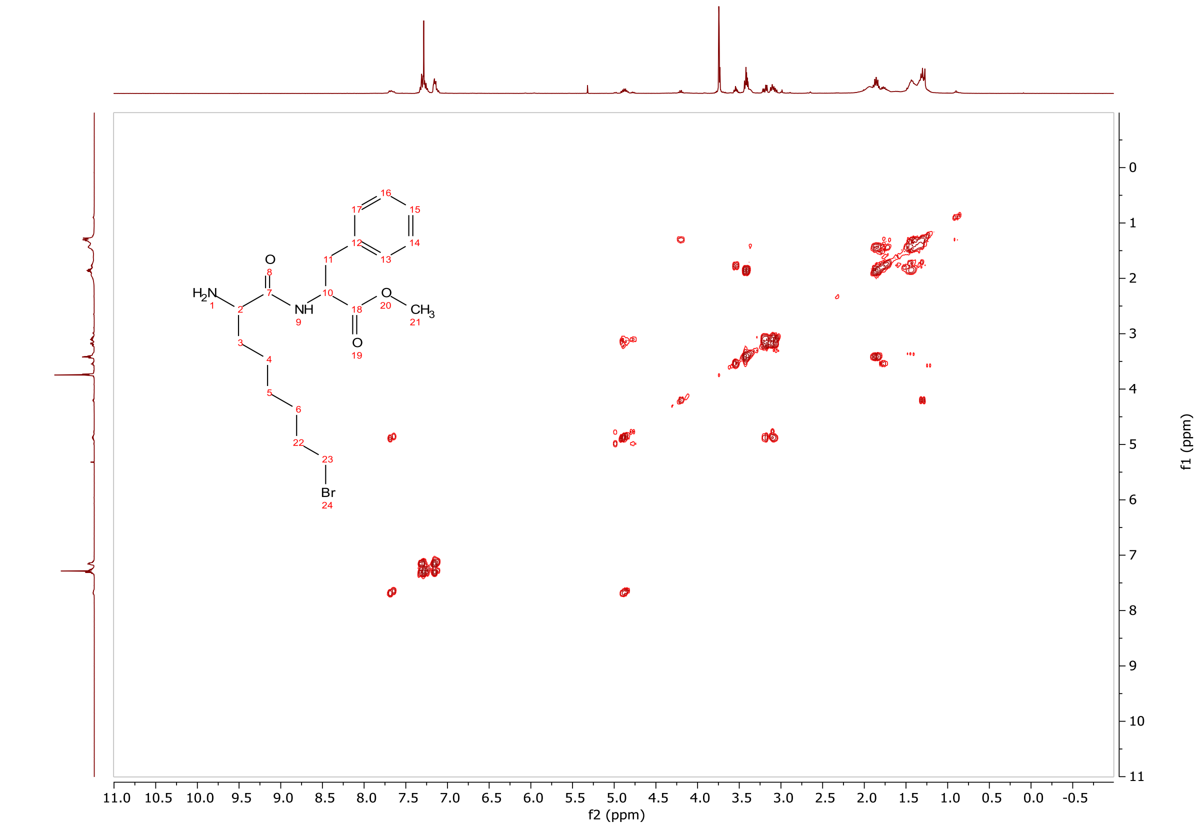

f1 (ppm)
f2 (ppm)

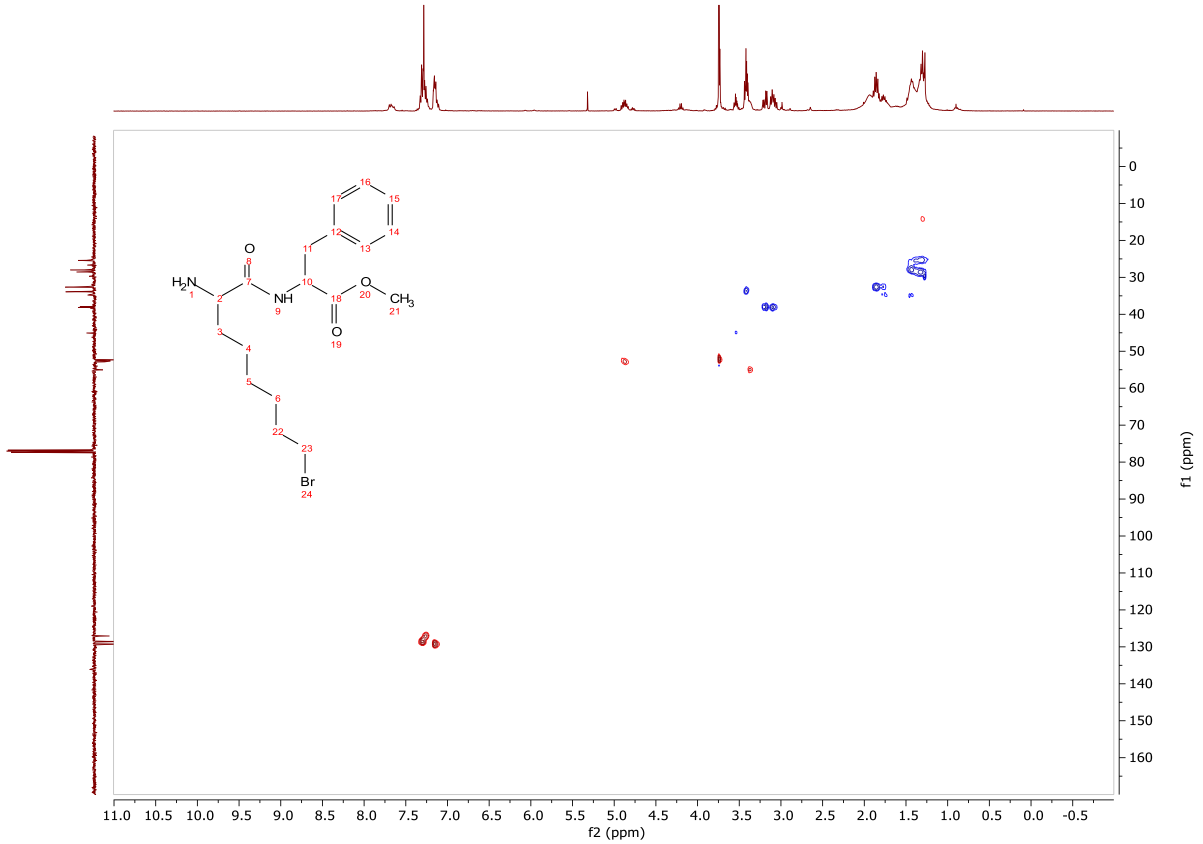

f1 (ppm)
f2 (ppm)

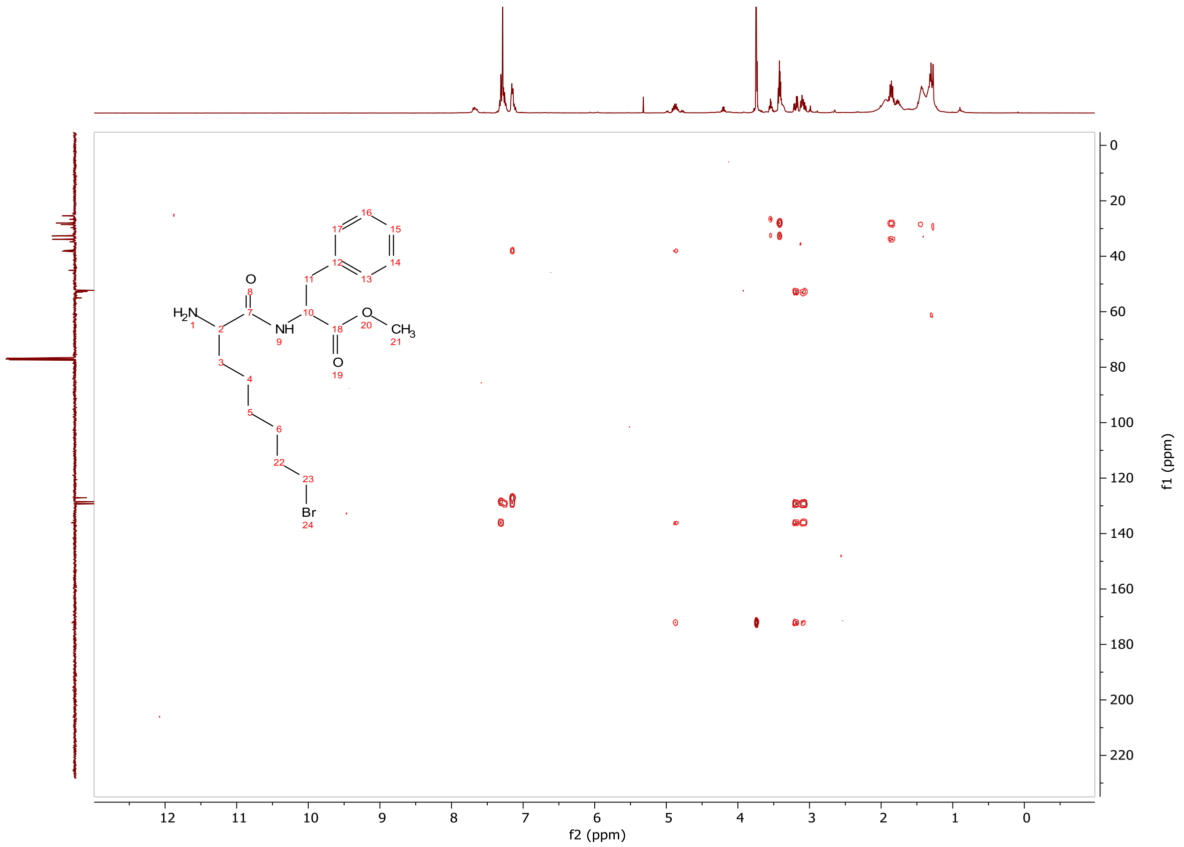

H2N
NH
O
O
CH3
Br
f2 (ppm)
f1 (ppm)

1-(2-bromoethyl)indoline-2,3-dione- 1j

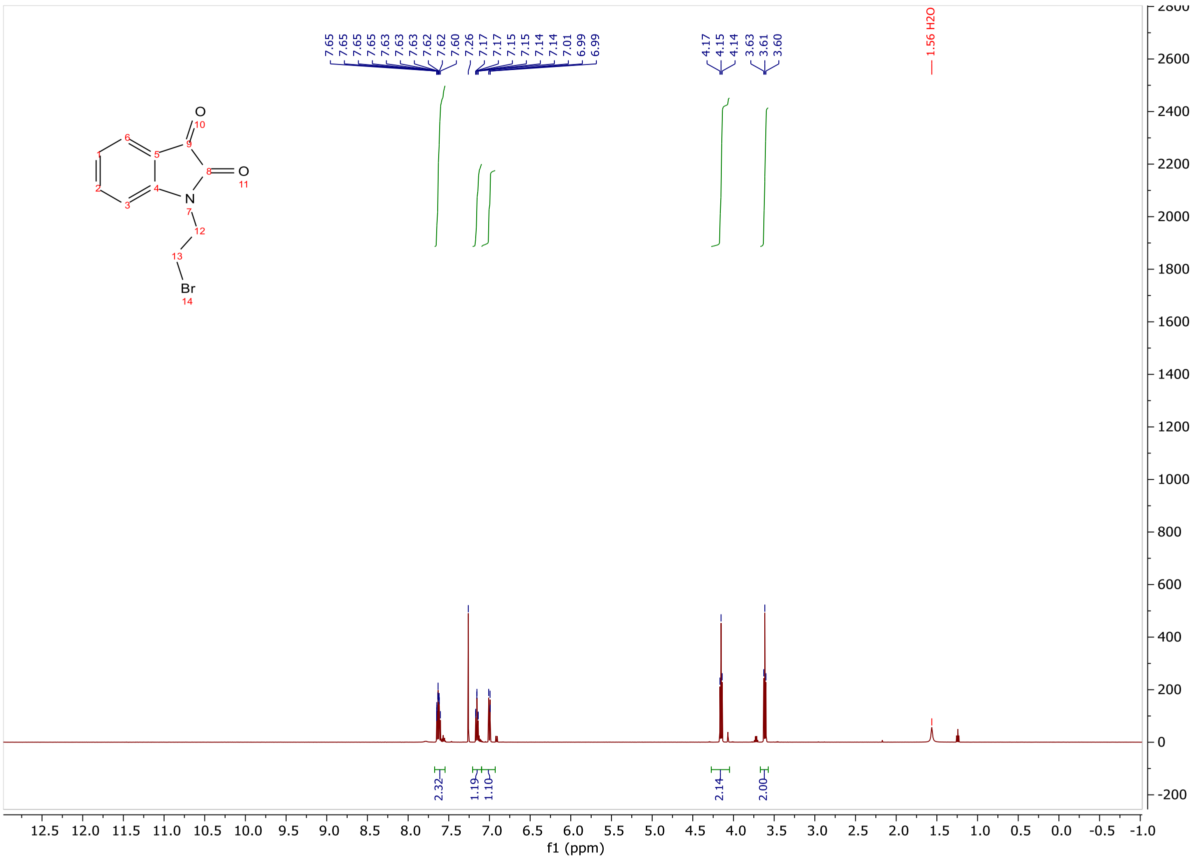

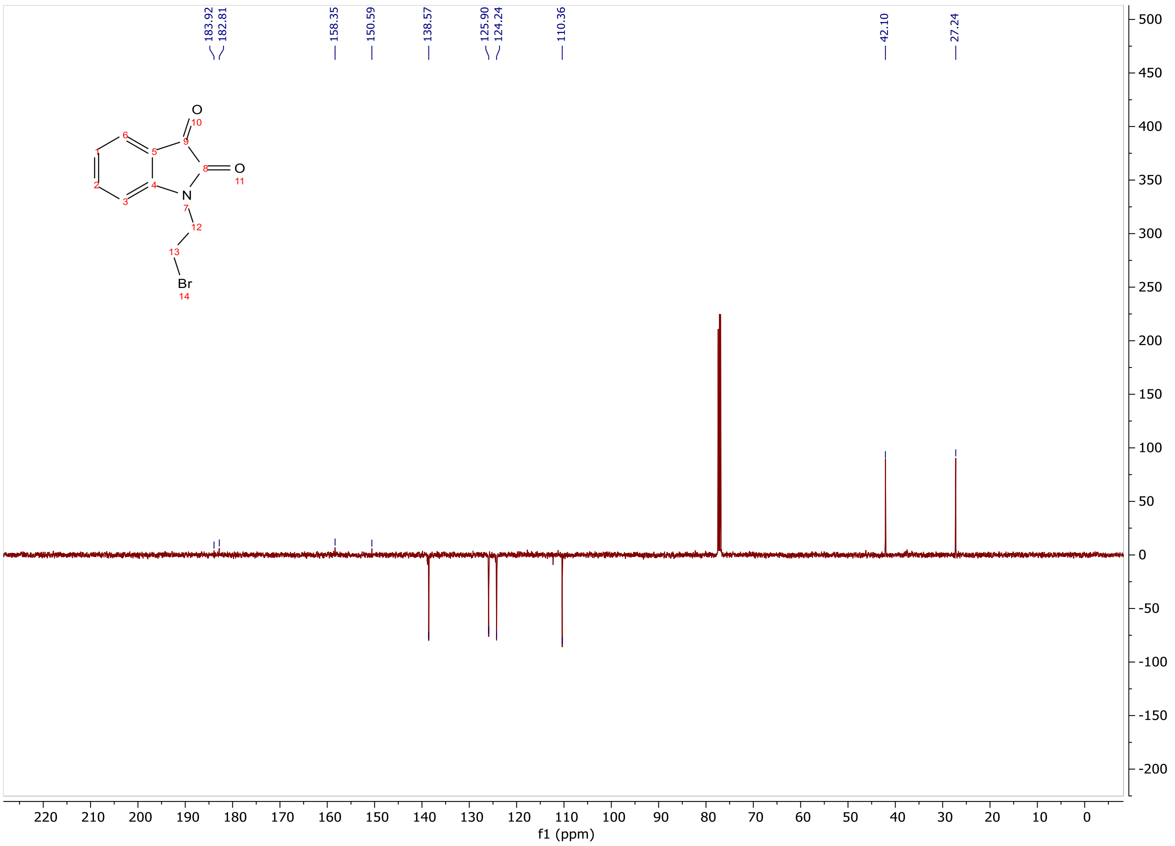

N-(2-ethyl-6-methylphenyl)-2-iodo-N-(1-methoxypropan-2-yl)acetamide- 1k

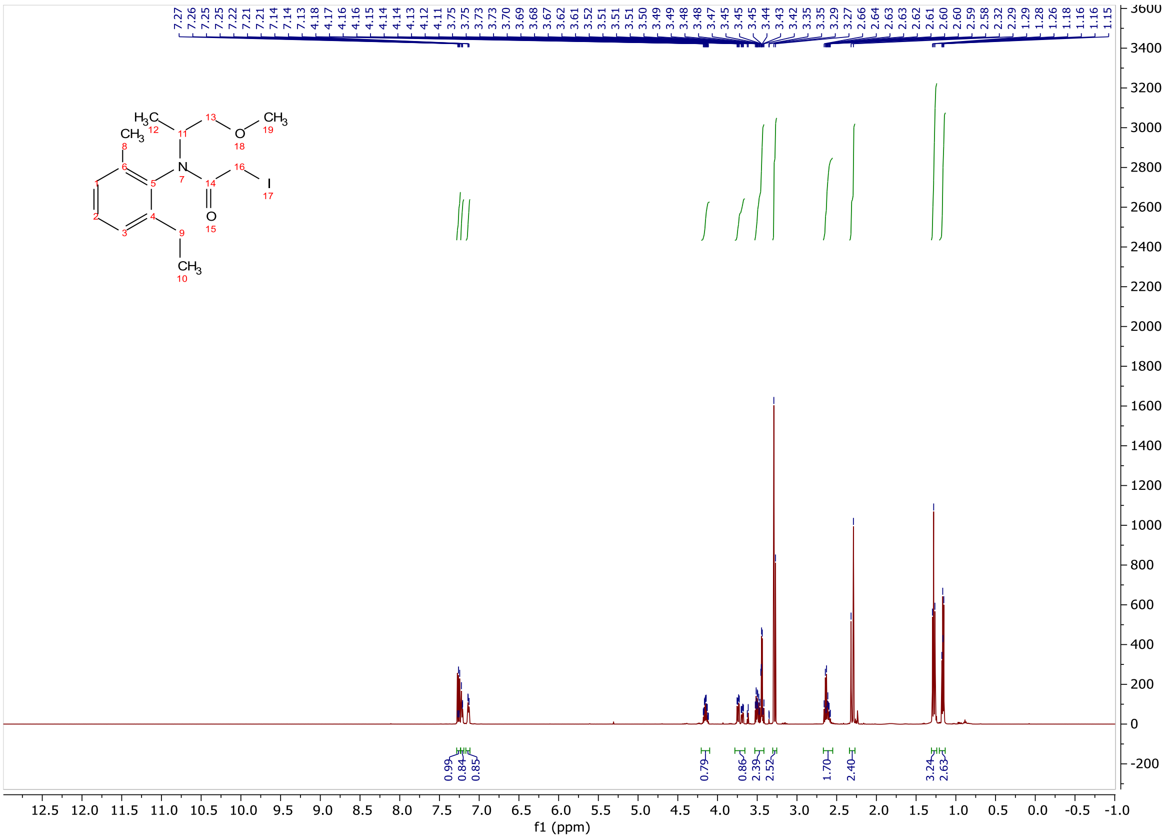

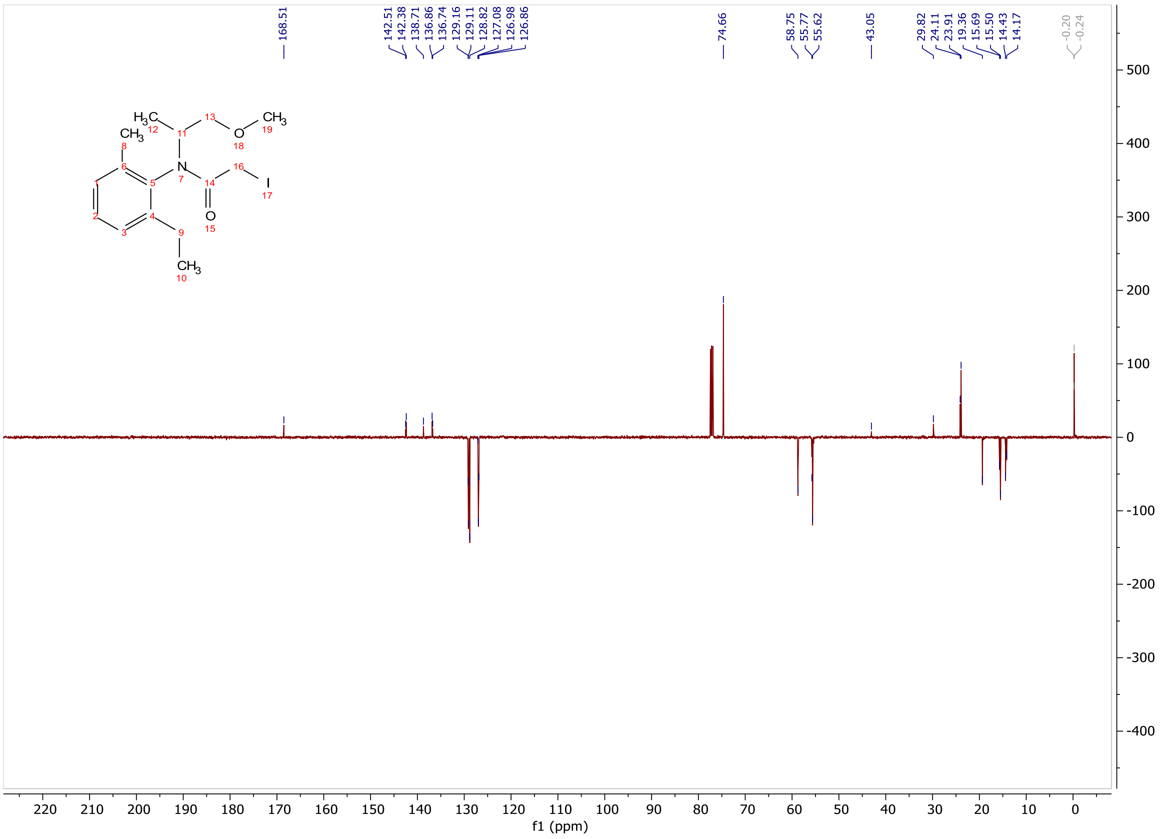

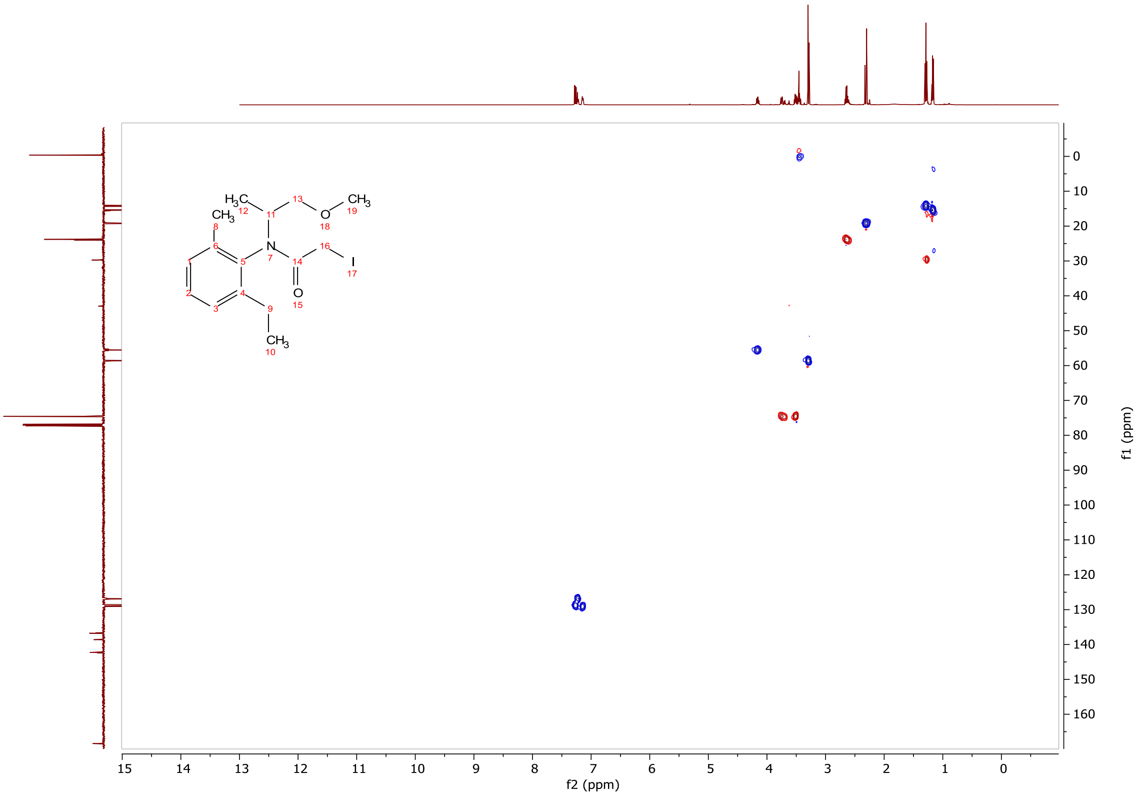

H3C
12
11
13
O
18
CH3
19
CH3
8
6
N
7
5
14
16
I
17
2
4
O
15
3
9
CH3
10
f2 (ppm)
f1 (ppm)

2-amino-8-hydroxyoctanoic acid- 1a-OH

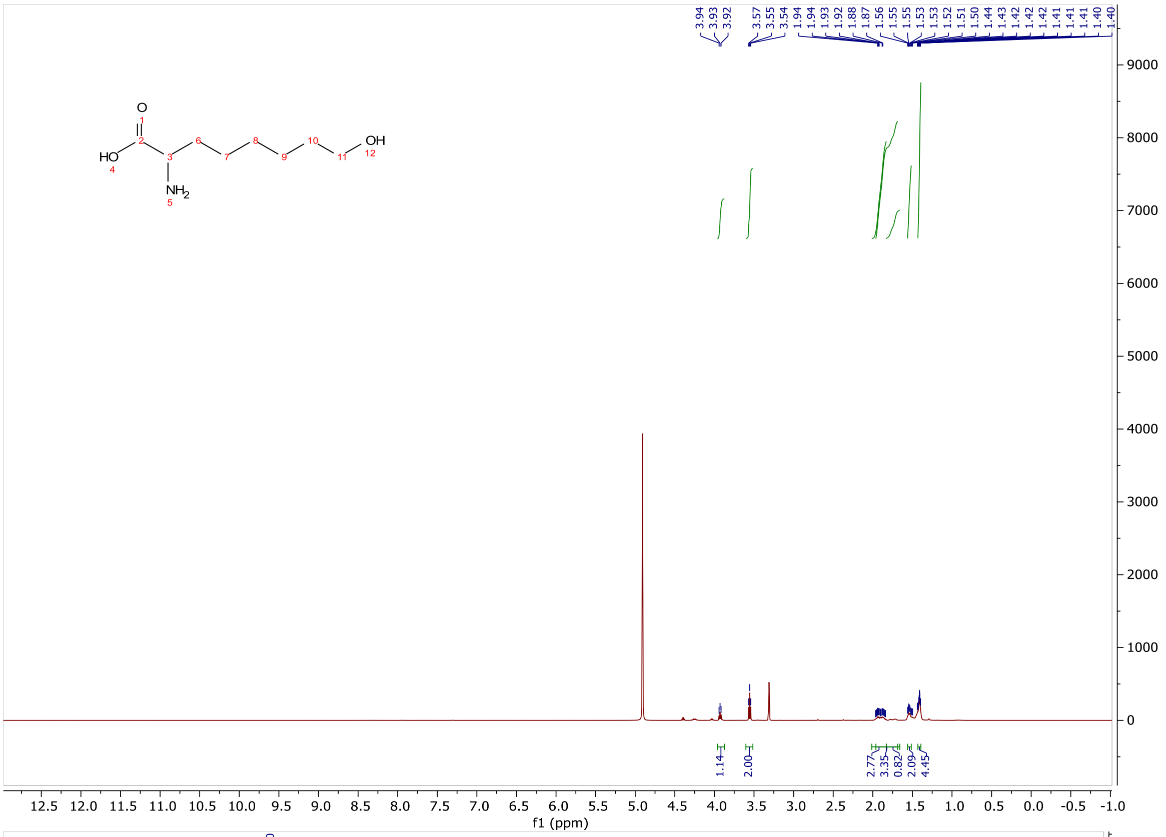

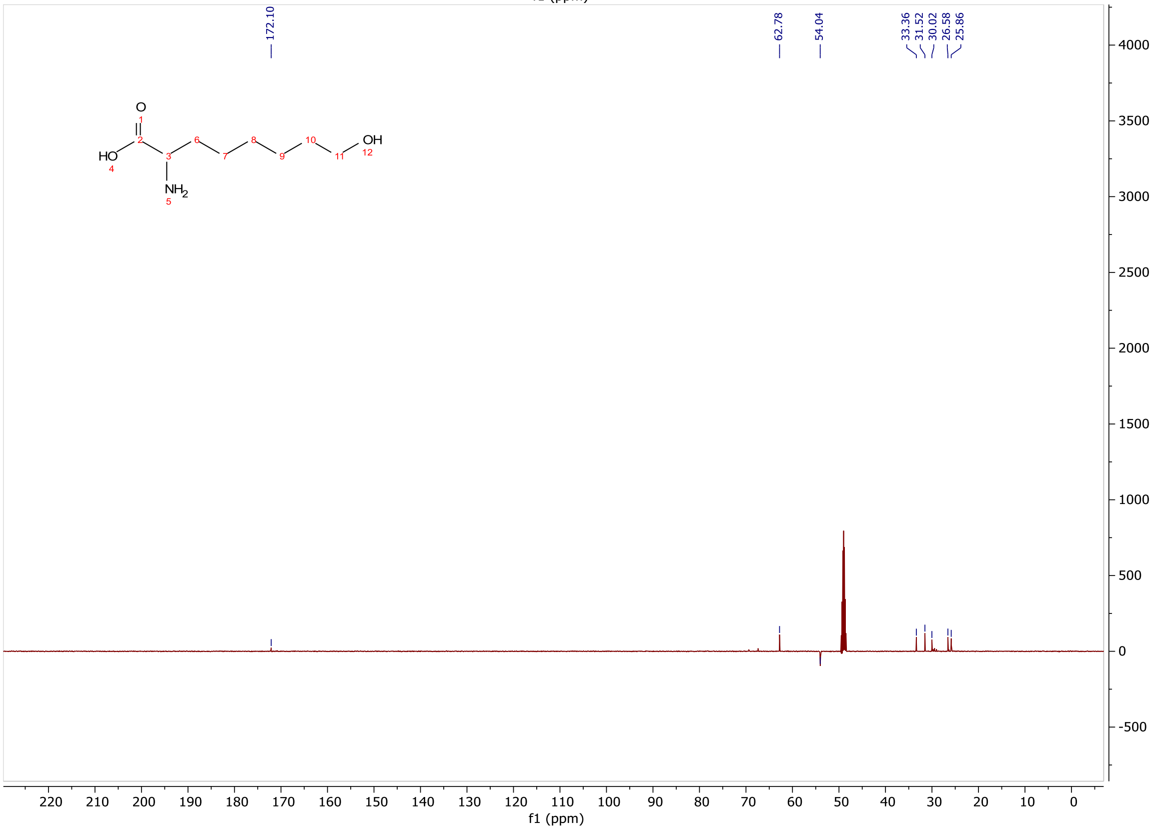

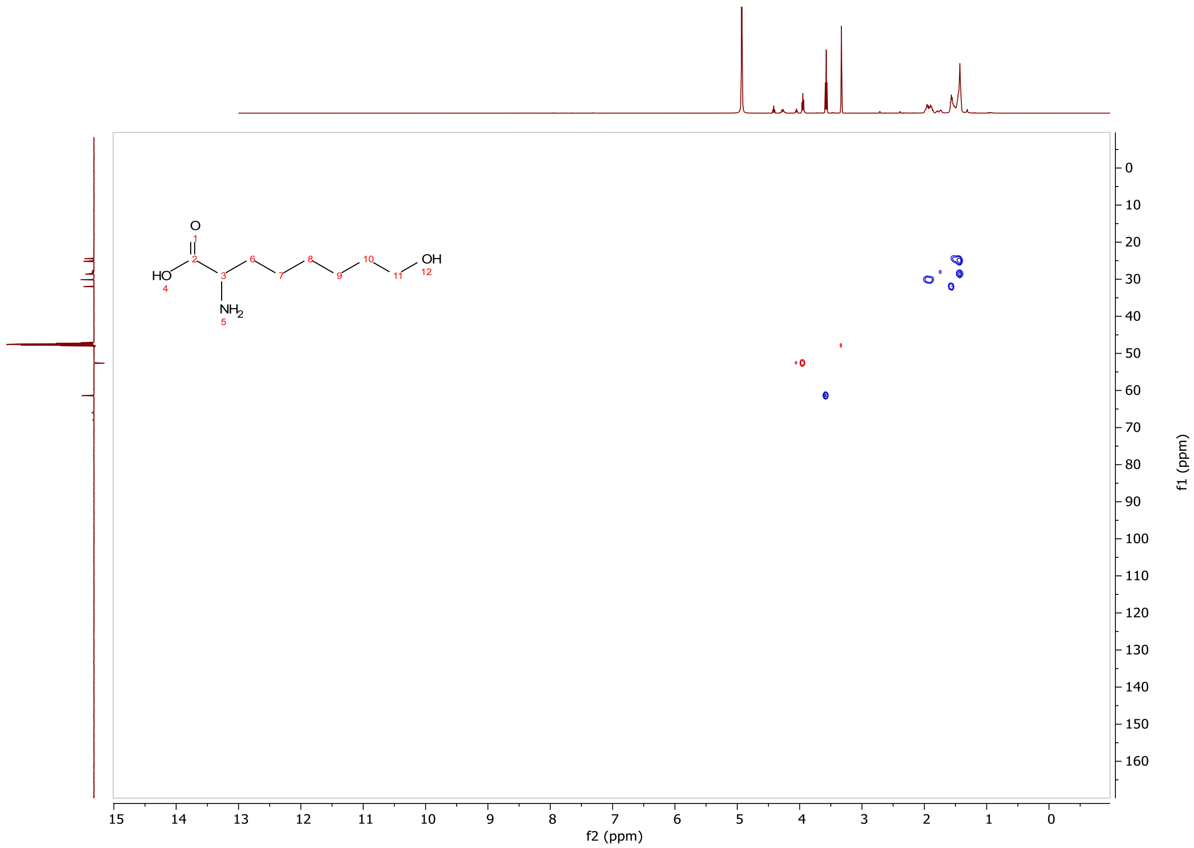
O
HO
NH2
OH
f1 (ppm)
f2 (ppm)

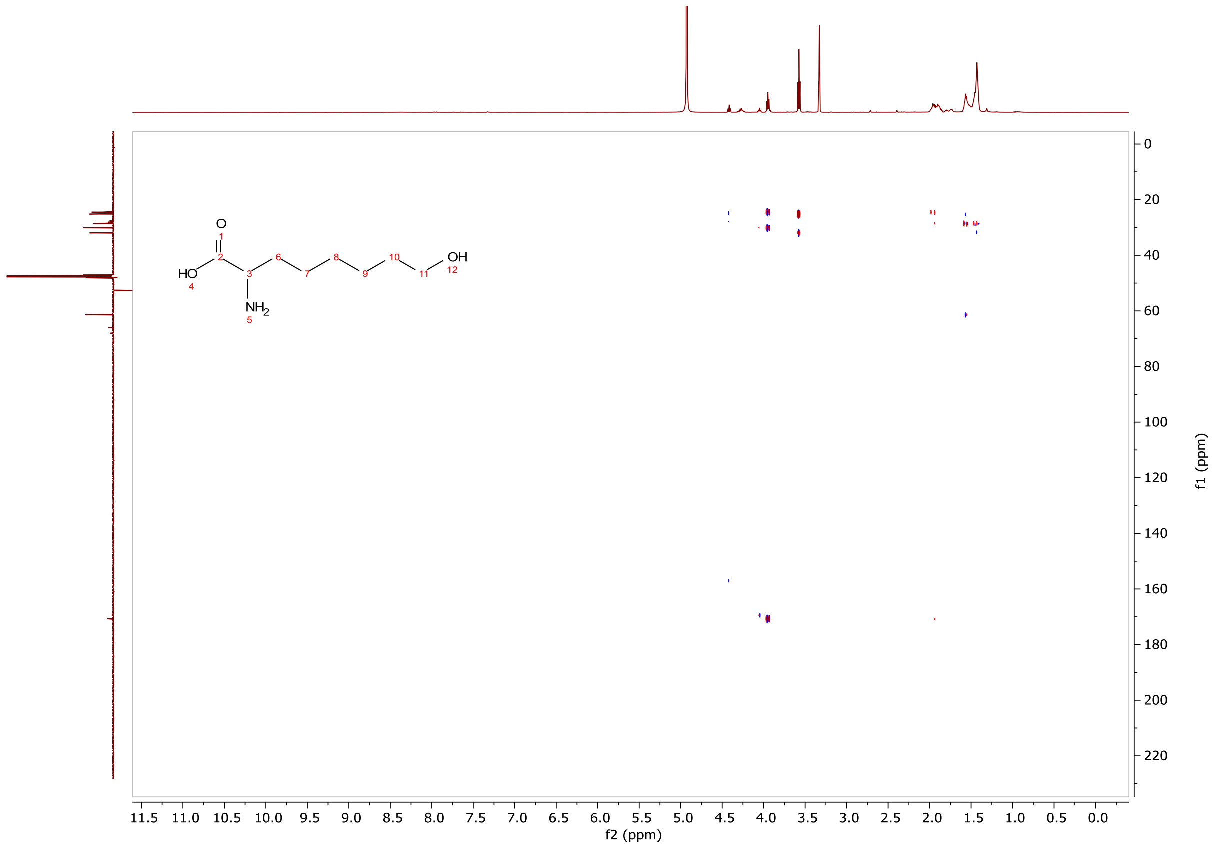
O
HO
NH2
OH
f1 (ppm)
f2 (ppm)
